# One-Pot, Telescoped Alkenylation of Amides via Stable Tetrahedral Intermediates as Lithium Enolate Precursors

**DOI:** 10.1021/acs.orglett.3c01269

**Published:** 2023-05-23

**Authors:** Simone Ghinato, Carolina Meazzo, Federica De Nardi, Andrea Maranzana, Marco Blangetti, Cristina Prandi

**Affiliations:** Dipartimento di Chimica, Università di Torino, Via Pietro Giuria 7, I-10125 Torino, Italy

## Abstract

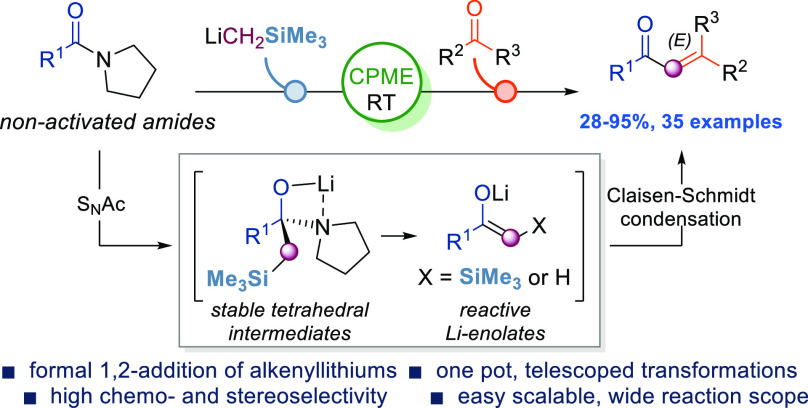

A mild and efficient telescoped procedure for the stereoselective alkenylation of simple, non-activated amides using LiCH_2_SiMe_3_ and carbonyl compounds as surrogates of alkenyllithium reagents is reported. Our methodology relies on the formation of stable tetrahedral intermediates, which, upon collapse into highly reactive lithium enolates in a solvent-dependent fashion, allows for the assembly of α,β-unsaturated ketones in a single synthetic operation with high stereoselectivity.

Amides are excellent substrates for the chemoselective assembly of ketones by direct 1,2-nucleophilic addition.^1^ To this aim, several chemoselective strategies have been developed to overcome the inertness of the amide C–N bond toward nucleophiles.^2^ However, there is still an urgent need to develop new bench-stable reagents for the high chemoselective transformation of amides using cheap and readily available synthons. In this context, the conversion of amides into α,β-unsaturated ketones by direct nucleophilic addition of unsaturated organometallic reagents has received scant attention, and only a few examples of alkenylation of amides using non-stabilized vinyllithium reagents have been described.^3^ Additionally, among the several methods developed for the preparation of chalcones (including Claisen–Schmidt condensation,^4^ cross-coupling approaches,^5^ Friedel–Crafts acylation,^6^ photo-Fries rearrangement,^7^ and catalytic one-pot synthesis from alcohols and ketones^8^), the transformation of benzamides into chalcones by nucleophilic addition of β-styrenyllithiums remains hitherto unexplored. One possible explanation for this is the cumbersome methods often required for the preparation of alkenyllithiums. Although these reagents are stable and could be conveniently handled at room temperature,^9^ their generation from alkenes by metalation, X/Li exchange, or reductive lithiation is often performed at low temperatures to avoid competitive elimination processes or configurational instability issues, using lithium metal or highly pyrophoric alkyllithiums under strictly controlled Schlenk conditions.^10^ To overcome these issues, one-step methods based on the direct addition of α-alkoxyvinyllithiums^11^ (Figure 1A, i), Grignard reagents (Figure 1A, ii) to both activated^12^ and non-activated^13^ amides, or weaker nucleophiles (alkenylcerium^14^ and olefins^15^) to secondary amides upon electrophilic activation (Figure 1A, iii) as well as a lithium diisopropylamide (LDA)-promoted Claisen–Schmidt condensation approach on salicylamides (Figure 1A, iv)^16^ have been developed. The formal alkenylation of amides using organometallic reagents could however be realized resorting to canonical multistep approaches proceeding via isolated ketone intermediates.^17^

**Figure 1:**
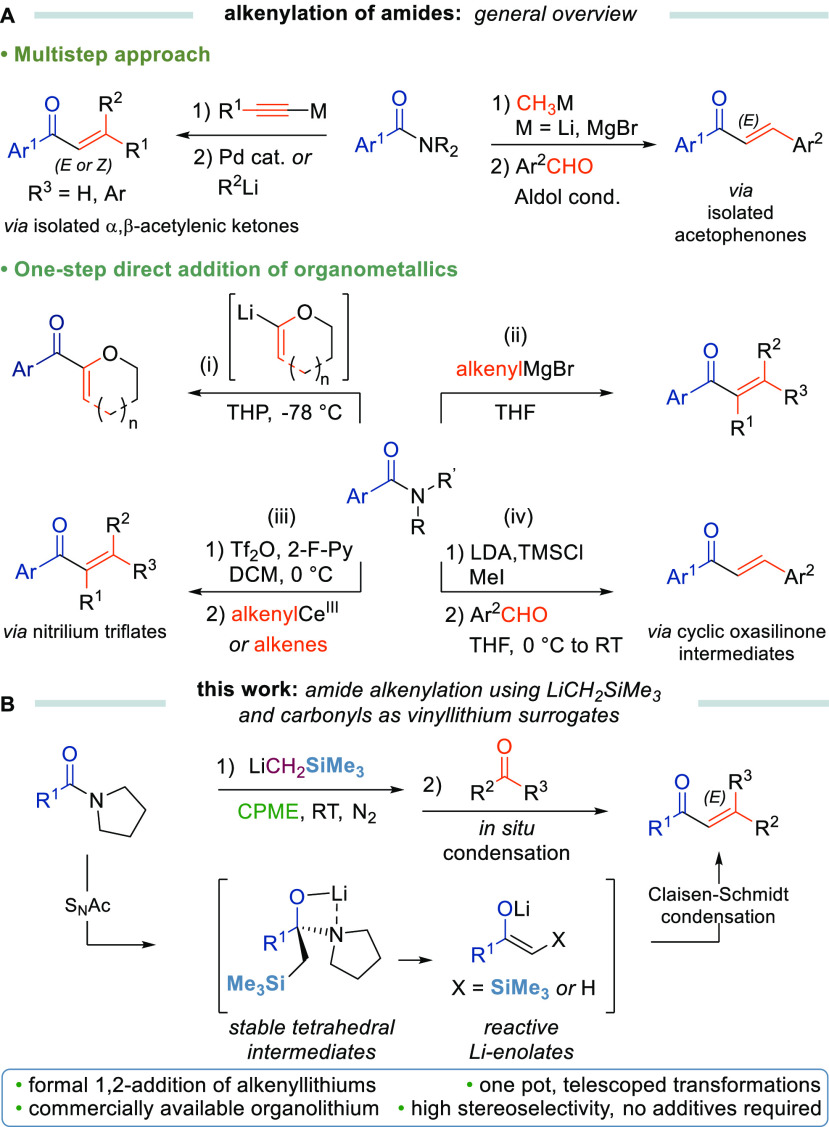
State of the art of the alkenylation of amides.

In the course of our studies on the reactivity of *s*-block polar organometallic reagents under bench-type aerobic conditions,^18^ we reported a general chemoselective route to ketones from amides using non-activated *N*-acylpyrrolidines as privileged acylating agents of organolithiums.^19^ The notorious overaddition reaction was effectively suppressed, owing to the stabilizing effect of the reaction medium [cyclopentyl methyl ether (CPME)] on the dimeric tetrahedral intermediates. On these grounds, we envisioned that the addition of α-silylated organolithium to a simple amide could mediate the generation of a stable tetrahedral intermediate in CPME, which could be exploited as a transient nucleophile upon collapse to promote a Claisen–Schmidt-type olefination process in the presence of a carbonyl source.

We thus herein report a systematic study on the synergic combination of LiCH_2_SiMe_3_ (a canonical synthon for olefination reactions)^20^ and carbonyl compounds to telescope the transformation of simple, non-activated amides into α,β-unsaturated ketones (Figure 1B). This protocol allows for the high (*E*)-stereoselective alkenylation of amides avoiding the preparation/use of unsaturated organolithiums, working under mild reaction conditions and in the absence of additives typically required to suppress the formation of disproportionation byproducts.^21^

We started our preliminary investigations using amide **1a** as a model substrate. On the basis of our previous results, a solution of compound **1a** (0.2 mmol, 0.04 M) in CPME was reacted with a commercially available solution of LiCH_2_SiMe_3_ (0.7 M in hexanes, 1.0 equiv) at room temperature (RT) (entry 1 in Table 1). After 30 min, the reaction was quenched with benzaldehyde (1.2 equiv), releasing in 1 h the desired chalcone **2a** in 35% yield and complete (*E*) stereoselectivity. Pleasingly, increasing the amount of LiCH_2_SiMe_3_ significantly improved the yield of compound **2a** without affecting the stereoselectivity of the condensation step (entries 2 and 3), with optimal results using 1.5 equiv of organolithium (entry 2). Less satisfactory results were obtained increasing the amount of benzaldehyde (entry 4), the reaction time (entry 5), or the temperature (entry 6). Performing the alkenylation reaction in the presence of quinuclidine or LiCl as additives releases the target chalcone **2a** in 57 and 53% yields, respectively (Table S1 of the Supporting Information). The use of solvents with higher coordinating ability,^22^ such as tetrahydrofuran (THF) (entry 7) and its greener alternative 2-MeTHF (entry 8), was slightly less effective in promoting the reaction. As expected, comparable results in THF and 2-MeTHF were obtained when the analogous Weinreb amide of compound **1a** (*N*,4-dimethoxy-*N*-methylbenzamide) was chosen as the substrate (entries 10 and 11), thus confirming our previous findings on the efficacy of non-chelating *N*-acylpyrrolidines as chemoselective acylating agents.^19^ Noteworthy, the title alkenylation reaction could be performed under aerobic conditions (entry 12), however with slightly lower yields, owing to the non-negligible competitive protonolysis of organolithium occurring over prolonged reaction times. In this case, the use of highly hydrophobic CPME is essential to prevent the moisture-induced protonolysis process. The use of more hygroscopic solvents (CPME < 2-MeTHF ≪ THF) led to a progressive decrease of the reaction yield (Table S1 of the Supporting Information).

**Table 1:**
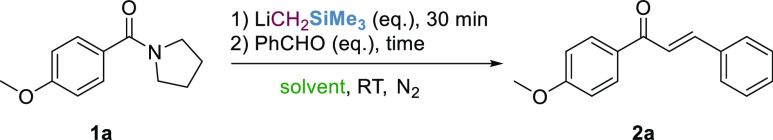
Alkenylation of *N*-Acylpyrrolidine **1a** under Different Conditions^a^

entry	solvent	LiCH_2_SiMe_3_ (equiv)	PhCHO (equiv)	time (h)	compound **2a** (%)^b^
1	CPME	1.0	1.2	1	35
2	CPME	1.5	1.2	1	67^c^
3	CPME	2.0	1.2	1	52
4	CPME	1.5	2.0	1	62
5	CPME	1.5	1.2	12	49
6	CPME	1.5	1.2	1	59^d^
7	THF	1.5	1.2	1	60
8	2-MeTHF	1.5	1.2	1	57
9	CPME	1.5	1.2	1	40^e^
10	THF	1.5	1.2	1	67^e^
11	2-MeTHF	1.5	1.2	1	48^e^
12	CPME	1.5	1.2	1	51^f^

a Reaction conditions: compound **1a** (0.2 mmol), LiCH_2_SiMe_3_ (0.7 M in hexanes), solvent (5.0 mL), and RT.

b Determined by quantitative ^1^H nuclear magnetic resonance (NMR) using heptane as the internal standard. *E*/*Z* ratio > 99:1 (by ^1^H NMR).

c At a 65% isolated yield (see the Supporting Information).

d Reaction run at 60 °C.

e *N*,4-dimethoxy-*N*-methylbenzamide (Weinreb amide) was used as the substrate.

f Reaction run under air.

With satisfactory conditions in place, the scope and limitations of this transformation were evaluated for a series of functionalized amides (Scheme 1). The alkenylation of *N*-acylpyrrolidines **1** proceeded smoothly *en route* to a variety of substituted chalcones bearing electron-donating (**2c**–**2e**), fluorinated (**2f** and **2g**), naphthalene (**2h**), and heteroaromatic (**2l** and **2m**) groups with moderate to good yields (28–80%). Our methodology also allowed (a) the chemoselective preparation of highly conjugated chalcones **2i** and **2j** and (b) the simultaneous alkenylation of two amide groups (**2k**) by simply increasing the amount of LiCH_2_SiMe_3_ and carbonyl compound in a single synthetic operation. Remarkably, the preparation of chalcone **2b** has been easily scaled up to 5.7 mmol of compound **1b** (1 g) with comparable efficiency in terms of the yield and selectivity (69% versus 80% on a small scale). However, other sensitive functional groups, such as cyano-, nitro-, diazo-, bromine, and hydroxyl, were incompatible with the reaction conditions, affording complex reaction mixtures or recovery of the starting material after workup (see Table S2 of the Supporting Information).

**Scheme 1:**
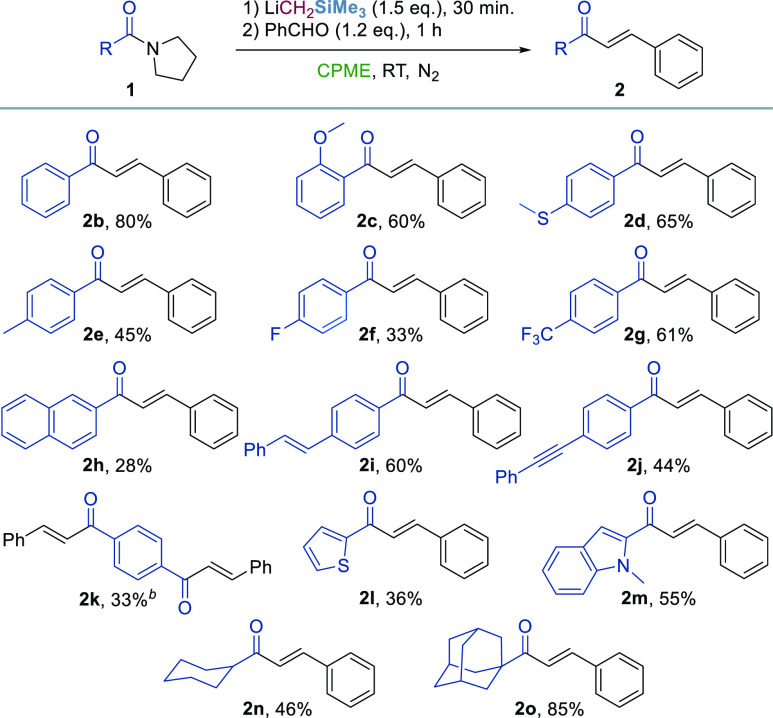
Amide Scope of the Reaction Reaction conditions: compound **1** (0.2 mmol), LiCH_2_SiMe_3_ (0.7 M in hexanes, 0.3 mmol), CPME (5 mL), 30 min, and RT, under N_2_ and then PhCHO (0.24 mmol), 1 h, and RT. A total of 0.6 mmol of LiCH_2_SiMe_3_ and 0.48 mmol of PhCHO were used. Reported yields refer to isolated products.

We next investigated the aldehyde scope of the reaction (Scheme 2). The methodology well tolerates the use of several electron-donating group (EDG)-substituted (**2p**–**2r** and **2v**) and halogenated (**2s** and **2t**) aromatic aldehydes, including iodinated derivatives (**2u**), which enable further functionalization strategies. Unsaturated, heterocyclic, and alicyclic aldehydes delivered the desired chalcones **2w**–**2aa** in 40–62% yield in a complete (*E*)-stereoselective fashion. Also, ketones could be efficiently employed as carbonyl partners (**2ab** and **2ac**); however, longer reaction times are required (see the Supporting Information for details).

**Scheme 2:**
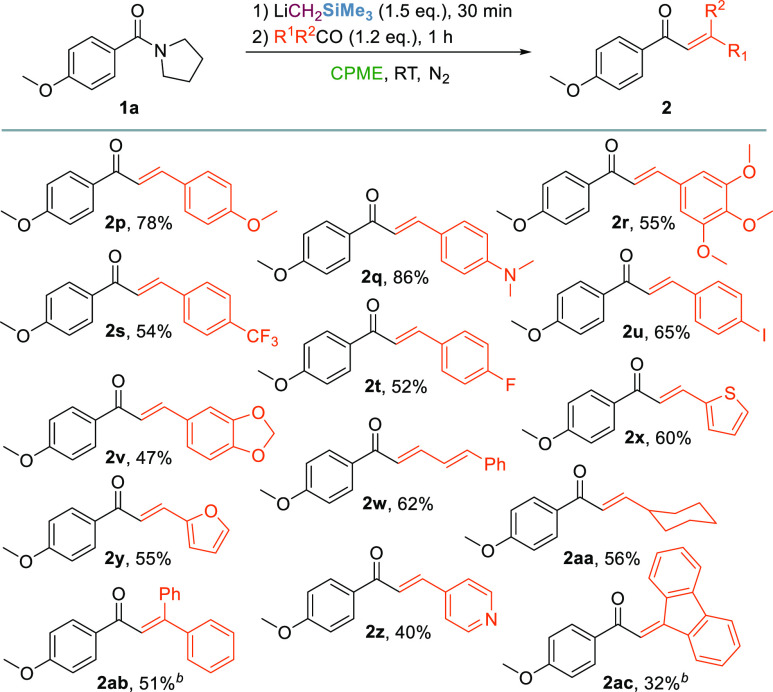
Carbonyl Scope of the Reaction Reaction conditions: compound **1a** (0.2 mmol), LiCH_2_SiMe_3_ (0.7 M in hexanes, 0.3 mmol), CPME (5 mL), 30 min, and RT, under N_2_ and then R^1^R^2^CO (0.24 mmol), 1 h, and RT. Reaction time = 2.5 h after the addition of ketone. Reported yields refer to isolated products.

Owing to the large potential of the chalcone scaffold in drug discovery,^4^ we then applied our alkenylation conditions for the preparation of selected chalcones with prominent pharmacological applications (Scheme 3). Pleasingly, a series of chalcones with potential biological activity for the treatment of cancer (**2ad** and **2af**),^23^ microbial infections (**2ag**),^24^ chronic myeloid leukemia (**2ah**),^25^ and inflammations (**2ai**)^26^ or possessing enhanced fluorescent properties for bioimaging purposes (**2ae**)^27^ have been obtained starting from the properly substituted amides **1** and aldehydes in satisfactory yields (50–95%).

**Scheme 3:**
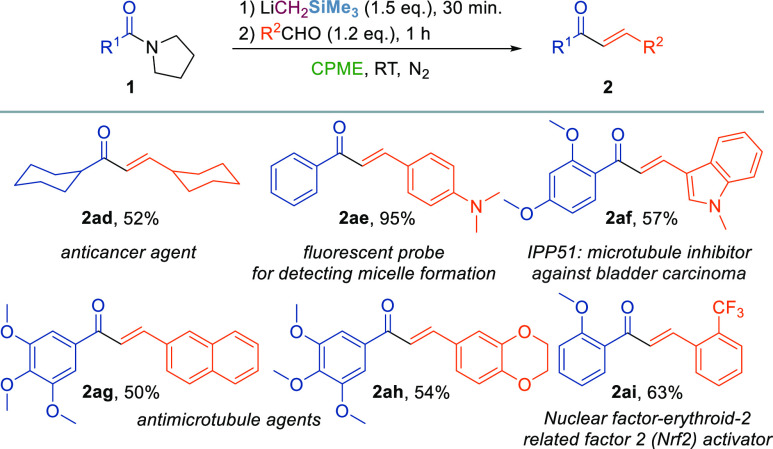
Synthesis of Biologically Relevant Chalcones Reaction conditions: compound **1** (0.2 mmol), LiCH_2_SiMe_3_ (0.7 M in hexanes, 0.3 mmol), CPME (5 mL), 30 min, and RT, under N_2_ and then aldehyde (0.24 mmol), 1 h, and RT. Reported yields refer to isolated products.

To gain more mechanistic insights into the nucleophilic acyl substitution (S_N_Ac)/condensation sequence, additional electrophilic quenching experiments were performed (Scheme 4A). As expected, treatment of amide **1a** with LiCH_2_SiMe_3_ (1.5 equiv) in CPME under optimized reaction conditions, followed by quenching with water (5 equiv), led to the exclusive formation of the α-silyl ketone **3a** (entry 1).

**Scheme 4:**
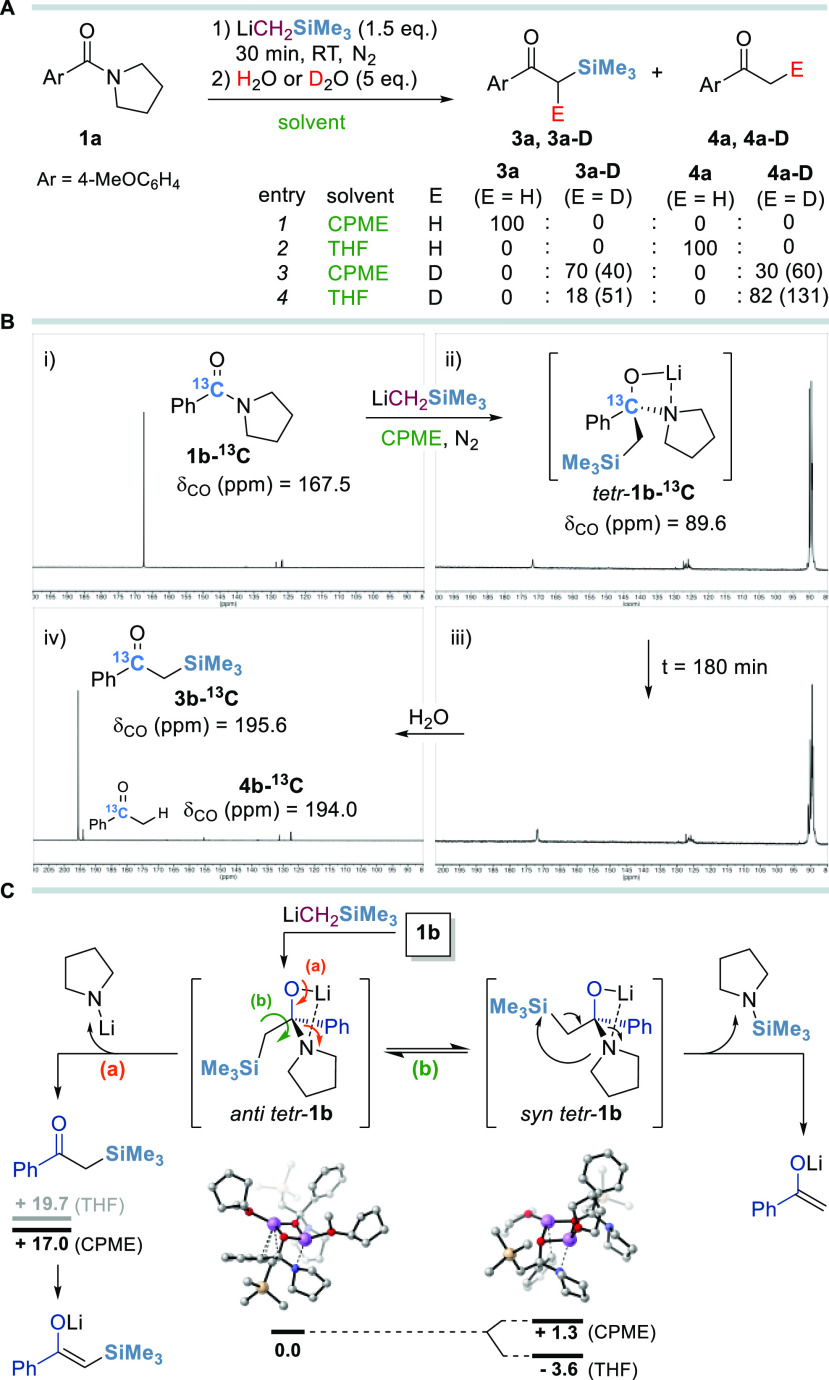
Mechanistic Insights into the S_N_Ac Step (A) Reaction conditions: compound **1a** (0.2 mmol), LiCH_2_SiMe_3_ (0.7 M in hexanes, 0.3 mmol), CPME or THF (5 mL), 30 min, and RT, under N_2_ and then H_2_O or D_2_O. Ratios are based on ^1^H NMR integration. Values in parentheses refer to the overall D incorporation (%) based on ^1^H NMR integration and confirmed with ^2^H NMR. (B) *In situ*^13^C NMR monitoring of the S_N_Ac reaction on labeled **1b**-^**13**^**C** in dry CPME. (C) Proposed reaction mechanism based on experimental data and reaction free energies (kcal mol^–1^, at 298 K) estimated by preliminary density functional theory (DFT) calculations [M06-2X/6-311+G(d) level; see the Supporting Information for details]. Dimeric aggregates in solution were used in the computations.^19^ For clarity, structures are represented as monomers and hydrogen atoms have been omitted.

Interestingly, performing the S_N_Ac step in THF led to the sole formation of acetophenone derivative **4a** lacking the SiMe_3_ group upon aqueous quenching (entry 2). Deuterium-labeling experiments afforded (a) α-deuterated α-SiMe_3_ ketone **3a**-**D** (40% D incorporation) when the reaction was performed in CPME (entry 3) and (b) deuterated acetophenone **4a**-**D** as a mixture of isotopomers with an overall 130% D incorporation using THF as the reaction medium (entry 4).^28^ These findings suggest the solvent-dependent formation of two different lithium enolates upon the addition of the electrophile to the reaction mixture, which can act as nucleophiles in a Claisen–Schmidt-type condensation in the presence of a carbonyl compound. To confirm that the formation of ketones **3a**–**4a** occurs only upon electrophilic quench, we next investigated the stability of the tetrahedral intermediate by ^13^C NMR analysis (Scheme 4B, i–iv). After 30 min from the addition of LiCH_2_SiMe_3_ (1.5 equiv) to a 0.12 M solution of ^13^C-labeled amide **1b**-^**13**^**C** (0.07 mmol, 1.0 equiv) in dry CPME under nitrogen, neither starting material nor ketones **3b**-^**13**^**C** or **4b**-^**13**^**C** were detected in the ^13^C NMR spectra. Evidence of the formation of the tetrahedral intermediate tetr-**1b**-^**13**^**C**, stable under these conditions up to 3 h, was assessed by a significant upfield shift of amide carbonyl to 89.6 ppm. The addition of a stoichiometric amount of water induced the rapid conversion of tetr-**1b**-^**13**^**C** into α-silyl ketone **3b**-^**13**^**C** (δ_CO_ = 195.6 ppm), alongside a negligible amount of acetophenone **4b**-^**13**^**C**. While the formation of the tetrahedral intermediate in the S_N_Ac step is undeniable, its solvent-dependent collapse into two different lithium enolates remains however unclear. Preliminary DFT calculations on the addition of LiCH_2_SiMe_3_ to amide **1b** revealed that tetr-**1b** exists as two conformations in which the SiMe_3_ group arranges in an *anti* position (*anti*-tetr-**1b**) or between a *gauche* and *syn* conformation (*syn*-tetr-**1b**) with respect to OLi (Scheme 4C). Interestingly, the relative stability of the *syn* conformation is higher in THF (−3.6 kcal mol^–1^ versus *anti*-tetr-**1b**), whereas the *anti* conformation is more stable in less coordinating CPME (−1.3 kcal mol^–1^ versus *syn*-tetr-**1b**). Hence, we are inclined to propose the initial LiCH_2_SiMe_3_ addition to amide to form the stable tetrahedral intermediate tetr-**1b**, which can equilibrate to the *anti* or *syn* conformation depending upon the reaction media. In CPME, the collapse of *anti*-tetr-**1b** upon electrophilic quench (path a) affords the α-SiMe_3_ ketone, which can be further deprotonated to corresponding C-silylated lithium enolate by the excess of LiCH_2_SiMe_3_ or the lithium amide leaving group, releasing a reactive nucleophile for a Claisen–Schmidt-type olefination process. In addition, the endergonic heterolytic dissociation of the C–N bond in the tetr-**1b** intermediate has slightly higher energy in THF than in CPME (19.7 and 17.0 kcal mol^–1^, respectively). In THF, an intramolecular elimination of *N*-SiMe_3_ pyrrolidine from the predominant *syn*-tetr-**1b** conformer might occur (path b), leading to the formation of the corresponding lithium enolate intermediate.^29^

In conclusion, we have developed an efficient one-pot, telescoped procedure for the stereoselective alkenylation of simple, non-activated amides using LiCH_2_SiMe_3_ and carbonyl compounds as surrogates of β-alkenyllithium reagents. Our strategy relies on the preliminary formation of stable tetrahedral intermediates, which, upon collapse in a solvent-dependent fashion, efficiently release highly reactive lithium enolates for *in situ* Claisen–Schmidt-type condensations. Our methodology allows for the assembly of substituted chalcones in good yields in a single synthetic operation with high stereoselectivity. Furthermore, bench-type aerobic conditions could also be employed using highly hydrophobic CPME as sustainable reaction media. The development of other electrophilic quenching strategies for the chemo- and stereoselective one-pot functionalization of lithium enolates, and complete DFT calculations aimed at clarifying the whole reaction mechanism and evaluating the energy barriers involved are under investigation and will be reported in due course.

## Data Availability

The data underlying this study are available in the published article and its Supporting Information.

## Supporting Information Available

General procedures, experimental details, characterization data for new compounds, copies of NMR spectra, and DFT calculation details (PDF)
