# Supplementary material for: One-Pot, Telescoped Alkenylation of Amides via Stable Tetrahedral Intermediates as Lithium Enolate Precursors
Source: Org Lett. 2023 May 23;25(21):3904–9. doi: 10.1021/acs.orglett.3c01269 (PMC10243110; doi:10.1021/acs.orglett.3c01269)
Supplement: Supplementary file 1 — ol3c01269_si_001.pdf [file ol3c01269_si_001.pdf]

Supporting Information for

One-pot, Telescoped Alkenylation of Amides via Stable Tetrahedral Intermediates as Lithium Enolate Precursors

Simone Ghinato,‡ Carolina Meazzo,‡ Federica De Nardi, Andrea Maranzana, Marco Blangetti and Cristina Prandi*

Dipartimento di Chimica, Università di Torino, via P. Giuria 7, I-10125 Torino, Italy

marco.blangetti@unito.it

Table of Contents

Experimental details	S3
Preparation of substrates: *N*-acylpyrrolidines **1**	S4
^{1}H and ^{13}C NMR spectra of *N*-acylpyrrolidines **1i** and **1j**	S9
Alkenylation of **1a** under different reaction conditions	S11
^{1}H and ^{13}C NMR spectra of compound **2a**	S12
Synthesis and analysis of compounds **2b-2o**	S22
^{1}H and ^{13}C NMR spectra of compounds **2b-2o**	S27
Gram-scale synthesis of (*E*)-chalcone (**2b**)	S43
^{1}H and ^{13}C NMR spectra of chalcone **2b** from gram-scale procedure	S44
Synthesis and analysis of compounds **2p-2ac**	S45
^{1}H and ^{13}C NMR spectra of compounds **2p-2ac**	S50
Synthesis and analysis of compounds **2ad-2ai**	S66
^{1}H and ^{13}C NMR spectra of compounds **2ad-2ai**	S69

Substrate limitations S76

Electrophilic quench experiments S77

In situ ^{13}C NMR monitoring of the S_NAc reaction on ^{13}C-labelled **1b-^{13}C** S83

^{1}H and ^{13}C NMR spectra of *N*-benzoylpyrrolidine **1b-^{13}C** S84

^{13}C{^{1}H} NMR spectra of *tetr*-**1b**-^{13}C S85

Computational details S89

References S104

Experimental details

Materials and methods. All reactions involving air-sensitive reagents were performed under nitrogen in oven-dried glassware using syringe-septum cap technique. Unless specified, all reagents were purchased from common suppliers and used as received. Anhydrous tetrahydrofuran (THF), 2-methyltetrahydrofuran (2-MeTHF) and cyclopentyl methyl ether (CPME) were distilled under nitrogen over Na/benzophenone ketyl prior to use. Reactions were monitored by GC-MS analysis and/or by thin-layer chromatography (TLC) carried out on 0.25 mm silica gel coated aluminium plates (60 Merck F254) with UV light (254 nm) as visualizing agent. Chromatographic separations were carried out under pressure on silica gel (40–63 µm, 230–400 mesh) using flash-column techniques. The following organolithium reagent was furnished by Merck-Aldrich and was used with the following concentration: $LiCH_2SiMe_3$ 0.7 M in hexanes. The exact concentration of the organolithium solution was determined by titration with diphenylacetic acid in anhydrous THF prior to use[1] and/or by No-D ^1H NMR techniques.[2] Full characterisation data, including copies of ^1H and ^{13}C NMR spectra, have been reported for both the newly synthesized compounds and the known compounds.

Instrumentation. ^1H NMR (600 MHz), $^{13}C\{^1H\}$ (150 MHz), ^{19}F (564 MHz) NMR spectra were recorded on a Jeol ECZR600 spectrometer at room temperature using residual solvent peak as an internal reference. ^2H NMR (92.07 MHz) spectra were obtained in CH_2Cl_2 using residual CD_2Cl_2 as an internal standard. Chemical shifts (δ) are given in parts per million (ppm) and coupling constants (*J*) in Hertz (Hz). Multiplicities are reported as follows: s (singlet), d (doublet), t (triplet), quint (quintet), sext (sextet), sept (septet), m (multiplet), br (broad). Low-resolution MS spectra were recorded at an ionizing voltage of 70 eV on a HP 5989B mass selective detector connected to an HP 5890 GC with a methyl silicone capillary column (EI). The MS flow-injection analyses were run on a high resolving power hybrid mass spectrometer (HRMS) Orbitrap Fusion (Thermo Scientific, Rodano, Italy), equipped with an ESI ion source. The samples were analyzed in acetonitrile solution using a syringe pump at a flow rate of 5 µL/min. The tuning parameters adopted for the ESI source were: source voltage 4.0 kV. The heated capillary temperature was maintained at 275 °C. The mass accuracy of the recorded ions (vs. the calculated ones) was ± 2.5 mmu (milli-mass units). Analyses were run using both full MS (150-2000 *m/z* range) and MS/MS acquisition, at 500000 resolutions (200 *m/z*).

n-Heptane was used as the internal standard (ISTD) for quantitative ^1H NMR analysis of the crude reaction mixtures. The amount of product was determined by applying the following equation:

$$Equation\ (1):\qquad yield\ (\%) = \frac{x\ (product) \cdot n\ (ISTD)}{n(starting\ material)} \cdot f \cdot 100$$

where:

x is the value of integral/number of protons;

n is the amount of the starting material and of internal standard (ISTD) in mmol;

f the diluting factor used for the preparation of the sample.

Preparation of substrates: *N*-acylpyrrolidines **1**

Synthesis of *N*-acylpyrrolidines **1a-h**, **1k-o**, **1af-ag**

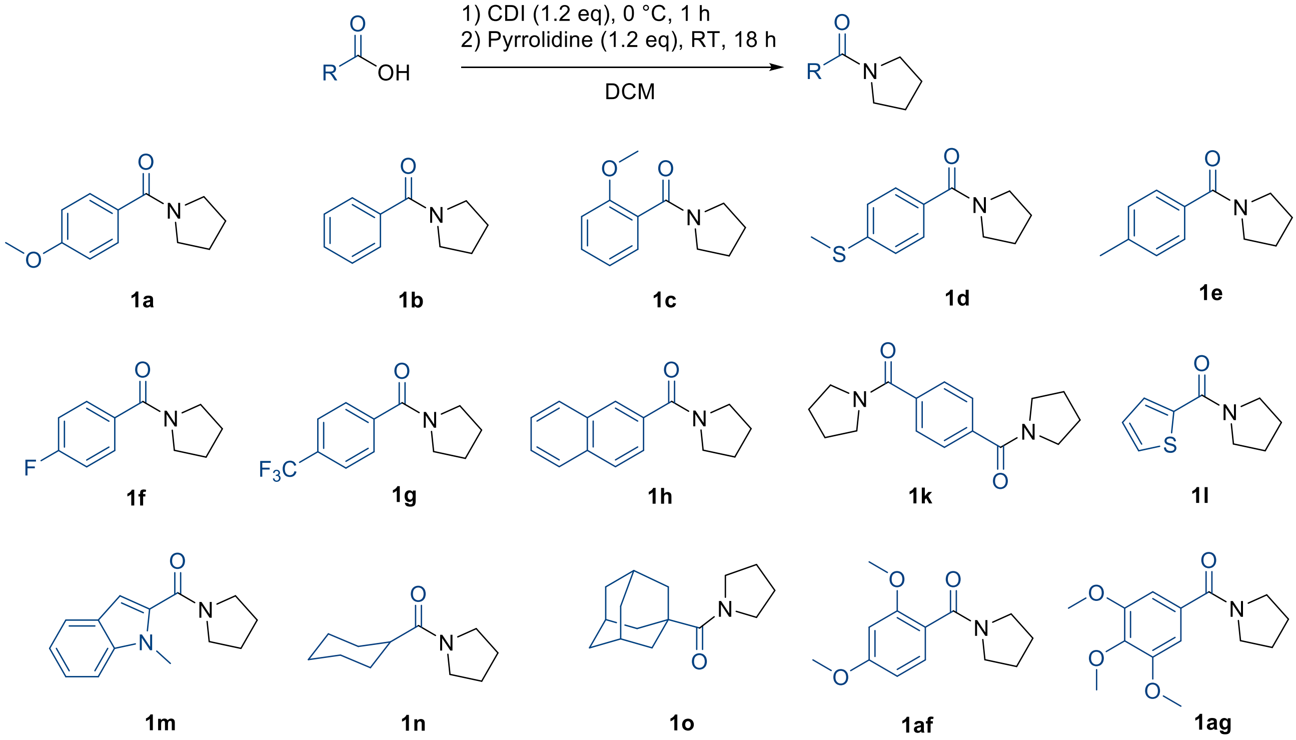

Scheme S1. Synthesis of *N*-acylpyrrolidines **1a-h**, **1k-o**, **1af**, **1ag**.

General procedure. To a stirred solution of the appropriate carboxylic acid (10 mmol, 1.0 eq.) in DCM (0.5 M, 20 mL), 1,1-carbonyldiimidazole (CDI, 12 mmol, 1.2 eq., 1.94 g) was added at 0 °C. The mixture was allowed to warm to room temperature and stirred for 1 h, then pyrrolidine (12 mmol, 1.2 eq., 0.99 mL) was added. The solution was stirred for 18 h at room temperature and quenched with 10 mL of aqueous 1 M HCl. After extraction with DCM, the combined organic layers were washed with aqueous 1 M HCl (3 x 10 mL) and aqueous 1 M $NaHCO_3$ (3 x 10 mL), dried over Na_2SO_4 and evaporated under reduced pressure. The crude products were used without further purification.

(4-Methoxyphenyl)(pyrrolidin-1-yl)methanone (1a): General procedure starting from 4-methoxybenzoic acid gave **1a** as a white solid (1.95 g, 95%). ^{1}H NMR (600 MHz, $CDCl_3$) δ 7.50-7.48 (m, 2H), 6.88-6.86 (m, 2H), 3.80 (s, 3H), 3.60 (t, J = 7.1 Hz, 2H), 3.45 (t, J = 6.7 Hz, 2H), 1.93 (quint, J = 6.8 Hz, 2H), 1.85 (quint, J = 6.7 Hz, 2H). ^{13}C{^{1}H} NMR (150 MHz, $CDCl_3$) δ 169.5, 160.9, 129.5, 129.3, 113.5, 55.4, 49.8, 46.5, 26.5, 24.7. All analytical data were in good accordance with data reported in the literature.[3]

Phenyl(pyrrolidine-1-yl)methanone (1b): General procedure starting from benzoic acid gave **1b** as a colorless oil (1.63 g, 93%). ^{1}H NMR (600 MHz, $CDCl_3$) δ 7.51-7.50 (m, 2H), 7.40-7.38 (m, 3H), 3.64 (t, J = 7.1 Hz, 2H), 3.42 (t, J = 6.7 Hz, 2H), 1.95 (quint, J = 7.3 Hz, 2H), 1.87 (quint, J = 6.8 Hz, 2H). ^{13}C{^{1}H} NMR

(150 MHz, $CDCl_3$) δ 169.7, 137.3, 129.8, 128.3, 127.1, 49.6, 46.2, 26.4, 24.5. All analytical data were in good accordance with data reported in the literature.[4]

(2-Methoxyphenyl)(pyrrolidin-1-yl)methanone (1c): General procedure starting from 2-methoxybenzoic acid gave **1c** as a colorless liquid (1.94 g, 95%). ^{1}H NMR (600 MHz, $CDCl_3$) δ 7.34-7.33 (m, 1H), 7.26-7.25 (m, 1H), 6.97 (tt, J = 7.4, 0.8 Hz, 1H), 6.91 (d, J = 8.4 Hz, 1H), 3.83 (s, 3H), 3.64 (t, J = 7.1 Hz, 2H), 3.21 (t, J = 6.8 Hz, 2H), 1.94 (quint, J = 6.8 Hz, 2H), 1.85 (quint, J = 6.8 Hz, 2H). ^{13}C{^{1}H} NMR (150 MHz, $CDCl_3$) δ 167.9, 155.4, 130.4, 127.9, 127.6, 120.9, 111.2, 55.7, 47.8, 45.6, 26.0, 24.7. All analytical data were in good accordance with data reported in the literature.[4]

(4-(Methylthio)phenyl)(pyrrolidine-1-yl)methanone (1d): General procedure starting from 4-(methylthio)benzoic acid gave **1d** as a white solid (1.98 g, 90%). ^{1}H NMR (600 MHz, $CDCl_3$) δ 7.47-7.45 (m, 2H), 7.25-7.23 (m, 2H), 3.63 (t, J = 7.0 Hz, 2H), 3.44 (t, J = 6.6 Hz, 2H), 2.49 (s, 3H), 1.95 (quint, J = 6.9 Hz, 2H), 1.89-1.86 (quint, J = 6.7 Hz, 2H). ^{13}C{^{1}H} NMR (150 MHz, $CDCl_3$) δ 169.3, 141.2, 133.5, 127.9, 125.6, 49.6, 46.3, 26.2, 24.9, 15.4. All analytical data were in good accordance with data reported in the literature.[5]

Pyrrolidin-1-yl(*p*-tolyl)methanone (1e): General procedure starting from 4-methylbenzoic acid gave **1e** as a white solid (1.76 g, 93%). ^{1}H NMR (600 MHz, $CDCl_3$) δ 7.43-7.41 (m, 2H), 7.21-7.18 (m, 2H), 3.54 (br s, 4H), 2.37 (s, 3H), 1.91 (br s, 4H). ^{13}C{^{1}H} NMR (150 MHz, $CDCl_3$) δ 169.9, 140.0, 134.4, 128.9, 127.3, 49.7, 46.3, 26.4, 24.6, 21.5. All analytical data were in good accordance with data reported in the literature.[4]

(4-Fluorophenyl)(pyrrolidin-1-yl)methanone (1f): General procedure starting from 4-fluorobenzoic acid gave **1f** as a white solid (1.68 g, 87%). ^{1}H NMR (600 MHz, $CDCl_3$) δ 7.55-7.52 (m, 2H), 7.10-7.06 (m, 2H), 3.64 (t, J = 7.0 Hz, 2H), 3.43 (t, J = 6.7 Hz, 2H), 1.96 (quint, J = 6.7 Hz, 2H), 1.89 (quint, J = 6.6 Hz, 2H). ^{13}C{^{1}H} NMR (150 MHz, $CDCl_3$) δ 168.8, 163.6 (d, J = 249.5 Hz, 1C), 133.4 (d, J = 3.5 Hz, 1C), 129.6 (d, J = 8.3 Hz, 1C), 115.4 (d, J = 21.7 Hz, 1C), 49.7, 46.7, 26.5, 24.5. All analytical data were in good accordance with data reported in the literature.[4]

Pyrrolidin-1-yl(4-(trifluoromethyl)phenyl)methanone (1g): General procedure starting from 4-(trifluoromethyl)benzoic acid gave **1g** as a white solid (2.21 g, 91%). ^{1}H NMR (600 MHz, $CDCl_3$) δ 7.68-7.66 (m, 2H), 7.63-7.61 (m, 2H), 3.66 (t, J = 7.1 Hz, 2H), 3.39 (t, J = 6.7 Hz, 2H), 1.98 (quint, J = 6.7 Hz, 2H), 1.90 (quint, J = 6.8 Hz, 2H). ^{13}C{^{1}H} NMR (150 MHz, $CDCl_3$) 168.4, 140.8, 131.8 (q, J = 32.6 Hz, 1C), 127.6, 125.5 (q, J = 3.2 Hz, 1C), 123.9 (q, J = 272.3 Hz, 1C), 49.6, 46.4, 26.5, 24.5. All analytical data were in good accordance with data reported in the literature.[4]

Naphthalen-2-yl(pyrrolidin-1-yl)methanone (1h): General procedure starting from 2-naphthoic acid gave **1h** as a white solid (1.97 g, 88%). ^{1}H NMR (600 MHz, $CDCl_3$) δ 8.01 (s, 1H), 7.88-7.84 (m, 3H), 7.62 (dd, J = 8.5, 1.7 Hz, 1H), 7.54-7.50 (m, 2H), 3.70 (t, J = 7.1 Hz, 2H), 3.49 (t, J = 6.7 Hz, 2H), 1.99 (quint, J = 6.9 Hz, 2H), 1.88 (quint, J = 6.8 Hz, 2H).^{13}C{^{1}H} NMR (150 MHz, $CDCl_3$) δ 169.8, 134.6, 133.9, 132.7, 128.6, 128.2, 127.9, 127.1, 127.0, 126.7, 124.5, 49.8, 46.4, 26.5, 24.6. All analytical data were in good accordance with data reported in the literature.[6]

1,4-Phenylenebis(pyrrolidin-1-ylmethanone) (1k) General procedure starting from benzoic acid, 2.2 eq. of CDI and 2.2 eq. of pyrrolidine gave **1k** as white solid (2.22 g, 82%). ^{1}H NMR (600 MHz, $CDCl_3$) δ 7.53 (s, 4H), 3.64 (t, *J* = 7.0 Hz, 4H), 3.39 (t, *J* = 6.7 Hz, 4H), 1.96 (quint, *J* = 6.8 Hz, 4H), 1.88 (quint, *J* = 6.7 Hz, 4H). ^{13}C{^{1}H} NMR (150 MHz, $CDCl_3$) δ 169.0, 138.6, 127.1, 49.6, 46.2, 26.4, 24.5. All analytical data were in good accordance with data reported in the literature.[7]

Pyrrolidin-1-yl(thiophen-2-yl)methanone (1l): General procedure starting from thiophene-2-carboxylic acid gave **1l** as a white solid (1.63 g, 90%). ^{1}H NMR (600 MHz, $CDCl_3$) δ 7.52 (dd, *J* = 3.7, 1.2 Hz, 1H), 7.47 (dd, *J* = 5.0, 1.2 Hz, 1H), 7.07 (dd, *J* = 5.0, 3.7 Hz, 1H), 3.77 (br s, 2H) superimposed to 3.68 (br s, 2H), 2.00 (br s, 2H) superimposed to 1.95 (br s, 2H). ^{13}C{^{1}H} NMR (150 MHz, $CDCl_3$) δ 161.9, 139.7, 129.7, 129.6, 127.2, 48.3 (br), 26.7, 24.6. All analytical data were in good accordance with data reported in the literature.[4]

(1-Methyl-*1H*-indol-2-yl)(pyrrolidin-1-yl)methanone (1m): General procedure starting from 1-methyl-1*H*-indole-2-carboxylic acid gave **1m** as a white solid (1.94 g, 85%). ^{1}H NMR (600 MHz, $CDCl_3$) δ 7.63 (dt, *J* = 7.9, 1.0 Hz, 1H), 7.37 (dd, *J* = 8.3, 1.0 Hz, 1H), 7.30 (ddd, *J* = 8.3, 6.9, 1.2 Hz, 1H), 7.14 (ddd, *J* = 8.0, 7.4, 1.1 Hz, 1H), 6.75 (d, *J* = 0.8 Hz, 1H), 3.94 (s, 3H), 3.71-3.69 (m, 4H), 1.98-1.96 (m, 4H). ^{13}C{^{1}H} NMR (150 MHz, $CDCl_3$) δ 162.5, 138.0, 132.8, 126.4, 123.6, 121.7, 120.2, 110.0, 104.7, 48.1 (br), 31.6, 25.6 (br). All analytical data were in good accordance with data reported in the literature.[4]

Cyclohexyl(pyrrolidin-1-yl)methanone (1n): General procedure starting from cyclohexanecarboxylic acid gave **1n** as a white solid (1.65 g, 91%). ^{1}H NMR (600 MHz, $CDCl_3$) δ 3.45 (t, *J* = 6.8 Hz, 4H), 2.33 (tt, *J* = 11.6, 3.5 Hz, 1H), 1.93-1.64 (m, 9H), 1.56-1.49 (m, 2H), 1.27-1.23 (m, 3H). ^{13}C{^{1}H} NMR (150 MHz, $CDCl_3$) δ 174.9, 46.3, 45.8, 43.0, 29.0, 26.3, 26.0, 25.9, 24.4. All analytical data were in good accordance with data reported in the literature.[4]

Adamantan-1-yl(pyrrolidin-1-yl)methanone (1o): General procedure starting from adamantane-1-carboxylic acid gave **1o** as a white solid (1.94 g, 83%). ^{1}H NMR (600 MHz, $CDCl_3$) δ 3.58 (br s, 4H), 2.03-2.02 (m, 3H), 2.00-1.97 (m, 6H), 1.93-1.82 (m, 3H), 1.74-1.68 (m, 7H). ^{13}C{^{1}H} NMR (150 MHz, $CDCl_3$) δ 176.0, 48.1, 41.8, 38.3, 36.8, 28.5. All analytical data were in good accordance with data reported in the literature.[4]

(2,4-Dimethoxyphenyl)(pyrrolidine-1-yl)methanone (1af): General procedure starting from 2,4-dimethoxybenzoic acid gave **1af** as a yellow liquid (2.33 g, 98%). ^{1}H NMR (600 MHz, $CDCl_3$) δ 7.21 (d, *J* = 8.3 Hz, 1H), 6.49 (dd, *J* = 8.3, 2.3 Hz, 1H), 6.45 (d, *J* = 2.2 Hz, 1H), 3.81 (s, 3H) superimposed to 3.81 (s, 3H), 3.62 (t, *J* = 7.1 Hz, 2H), 3.23 (t, *J* = 6.8 Hz, 2H), 1.93 (quint, *J* = 6.9 Hz, 2H), 1.84 (quint, *J* = 6.8 Hz, 2H). ^{13}C{^{1}H} NMR (150 MHz, $CDCl_3$) δ 167.8, 161.6, 156.8, 129.0, 120.5, 104.7, 98.8, 55.7, 55.5, 47.8, 45.7, 26.0, 24.7. All analytical data were in good accordance with data reported in the literature.[8]

(3,4,5-Trimethoxyphenyl)(pyrrolidine-1-yl)methanone (1ag): General procedure starting from 3,4,5-trimethoxybenzoic acid gave **1ag** as a colorless liquid (2.24 g, 91%). ^{1}H NMR (600 MHz, $CDCl_3$) δ 6.74 (s, 2H), 3.86 (s, 6H), 3.84 (s, 3H), 3.62 (t, *J* = 6.7 Hz, 2H), 3.45 (t, *J* = 6.8 Hz, 2H), 1.95 (quint, *J* = 7.0 Hz, 2H),

1.88 (quint, J = 6.4 Hz, 2H). $^{13}C\{^1H\}$ NMR (150 MHz, $CDCl_3$) δ 169.5, 153.1, 139.3, 132.6, 104.6, 104.6, 60.9, 56.3, 49.8, 46.4, 26.3, 24.6. All analytical data were in good accordance with data reported in the literature.[9]

Synthesis of *N*-acylpyrrolidines **1i-j**

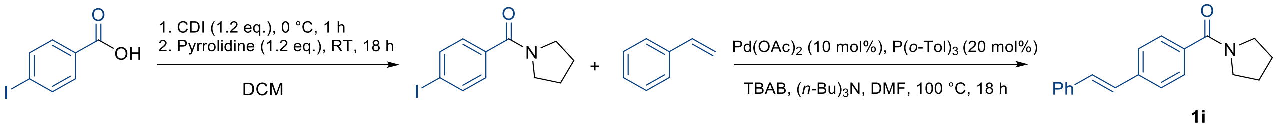

Scheme S2. Synthesis of (*E*)-pyrrolidin-1-yl(4-styrylphenyl)methanone **1i**.

(4-Iodophenyl)(pyrrolidin-1-yl)methanone. To a stirred solution of 4-iodobenzoic acid (10 mmol, 1.0 eq., 2.48 g) in DCM (0.5 M, 20 mL), 1,1-carbonyldiimidazole (CDI, 12 mmol, 1.2 eq., 1.94 g) was added at 0 °C. The mixture was allowed to warm to room temperature and stirred for 1 h, then pyrrolidine (12 mmol, 1.2 eq., 0.99 mL) was added. The solution was stirred for 18 h at room temperature and quenched with 10 mL of aqueous 1 M HCl. After extraction with DCM, the combined organic layers were washed with aqueous 1 M HCl (3 x 10 mL) and aqueous 1 M $NaHCO_3$ (3 x 10 mL), dried over Na_2SO_4 and evaporated under reduced pressure. The crude product was purified by flash column chromatography on silica gel (petroleum ether/EtOAc 6/4 v/v) to give (4-iodophenyl)(pyrrolidin-1-yl)methanone as a white solid (2.66 g, 88%, R_f = 0.25 petroleum ether/EtOAc 6/4 v/v). ^1H NMR (600 MHz, $CDCl_3$) 7.76-7.73 (m, 2H), 7.27-7.24 (m, 2H), 3.63 (t, J = 6.9 Hz, 2H), 3.41 (t, J = 6.6 Hz, 2H), 1.95 (quint, J = 6.6 Hz, 2H), 1.88 (quint, J = 6.4 Hz, 2H).$^{13}C\{^1H\}$ NMR (150 MHz, $CDCl_3$) δ 168.8, 137.5, 136.6, 129.0, 96.2, 49.7, 46.4, 26.5, 24.5. All analytical data were in good accordance with data reported in the literature.[10]

(*E*)-Pyrrolidin-1-yl(4-styrylphenyl)methanone (1i). A solution of (4-iodophenyl)(pyrrolidin-1-yl)methanone (3.3 mmol, 1.0 eq., 1.0 g) in anhydrous DMF (0.33 M, 10 mL) was degassed with nitrogen for 30 min. Tetrabutylammonium bromide (TBAB) (1.32 mmol, 0.4 eq., 430 mg), $(n\text{-Bu})_3N$ (6.6 mmol, 2.0 eq., 1.57 mL), $Pd(OAc)_2$ (0.33 mmol, 0.10 eq., 75 mg), $P(o\text{-Tol})_3$ (0.66 mmol, 0.2 eq., 200 mg) and styrene (6.6 mmol, 2.0 eq., 687 mg) were sequentially added. The resulting reaction mixture was stirred for 18 h at 100 °C. The mixture was cooled to room temperature and then filtered over a celite pad. After extraction with EtOAc (3 x 15 mL), the combined organic layers were washed with brine (3 x 10 mL), dried over Na_2SO_4 and concentrated under reduced pressure. Purification by flash column chromatography on silica gel (petroleum ether/EtOAc 4/6 v/v) gave **1i** as a white solid (0.41 g, 45%, R_f= 0.3 PE/EtOAc 4/6 v/v), mp. 162.1-164.8 °C. ^1H NMR (600 MHz, $CDCl_3$) δ 7.54-7.51 (m, 6H), 7.39-7.35 (m, 2H), 7.29-7.27 (m, 1H), 7.16 (d, J = 16.4 Hz, 1H), 7.11 (d, J =16.4 Hz, 1H), 3.64 (br s, 2H) superimposed to 3.49 (br s, 2H), 1.93 (br s, 4H). $^{13}C\{^1H\}$ NMR (150 MHz, $CDCl_3$) δ 169.6, 139.0, 137.1, 136.2, 130.1, 128.9, 128.1, 128.0, 127.9, 126.8, 126.4, 49.8, 46.4, 26.6, 24.6. HRMS (ESI) m/z: $[M+H]^+$ Calcd for $C_{19}H_{20}NO$ 278.1539; Found 278.1524.

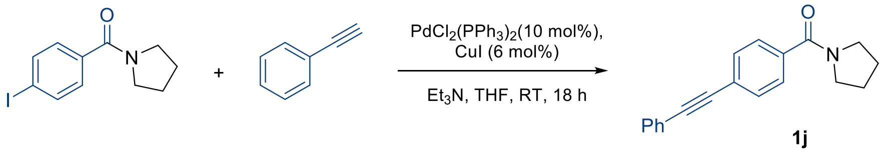

Scheme S3. Synthesis of (4-(phenylethynyl)phenyl)(pyrrolidin-1-yl)methanone **1j**

(4-(Phenylethynyl)phenyl)(pyrrolidin-1-yl)methanone (1j). A solution of (4-iodophenyl)(pyrrolidin-1-yl)methanone (3.3 mmol, 1.0 eq., 1.0 g) and Et_3N (4.29 mmol, 1.3 eq., 0.59 mL) in anhydrous THF (0.33 M, 10 mL) was degassed with nitrogen for 30 min. $PdCl_2(PPh_3)_2$ (0.08 mmol, 0.025 eq. 56 mg), CuI (0.198 mmol, 0.06 eq. 38 mg) and ethynylbenzene (4.29 mmol, 1.3 eq., 0.47 mL) were sequentially added and the resulting mixture was stirred for 18 h at room temperature. The reaction was quenched with a saturated aqueous NH_4Cl solution (20 mL) and then extracted with EtOAc (3 x 10 mL), dried over Na_2SO_4 and concentrated under reduced pressure. Purification by flash column chromatography on silica gel (petroleum ether/EtOAc 4/6 v/v) followed by recrystallization with *n*-hexane/EtOAc gave **1j** as white solid (0.40 g, 53%, R_f = 0.35 petroleum ether/EtOAc 4/6 v/v), mp. 129.6-131.3 °C. ^{1}H NMR (600 MHz, $CDCl_3$) δ 7.57-7.50 (m, 6H), 7.38-7.34 (m, 3H), 3.65 (t, J = 7.0 Hz, 2H), 3.44 (t, J = 6.7 Hz, 2H), 1.97 (quint, J = 7.0 Hz, 2H), 1.89 (quint, J = 6.8 Hz, 2H). ^{13}C{^{1}H} NMR (150 MHz, $CDCl_3$) δ 169.2, 136.9, 131.8, 131.6, 128.7, 128.6, 127.4, 125.0, 123.1, 91.0, 88.9, 49.7, 46.4, 26.6, 24.6. HRMS (ESI) *m*/*z*: $[M+H]^+$ Calcd for $C_{19}H_{18}NO$ 276.1383; Found 276.1391.

^{1}H and ^{13}C NMR spectra of *N*-acylpyrrolidines **1i** and **1j**

(*E*)-Pyrrolidin-1-yl(4-styrylphenyl)methanone (1i)

^{1}H NMR (600 MHz, $CDCl_3$)

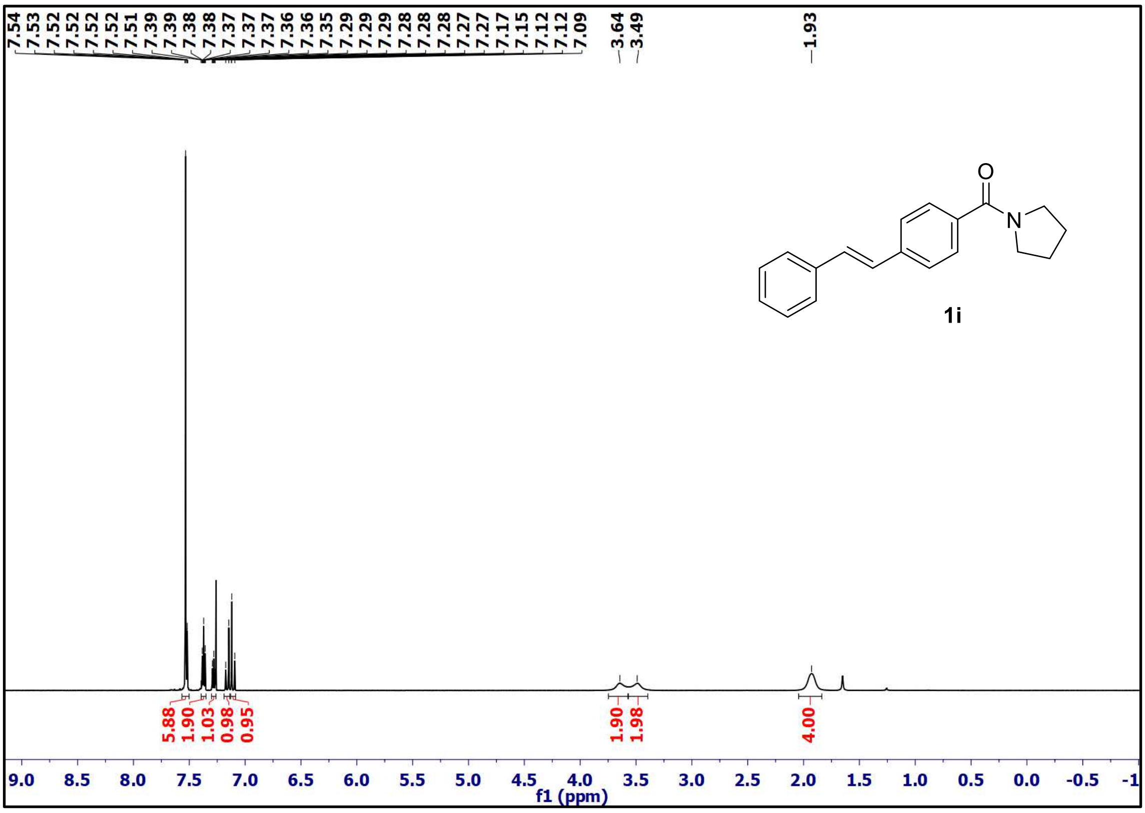

^{13}C NMR (150 MHz, $CDCl_3$)

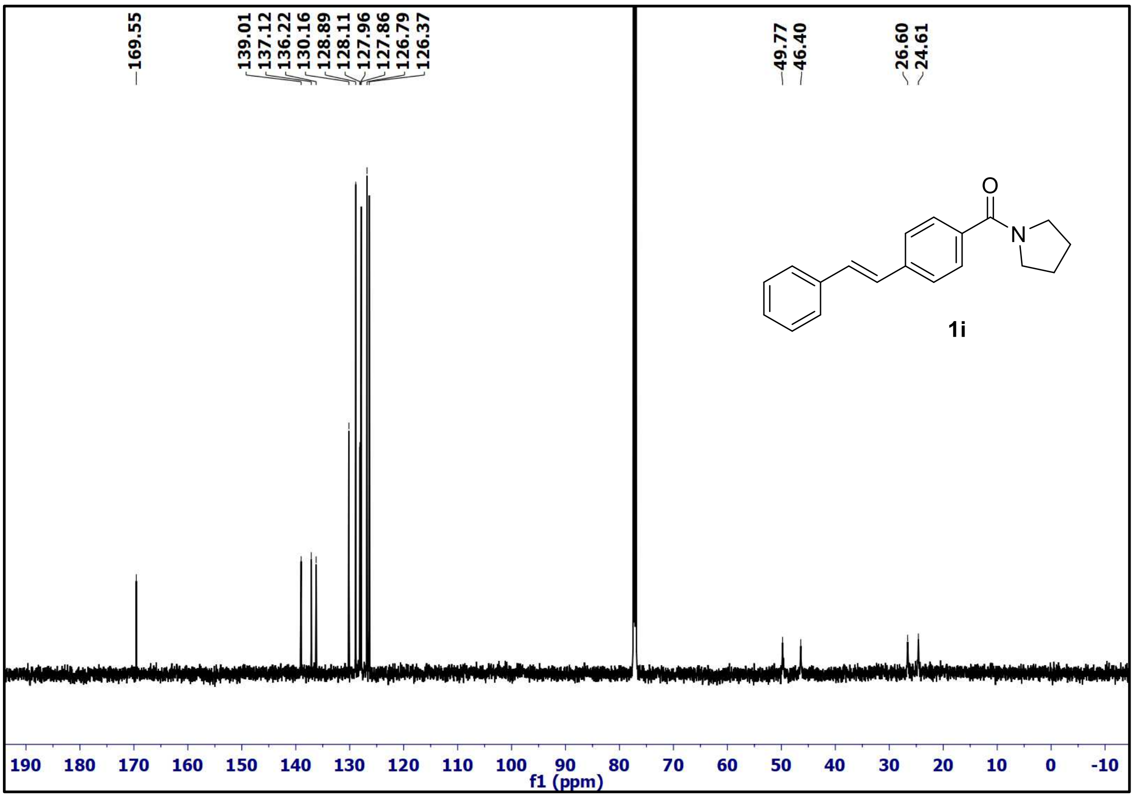

(4-(Phenylethynyl)phenyl)(pyrrolidin-1-yl)methanone (1j)

^{1}H NMR (600 MHz, $CDCl_3$)

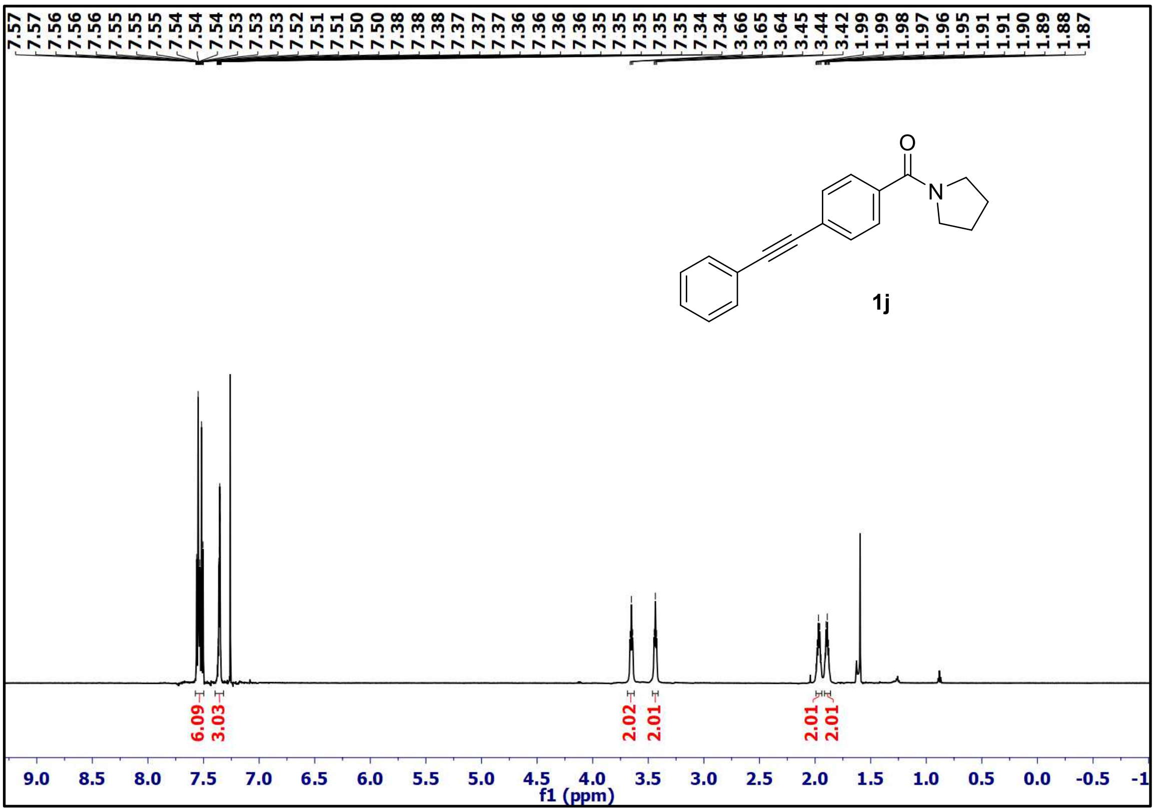

^{13}C NMR (150 MHz, $CDCl_3$)

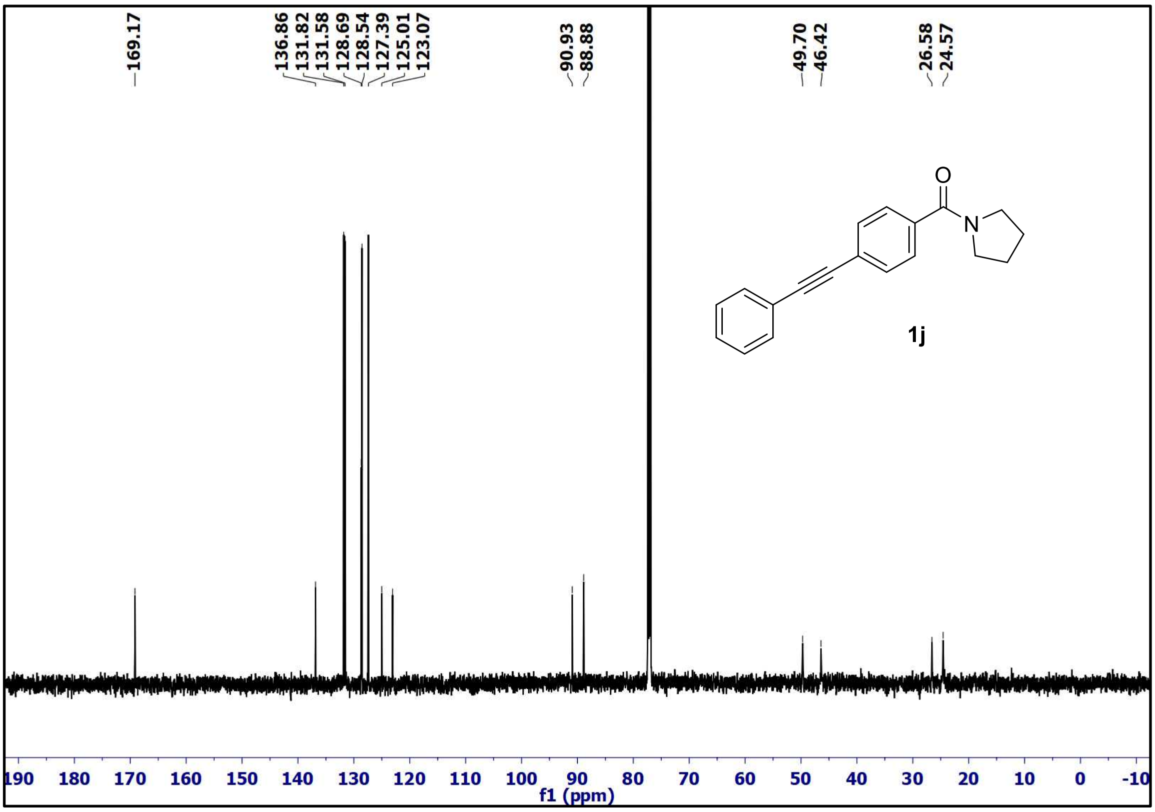

Alkenylation of **1a** under different reaction conditions

General procedure. In a flame-dried flask equipped with a magnetic stirrer, the appropriate amount of $LiCH_2SiMe_3$ (0.7 M in hexanes) was slowly added to a stirred solution of (4-methoxyphenyl)(pyrrolidin-1-yl)methanone **1a** (0.2 mmol, 1 eq., 41 mg) in the selected dry solvent (5 mL). The reaction mixture was stirred at room temperature until complete consumption of the starting material (monitored by TLC analysis, 30 min), then the appropriate amount of benzaldehyde was added. The mixture was stirred at the indicated temperature for the selected reaction time (Table S1), then quenched with H_2O (5 mL). After extraction with EtOAc (3 x 10 mL), the combined organic layers were dried over Na_2SO_4 and the solvent removed under reduced pressure. The yield of **2a** was determined by ^{1}H NMR analysis of the crude reaction mixture. A sample of **2a** was synthesized according to the procedure reported in the literature and used as reference for qNMR analysis.[6] Yields of **2a** were determined by quantitative ^{1}H NMR analysis of the crude reaction mixtures using *n*-heptane (0.2 mmol, 28 μL) as the internal standard and a diluting factor 1 for the preparation of the sample (see Eq. 1).

SAFETY NOTE: Organolithiums were handled under an inert atmosphere (Schlenk techniques). No particular problems were experienced during the addition under air. Organolithiums, however, are notoriously prone to ignition in air, and caution should be exercised in adopting the recommended procedure, especially on a larger scale.

(*E*)-1-(4-Methoxyphenyl)-3-phenylprop-2-en-1-one (2a): General procedure starting from **1a** (0.2 mmol, 1 eq., 41 mg), $LiCH_2SiMe_3$ (0.7 M in hexanes, 0.3 mmol, 1.5 eq., 0.43 mL) and benzaldehyde (0.24 mmol, 1.2 eq., 24 μL). Reaction time: 1 h, conditions reported in Table S1, entry 2. Purification by flash column chromatography on silica gel (petroleum ether/EtOAc 9/1 v/v) gave **2a** as a white solid (31 mg, 65%, R_f = 0.3 petroleum ether/EtOAc 9/1 v/v), mp 103.0-104.0 °C.[6] ^{1}H NMR (600 MHz, $CDCl_3$) δ 8.06-8.04 (m, 2H), 7.81 (d, *J* = 15.7 Hz, 1H), 7.66-7.64 (m, 2H), 7.55 (d, *J* = 15.6 Hz, 1H), 7.44.7.39 (m, 3H), 7.00-6.98 (m, 2H), 3.90 (s, 3H). $^{13}C\{^1H\}$ NMR (150 MHz, $CDCl_3$) δ 188.9, 163.6, 144.1, 135.2, 131.3, 131.0, 130.5, 129.1, 128.5, 122.0, 114.0, 55.7. EI-MS *m/z* (%): 238 (M^+, 100), 237 (95), 135 (89). HRMS (ESI) *m/z*: $[M+H]^+$ Calcd for $C_{16}H_{15}O_2$ 239.1067; Found 239.1066.

^{1}H and ^{13}C NMR spectra of compound **2a**

^{1}H NMR (600 MHz, $CDCl_3$)

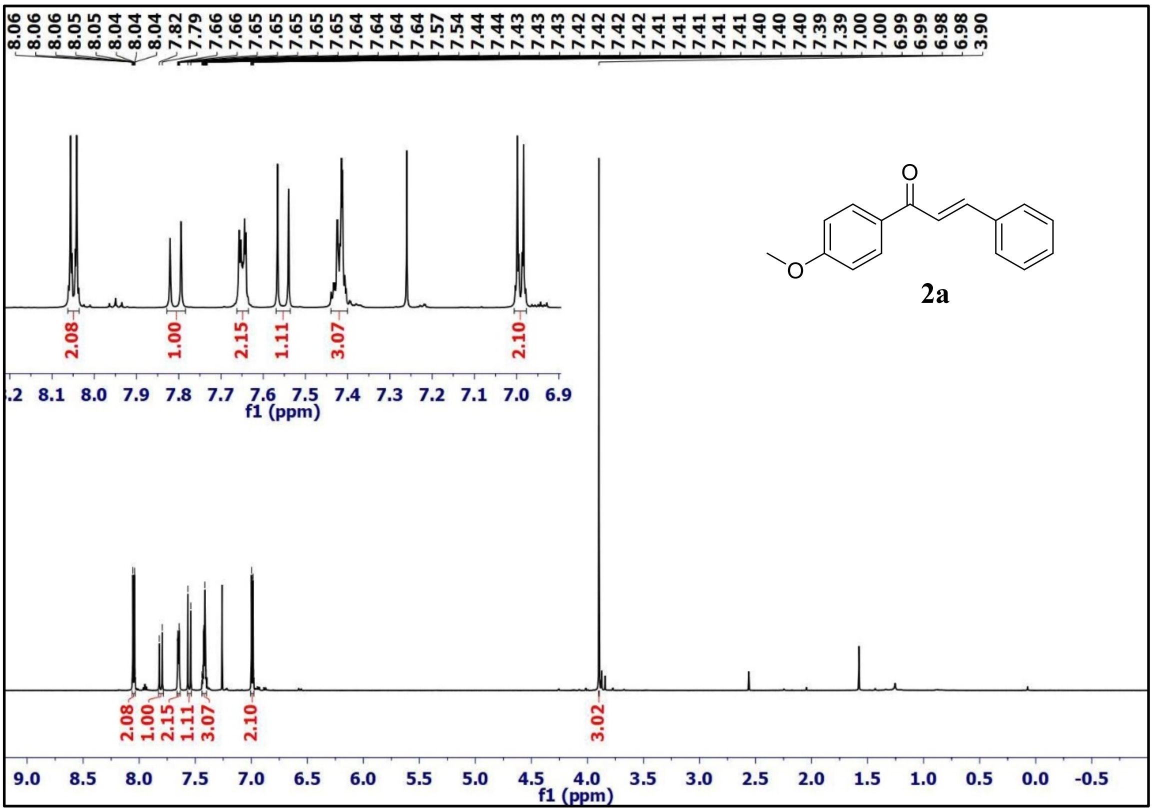

^{13}C NMR (150 MHz, $CDCl_3$)

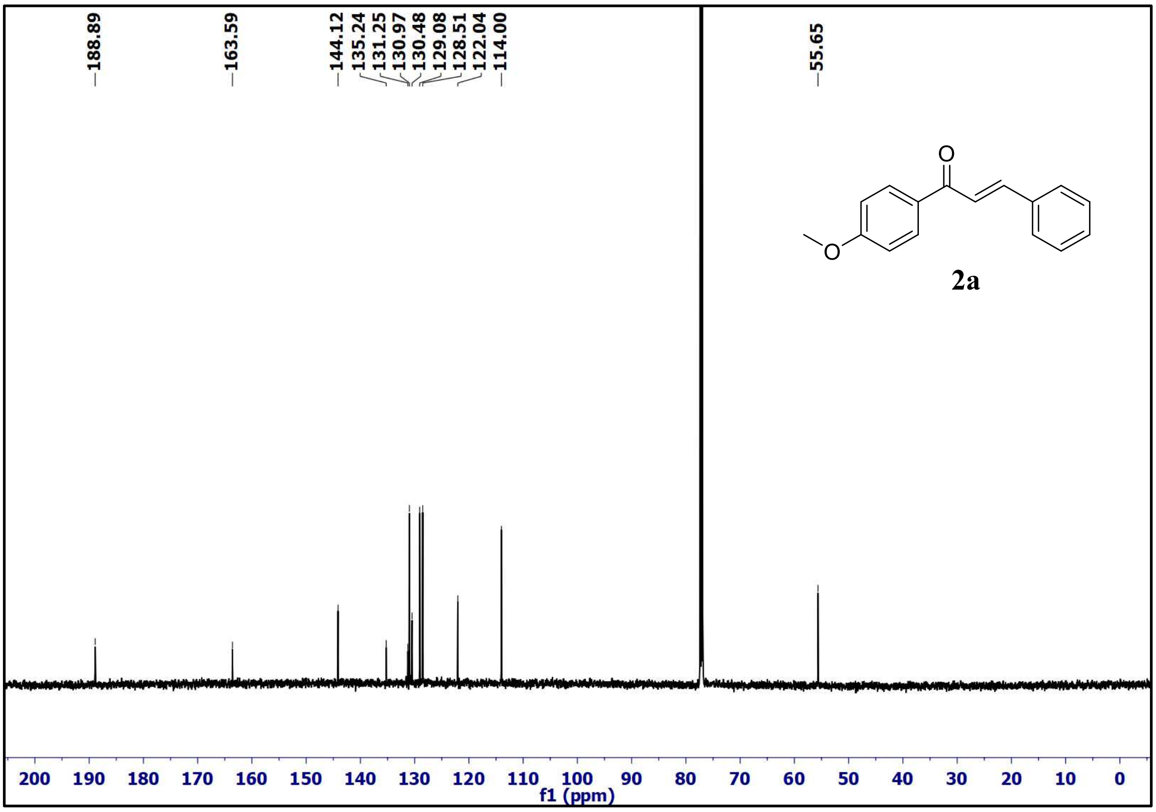

Table S1. Alkenylation of **1a** under different reaction conditions.[a]

Entry	Solvent	$LiCH_2SiMe_3$ (eq.)	PhCHO (eq.)	time (h)	**2a** (%)[b]
1	CPME	1.0	1.2	1	35
2	CPME	1.5	1.2	1	67
3	CPME	2.0	1.2	1	52
4	CPME	1.5	2.0	1	62
5	CPME	1.5	1.2	12	49
6	CPME	1.5	1.2	1	59[c]
7	THF	1.5	1.2	1	60
8	2-MeTHF	1.5	1.2	1	57
9	CPME	1.5	1.2	1	40[d]
10	THF	1.5	1.2	1	67[d]
11	2-MeTHF	1.5	1.2	1	48[d]
12	CPME	1.5	1.2	1	51[e]
13	THF	1.5	1.2	1	35[e]
14	2-MeTHF	1.5	1.2	1	39[e]
15	CPME	1.5	1.2	1	57[f]
16	CPME	1.5	1.2	1	53[g]

[a] Reaction conditions: **1a** (0.2 mmol), $LiCH_2SiMe_3$ (0.7 M in hexanes), solvent (5.0 mL), room temperature. [b] Determined by quantitative ^{1}H NMR using *n*-heptane as the internal standard. *E*/*Z* ratio > 99/1. [c] Reaction with benzaldehyde performed at 60 °C. [d] *N*,4-dimethoxy-*N*-methylbenzamide (Weinreb amide) was used as substrate. [e] Reaction performed under air. [f] Reaction performed in the presence of quinuclidine (0.30 mmol, 1.5 eq., 33 mg). [g] Reaction performed in the presence of LiCl (0.30 mmol, 1.5 eq., 13 mg).

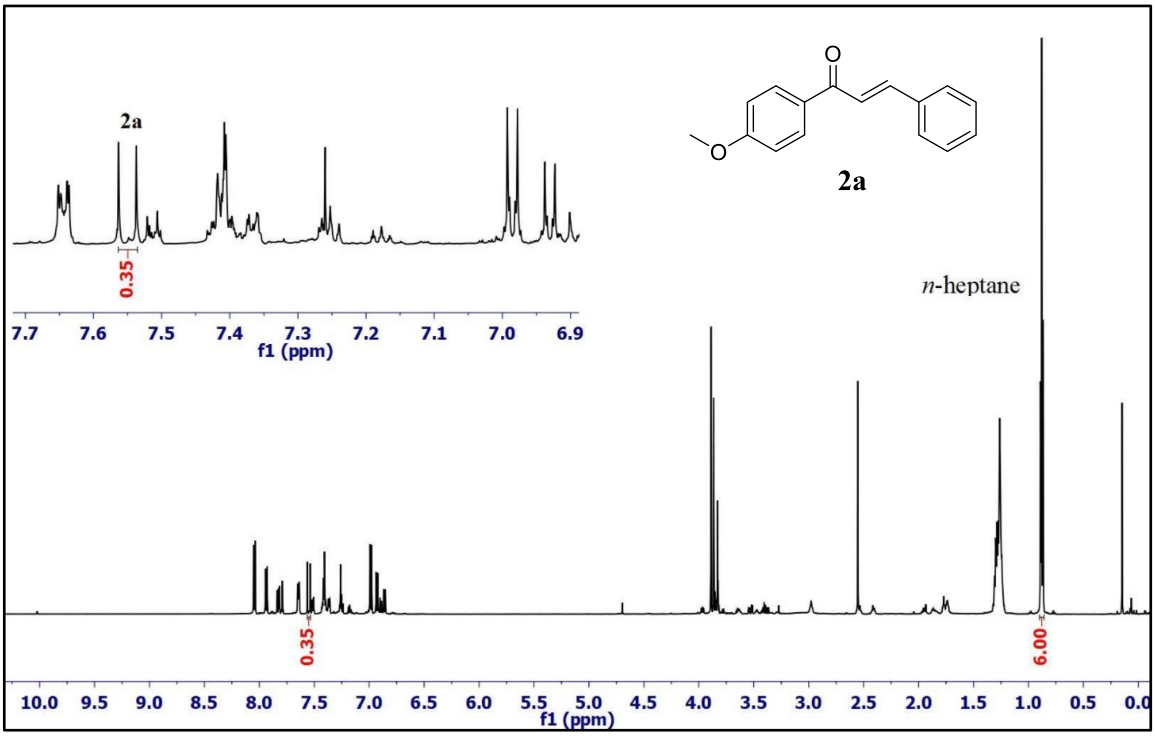

Figure S1: Table S1 entry 1: crude ^{1}H NMR spectrum of the reaction performed on **1a** (0.20 mmol, 1.0 eq.), CPME (5 mL), $LiCH_2SiMe_3$ (0.20 mmol, 1.0 eq.), PhCHO (0.24 mmol, 1.2 eq.), 1 h. Yield **2a** 35%.

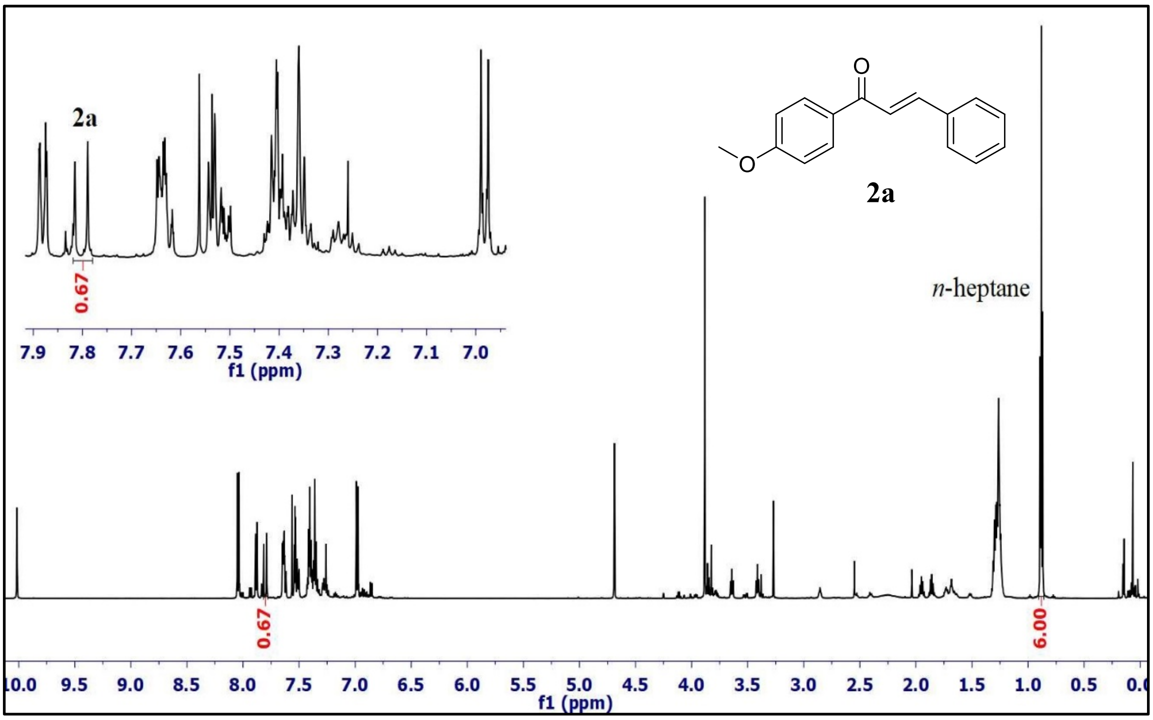

Figure S2: Table S1 entry 2: crude ^{1}H NMR spectrum of the reaction performed on **1a** (0.20 mmol, 1.0 eq.), CPME (5 mL), $LiCH_2SiMe_3$ (0.30 mmol, 1.5 eq.), PhCHO (0.24 mmol, 1.2 eq.), 1 h. Yield **2a** 67%.

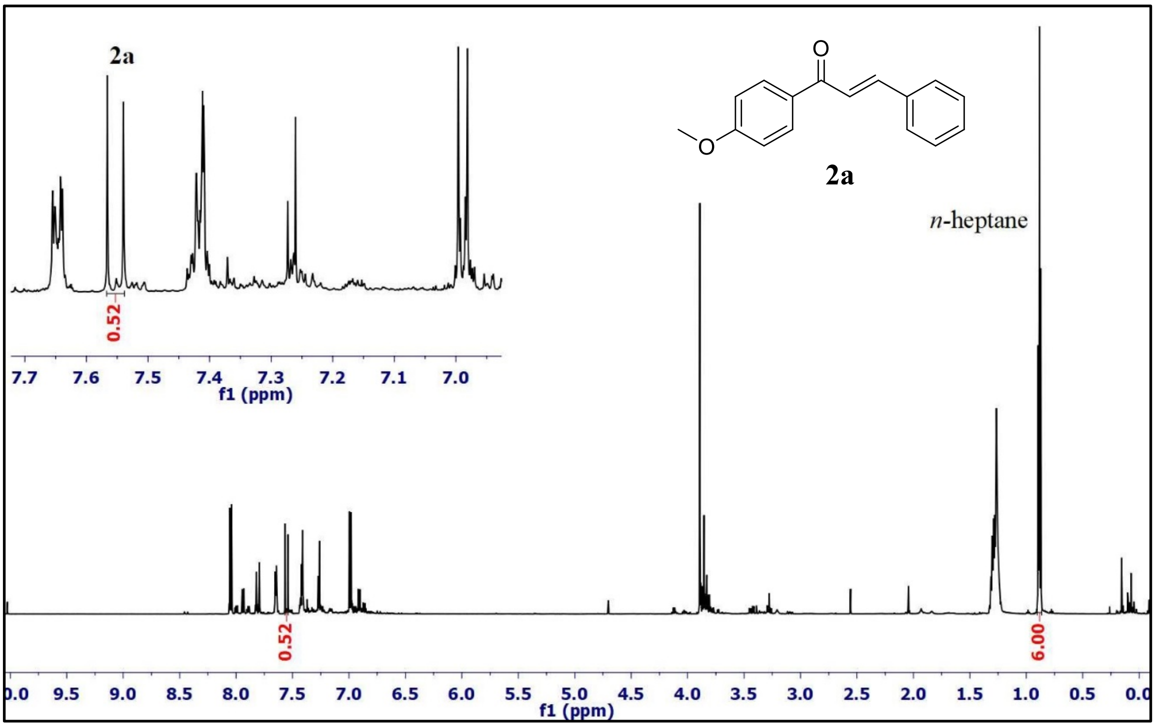

Figure S3: Table S1 entry 3: crude ^{1}H NMR spectrum of the reaction performed on **1a** (0.20 mmol, 1.0 eq.), CPME (5 mL), $LiCH_2SiMe_3$ (0.40 mmol, 2.0 eq.), PhCHO (0.24 mmol, 1.2 eq.), 1 hour. Yield **2a** 52%.

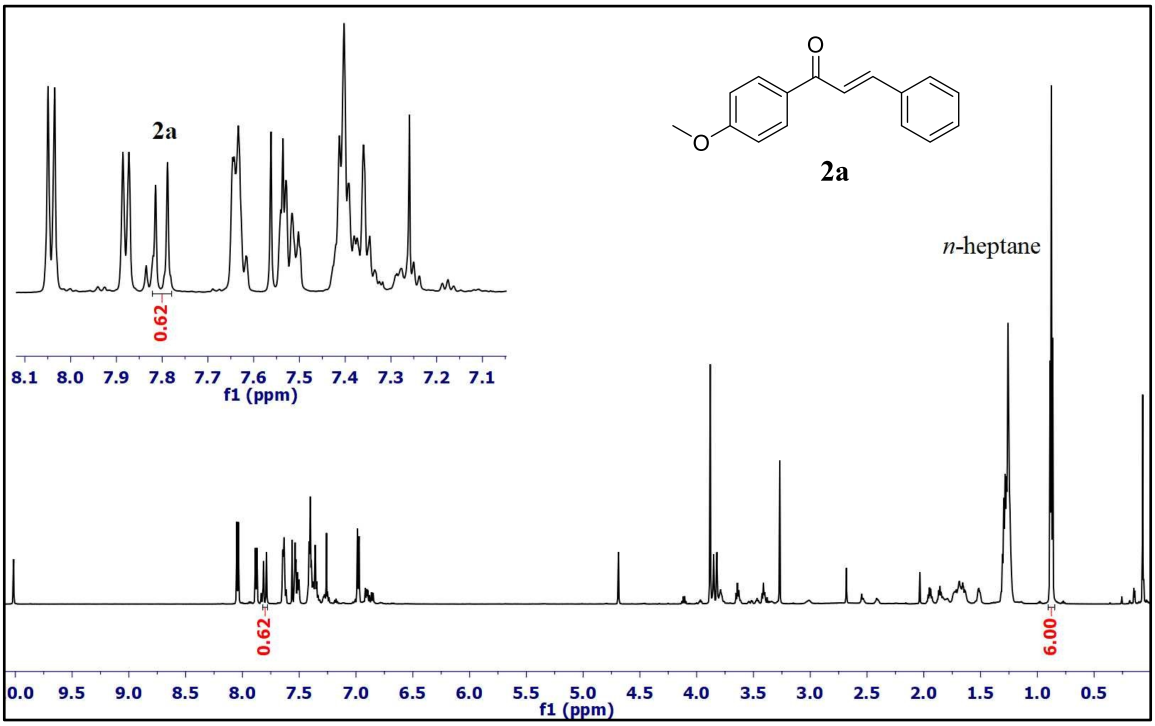

Figure S4: Table S1 entry 4: crude ^{1}H NMR spectrum of the reaction performed on **1a** (0.20 mmol, 1.0 eq.), CPME (5 mL), $LiCH_2SiMe_3$ (0.30 mmol, 1.5 eq.), PhCHO (0.40 mmol, 2.0 eq.), 1 h. Yield **2a** 62%.

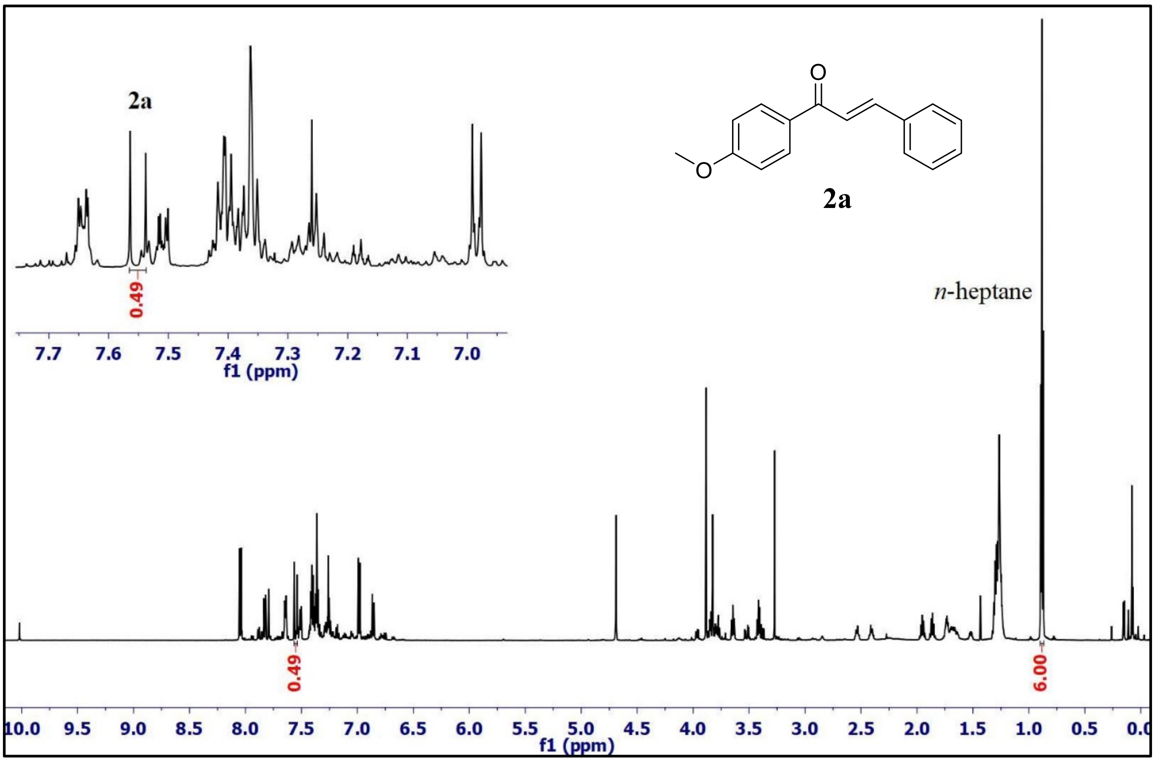

Figure S5: Table S1 entry 5: crude ^{1}H NMR spectrum of the reaction performed on **1a** (0.20 mmol, 1.0 eq.), CPME (5 mL), $LiCH_2SiMe_3$ (0.30 mmol, 1.5 eq.), PhCHO (0.24 mmol, 1.2 eq.), 12 h. Yield **2a** 49%.

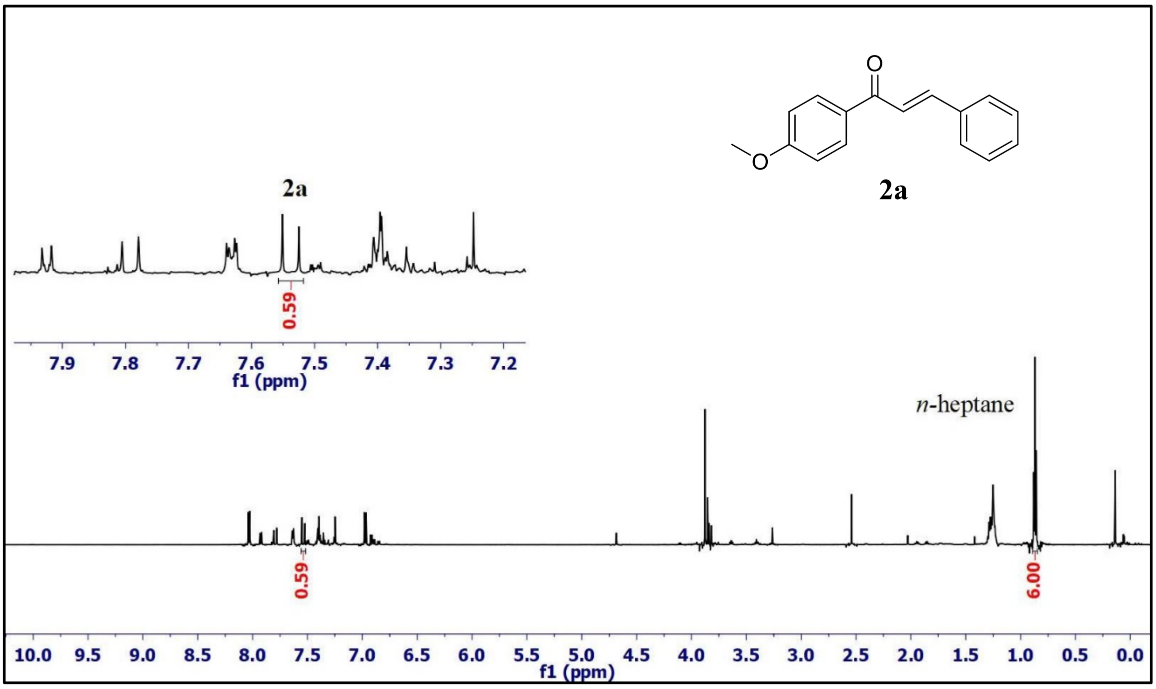

Figure S6: Table S1 entry 6: crude ^{1}H NMR spectrum of the reaction performed on **1a** (0.20 mmol, 1.0 eq.), CPME (5 mL), $LiCH_2SiMe_3$ (0.30 mmol, 1.5 eq.), PhCHO (0.24 mmol, 1.2 eq.), 1 h. Reaction with benzaldehyde performed at 60 °C. Yield **2a** 59%.

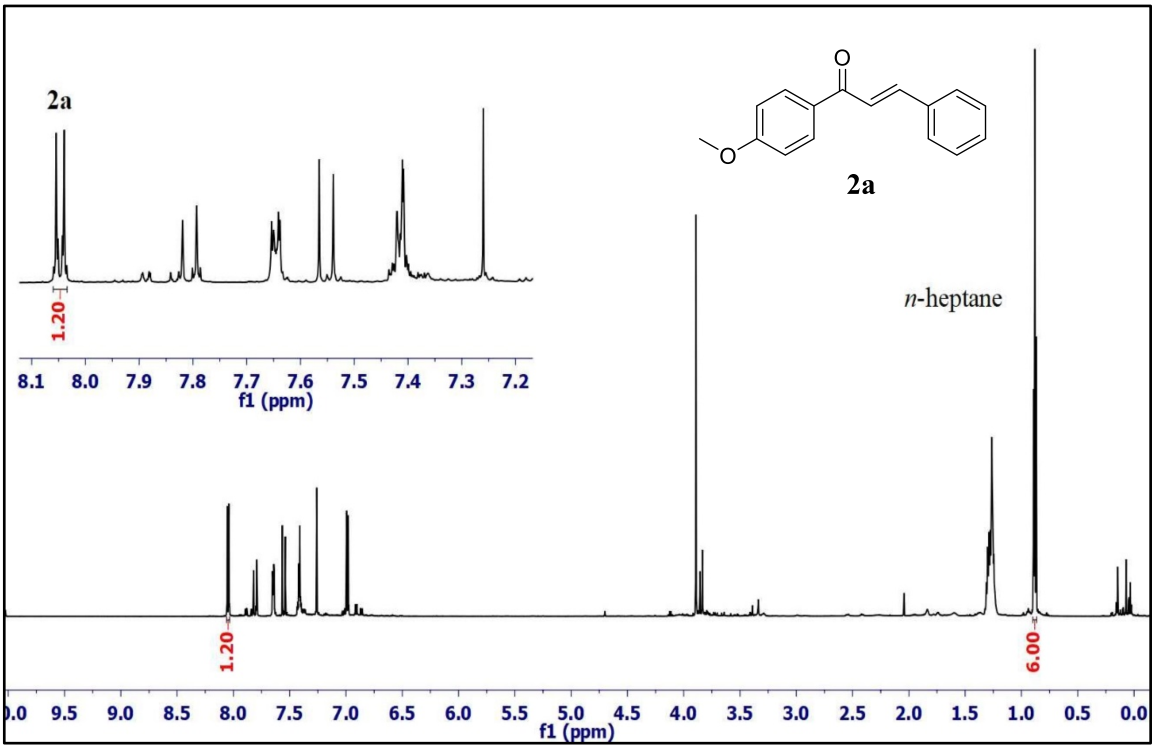

Figure S7: Table S1 entry 7: crude ^{1}H NMR spectrum of the reaction performed on **1a** (0.20 mmol, 1.0 eq.), THF (5 mL), $LiCH_2SiMe_3$ (0.30 mmol, 1.5 eq.), PhCHO (0.24 mmol, 1.2 eq.), 1 h. Yield **2a** 60%.

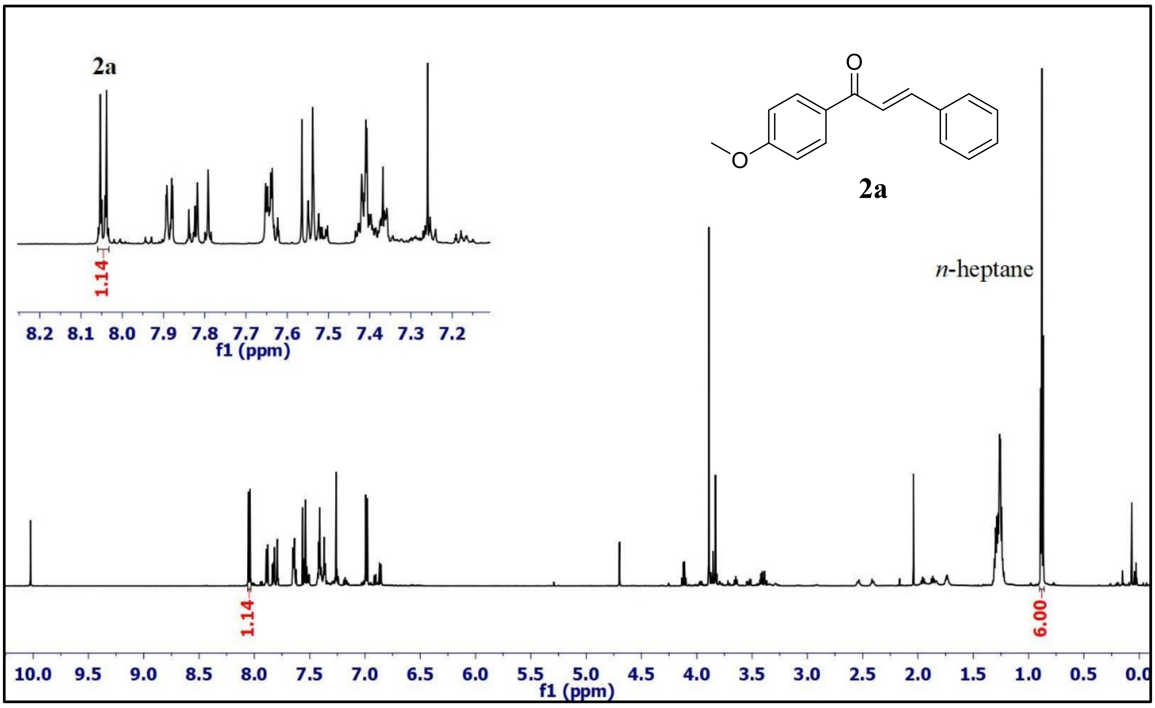

Figure S8: Table S1 entry 8: crude ^{1}H NMR spectrum of the reaction performed on **1a** (0.20 mmol, 1.0 eq.), 2-MeTHF (5 mL), $LiCH_2SiMe_3$ (0.30 mmol, 1.5 eq.), PhCHO (0.24 mmol, 1.2 eq.), 1 h. Yield **2a** 57%.

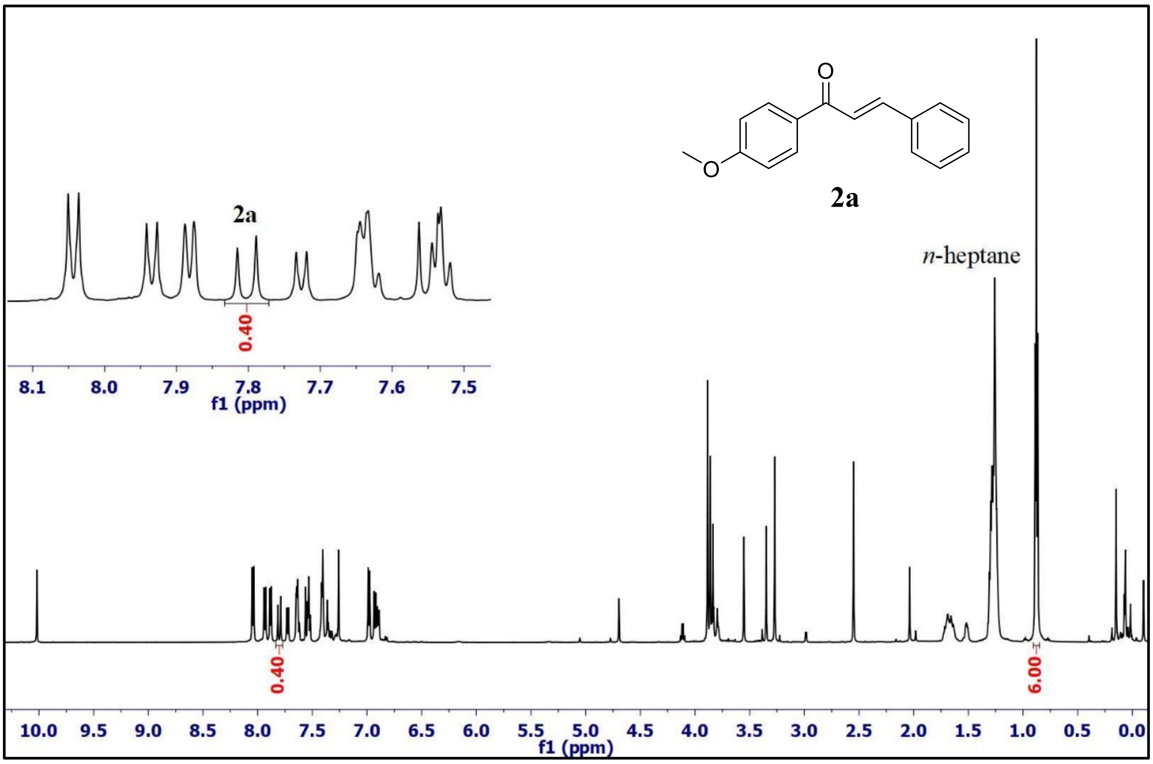

Figure S9: Table S1 entry 9: crude ^{1}H NMR spectrum of the reaction performed on *N*,4-dimethoxy-*N*-methylbenzamide (0.20 mmol, 1.0 eq.), CPME (5 mL), $LiCH_2SiMe_3$ (0.30 mmol, 1.5 eq.), PhCHO (0.24 mmol, 1.2 eq.), 1 h. Yield **2a** 26%.

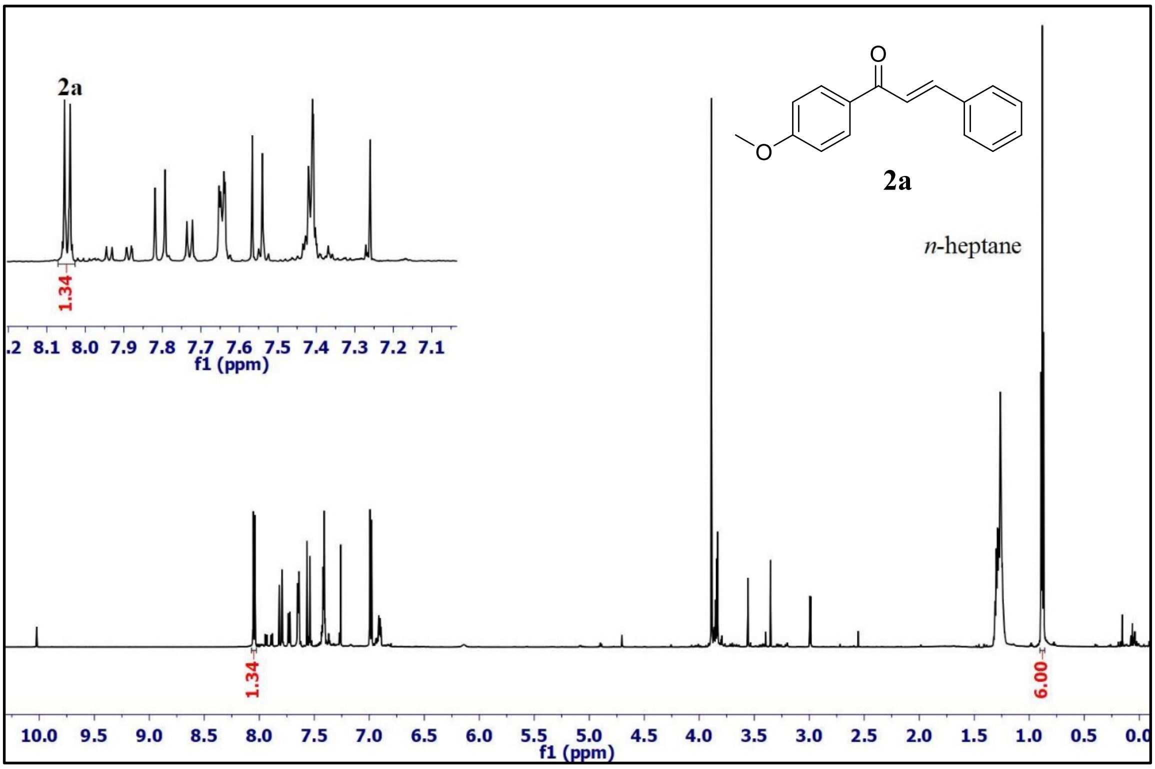

Figure S10: Table S1 entry 10: crude ^{1}H NMR spectrum of the reaction performed on *N*,4-dimethoxy-*N*-methylbenzamide (0.20 mmol, 1.0 eq.), THF (5 mL), $LiCH_2SiMe_3$ (0.30 mmol, 1.5 eq.), PhCHO (0.24 mmol, 1.2 eq.), 1 h. Yield **2a** 67%.

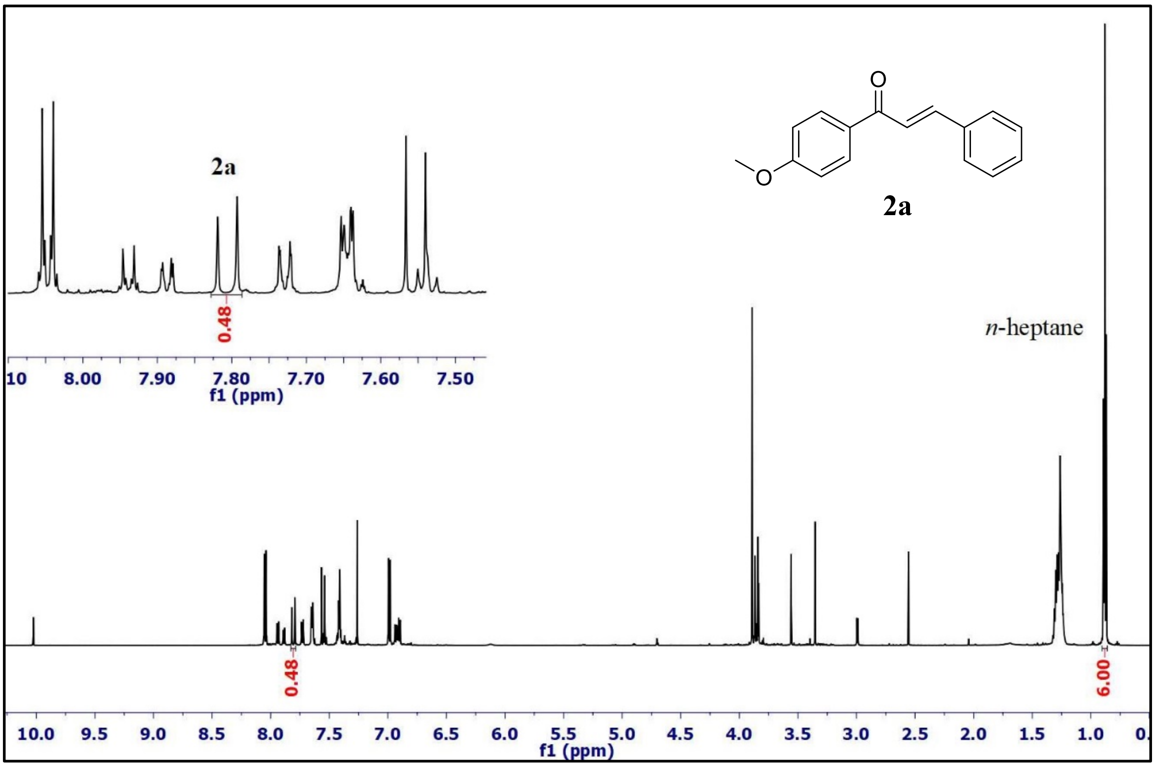

Figure S11: Table S1 entry 11: crude ^{1}H NMR spectrum of the reaction performed on *N*,4-dimethoxy-*N*-methylbenzamide (0.20 mmol, 1.0 eq.), 2-MeTHF (5 mL), $LiCH_2SiMe_3$ (0.30 mmol, 1.5 eq.), PhCHO (0.24 mmol, 1.2 eq.), 1 hour. Yield **2a** 48%.

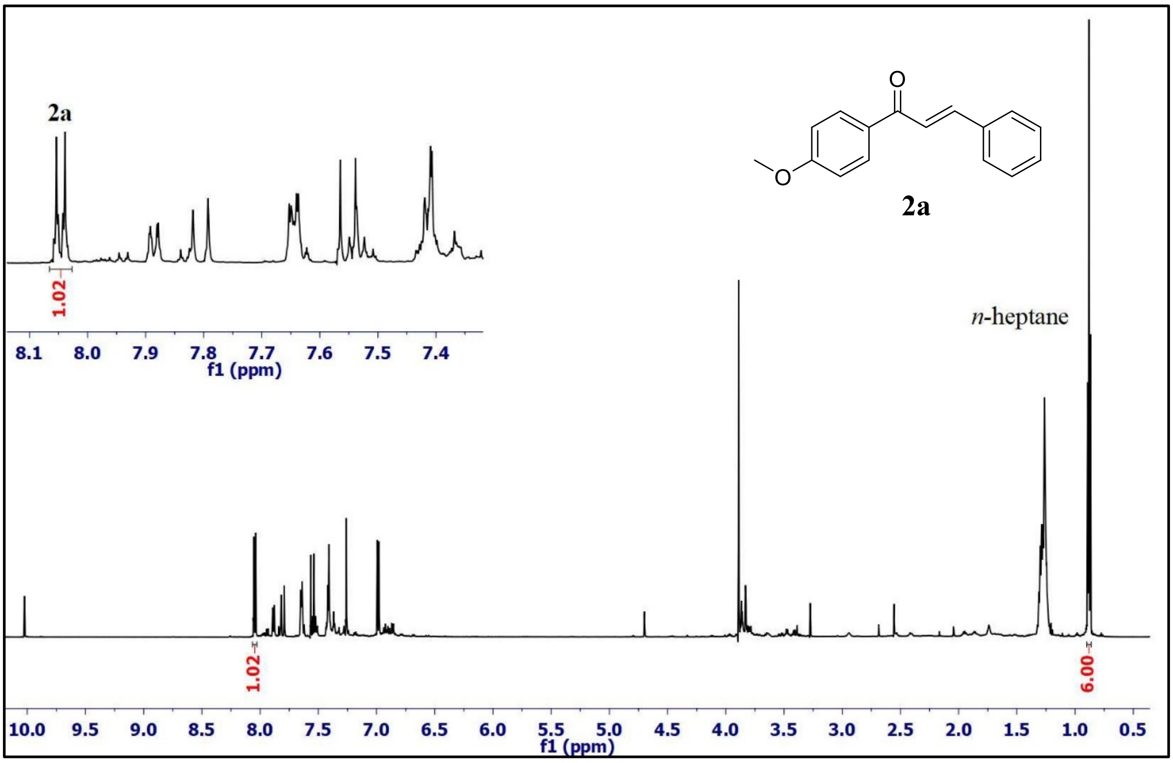

Figure S12: Table S1 entry 12: crude ^{1}H NMR spectrum of the reaction performed on **1a** (0.20 mmol, 1.0 eq.), CPME (5 mL), $LiCH_2SiMe_3$ (0.30 mmol, 1.5 eq.), PhCHO (0.24 mmol, 1.2 eq.), 1 h. Reaction performed under air. Yield **2a** 51%.

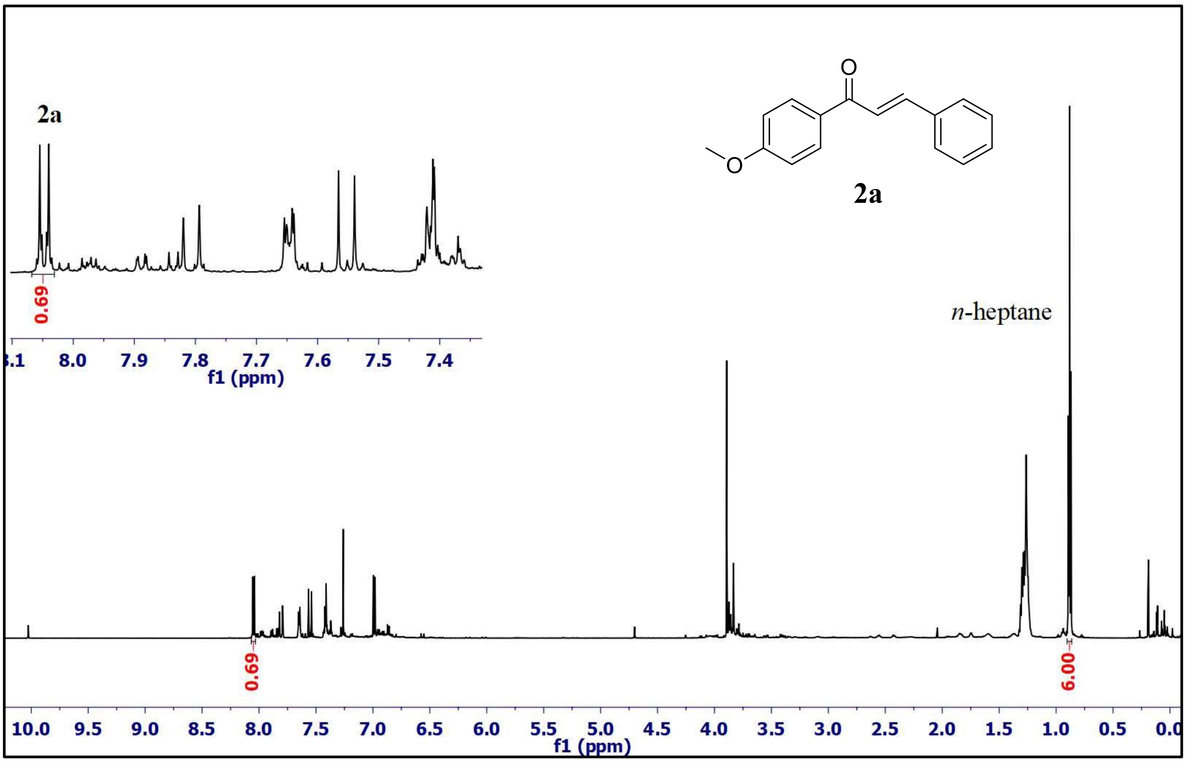

Figure S13: Table S1 entry 13: crude ^{1}H NMR spectrum of the reaction performed on **1a** (0.20 mmol, 1.0 eq.), THF (5 mL), $LiCH_2SiMe_3$ (0.30 mmol, 1.5 eq.), PhCHO (0.24 mmol, 1.2 eq.), 1 h. Reaction performed under air. Yield **2a** 35%.

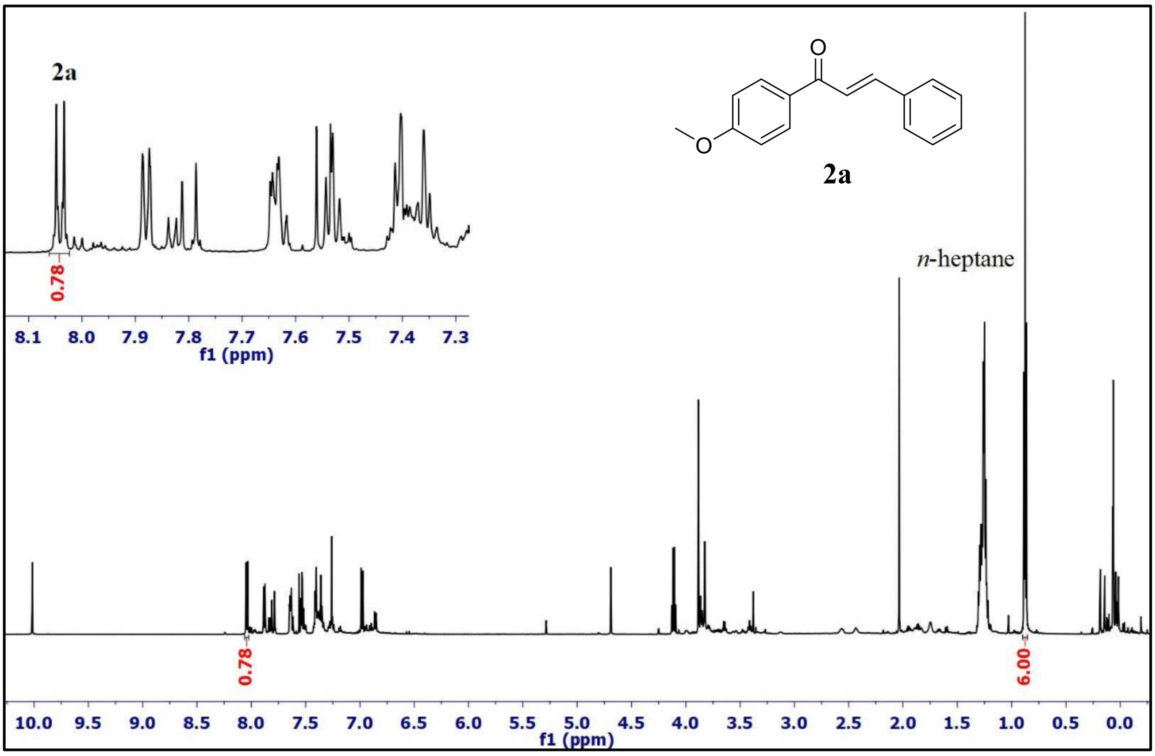

Figure S14: Table S1 entry 14: crude ^{1}H NMR spectrum of the reaction performed on **1a** (0.20 mmol, 1.0 eq.), 2-MeTHF (5 mL), $LiCH_2SiMe_3$ (0.30 mmol, 1.5 eq.), PhCHO (0.24 mmol, 1.2 eq.), 1 h. Reaction performed under air. Yield **2a** 39%.

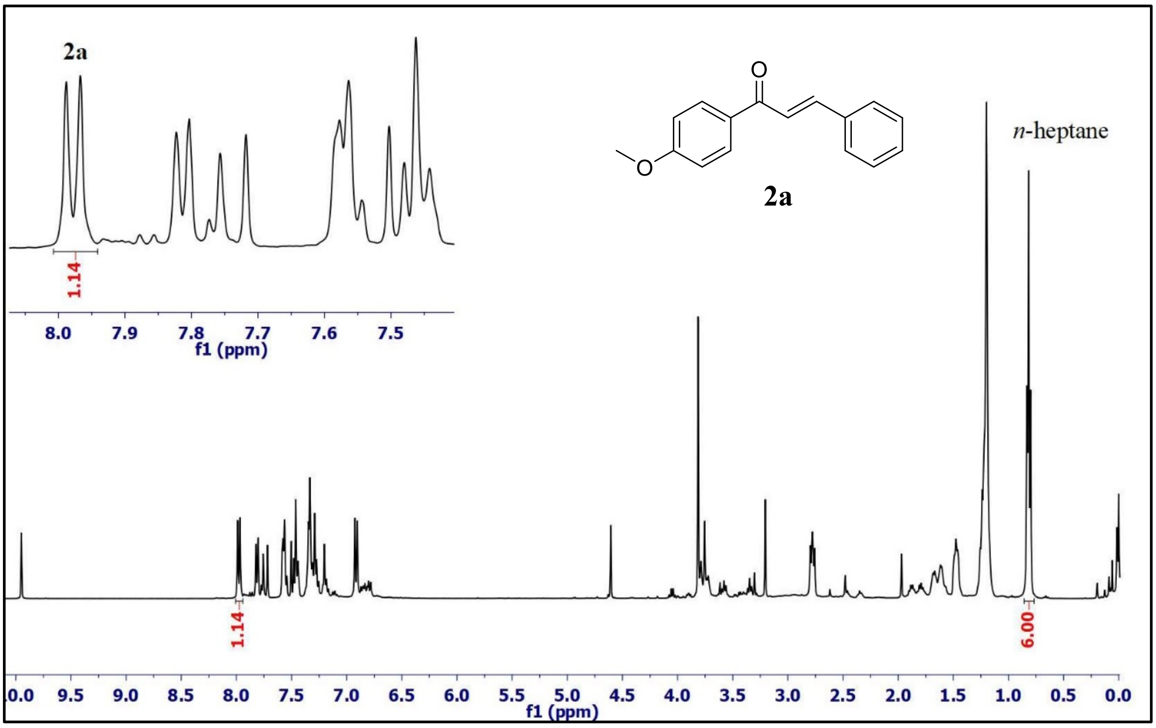

Figure S15: Table S1 entry 15: crude ^{1}H NMR spectrum of the reaction performed on **1a** (0.20 mmol, 1.0 eq.), CPME (5 mL), quinuclidine (0.30 mmol, 1.5 eq.), $LiCH_2SiMe_3$ (0.30 mmol, 1.5 eq.), PhCHO (0.24 mmol, 1.2 eq.), 1 h. Yield **2a** 57%.

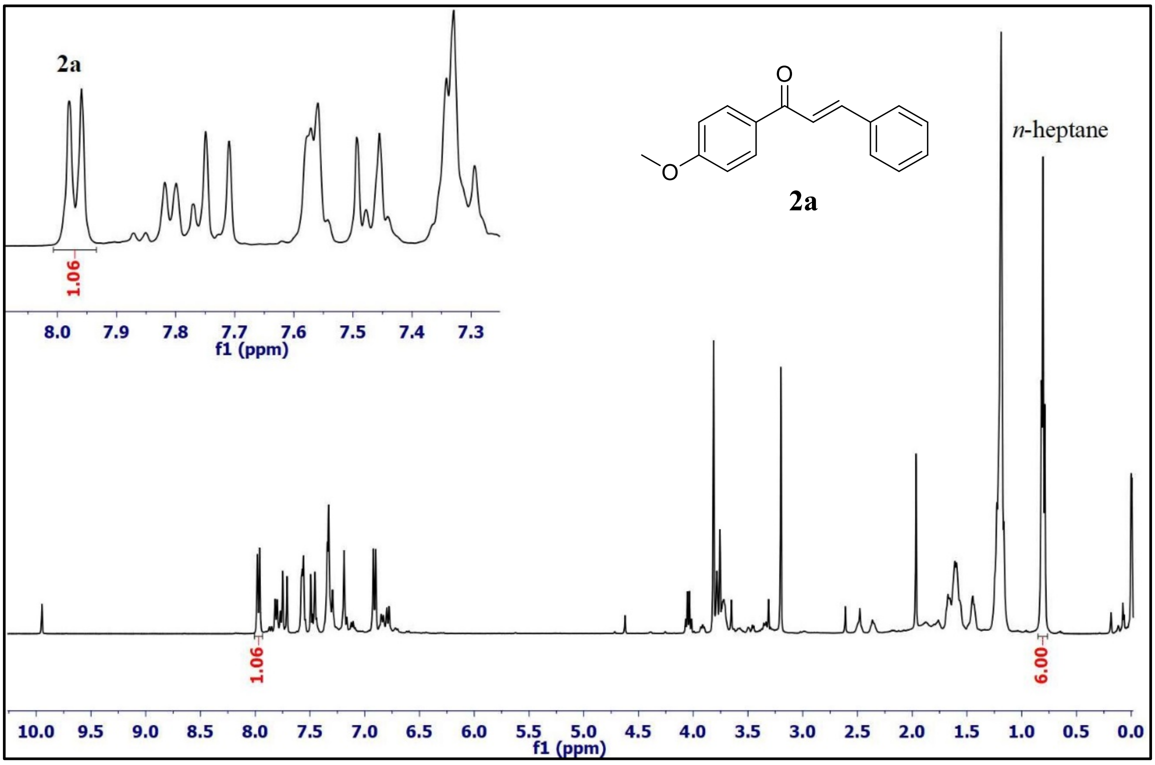

Figure S16: Table S1 entry 16: crude ^{1}H NMR spectrum of the reaction performed on **1a** (0.20 mmol, 1.0 eq.), CPME (5 mL), LiCl (0.30 mmol, 1.5 eq.), $LiCH_2SiMe_3$ (0.30 mmol, 1.5 eq.), PhCHO (0.24 mmol, 1.2 eq.), 1 h. Yield **2a** 53%.

Synthesis and analysis of compounds **2b-2o**

General procedure. To a stirred solution of the appropriate *N*-acylpyrrolidine **1b-1o** (0.2 mmol, 1.0 eq.) in dry CPME (0.04 M, 5 mL) under nitrogen, $LiCH_2SiMe_3$ (0.7 M in hexanes, 0.3 mmol, 1.5 eq., 0.43 mL) was added at room temperature. The resulting mixture was stirred for 30 min, then benzaldehyde (0.24 mmol, 1.2 eq., 24 μL) was added. The mixture was stirred for 1 h and then quenched with H_2O. After extraction with EtOAc (3 x 10 mL), the combined organic layers were dried over Na_2SO_4 and evaporated under reduced pressure. The crude products were purified by flash column chromatography on silica gel.

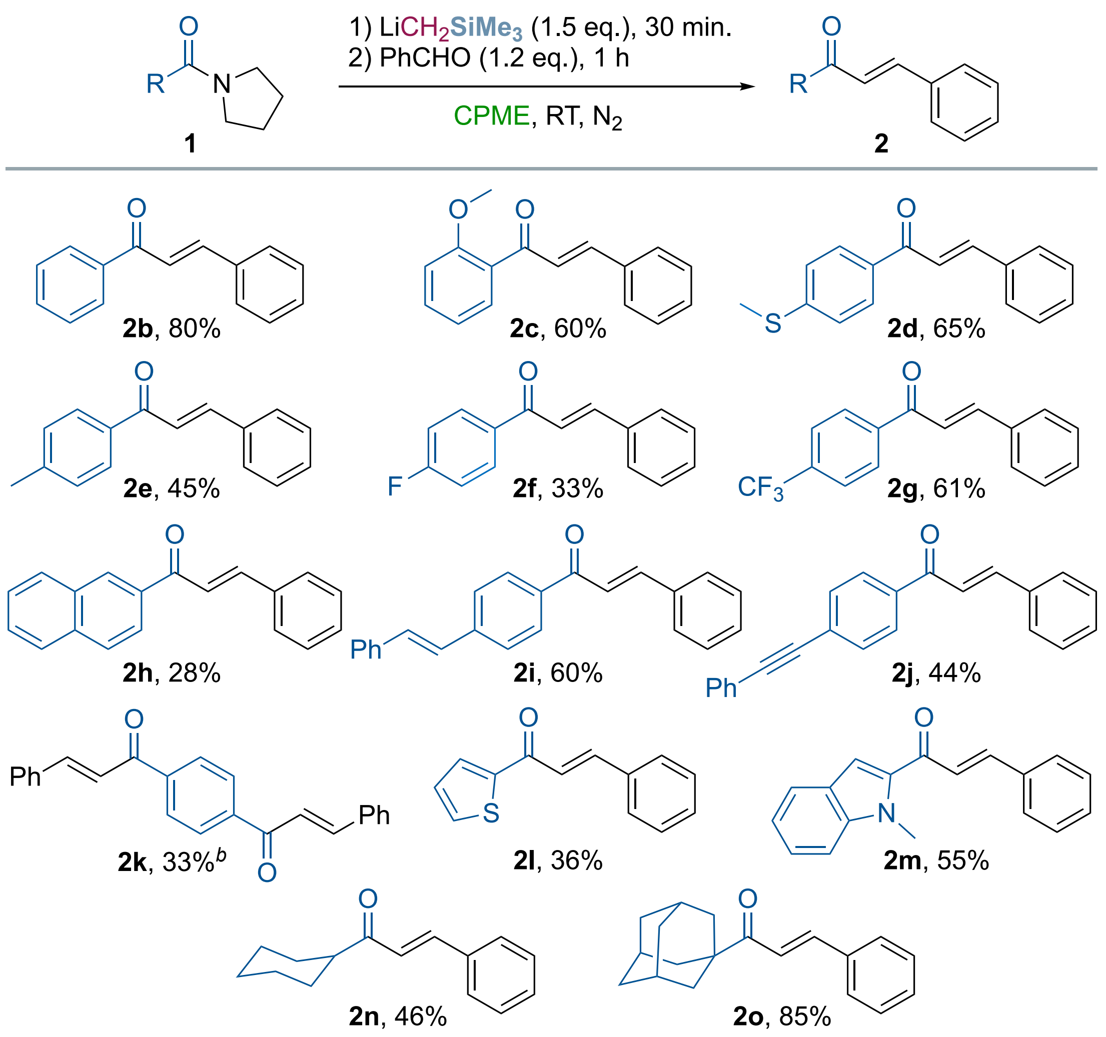

Scheme S4. Synthesis of α,β-unsaturated ketones **2b-2o**. Reaction conditions: **1b-1o** (0.2 mmol, 1.0 eq.), CPME (5 mL), $LiCH_2SiMe_3$ (0.3 mmol, 1.5 eq.), 30 min, RT, under N_2, then PhCHO (0.24 mmol, 1.2 eq.), 1 h. Reported yields refer to isolated products after flash column chromatography on silica gel.

(*E*)-Chalcone (2b): General procedure starting from **1b**. Purification by flash column chromatography on silica gel (petroleum ether/DEE 85/15 v/v) gave **2b** as a white solid (33 mg, 80%, R_f = 0.53 petroleum ether/DEE 85/15 v/v), mp 52.4-53.4 °C.[6] ^{1}H NMR (600 MHz, $CDCl_3$) δ 8.04-8.02 (m, 2H), 7.82 (d, *J* = 15.7 Hz, 1H), 7.66-7.65 (m, 2H), 7.61-7.58 (m, 1H), 7.54 (d, *J* = 15.8 Hz, 1H) superimposed to 7.53-7.50 (m, 2H), 7.43-7.42 (m, 3H). ^{13}C{^{1}H} NMR (150 MHz, $CDCl_3$) δ 190.7, 145.0, 138.4, 135.0, 133.0, 130.7, 129.1, 128.8,

128.7, 128.6, 122.2. EI-MS *m/z* (%): 208 (M^+, 77), 207 (100), 77 (52). HRMS (ESI) *m/z*: $[M+H]^+$ Calcd for $C_{15}H_{13}O$ 209.0961; Found 209.0958.

(*E*)-1-(2-Methoxyphenyl)-3-phenylprop-2-en-1-one (2c): General procedure starting from **1c**. Purification by flash column chromatography on silica gel (petroleum ether/DEE 9/1 v/v) gave **2c** as a yellow liquid (28 mg, 60%, R_f = 0.20 petroleum ether/DEE 9/1 v/v).[11] ^{1}H NMR (600 MHz, $CDCl_3$) δ 7.63 (d, *J* = 15.9 Hz, 1H) superimposed to 7.62 (dd, *J* = 7.6, 1.8 Hz, 1H) 7.60-7.58 (m, 2H), 7.48 (ddd, *J* = 8.4, 7.4, 1.8 Hz, 1H), 7.41-7.38 (m, 3H) superimposed to 7.38 (d, *J* = 15.9 Hz, 1H), 7.05 (td, *J* = 7.5, 1.0 Hz, 1H), 7.00 (dd, *J* = 8.3, 1.0 Hz, 1H), 3.90 (s, 3H). $^{13}C\{^1H\}$ NMR (150 MHz, $CDCl_3$) δ 193.2, 158.2, 143.4, 135.3, 133.0, 130.5, 130.4, 129.4, 129.0, 128.5, 127.2, 120.9, 111.8, 55.9. EI-MS *m/z* (%): 238 (M^+, 74), 237 (87), 77 (100), 135 (89). HRMS (ESI) *m/z*: $[M+H]^+$ Calcd for $C_{16}H_{15}O_2$ 239.1067; Found 239.1067.

(*E*)-1-(4-(Methylthio)phenyl)-3-phenylprop-2-en-1-one (2d): General procedure starting from **1d**. Purification by flash column chromatography on silica gel (petroleum ether/DEE 9/1 v/v) gave **2d** as a yellow solid (33 mg, 65%, R_f = 0.18 petroleum ether/DEE 9/1 v/v), mp. 114.2-115.8 °C.[12] ^{1}H NMR (600 MHz, $CDCl_3$) δ 7.97-7.95 (m, 2H), 7.81 (d, *J* =15.7 Hz, 1H), 7.65-7.64 (m, 2H), 7.53 (d, *J* = 15.7 Hz, 1H), 7.44-7.40 (m, 3H), 7.32-7.30 (m, 2H), 2.54 (s, 3H).$^{13}C\{^1H\}$ NMR (150 MHz, $CDCl_3$) δ 189.4, 145.9, 144.7, 135.1, 134.6, 130.6, 129.1, 128.6, 125.2, 121.9, 15.0. EI-MS *m/z* (%): 254 (M^+, 100), 253 (86), 207 (59). HRMS (ESI) *m/z*: $[M+H]^+$ Calcd for $C_{16}H_{15}OS$ 255.0838; Found 255.0842.

(*E*)-3-Phenyl-1-(*p*-tolyl)prop-2-en-1-one (2e): General procedure starting from **1e**. Purification by flash column chromatography on silica gel (petroleum ether/EtOAc 9/1 v/v) gave **2e** as a white solid (20 mg, 45%, R_f = 0.75 petroleum ether/EtOAc 9/1 v/v), mp. 50.1-53.0 °C.[6] ^{1}H NMR (600 MHz, $CDCl_3$) δ 7.95-7.93 (m, 2H), 7.81 (d, *J* = 15.7 Hz, 1H), 7.66-7.64 (m, 2H), 7.54 (d, *J* = 15.7 Hz, 1H), 7.44-7.40 (m, 3H), 7.32-7.29 (m, 2H), 2.44 (s, 3H). $^{13}C\{^1H\}$ NMR (150 MHz, $CDCl_3$) δ 190.2, 144.6, 143.8, 135.8, 135.1, 130.6, 129.5, 129.1, 128.8, 128.6, 122.2, 21.8. EI-MS *m/z* (%): 222 (M^+, 88), 221 (100), 119 (40), 91 (41). HRMS (ESI) *m/z*: $[M+H]^+$ Calcd for $C_{16}H_{15}O$ 223.1117; Found 223.1121.

(*E*)-1-(4-Fluorophenyl)-3-phenylprop-2-en-1-one (2f): General procedure starting from **1f**. Purification by flash column chromatography on silica gel (petroleum ether/EtOAc 98/2 v/v) gave **2f** as a white solid (15 mg, 33%, R_f = 0.37 petroleum ether/EtOAc 98/2 v/v), mp. 75.1-77.5 °C.[6] ^{1}H NMR (600 MHz, $CDCl_3$) δ 8.08-8.05 (m, 2H), 7.82 (d, *J* = 15.7 Hz, 1H), 7.66-7.64 (m, 2H), 7.51 (d, *J* = 15.7 Hz, 1H), 7.44-7.42 (m, 3H), 7.20-7.16 (m, 2H).$^{13}C\{^1H\}$ NMR (150 MHz, $CDCl_3$) δ 189.0, 165.8 (d, *J* = 254.4 Hz, 1C), 145.2, 134.9, 134.7 (d, *J* = 2.9 Hz, 1C), 131.2 (d, *J* = 9.3 Hz, 2C), 130.8, 129.1, 128.6, 121.7, 115.9 (d, *J* = 21.7 Hz, 2C). ^{19}F NMR (564 MHz, $CDCl_3$) δ -105.45 (s, 1F). EI-MS *m/z* (%): 226 (M^+, 71), 225 (100), 123 (37), 95 (40). HRMS (ESI) *m/z*: $[M+H]^+$ Calcd for $C_{15}H_{12}FO$ 227.0867; Found 227.0868.

(*E*)-3-Phenyl-1-(4-(trifluoromethyl)phenyl)prop-2-en-1-one (2g): General procedure starting from **1g**. Purification by flash column chromatography on silica gel (petroleum ether/DEE 95/5 v/v) gave **2g** as a yellow solid (34 mg, 61%, R_f = 0.43 petroleum ether/DEE 95/5 v/v), mp. 111.2-114.0 °C.[13] ^{1}H NMR (600 MHz, $CDCl_3$) δ 8.11-8.10 (m, 2H), 7.84 (d, *J* = 15.8 Hz, 1H), 7.79-7.77 (m, 2H), 7.68-7.64 (m, 2H), 7.49 (d, *J* = 15.7 Hz, 1H), 7.46-7.43 (m, 3H). ^{13}C{^{1}H} NMR (150 MHz, $CDCl_3$) δ 189.8, 146.3, 141.2, 134.7, 134.2 (q, *J* = 32.8 Hz, 1C), 131.1, 129.2, 128.9, 128.8, 125.8 (q, *J* = 4.8 Hz, 2C), 123.8 (q, *J* = 272.9 Hz, 1C), 121.7. ^{19}F NMR (564 MHz, $CDCl_3$) δ -62.90 (s, 3F). EI-MS *m/z* (%): 276 (M^+, 71), 275 (100), 145 (32), 131 (27). HRMS (ESI): *m*/*z*: $[M+H]^+$ Calcd for $C_{16}H_{12}F_3O$ 277.0835; Found 277.0839.

(*E*)-1-(Naphthalen-2-yl)-3-phenylprop-2-en-1-one (2h): General procedure starting from **1h**. Purification by flash column chromatography on silica gel (petroleum ether/EtOAc 9/1 v/v) gave **2h** as a white solid (15 mg, 28%, R_f = 0.3 petroleum ether/EtOAc 9/1 v/v), mp. 148.4-150.1 °C.[6] ^{1}H NMR (600 MHz, $CDCl_3$) δ 8.55 (s, 1H), 8.11 (dd, *J* = 8.6, 1.8 Hz, 1H), 8.01 (d, *J* = 8.1 Hz, 1H), 7.96 (d, *J* = 8.6 Hz, 1H), 7.93-7.86 (m, 1H) superimposed to 7.89 (d, *J* = 15.0 Hz, 1H), 7.72-7.70 (m, 3H), 7.62 (ddd, *J* = 8.1, 6.8, 1.4 Hz, 1H), 7.58 (ddd, *J* = 8.1, 6.8, 1.4 Hz, 1H), 7.47-7.42 (m, 3H). ^{13}C{^{1}H} NMR (150 MHz, $CDCl_3$) δ 190.3, 144.8, 135.5, 135.5, 134.9, 132.5, 130.6, 129.9, 129.5, 129.0, 128.6, 128.5, 128.4, 127.8, 126.8, 124.4, 122.1. HRMS (ESI) *m*/*z*: $[M+Na]^+$ Calcd for $C_{19}H_{14}ONa$ 281.0937; Found 281.0947.

(*E*)-3-Phenyl-1-(4-((*E*)-styryl)phenyl)prop-2-en-1-one (2i): General procedure starting from **2i**. Purification by flash column chromatography on silica gel (petroleum ether/EtOAc 95/5 v/v) gave **2i** as a white solid (37 mg, 60%, R_f = 0.23 petroleum ether/EtOAc 95/5 v/v), mp. 162.4-166.0 °C. ^{1}H NMR (600 MHz, $CDCl_3$) δ 8.06-8.03 (m, 2H), 7.84 (d, *J* = 15.7 Hz, 1H), 7.68-7.65 (m, 2H), 7.65-7.62 (m, 2H), 7.59-7.54 (m, 3H), 7.45-7.42 (m, 3H), 7.39 (t, *J* = 7.7 Hz, 2H), 7.33-7.30 (m, 1H), 7.26 (d, *J* = 16.3 Hz, 1H), 7.16 (d, *J* = 16.3 Hz, 1H). ^{13}C{^{1}H} NMR (150 MHz, $CDCl_3$) δ 189.7, 144.8, 142.0, 137.1, 136.9, 135.1, 131.6, 130.7, 129.2, 129.1, 129.0, 128.6, 128.4, 127.7, 127.0, 126.7, 122.1. HRMS (ESI) *m*/*z*: $[M+H]^+$ Calcd for $C_{23}H_{19}O$ 311.1430; Found 311.1437.

(*E*)-3-Phenyl-1-(4-(phenylethynyl)phenyl)prop-2-en-1-one (2j): General procedure starting from **1j**. Purification by flash column chromatography on silica gel (petroleum ether/EtOAc 98/2 v/v) gave **2j** as a white solid (27 mg, 44%, R_f = 0.25 petroleum ether/EtOAc 98/2 v/v), mp. 147.4-150.0 °C.[14] ^{1}H NMR (600 MHz, $CDCl_3$) δ 8.03-8.01 (m, 2H), 7.84 (d, *J* = 15.7 Hz, 1H), 7.67-7.65 (m, 4H), 7.58-7.56 (m, 2H) superimposed to 7.54 (d, *J* = 15.6 Hz, 1H), 7.45-7.42 (m, 3H), 7.39-7.37 (m, 3H). ^{13}C{^{1}H} NMR (150 MHz, $CDCl_3$) δ 189.7, 145.3, 137.5, 135.0, 132.0, 130.8, 129.1, 129.0, 128.7, 128.6, 128.6, 128.1, 122.9, 122.0, 92.8, 88.9. HRMS (ESI) *m*/*z*: $[M+H]^+$ Calcd for $C_{23}H_{17}O$ 309.1274; Found 309.1283.

(2*E*,2'*E*)-1,1'-(1,4-Phenylene)bis(3-phenylprop-2-en-1-one) (2k): General procedure starting from **1k**, $LiCH_2SiMe_3$ (0.6 mmol, 0.86 mL) and benzaldehyde (0.48 mmol, 48 μL). Purification by flash column

chromatography on silica gel (petroleum ether/EtOAc 9/1 v/v) gave **2k** as a white solid (22 mg, 33%, R_f = 0.15 petroleum ether/EtOAc 9/1 v/v), mp. 146.8-148.2 °C.[7] ^{1}H NMR (600 MHz, $CDCl_3$) δ 8.13 (s, 4H), 7.85 (d, *J* = 15.7 Hz, 2H), 7.68-7.67 (m, 4H), 7.54 (d, *J* = 15.8 Hz, 2H), 7.47-7.42 (m, 6H). $^{13}C\{^1H\}$ NMR (150 MHz, $CDCl_3$) δ 190.1, 145.9, 141.3, 134.6, 130.3, 129.1, 128.7, 128.6, 121.9. HRMS (ESI) *m/z*: $[M+Na]^+$ Calcd for $C_{24}H_{18}O_2Na$ 361.1199; Found 361.1206.

(*E*)-3-Phenyl-1-(thiophen-2-yl)prop-2-en-1-one (2l): General procedure starting from **1l**. Purification by flash column chromatography on silica gel (petroleum ether/EtOAc 9/1 v/v) gave **2l** as a white solid (16 mg, 36%, R_f = 0.37 PE/EtOAc 9/1 v/v), mp. 74.0-76.5 °C.[6] ^{1}H NMR (600 MHz, $CDCl_3$) δ 7.88 (d, *J* = 1.1 Hz, 1H) superimposed to 7.86 (d, *J* = 15.6 Hz, 1H), 7.69 (dd, *J* = 5.0, 1.1 Hz, 1H), 7.66-7.64 (m, 2H), 7.45-7.41 (m, 4H), 7.19 (dd, *J* = 4.9, 3.8 Hz, 1H). $^{13}C\{^1H\}$ NMR (150 MHz, $CDCl_3$) δ 182.2, 145.7, 144,2, 134.9, 134.0, 131.95, 130.8, 129.1, 128.6, 128.4, 121.8. EI-MS *m/z* (%): 214 (M^+, 76), 213 (100), 185 (30), 111 (40). HRMS (ESI) *m/z*: $[M+H]^+$ Calcd for $C_{13}H_{11}OS$ 215.0525; Found 215.0526.

(*E*)-1-(1-Methyl-1*H*-indol-2-yl)-3-phenylprop-2-en-1-one (2m): General procedure starting from **1m**. Purification by flash column chromatography on silica gel (petroleum ether/EtOAc 98/2 v/v) gave **2m** as a yellow solid (29 mg, 55%, R_f = 0.20 petroleum ether/EtOAc 98/2 v/v), which was further purified by recrystallization with *n*-hexane yielding **2m** as a yellow solid. mp. 90.1-92.6 °C (*n*-hexane).[15] ^{1}H NMR (600 MHz, $CDCl_3$) δ 7.82 (d, *J* = 15.6 Hz, 1H), 7.74 (dt, *J* = 8.1, 1.0 Hz, 1H), 7.68-7.66 (m, 2H), 7.58 (d, *J* = 15.6 Hz, 1H), 7.45-7.40 (m, 6H), 7.18 (ddd, *J* = 7.9, 6.2, 1.6 Hz, 1H), 4.17 (s, 3H). $^{13}C\{^1H\}$ NMR (150 MHz, $CDCl_3$) δ 182.9, 142.9, 140.7, 136.4, 135.1, 130.5, 129.1, 128.5, 126.2, 126.1, 124.0, 123.1, 121.0, 111.7, 110.6, 32.4. EI-MS *m/z* (%): 261 (M^+, 100), 260 (33), 232 (26), 184 (23), 170 (23), 144 (24), 130 (29), 89 (43).

(*E*)-1-Cyclohexyl-3-phenylprop-2-en-1-one (2n): General procedure starting from **1n**. Purification by flash column chromatography on silica gel (petroleum ether/EtOAc 95/5 v/v) gave **2n** as a yellow liquid (20 mg, 46%, R_f = 0.30 petroleum ether/EtOAc 95/5 v/v).[16] ^{1}H NMR (600 MHz, $CDCl_3$) δ 7.59 (d, *J* = 16.0 Hz, 1H), 7.57-7.55 (m, 2H), 7.40-7.37 (m, 3H), 6.82 (d, *J* = 16.0 Hz, 1H), 2.66 (tt, *J* = 11.4, 3.4 Hz, 1H), 1.92-1.88 (m, 2H), 1.86-1.80 (m, 2H), 1.74-1.69 (m, 1H), 1.43 (tdd, *J* = 12.7, 11.5, 2.9 Hz, 2H), 1.34 (qt, *J* = 12.5, 3.4 Hz, 2H), 1.25 (qt, *J* = 12.5, 3.4 Hz, 1H). $^{13}C\{^1H\}$ NMR (150 MHz, $CDCl_3$) δ 203.3, 142.4, 134.9, 130.4, 129.0, 128.4, 124.9, 49.5, 28.9, 26.0, 25.9. EI-MS *m/z* (%): 214 (M^+, 21), 131 (100), 103 (29), 77 (14). HRMS (ESI) *m/z*: $[M+H]^+$ Calcd for $C_{15}H_{19}O$ 215.1430; Found 215.1431.

(*E*)-1-(Adamantan-1-yl)-3-phenylprop-2-en-1-one (2o): General procedure starting from **1o**. Purification by flash column chromatography on silica gel (*n*-hexane/EtOAc 95/5 v/v) gave a **1o** as a white solid (45 mg, 85%, R_f = 0.35 (*n*-hexane/EtOAc 95/5 v/v), mp. 86.1-88.4 °C.[17] ^{1}H NMR (600 MHz, $CDCl_3$) δ 7.67 (d, *J* = 15.6 Hz, 1H), 7.59-7.56 (m, 2H), 7.40-7.37 (m, 3H), 7.18 (d, *J* = 15.6 Hz, 1H), 2.10-2.09 (m, 3H), 1.91-1.87 (m, 6H), 1.81-1.76 (m, 3H),1.76-1.71 (m, 3H). $^{13}C\{^1H\}$ NMR (150 MHz, $CDCl_3$) δ 204.0, 143.0, 135.2, 130.3, 129.0,

128.4, 120.5, 45.7, 38.2, 36.7, 28.1. EI-MS *m/z* (%): 266 (M^+, 35), 238 (100), 93 (53), 79 (55). HRMS (ESI) *m*/*z*: $[M+H]^+$ Calcd for $C_{19}H_{23}O$ 267.1743; Found 267.1747.

^{1}H and ^{13}C NMR spectra of compounds **2b-2o**

(*E*)-Chalcone (2b)

^{1}H NMR (600 MHz, $CDCl_3$)

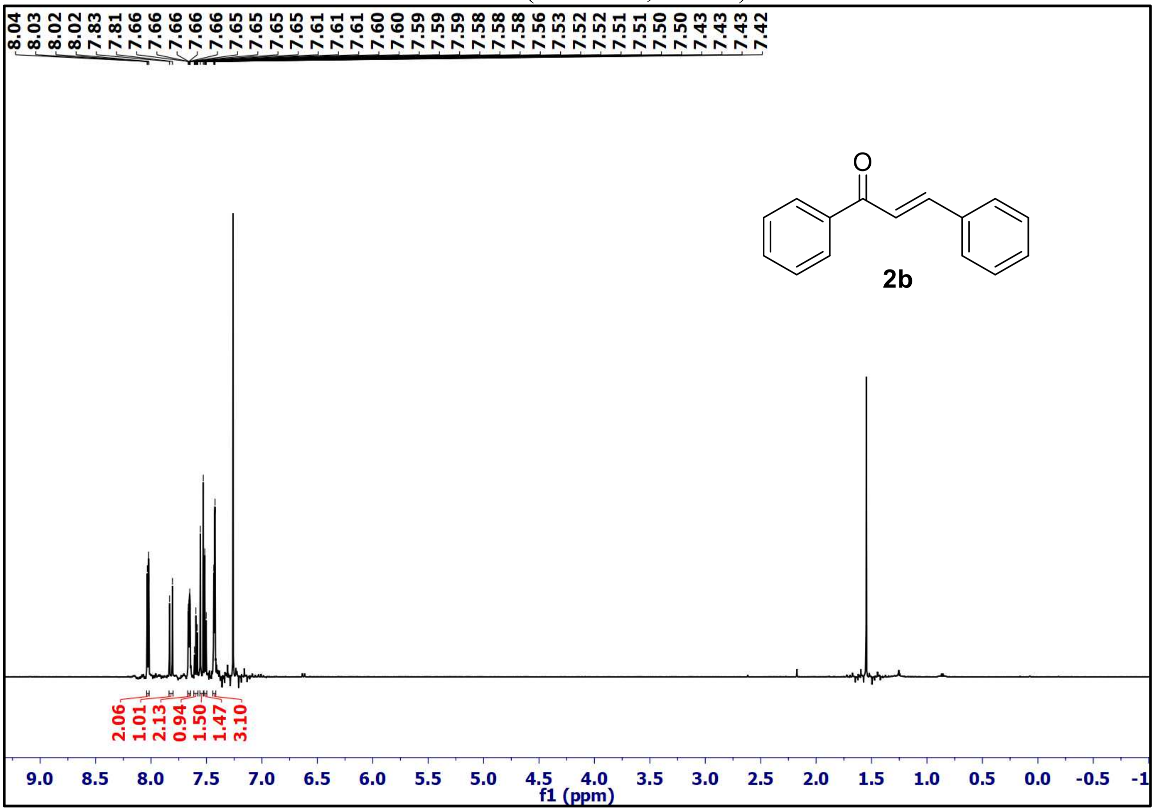

^{13}C NMR (150 MHz, $CDCl_3$)

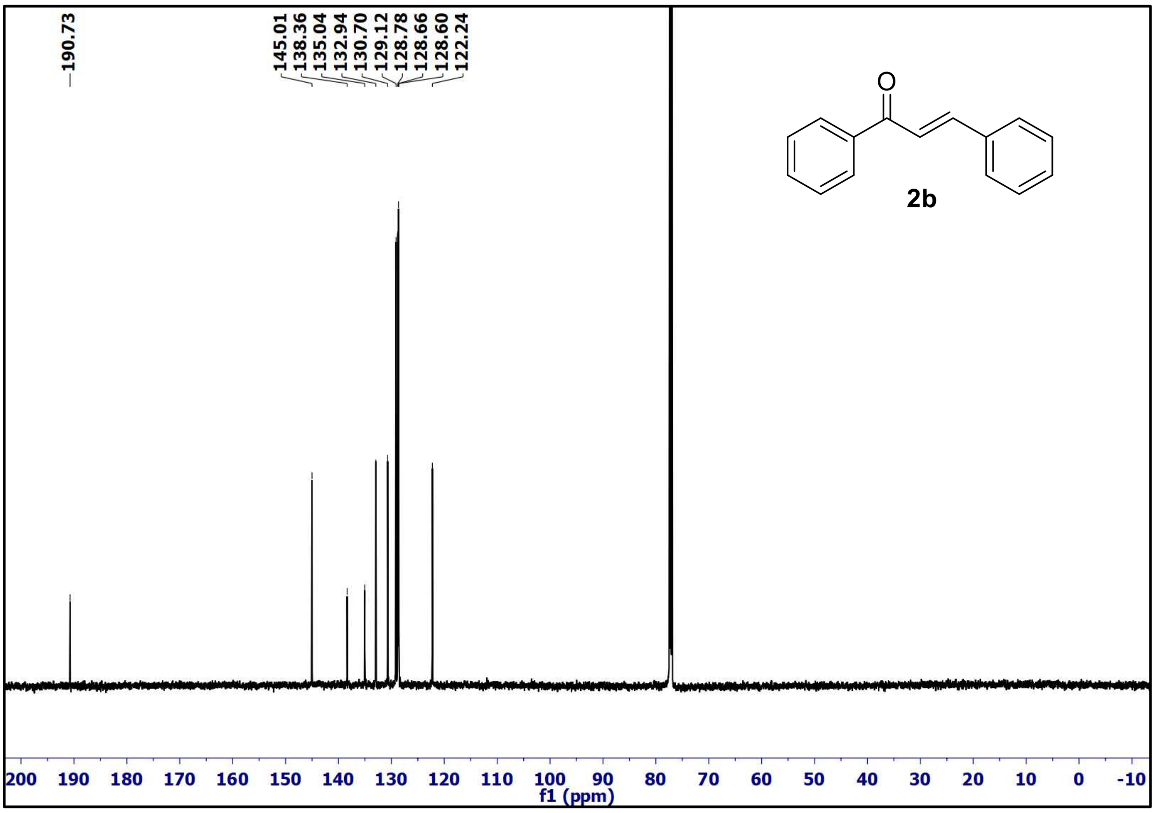

(*E*)-1-(2-Methoxyphenyl)-3-phenylprop-2-en-1-one (2c)

^{1}H NMR (600 MHz, $CDCl_3$)

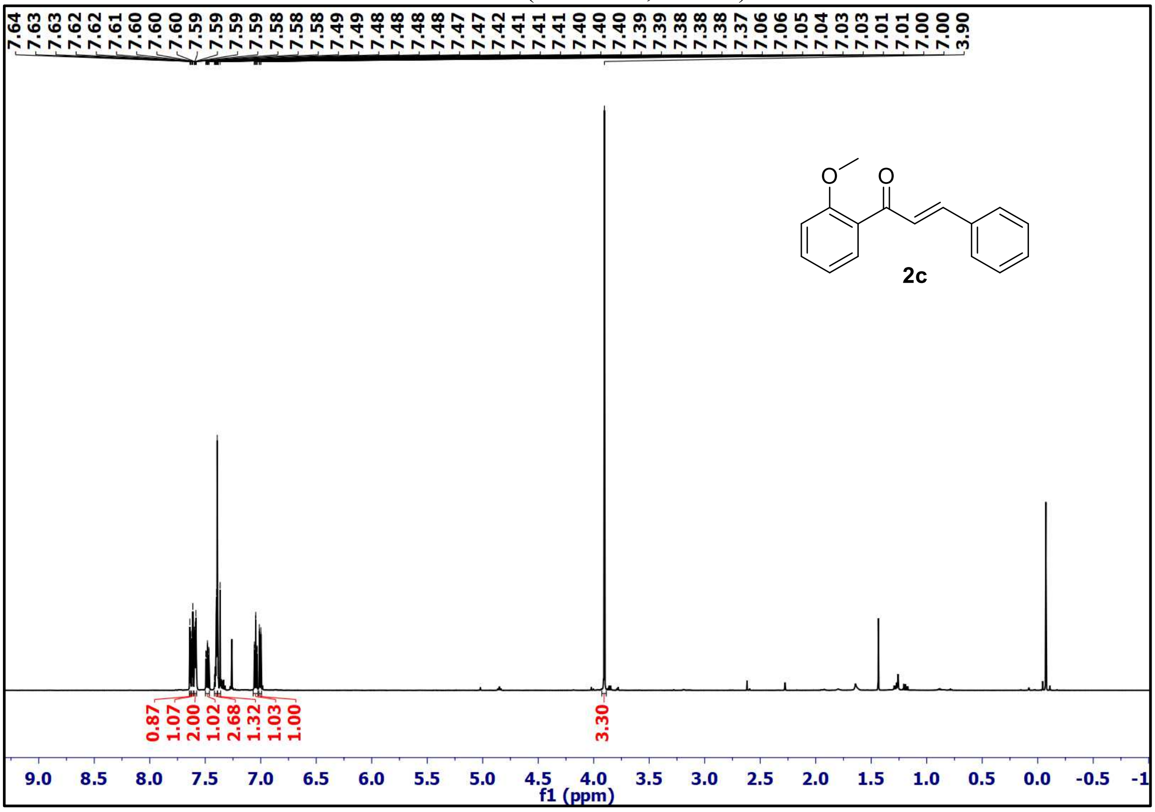

^{13}C NMR (150 MHz, $CDCl_3$)

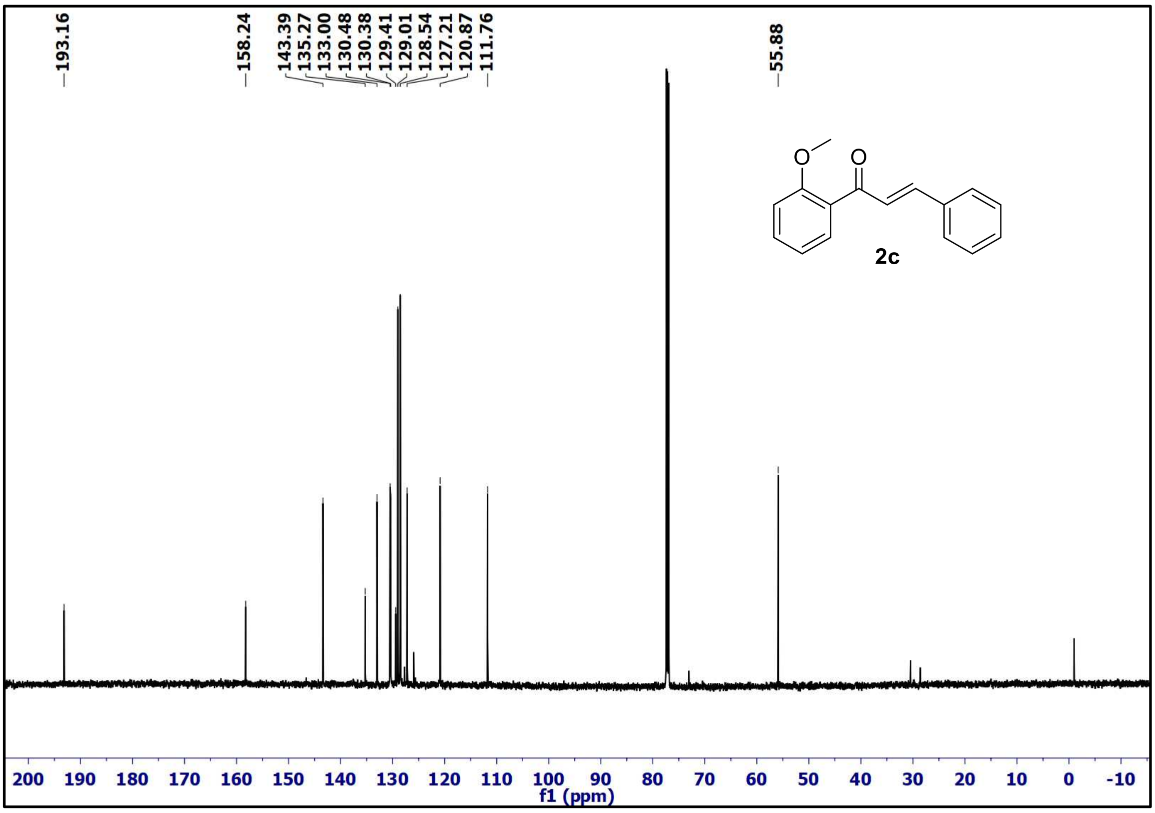

(*E*)-1-(4-(Methylthio)phenyl)-3-phenylprop-2-en-1-one (2d)

^{1}H NMR (600 MHz, $CDCl_3$)

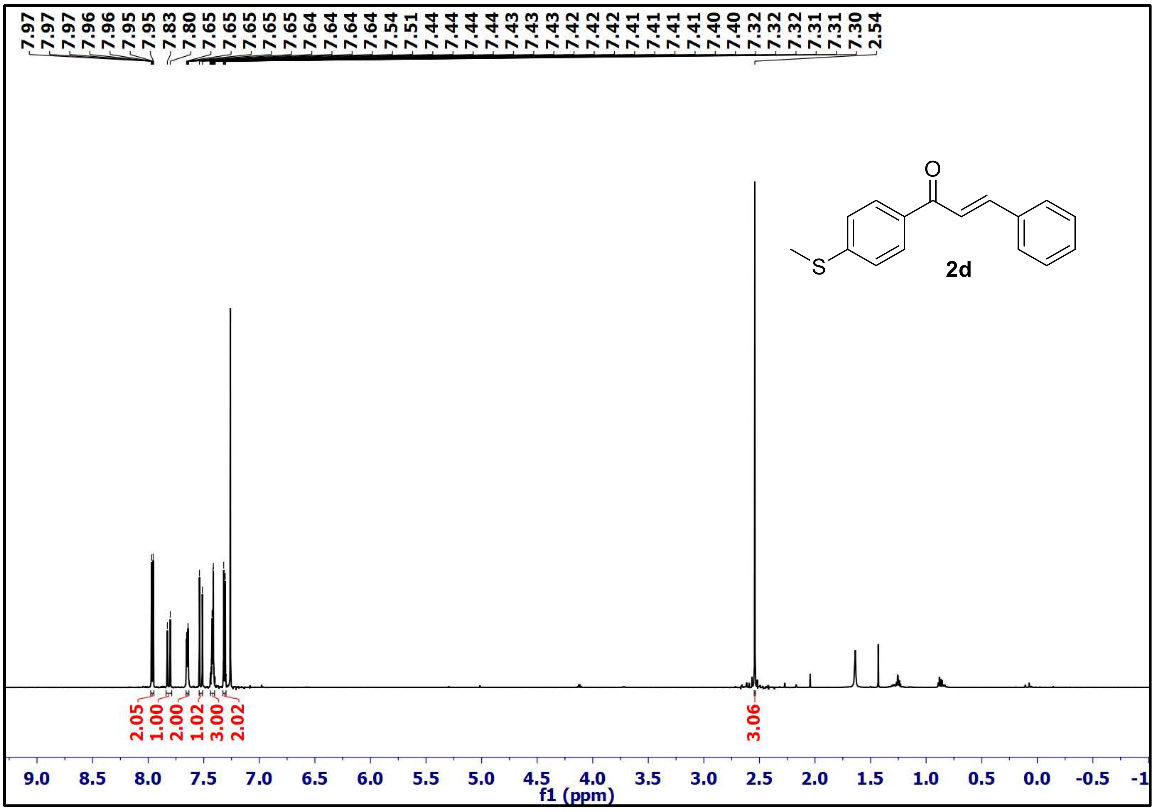

^{13}C NMR (150 MHz, $CDCl_3$)

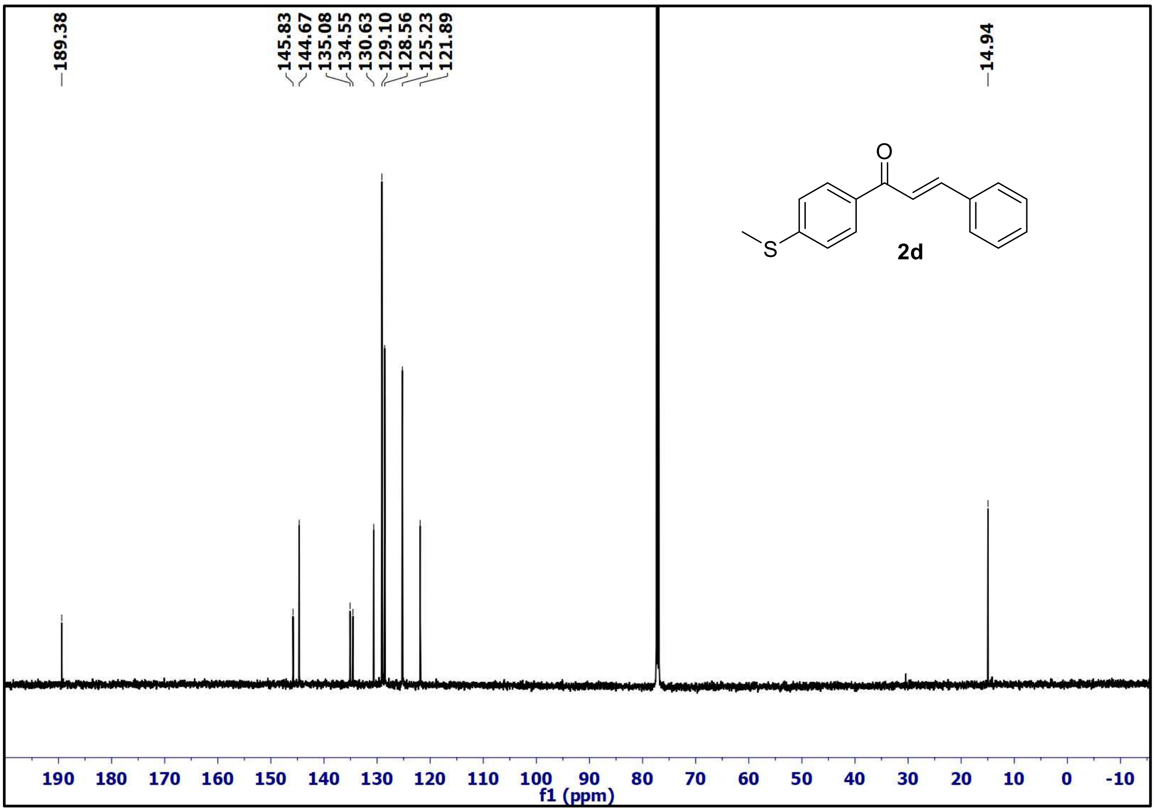

(*E*)-3-Phenyl-1-(*p*-tolyl)prop-2-en-1-one (2e)

^{1}H NMR (600 MHz, $CDCl_3$)

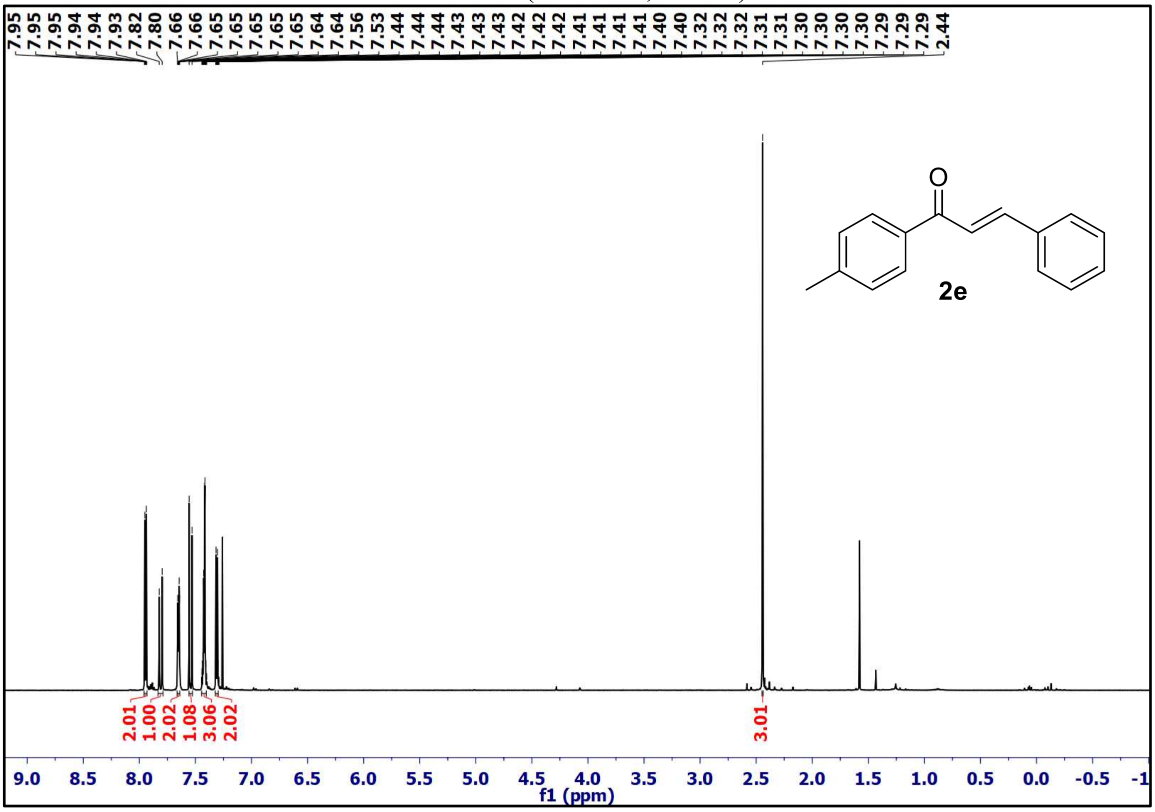

^{13}C NMR (150 MHz, $CDCl_3$)

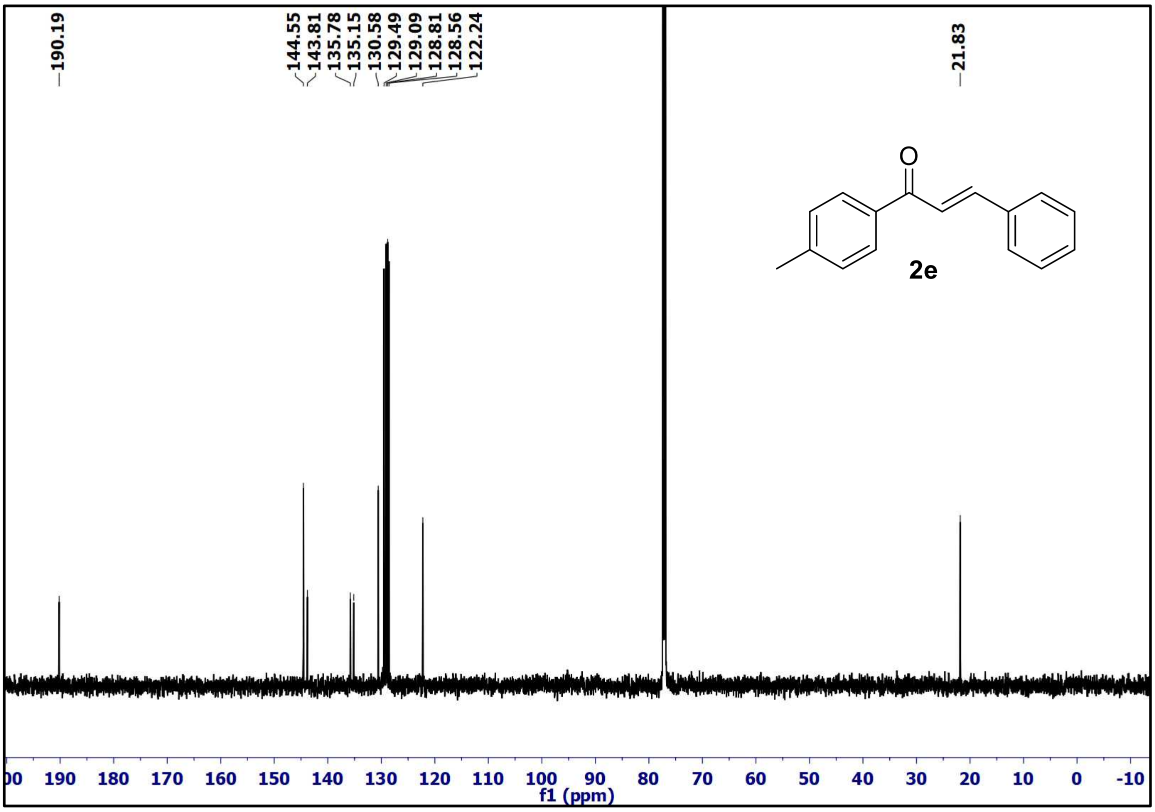

(*E*)-1-(4-Fluorophenyl)-3-phenylprop-2-en-1-one (2f)

^{1}H NMR (600 MHz, $CDCl_3$)

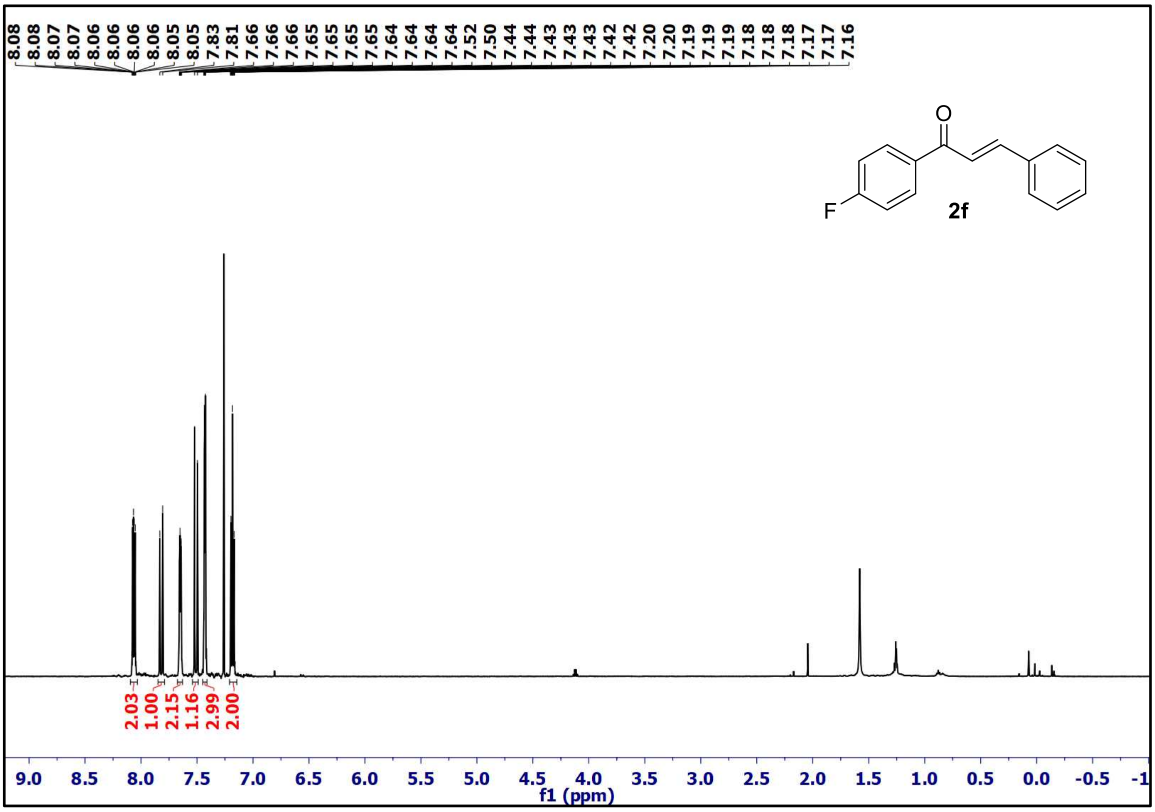

^{13}C NMR (150 MHz, $CDCl_3$)

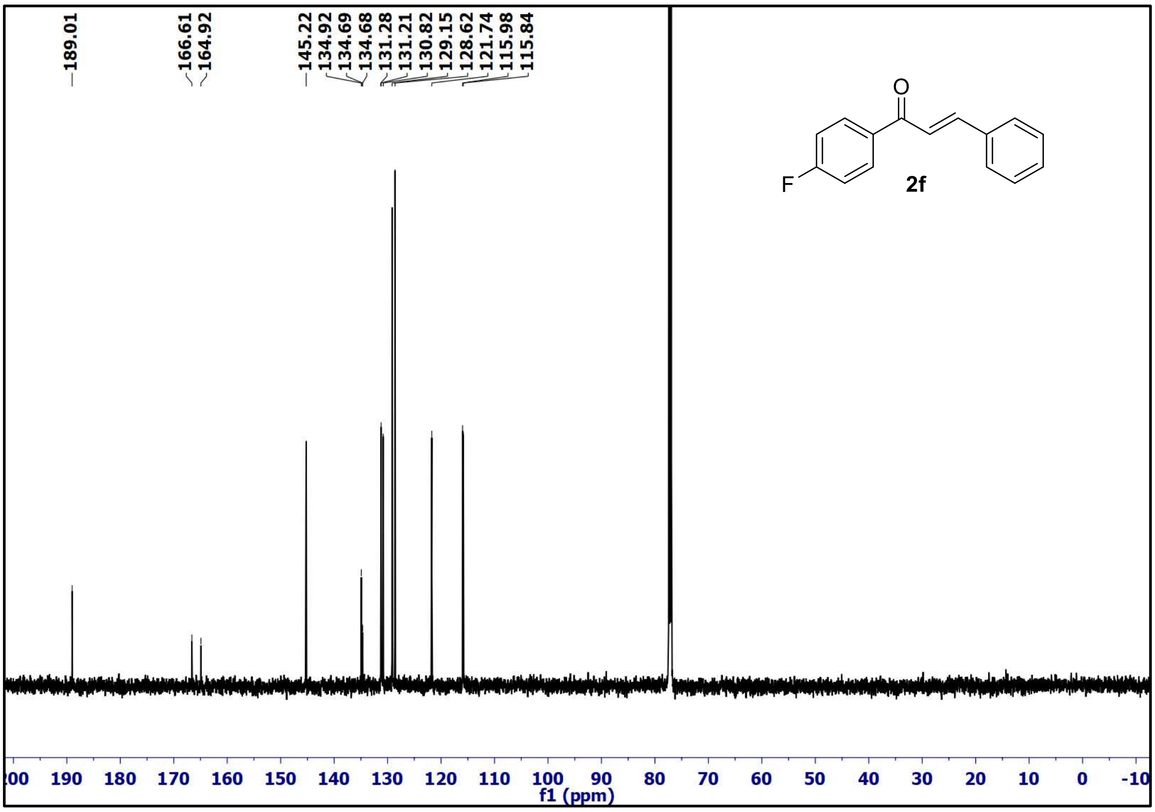

^{19}F NMR (564 MHz, $CDCl_3$)

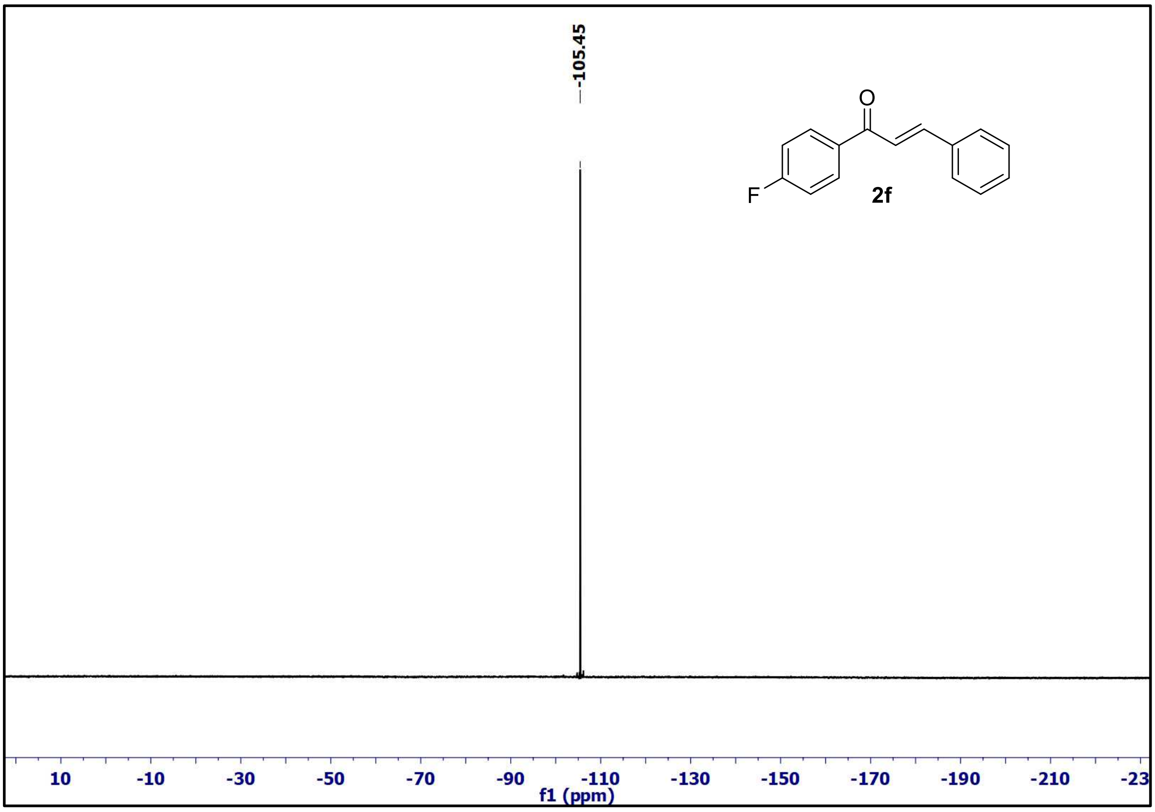

(*E*)-3-Phenyl-1-(4-(trifluoromethyl)phenyl)prop-2-en-1-one (2g)

^{1}H NMR (600 MHz, $CDCl_3$)

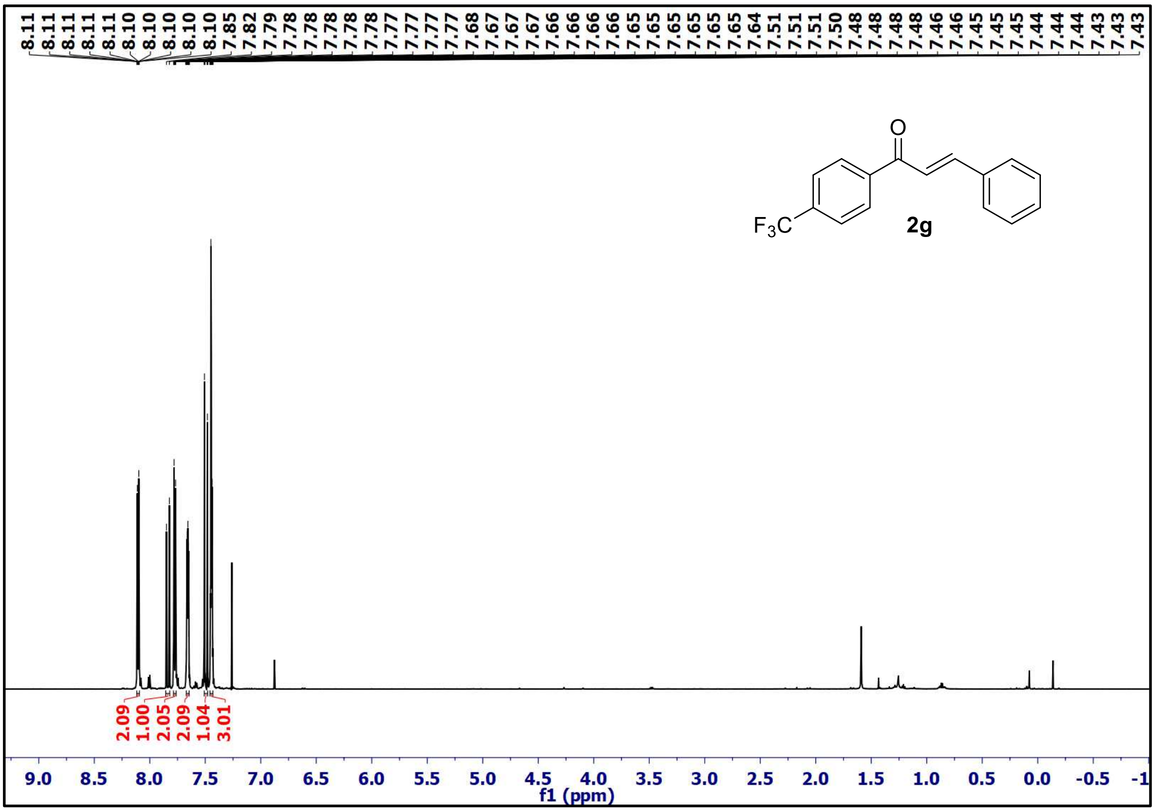

^{13}C NMR (150 MHz, $CDCl_3$)

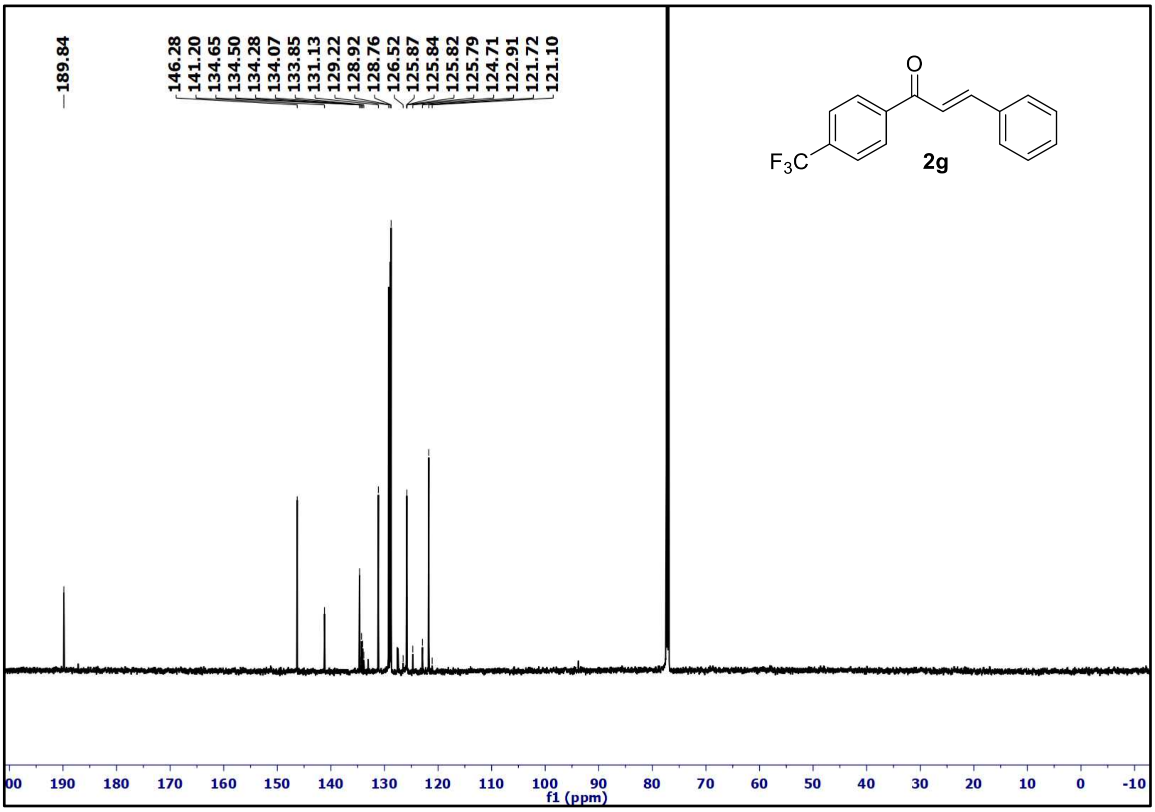

[19]**F NMR** (564 MHz, $CDCl_3$)

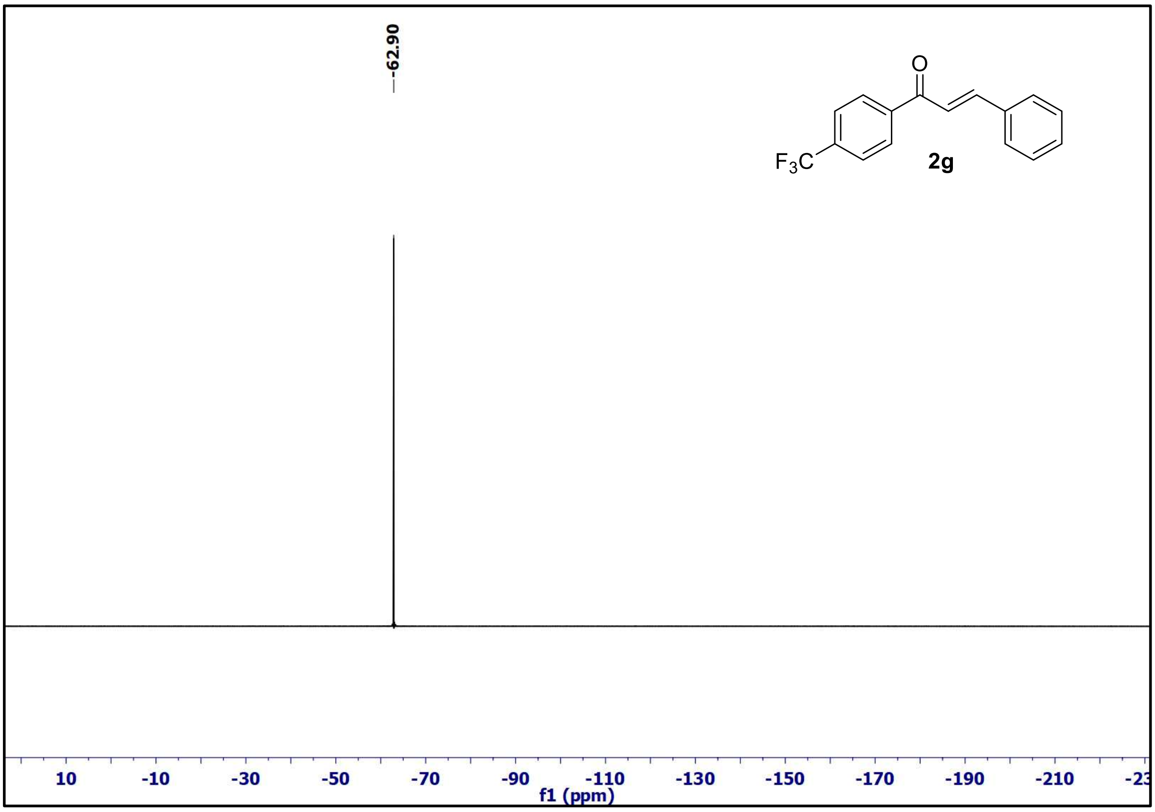

(*E*)-1-(Naphthalen-2-yl)-3-phenylprop-2-en-1-one (2h)

^{1}H NMR (600 MHz, $CDCl_3$)

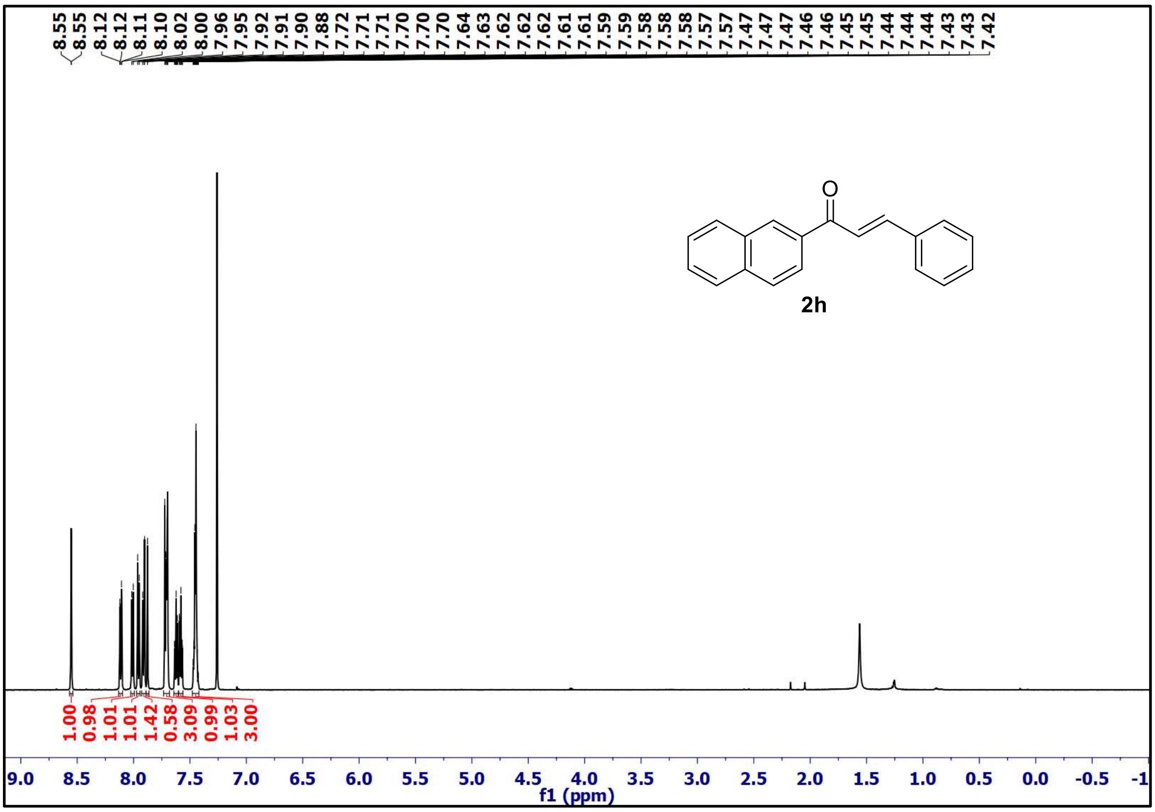

^{13}C NMR (150 MHz, $CDCl_3$)

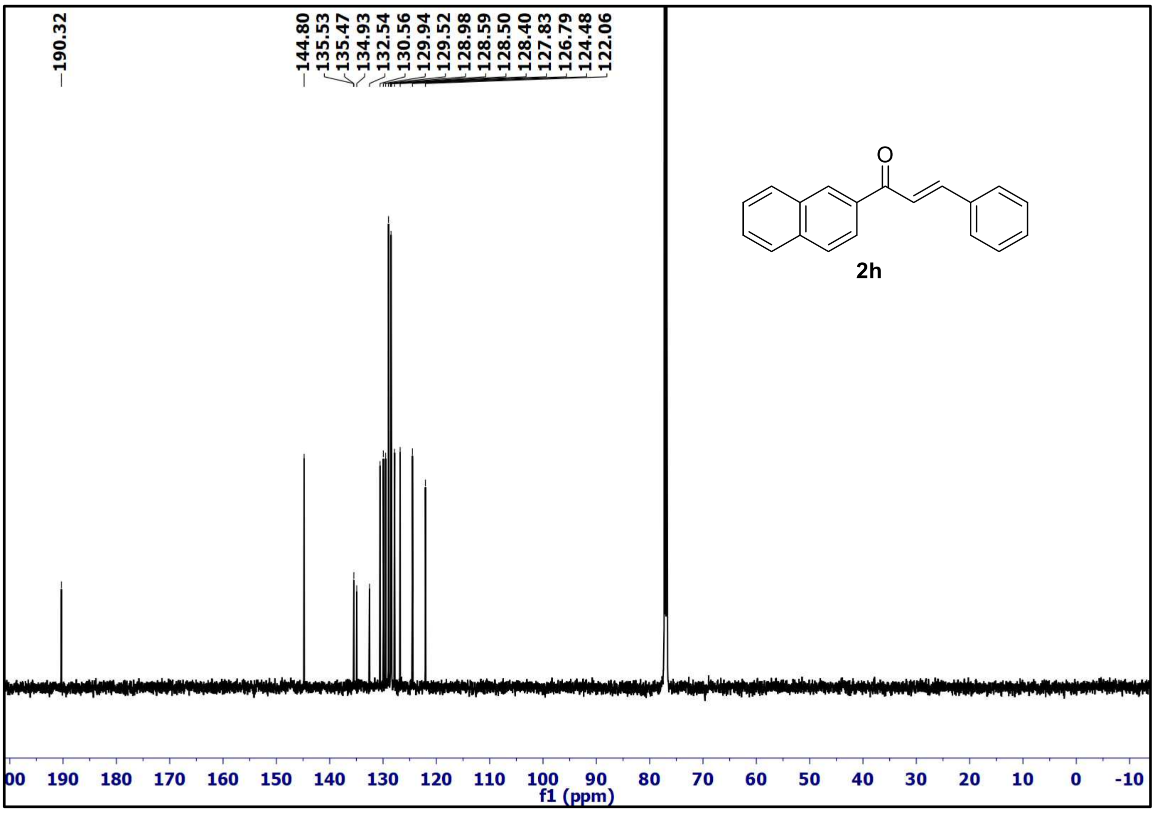

(*E*)-3-Phenyl-1-(4-((*E*)-styryl)phenyl)prop-2-en-1-one (2i)

^{1}H NMR (600 MHz, $CDCl_3$)

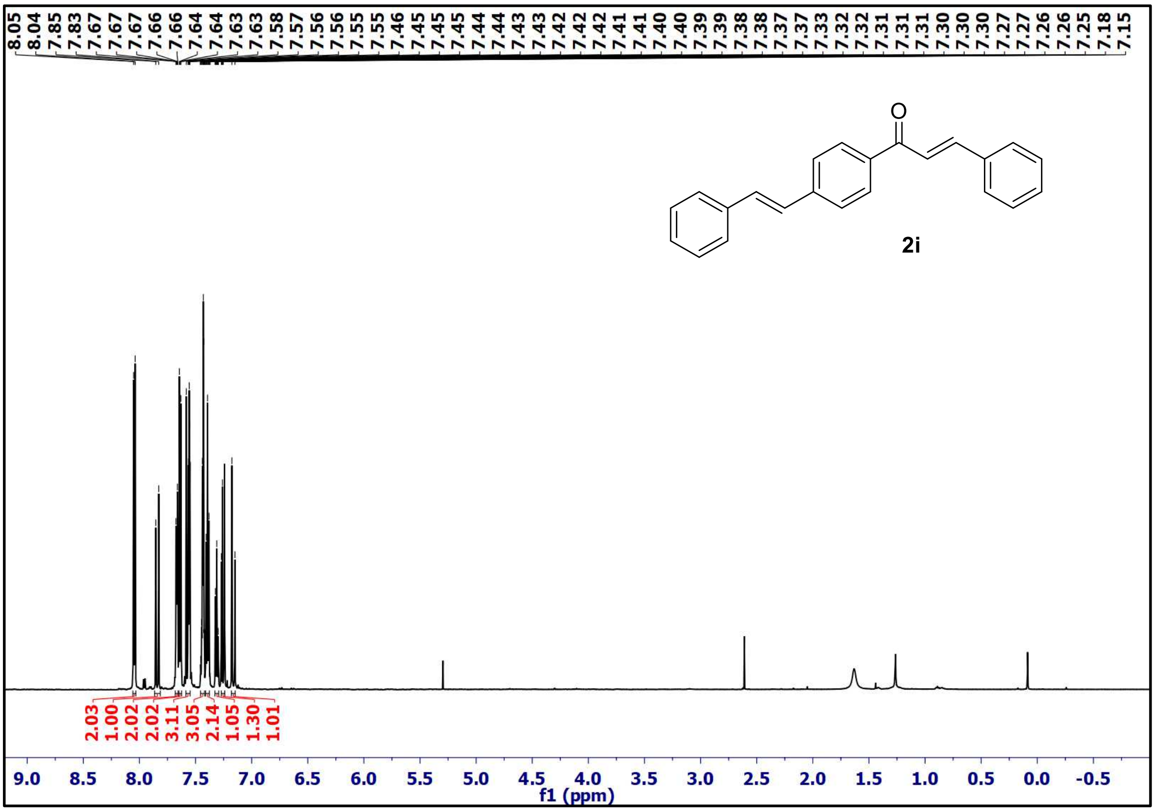

^{13}C NMR (150 MHz, $CDCl_3$)

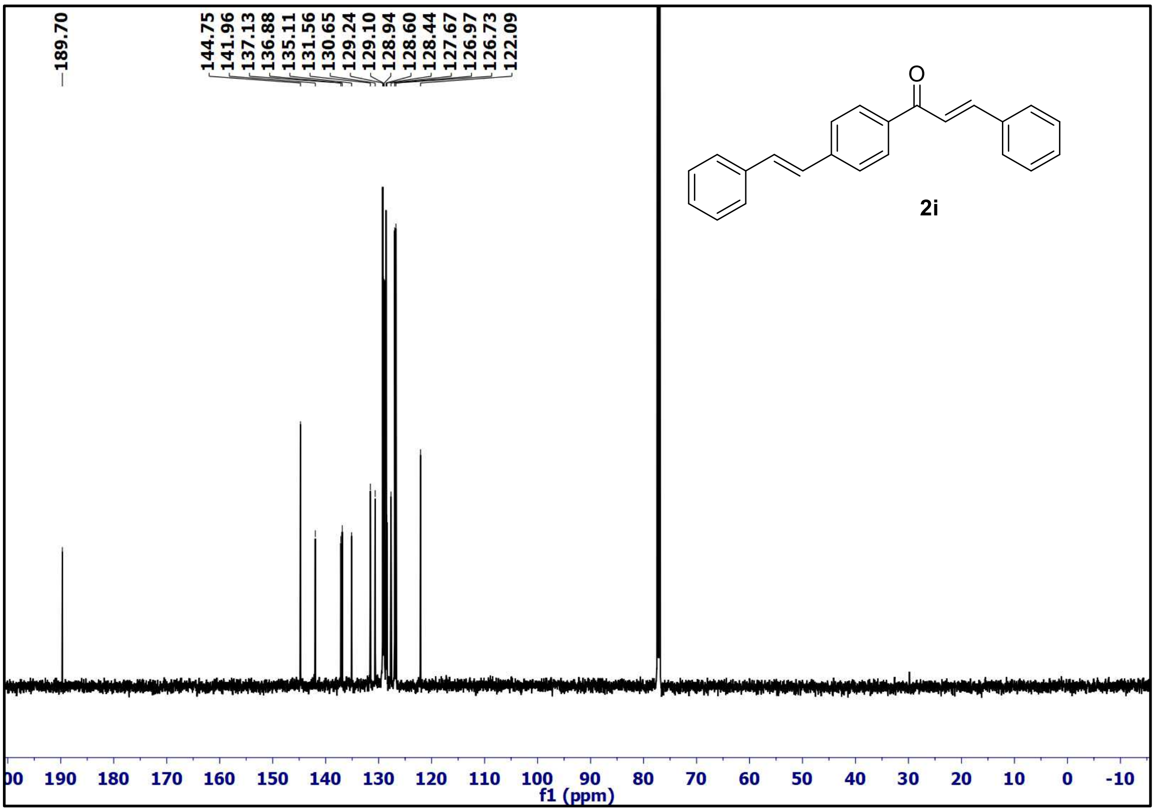

(*E*)-3-Phenyl-1-(4-(phenylethynyl)phenyl)prop-2-en-1-one (2j)

^{1}H NMR (600 MHz, $CDCl_3$)

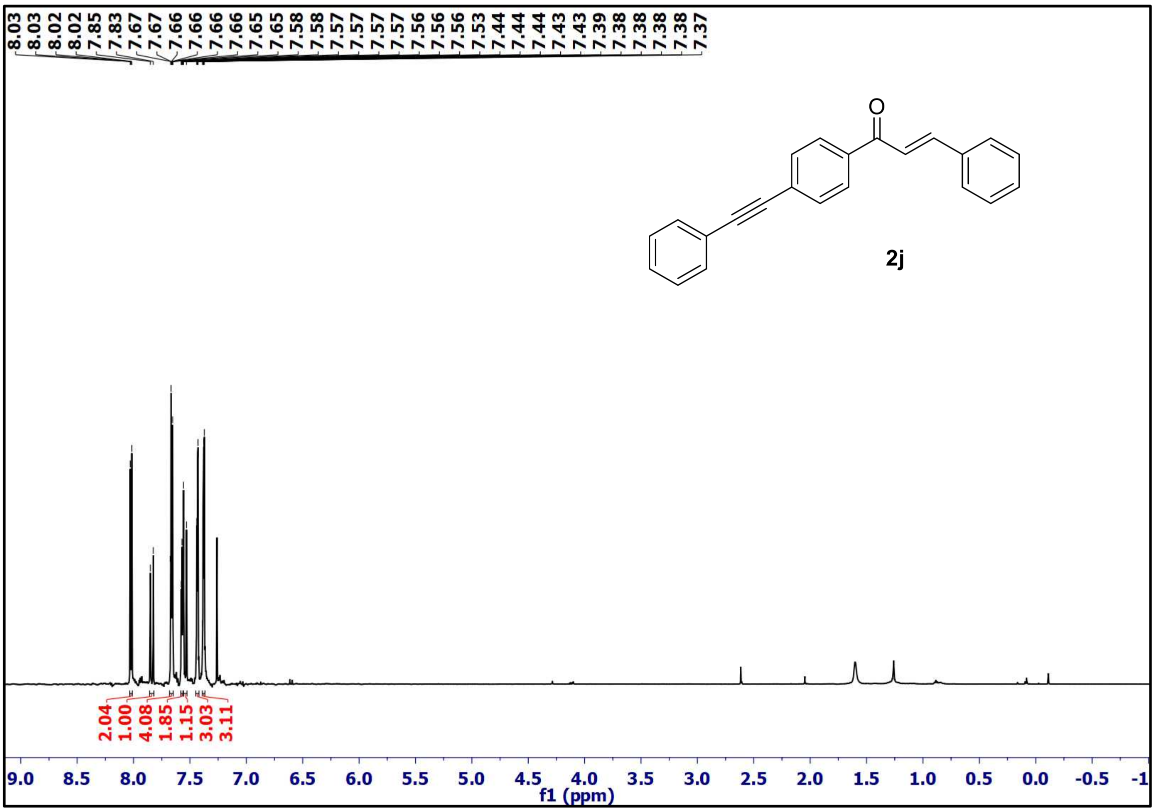

^{13}C NMR (150 MHz, $CDCl_3$)

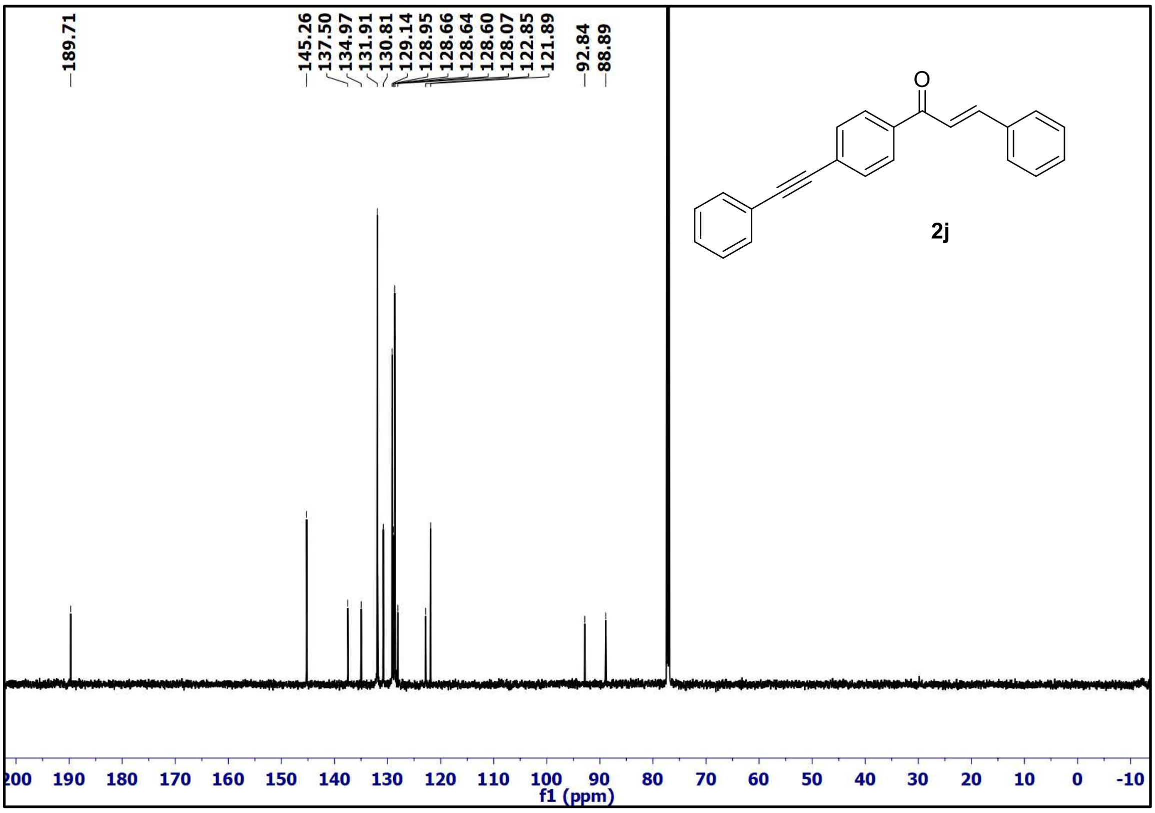

(2*E*,2'*E*)-1,1'-(1,4-Phenylene)bis(3-phenylprop-2-en-1-one) (2k)

^{1}H NMR (600 MHz, $CDCl_3$)

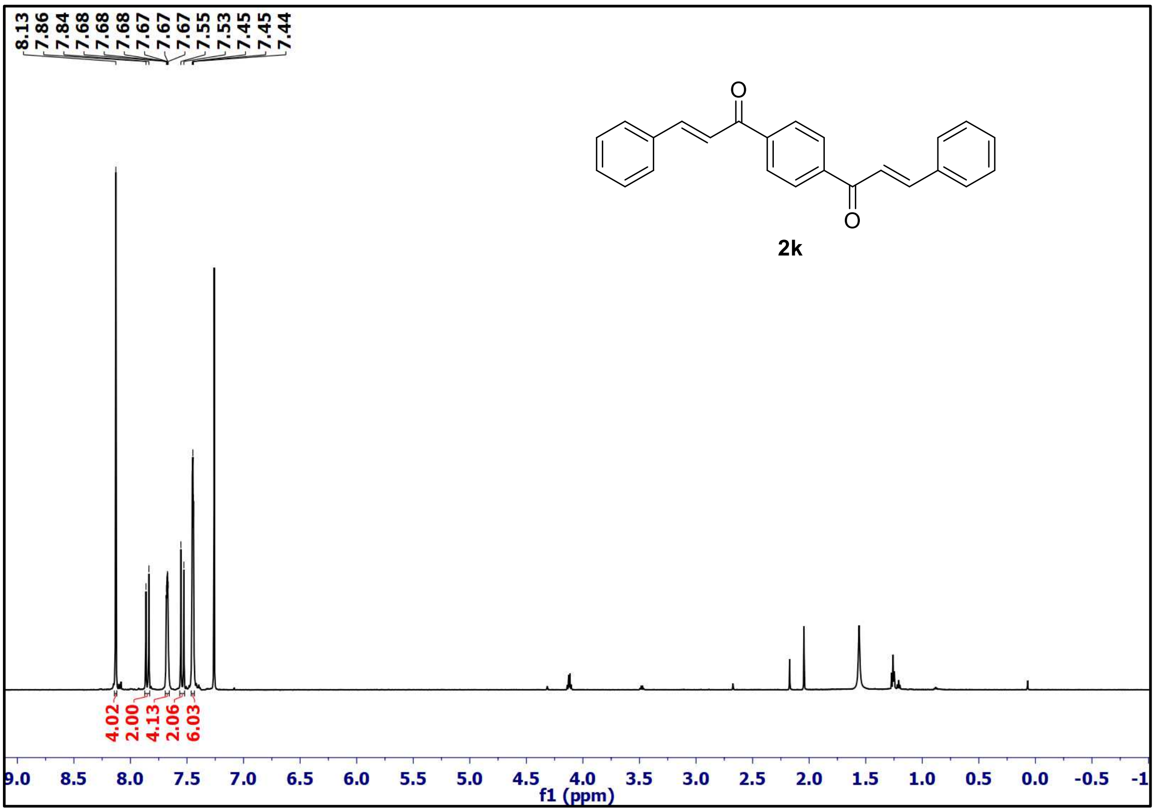

^{13}C NMR (150 MHz, $CDCl_3$)

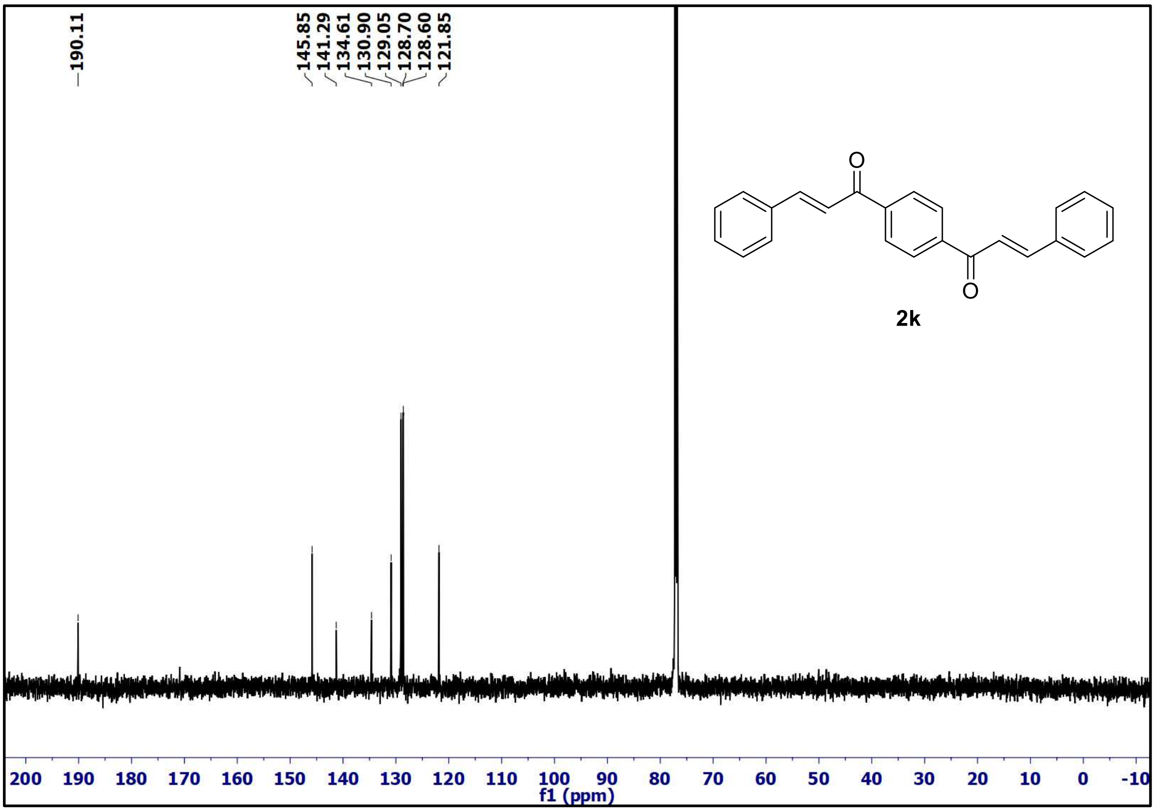

(*E*)-3-Phenyl-1-(thiophen-2-yl)prop-2-en-1-one (2l)

^{1}H NMR (600 MHz, $CDCl_3$)

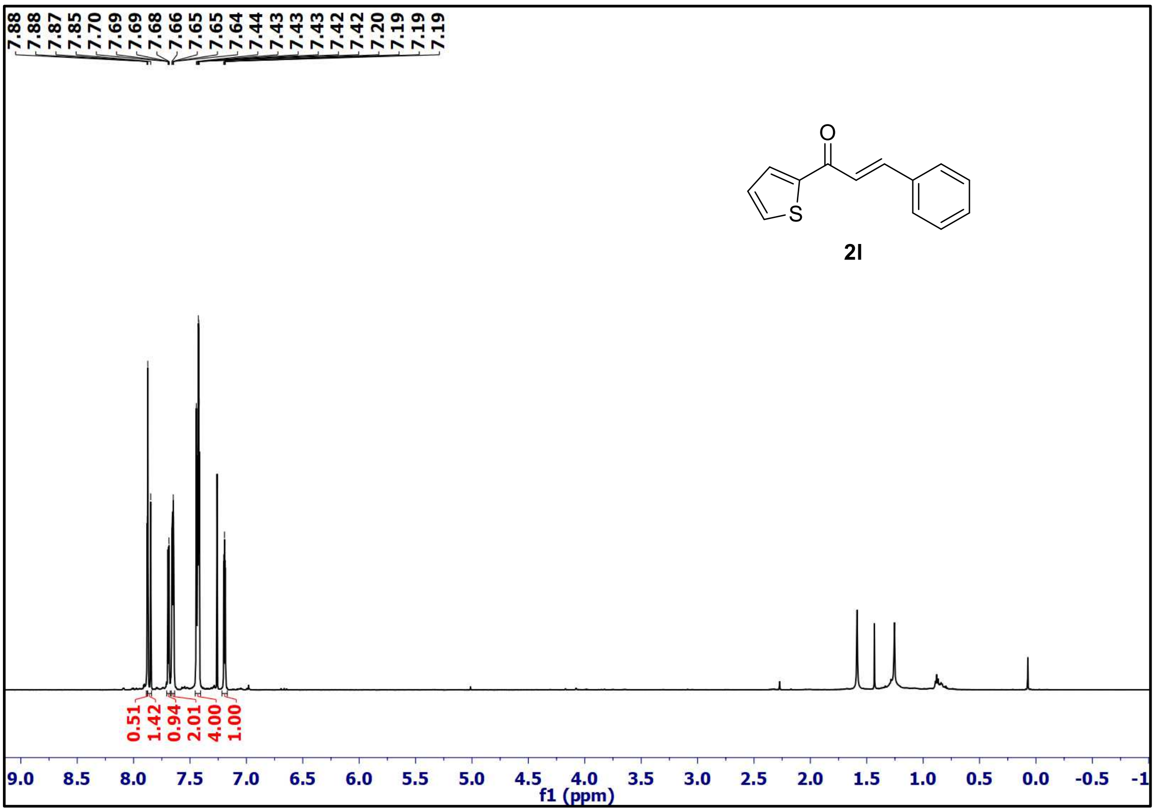

^{13}C NMR (150 MHz, $CDCl_3$)

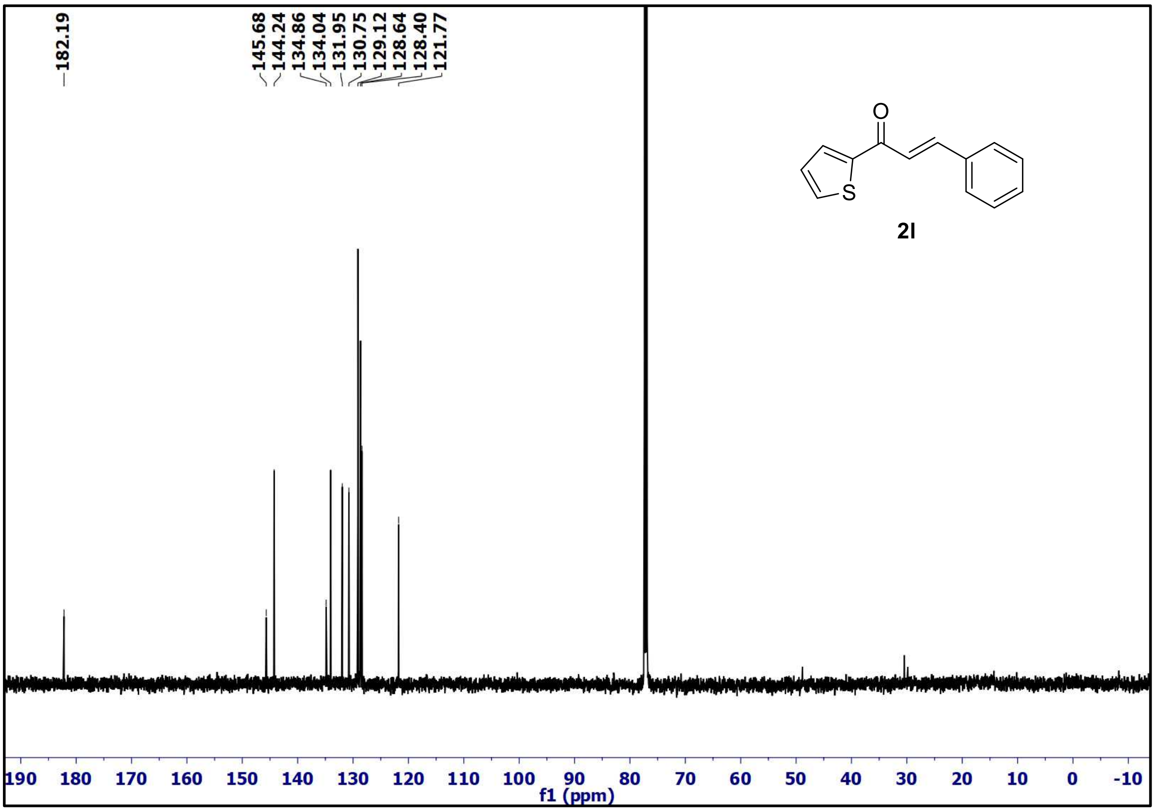

(*E*)-1-(1-Methyl-1*H*-indol-2-yl)-3-phenylprop-2-en-1-one (2m)

^{1}H NMR (600 MHz, $CDCl_3$)

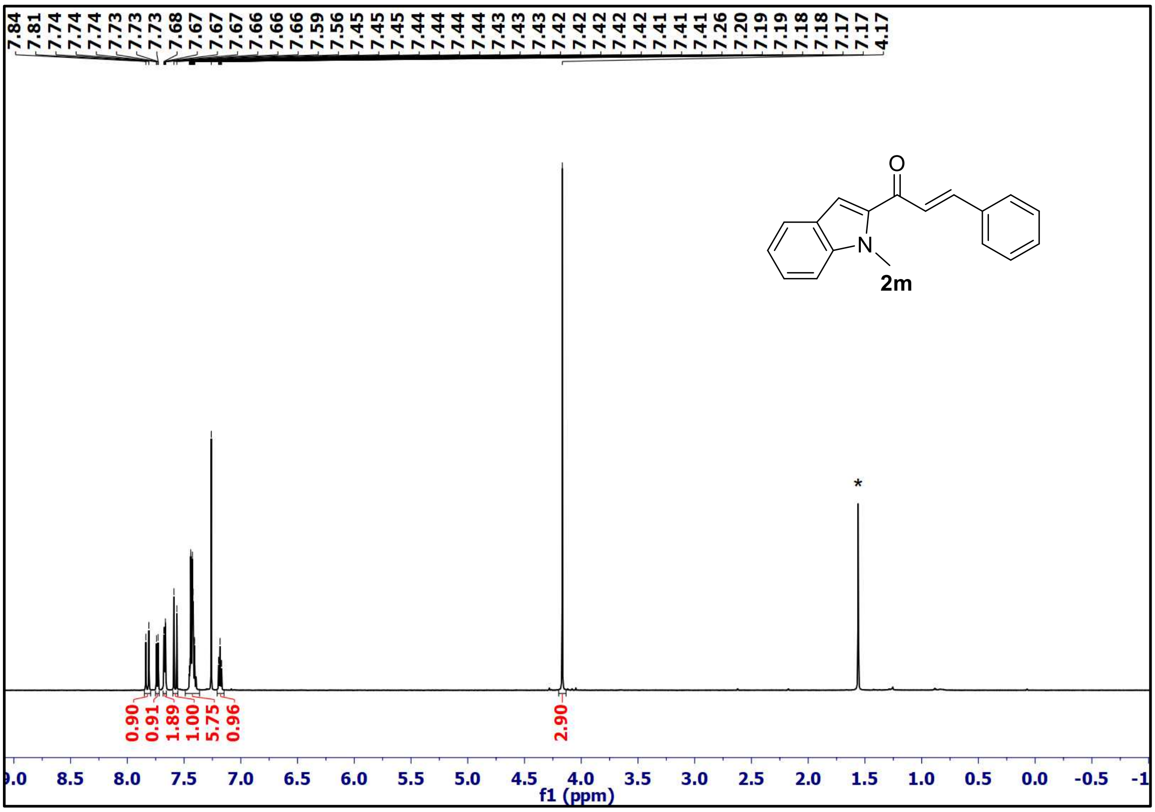

*H_2O residual peak

^{13}C NMR (150 MHz, $CDCl_3$)

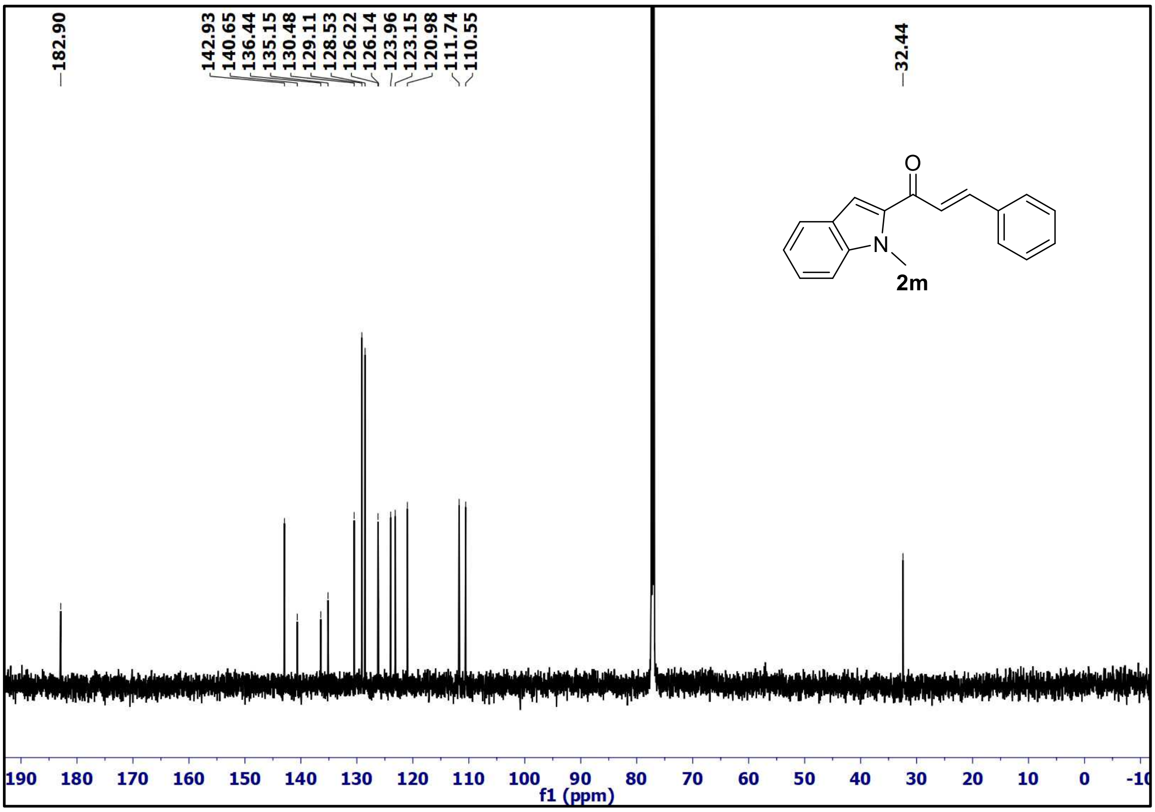

(*E*)-1-Cyclohexyl-3-phenylprop-2-en-1-one (2n)

^{1}H NMR (600 MHz, $CDCl_3$)

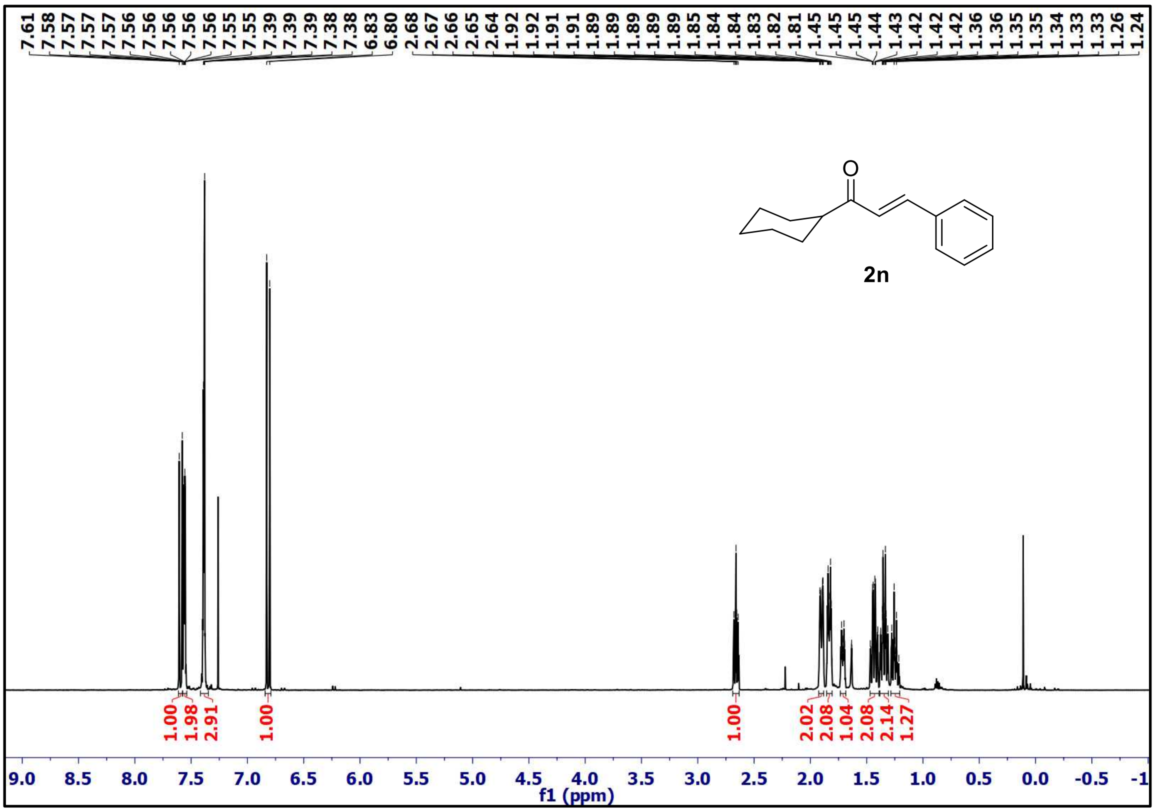

^{13}C NMR (150 MHz, $CDCl_3$)

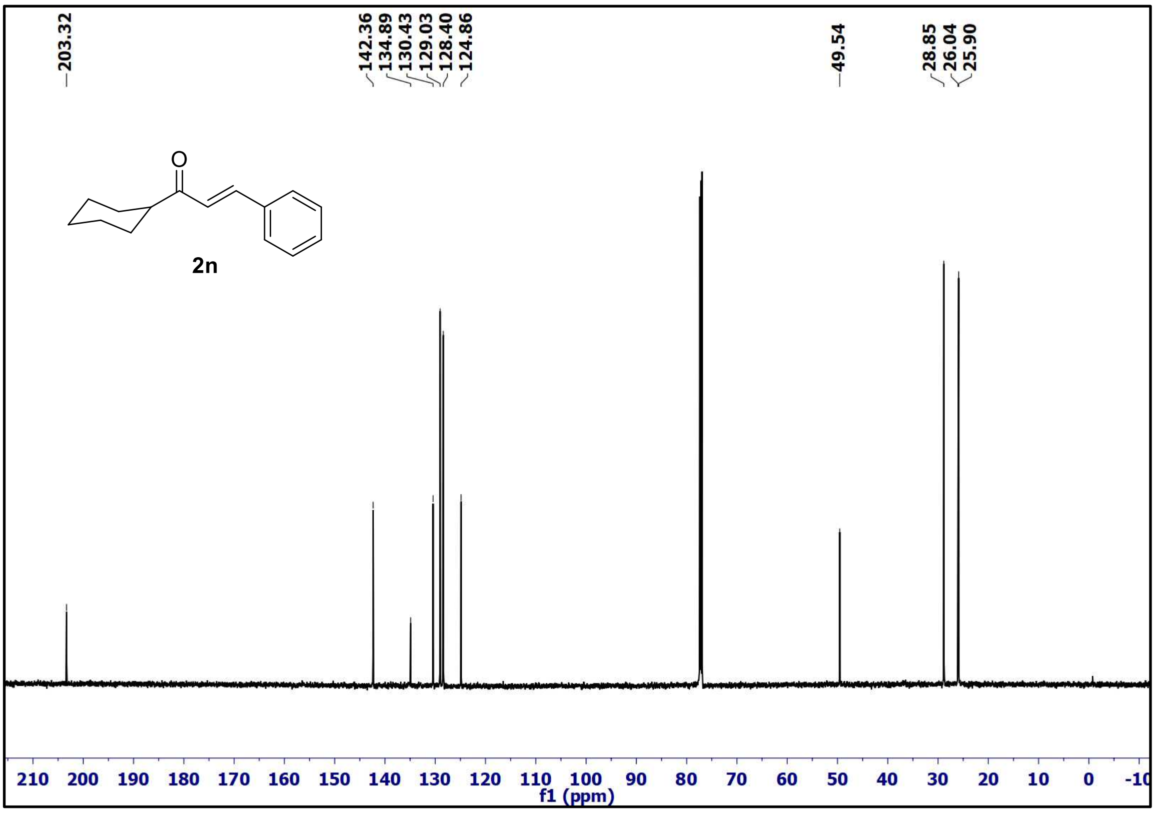

(*E*)-1-(Adamantan-1-yl)-3-phenylprop-2-en-1-one (2o)

^{1}H NMR (600 MHz, $CDCl_3$)

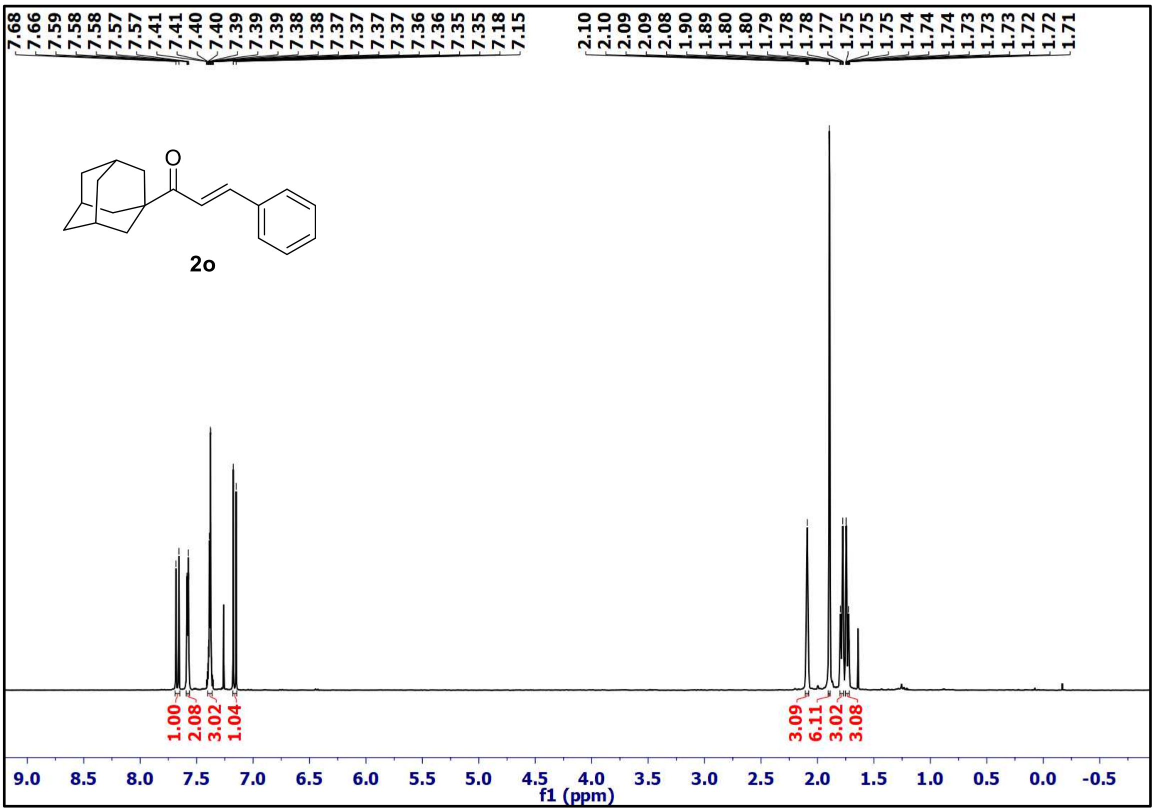

^{13}C NMR (150 MHz, $CDCl_3$)

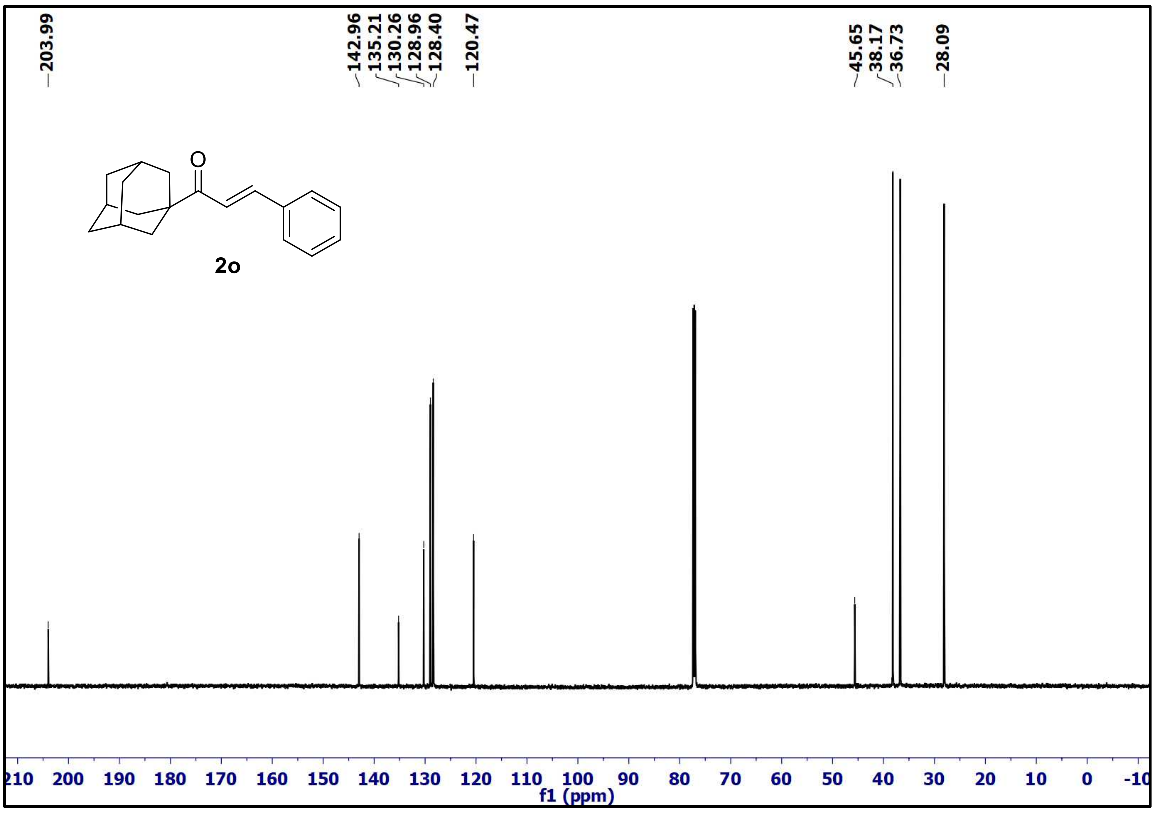

Gram-scale synthesis of (*E*)-chalcone (**2b**)

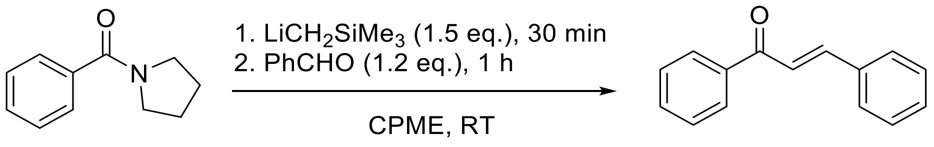

1b **2b**

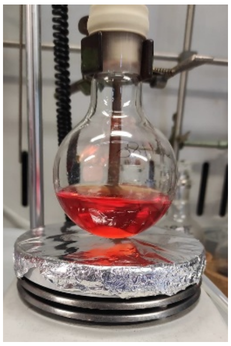

To a stirred solution of *N*-benzoylpyrrolidine **1b** (5.7 mmol, 1.0 eq., 1.0 g) in anhydrous CPME (0.11 M, 50 mL), $LiCH_2SiMe_3$ (0.7 M in hexanes, 8.55 mmol, 1.5 eq., 12.2 mL) was slowly added at room temperature. The resulting mixture was stirred for 30 min, then benzaldehyde (6.84 mmol, 1.2 eq., 0.81 mL) was added. The mixture was stirred for 1 h and then quenched with H_2O. After extraction with EtOAc (3 x 30 mL), the combined organic layers were dried over Na_2SO_4 and evaporated under reduced pressure. Purification by flash column chromatography on silica gel (petroleum ether/DEE 95/5 v/v) gave a **2b** as a yellow solid (816 mg, 69%, R_f= 0.17, petroleum ether/DEE 95/5 v/v). ^{1}H NMR (600 MHz, $CDCl_3$) δ 8.05-8.01 (m, 2H), 7.82 (d, *J* = 15.8 Hz, 1H), 7.67-7.64 (m, 2H), 7.59 (tt, *J* = 7.5, 1.3 Hz, 1H), 7.54 (d, *J* = 15.7 Hz, 1H) superimposed to 7.53-7.49 (m, 2H), 7.44-7.41 (m, 3H). ^{13}C{^{1}H} NMR (150 MHz, $CDCl_3$) δ 190.5, 144.8, 138.2, 134.8, 132.8, 130.5, 128.9, 128.6, 128.5, 128.4, 122.0. EI-MS *m/z* (%): 208 (M^+, 76), 207 (100), 77 (51).

^{1}H and ^{13}C NMR spectra of chalcone **2b** from gram-scale procedure

(*E*)-chalcone (2b)

^{1}H NMR (600 MHz, $CDCl_3$)

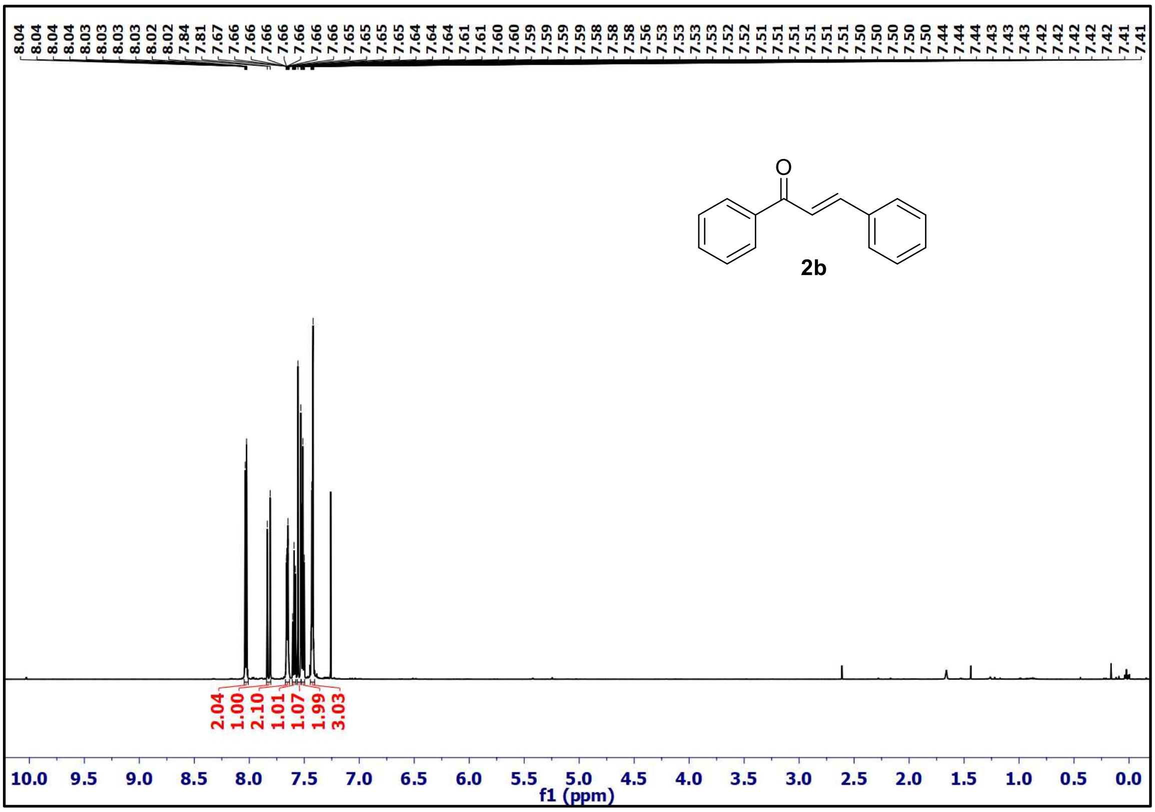

^{13}C NMR (150 MHz, $CDCl_3$)

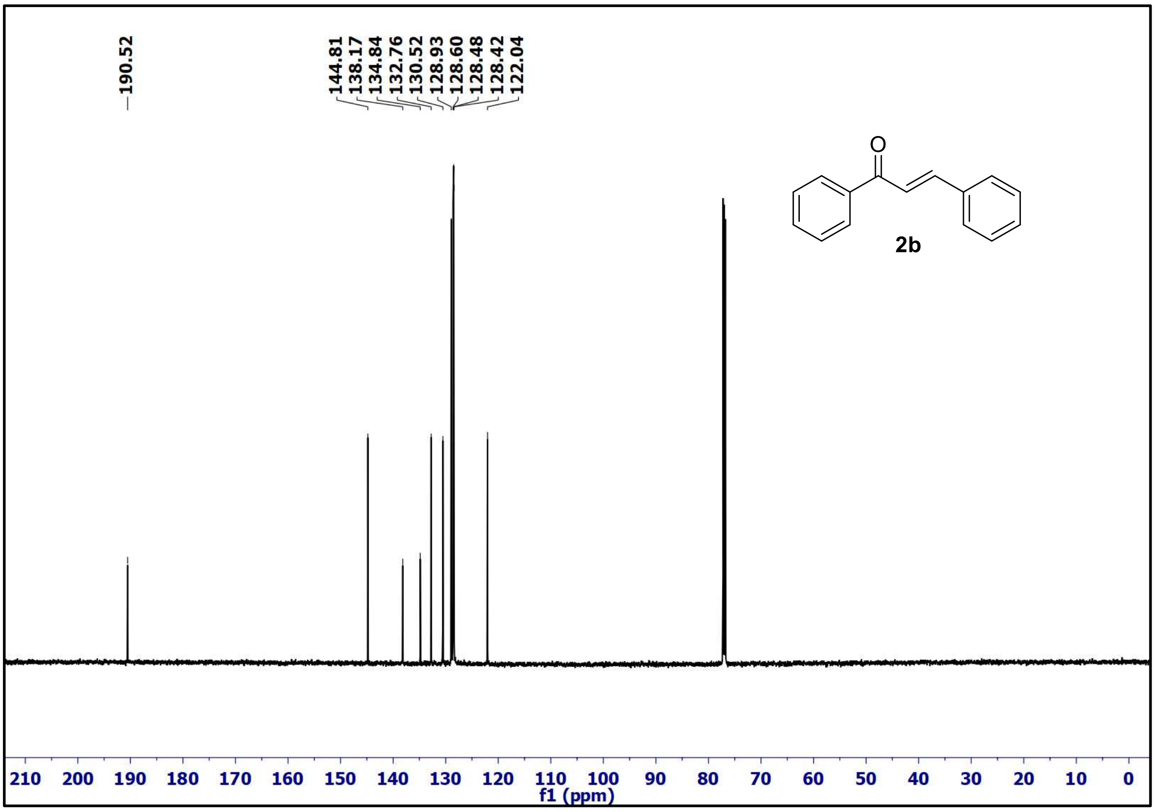

Synthesis and analysis of compounds **2p-2ac**

General procedure. To a stirred solution of (4-methoxyphenyl)(pyrrolidin-1-yl)methanone **1a** (0.2 mmol, 1.0 eq., 41 mg) in anhydrous CPME (0.04 M, 5 mL), $LiCH_2SiMe_3$ (0.7 M in hexanes, 0.3 mmol, 1.5 eq., 0.43 mL) was added at room temperature. The resulting mixture was stirred for 30 min, then the appropriate aldehyde (0.24 mmol, 1.2 eq.) was added. The mixture was stirred for 1 h and then quenched with H_2O. After extraction with EtOAc (3 x 10 mL), the combined organic layers were dried over Na_2SO_4 and evaporated under reduced pressure. The crude products were purified by flash column chromatography on silica gel.

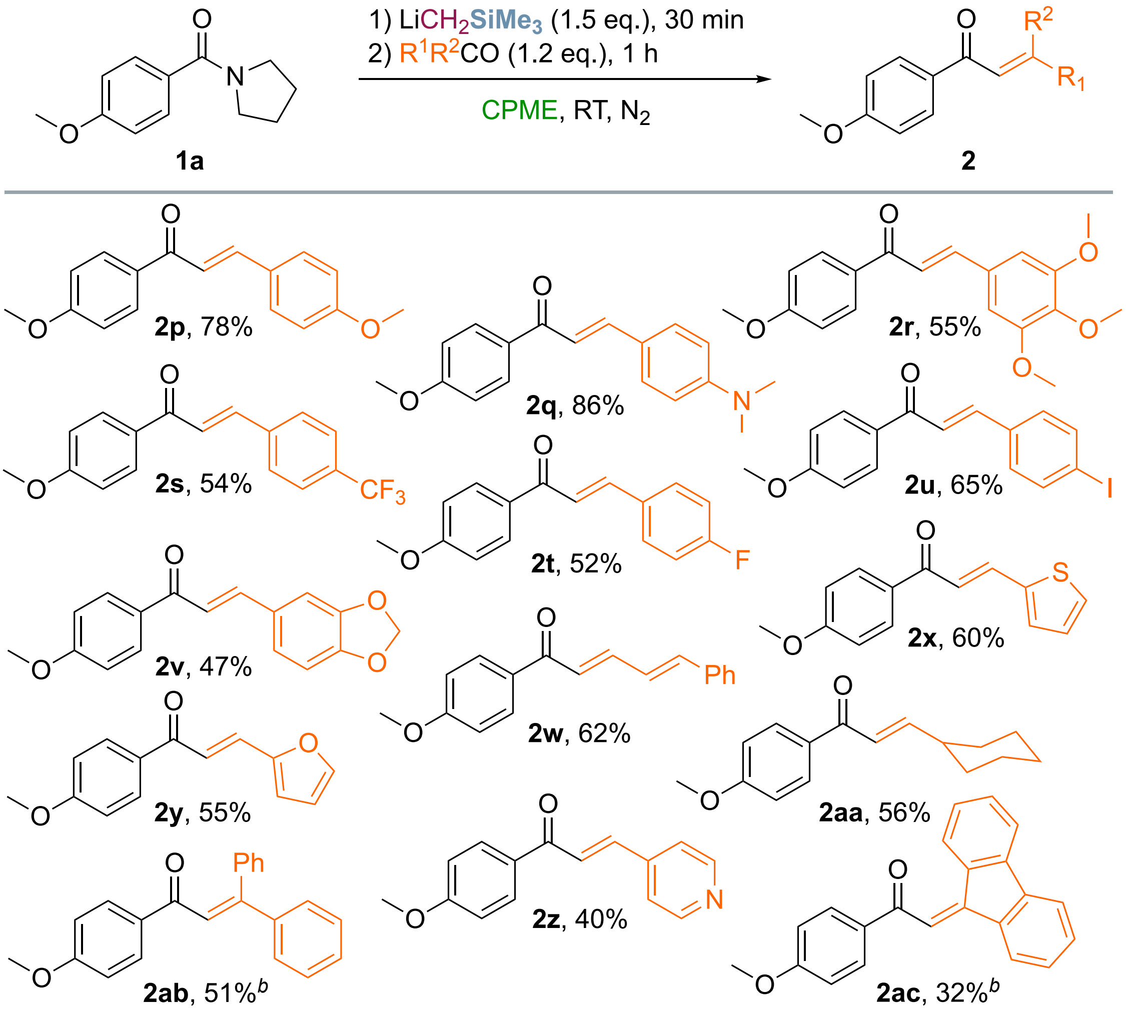

Scheme S5. Synthesis of α,β-unsaturated ketones **2p-2ac**. Reaction conditions: **1a** (0.2 mmol, 1.0 eq.), CPME (5 mL), $LiCH_2SiMe_3$ (0.3 mmol, 1.5 eq.) 30 min, RT, under N_2, then aldehyde (0.24 mmol, 1.2 eq.), 1 hour. [a] Reaction time: 2.5 h after the addition of ketone. Reported yields refer to isolated products after flash column chromatography on silica gel.

(*E*)-1,3-bis(4-Methoxyphenyl)prop-2-en-1-one (2p): General procedure starting from **1a** and 4-methoxybenzaldehyde. Purification by flash column chromatography on silica gel (petroleum ether/EtOAc 85/15 v/v) gave **2p** as a yellow solid (42 mg, 78%, R_f = 0.23 petroleum ether/EtOAc 85/15 v/v), mp. 98.2-100.3 °C.[18] ^{1}H NMR (600 MHz, $CDCl_3$) δ 8.05-8.02 (m, 2H), 7.78 (d, J = 15.6 Hz, 1H), 7.61-7.59 (m, 2H), 7.43 (d, J = 15.6 Hz, 1H), 6.99-6.97 (m, 2H), 6.95-6.92 (m, 2H), 3.89 (s, 3H), 3.85 (s, 3H). ^{13}C{^{1}H} NMR

(150 MHz, $CDCl_3$) δ 188.9, 163.4, 161.6, 144.0, 131.5, 130.8, 130.2, 128.0, 120.0, 114.5, 113.9, 55.6, 55.5. HRMS (ESI) *m*/*z*: $[M+H]^+$ Calcd for $C_{17}H_{17}O_3$ 269.1172; Found 269.1175.

(*E*)-3-(4-(Dimethylamino)phenyl)-1-(4-methoxyphenyl)prop-2-en-1-one (2q): General procedure starting from **1a** and 4-(*N*,*N*-dimethylamino)benzaldehyde. Purification by flash column chromatography on silica gel (petroleum ether/EtOAc 8/2 v/v) gave **2q** as a yellow solid (48 mg, 86%, R_f = 0.27 petroleum ether/EtOAc 8/2 v/v), mp. 123.0-125.6 °C.[19] ^1H NMR (600 MHz, $CDCl_3$) δ 8.04-8.02 (m, 2H), 7.79 (d, *J* = 15.4 Hz, 1H), 7.56-7.54 (m, 2H), 7.36 (d, *J* =15.4 Hz, 1H), 6.98-6.96 (m, 2H), 6.71-6.68 (m, 2H), 3.88 (s, 3H), 3.04 (s, 6H). $^{13}C\{^1H\}$ NMR (150 MHz, $CDCl_3$) δ 189.1, 163.1, 152.0, 145.1, 132.0, 130.7, 130.4, 123.0, 116.8, 113.8, 112.0, 55.6, 40.3. HRMS (ESI) *m*/*z*: $[M+H]^+$ Calcd for $C_{18}H_{20}NO_2$ 282.1493; Found 282.1498.

(*E*)-1-(4-Methoxyphenyl)-3-(3,4,5-trimethoxyphenyl)prop-2-en-1-one (2r): General procedure starting from **1a** and 3,4,5-trimethoxybenzaldehyde. Purification by flash column chromatography on silica gel (petroleum ether/EtOAc 8/2 v/v) gave **2r** as a yellow solid (36 mg, 55%, R_f = 0.20 petroleum ether/EtOAc 8/2 v/v), mp. 121.9-125.4 °C.[20] ^1H NMR (600 MHz, $CDCl_3$) δ 8.04-8.02 (m, 2H), 7.71 (d, *J* = 15.5 Hz, 1H), 7.42 (d, *J* = 15.5 Hz, 1H), 6.99-6.97 (m, 2H), 6.86 (s, 2H), 3.92 (s, 6H), 3.89 (s, 3H), 3.88 (s, 3H). $^{13}C\{^1H\}$ NMR (150 MHz, $CDCl_3$) δ 188.8, 163.5, 153.6, 144.3, 140.4, 131.3, 130.9, 130.7, 121.4, 114.0, 105.7, 61.1, 56.3, 55.6. HRMS (ESI) *m*/*z*: $[M+H]^+$ Calcd for $C_{19}H_{21}O_5$ 329.1384; Found 329.1390.

(*E*)-1-(4-Methoxyphenyl)-3-(4-(trifluoromethyl)phenyl)prop-2-en-1-one (2s): General procedure starting from **1a** and 4-(trifluoromethyl)benzaldehyde. Purification by flash column chromatography on silica gel (petroleum ether/EtOAc 95/5 v/v) followed by trituration with DEE gave **2s** as a white solid (33 mg, 54%, R_f = 0.18 petroleum ether/EtOAc 95/5 v/v), mp. 130.1-133.2 °C.[21] ^1H NMR (600 MHz, $CDCl_3$) δ 8.06-8.04 (m, 2H), 7.80 (d, *J* = 15.7 Hz, 1H), 7.74 (d, *J* = 8.1 Hz, 2H), 7.67 (d, *J* = 8.2 Hz, 2H), 7.61 (d, *J* = 15.7 Hz, 1H), 7.01-6.99 (m, 2H), 3.90 (s, 3H). $^{13}C\{^1H\}$ NMR (150 MHz, $CDCl_3$) δ 188.3, 163.9, 142.0, 138.6, 131.8 (q, *J* = 33.1 Hz, 1C), 131.1, 130.9, 128.6, 126.0 (q, *J* = 4.3 Hz, 2C), 124.3, 124.0 (q, *J* = 271.8 Hz, 1C), 114.1, 55.7. ^{19}F NMR (564 MHz, $CDCl_3$) δ -62.70 (s, 3F). EI-MS *m/z* (%): 306 (M^+, 100), 278 (39), 151 (31), 135 (93). HRMS (ESI) *m*/*z*: $[M+H]^+$ Calcd for $C_{17}H_{14}F_3O_2$ 307.0940; Found 307.0948.

(*E*)-3-(4-Fluorophenyl)-1-(4-methoxyphenyl)prop-2-en-1-one (2t): General procedure starting from **1a** and 4-fluorobenzaldehyde. Purification by flash column chromatography on silica gel (petroleum ether/EtOAc 9/1 v/v) followed by trituration with DEE gave **2t** as a white solid (26 mg, 52%, R_f = 0.22 petroleum ether/EtOAc 9/1 v/v), mp. 115.4-117.0 °C.[18] ^1H NMR (600 MHz, $CDCl_3$) δ 8.05-8.02 (m, 2H), 7.76 (d, *J* =15.6 Hz, 1H), 7.65-7.61 (m, 2H), 7.47 (d, *J*=15.5 Hz, 1H), 7.12-7.09 (m, 2H), 7.00-6.67 (m, 2H), 3.89 (s, 3H). $^{13}C\{^1H\}$ NMR (150 MHz, $CDCl_3$) δ 188.6, 164.1 (d, *J* = 251.5 Hz, 1C), 163.6, 142.8, 131.5 (d, *J* = 3.3 Hz, 1C), 131.1, 130.9, 130.4 (d, *J* = 8.3 Hz, 2C), 121.7, 116.2 (d, *J* = 21.8 Hz, 2C), 114.0, 55.6. ^{19}F NMR (564 MHz, $CDCl_3$) δ -

109.35 (s, 1F). EI-MS *m/z* (%): 256 (M^+, 100), 255 (61), 135 (81). HRMS (ESI) *m/z*: $[M+H]^+$ Calcd for $C_{16}H_{14}FO_2$ 257.0972; Found 257.0979.

(*E*)-3-(4-Iodophenyl)-1-(4-methoxyphenyl)prop-2-en-1-one (2u): General procedure starting from **1a** and 4-iodobenzaldehyde. Purification by flash column chromatography on silica gel (petroleum ether/EtOAc 9/1 v/v) followed by trituration with DEE gave **2u** as a white solid (47 mg, 65%, R_f = 0.22 petroleum ether/EtOAc 9/1 v/v), mp. 179.0-181.2 °C.[18] ^{1}H NMR (600 MHz, $CDCl_3$) δ 8.05-8.02 (m, 2H), 7.77-7.75 (m, 2H), 7.71 (d, *J* =15.6 Hz, 1H), 7.54 (d, *J* =15.6 Hz, 1H), 7.38-7.35 (m, 2H), 7.00-6.98 (m, 2H), 3.90 (s, 3H). $^{13}C\{^1H\}$ NMR (150 MHz, $CDCl_3$) δ 188.6, 163.7, 142.8, 138.3, 134.7, 131.1, 131.0, 129.9, 122.6, 114.1, 96.7, 55.7. EI-MS *m/z* (%): 364 (M^+, 100), 363 (48), 237 (40), 135 (68). HRMS (ESI) *m/z*: $[M+H]^+$ Calcd for $C_{16}H_{14}IO$ 365.0033; Found 365.0029.

(*E*)-3-(Benzo[d][1,3]dioxol-5-yl)-1-(4-methoxyphenyl)prop-2-en-1-one (2v): General procedure starting from **1a** and piperonal. Purification by flash column chromatography on silica gel (petroleum ether/EtOAc 8/2 v/v) gave **2v** as a yellow solid (27 mg, 47%, R_f = 0.32 petroleum ether/EtOAc 8/2 v/v), mp. 126.2-129.4 °C.[22] ^{1}H NMR (600 MHz, $CDCl_3$) δ 8.04-8.01 (m, 2H), 7.73 (d, *J* = 15.5 Hz, 1H), 7.78 (d, *J* = 15.5 Hz, 1H), 7.17 (d, *J* = 1.7 Hz, 1H), 7.12 (dd, *J* = 8.0, 1.7 Hz, 1H), 6.99-6.97 (m, 2H), 6.84 (d, *J* = 8.0 Hz, 1H), 6.02 (s, 2H), 3.89 (s, 3H). $^{13}C\{^1H\}$ NMR (150 MHz, $CDCl_3$) δ 188.8, 163.5, 149.9, 148.5, 144.0, 131.4, 130.9, 129.7, 125.2, 120.0, 114.0, 108.8, 106.8, 101.7, 55.6. HRMS (ESI) *m/z*: $[M+H]^+$ Calcd for $C_{17}H_{15}O_4$ 283.0965; Found 283.0970.

(2*E*,4*E*)-1-(4-Methoxyphenyl)-5-phenylpenta-2,4-dien-1-one (2w): General procedure starting from **1a** and *trans*-cinnamaldehyde. Purification by flash column chromatography on silica gel (petroleum ether/EtOAc 9/1 v/v) gave **2w** as a yellow solid (33 mg, 62%, R_f = 0.30 petroleum ether/EtOAc 9/1 v/v), mp. 77.1-80.4 °C.[23] ^{1}H NMR (600 MHz, $CDCl_3$) δ 8.01-7.98 (m, 2H), 7.60 (ddd, *J* = 14.9, 9.1, 1.2 Hz, 1H), 7.51-7.49 (m, 2H), 7.39-7.36 (m, 2H), 7.30 (tt, *J* = 7.2, 1.3 Hz, 1H), 7.11 (d, *J* = 14.9 Hz, 1H), 7.06-7.00 (m, 2H), 6.98-6.95 (m, 2H), 3.88 (s, 3H). $^{13}C\{^1H\}$ NMR (150 MHz, $CDCl_3$) δ 188.9, 163.5, 144.1, 141.5, 136.4, 131.3, 130.8, 129.2, 129.0, 127.4, 127.2, 125.4, 114.0, 55.6. HRMS (ESI) *m/z*: $[M+H]^+$ Calcd for $C_{18}H_{17}O_2$ 265.1223; Found 265.1220.

(*E*)-1-(4-Methoxyphenyl)-3-(thiophen-2-yl)prop-2-en-1-one (2x): General procedure starting from **1a** and thiophene-2-carbaldehyde. Purification by flash column chromatography on silica gel (petroleum ether/EtOAc 9/1 v/v) followed by trituration with DEE gave **2x** as a yellow solid (29 mg, 60%, R_f = 0.28 petroleum ether/EtOAc 9/1 v/v), mp. 104.3-105.1 °C.[24] ^{1}H NMR (600 MHz, $CDCl_3$) δ 8.04-8.01 (m, 2H), 7.93 (d, *J* = 15.3 Hz, 1H), 7.40 (d, *J* = 5.0 Hz, 1H), 7.34 (d, *J* = 3.4 Hz, 1H) superimposed to 7.34 (d, *J* = 15.3 Hz, 1H), 7.09 (dd, *J* = 5.0, 3.6 Hz, 1H), 6.99-6.97 (m, 2H), 3.89 (s, 3H).$^{13}C\{^1H\}$ NMR (150 MHz, $CDCl_3$) δ 188.2,

163.6, 140.7, 136.6, 131.9, 131.2, 130.9, 128.6, 128.4, 120.8, 114.0, 55.6. EI-MS *m/z* (%): 244 (M^+, 100), 135 (85), 160 (77). HRMS (ESI) *m/z*: $[M+H]^+$ Calcd for $C_{14}H_{13}O_2S$ 245.0631; Found 245.0634.

(*E*)-3-(Furan-2-yl)-1-(4-methoxyphenyl)prop-2-en-1-one (2y): General procedure starting from **1a** and furfural. Purification by flash column chromatography on silica gel (petroleum ether/EtOAc 95/5 v/v) gave **2y** as a yellow solid (25 mg, 55%, R_f = 0.15 petroleum ether/EtOAc 95/5 v/v), mp. 69.4-72.1 °C.[21] ^{1}H NMR (600 MHz, $CDCl_3$) δ 8.06-8.03 (m, 2H), 7.58 (d, *J* = 15.3 Hz, 1H), 7.52 (d, *J* = 1.8 Hz, 1H), 7.47 (d, *J* = 15.3 Hz, 1H), 6.99-6.96 (m, 2H), 6.70 (d, *J* = 3.4 Hz, 1H), 6.51 (dd, *J* = 3.4, 1.8 Hz, 1H), 3.88 (s, 3H). $^{13}C\{^1H\}$ NMR (150 MHz, $CDCl_3$) δ 188.3, 163.6, 152.0, 144.8, 131.2, 131.0, 130.1, 119.4, 115.9, 114.0, 112.7, 55.6. EI-MS *m/z* (%): 228 (M^+, 100), 174 (49), 135 (87). HRMS (ESI) *m/z*: $[M+H]^+$ Calcd for $C_{14}H_{13}O$ 229.0859; Found 229.0861.

(*E*)-1-(4-methoxyphenyl)-3-(pyridin-4-yl)prop-2-en-1-one (2z): General procedure starting from **1a** and isonicotinaldehyde. Purification by flash column chromatography on silica gel (petroleum ether/EtOAc 1/1 v/v) followed by trituration with DEE gave **2z** as a yellow solid (19 mg, 40%, R_f = 0.13 petroleum ether/EtOAc 1/1 v/v), mp. 65.0-66.4 °C.[24] ^{1}H NMR (600 MHz, $CDCl_3$) δ 8.68 (br s, 2H), 8.04 (d, *J* = 8.4 Hz, 2H), 7.68 (s, 2H), 7.47 (d, *J* = 5.7 Hz, 2H), 7.00 (d, *J* = 8.4 Hz, 2H), 3.90 (s, 3H). $^{13}C\{^1H\}$ NMR (150 MHz, $CDCl_3$) δ 188.1, 164.0, 150.7, 142.5, 140.8, 131.2, 130.6, 126.2, 122.2, 114.2, 55.7. EI-MS *m/z* (%): 239 (M^+, 97), 135 (100), 211 (80). HRMS (ESI) *m/z*: $[M+H]^+$ Calcd for $C_{15}H_{14}NO_2$ 240.1019; Found 240.1019.

(*E*)-3-Cyclohexyl-1-(4-methoxyphenyl)prop-2-en-1-one (2aa): General procedure starting from **1a** and cyclohexanecarbaldehyde. Purification by flash column chromatography on silica gel (petroleum ether/EtOAc 9/1 v/v) gave **2aa** as a yellow solid (27 mg, 56%, R_f = 0.30 petroleum ether/EtOAc 9/1 v/v), mp. 57.8-60.8 °C.[21] ^{1}H NMR (600 MHz, $CDCl_3$) δ 7.95-7.93 (m, 2H), 6.99 (dd, *J* = 15.5, 6.8 Hz, 1H), 6.95-6.93 (m, 2H), 6.83 (dd, *J* = 15.5, 1.4 Hz, 1H), 3.87 (s, 3H), 2.27-2.21 (m, 1H), 1.85-1.76 (m, 4H), 1.71-1.67 (m, 1H), 1.38-1.28 (m, 2H), 1.25-1.18 (m, 3H). $^{13}C\{^1H\}$ NMR (150 MHz, $CDCl_3$) δ 189.7, 163.4, 154.0, 131.2, 131.0, 123.2, 113.8, 55.6, 41.1, 32.1, 26.1, 25.9. EI-MS *m/z* (%): 244 (M^+, 23), 135 (100), 150 (80). HRMS (ESI) *m/z*: $[M+H]^+$ Calcd for $C_{16}H_{21}O_2$ 245.1536; Found 245.1540.

1-(4-Methoxyphenyl)-3,3-diphenylprop-2-en-1-one (2ab): General procedure starting from **1a** and benzophenone. Reaction time after addition of benzophenone: 2.5 h. Purification by flash column chromatography on silica gel (petroleum ether/EtOAc 85/15 v/v) gave **2ab** as a white solid (32 mg, 51%, R_f = 0.15 petroleum ether/EtOAc 85/15 v/v), mp. 95.7-96.6 °C.[25] ^{1}H NMR (600 MHz, $CDCl_3$) δ 7.94-7.90 (m, 2H), 7.41-7.34 (m, 5H), 7.29-7.23 (m, 3H), 7.20-7.16 (m, 2H), 7.08 (s, 1H), 6.89-6.84 (m, 2H), 3.84 (s, 3H). $^{13}C\{^1H\}$ NMR (150 MHz, $CDCl_3$) δ 191.4, 163.2, 153.6, 141.5, 139.1, 131.2, 131.1, 129.7, 129.1, 128.5, 128.4, 128.2, 128.0, 124.3, 113.6, 55.4. HRMS (ESI) *m/z*: $[M+H]^+$ Calcd for $C_{22}H_{19}O_2$ 315.1380; Found 315.1385.

2-(9*H*-Fluoren-9-ylidene)-1-(4-methoxyphenyl)ethan-1-one (2ac): General procedure starting from **1a** and 9-fluorenone. Reaction time after addition of 9-fluorenone: 2.5 h. Purification by flash column chromatography on silica gel (petroleum ether/EtOAc 9/1 v/v) gave **2ac** as a yellow solid (29 mg, 32%, R_f = 0.25 petroleum ether/EtOAc 9/1 v/v), mp. 104.7-106.1 °C.[27] ^{1}H NMR (600 MHz, $CDCl_3$) δ 8.18 (dt, *J* = 7.8, 0.9 Hz, 1H), 8.12-8.09 (m, 2H), 7.78 (dt, *J* = 7.7, 0.9 Hz, 1H), 7.65 (tt, *J* = 7.5, 0.9 Hz, 2H), 7.56 (s, 1H), 7.42 (td, *J* = 7.5, 1.0 Hz, 1H), 7.36 (td, *J* = 7.4, 1.0 Hz, 1H), 7.32 (td, *J* = 7.5, 1.1 Hz, 1H), 7.19 (td, *J* = 7.6, 1.1 Hz, 1H), 7.00-6.98 (m, 2H), 3.90 (s, 3H). $^{13}C\{^1H\}$ NMR (150 MHz, $CDCl_3$) δ 191.6, 163.9, 144.7, 142.1, 140.7, 138.7, 135.5, 131.3, 131.1, 130.4, 130.1, 127.8, 127.4, 127.3, 121.0, 120.1, 119.9, 119.7, 114.0, 55.5. HRMS (ESI) *m*/*z*: $[M+Na]^+$ Calcd for $C_{22}H_{16}O_2Na$ 335.1043; Found 335.1049.

^{1}H and ^{13}C NMR spectra of compounds **2p-2ac**

(*E*)-1,3-bis(4-Methoxyphenyl)prop-2-en-1-one (2p)

^{1}H NMR (600 MHz, $CDCl_3$)

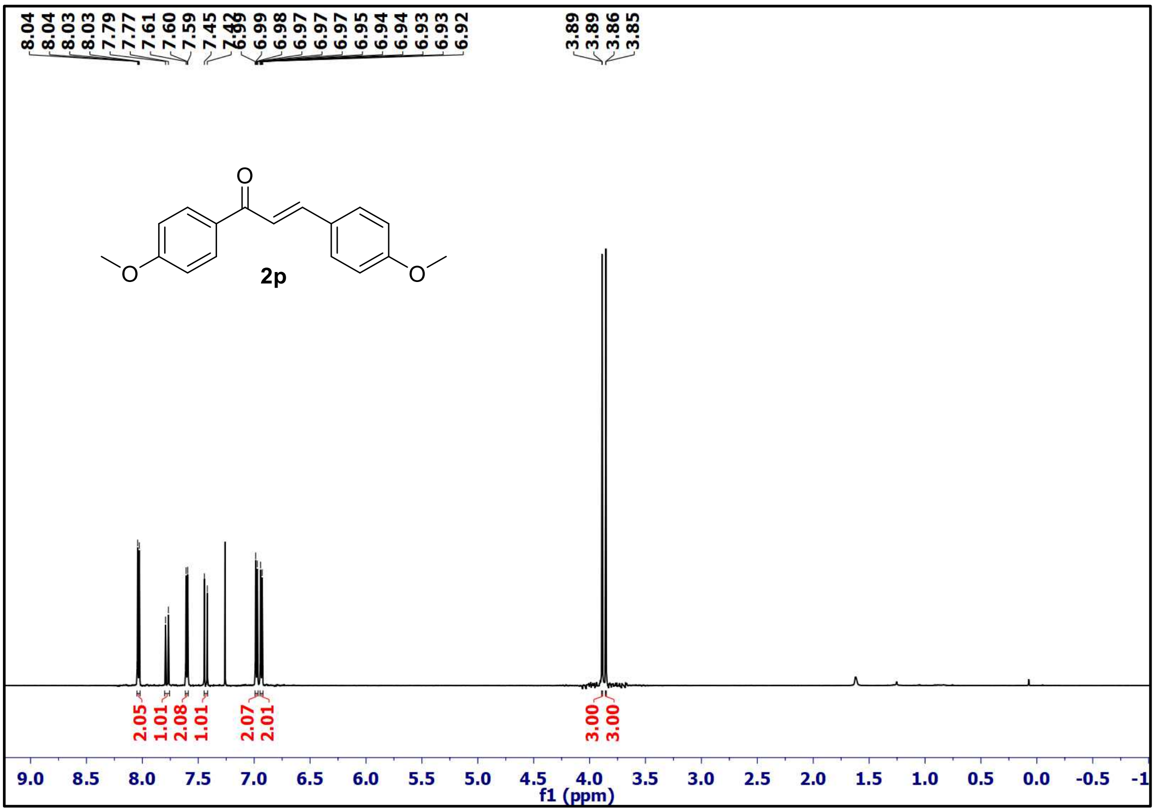

^{13}C NMR (150 MHz, $CDCl_3$)

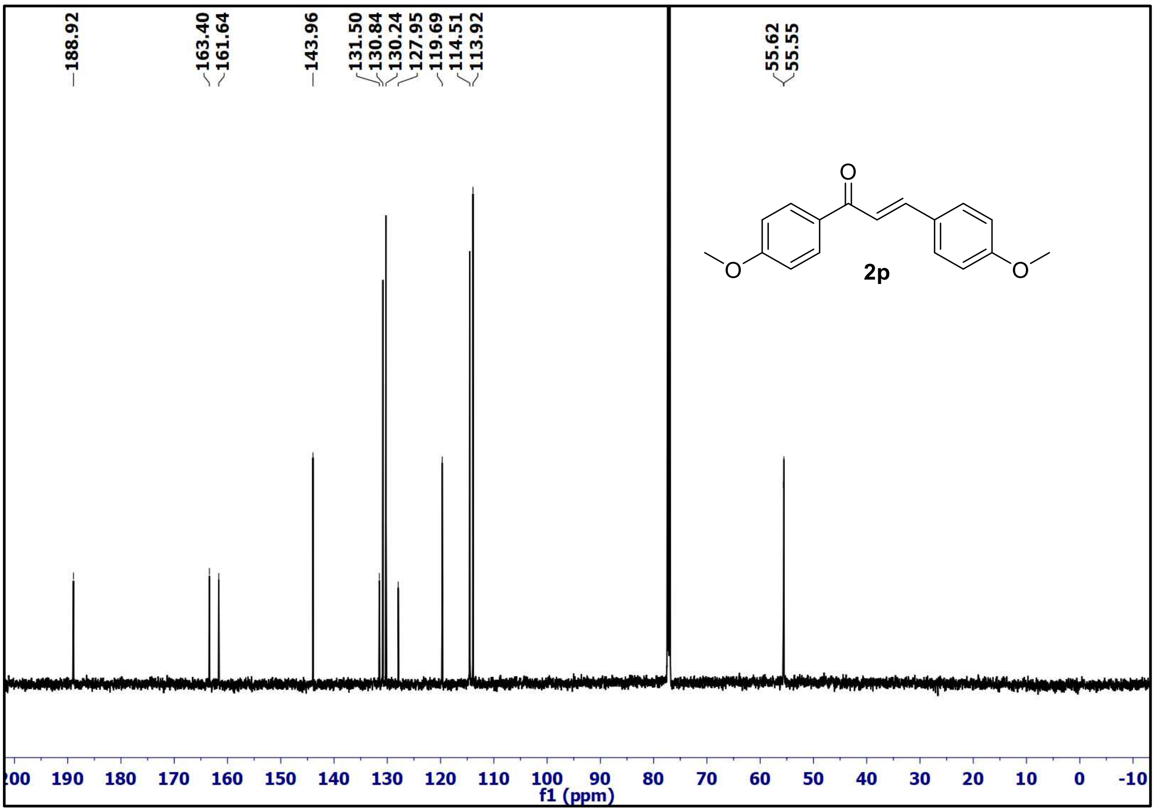

(*E*)-3-(4-(*N*,*N*-Dimethylamino)phenyl)-1-(4-methoxyphenyl)prop-2-en-1-one (2q)

^{1}H NMR (600 MHz, $CDCl_3$)

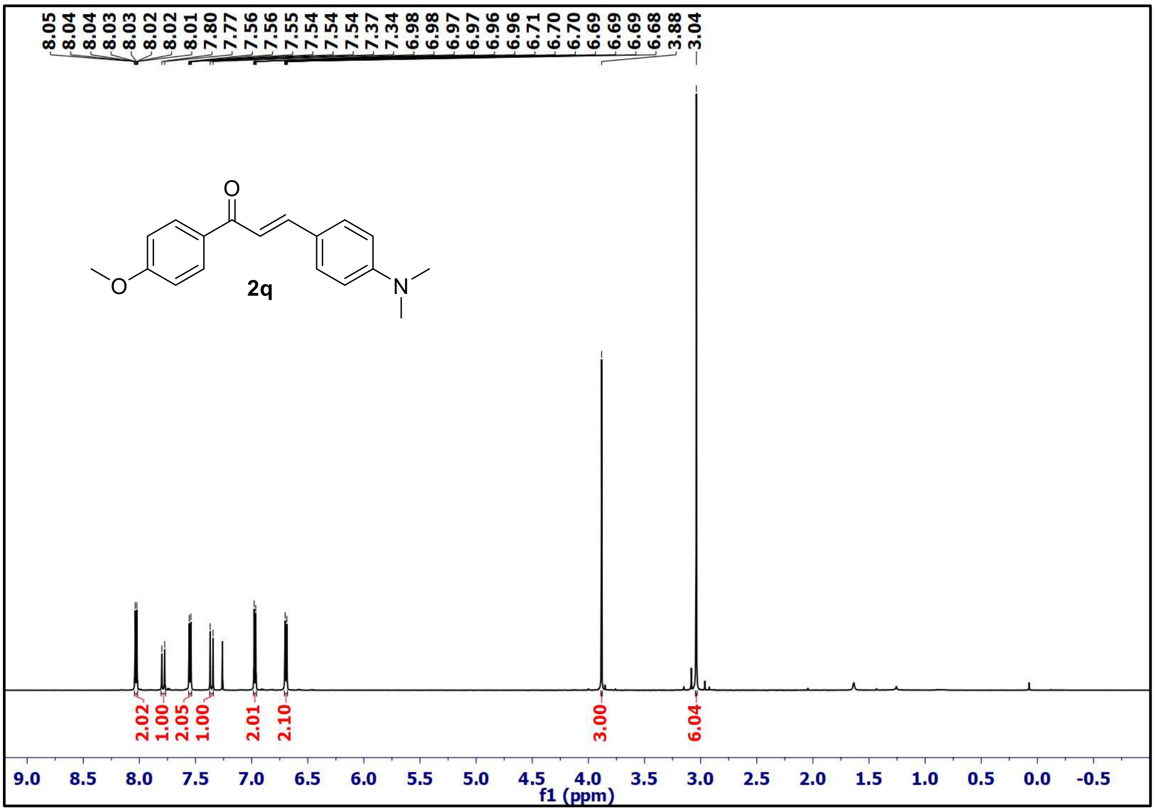

^{13}C NMR (150 MHz, $CDCl_3$)

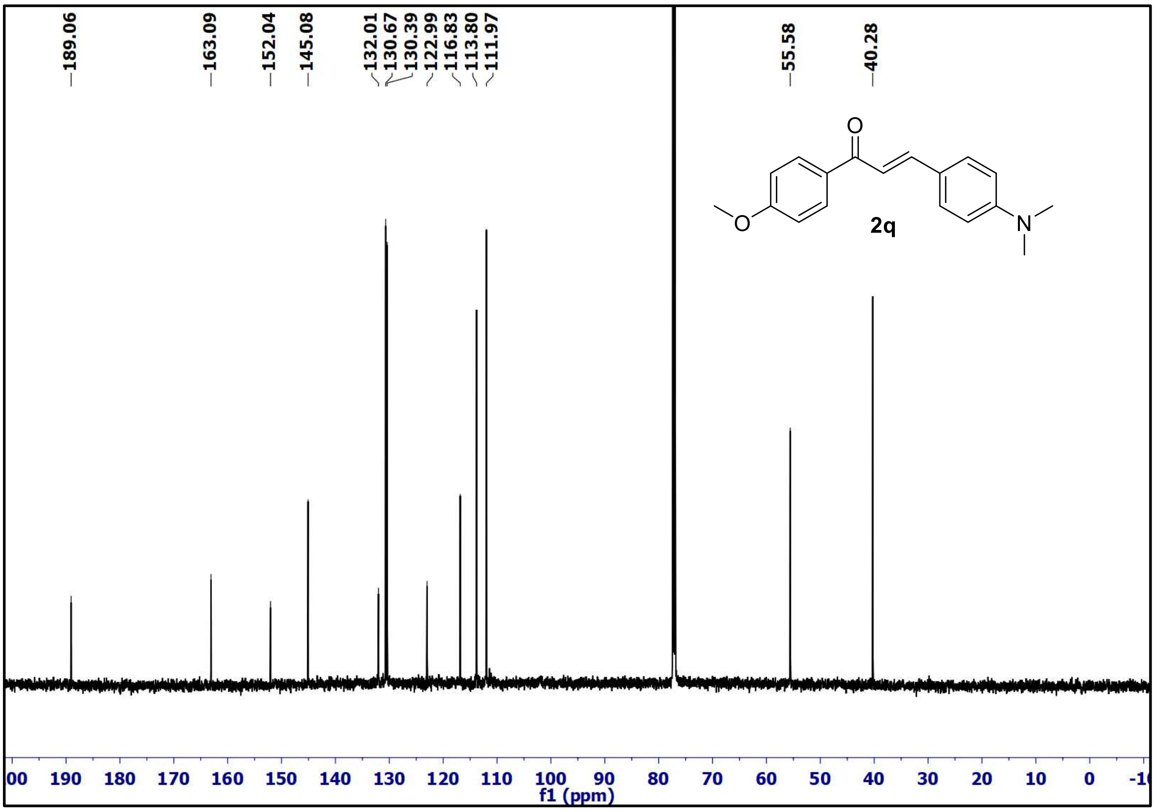

(*E*)-1-(4-Methoxyphenyl)-3-(3,4,5-trimethoxyphenyl)prop-2-en-1-one (2r)

^{1}H NMR (600 MHz, $CDCl_3$)

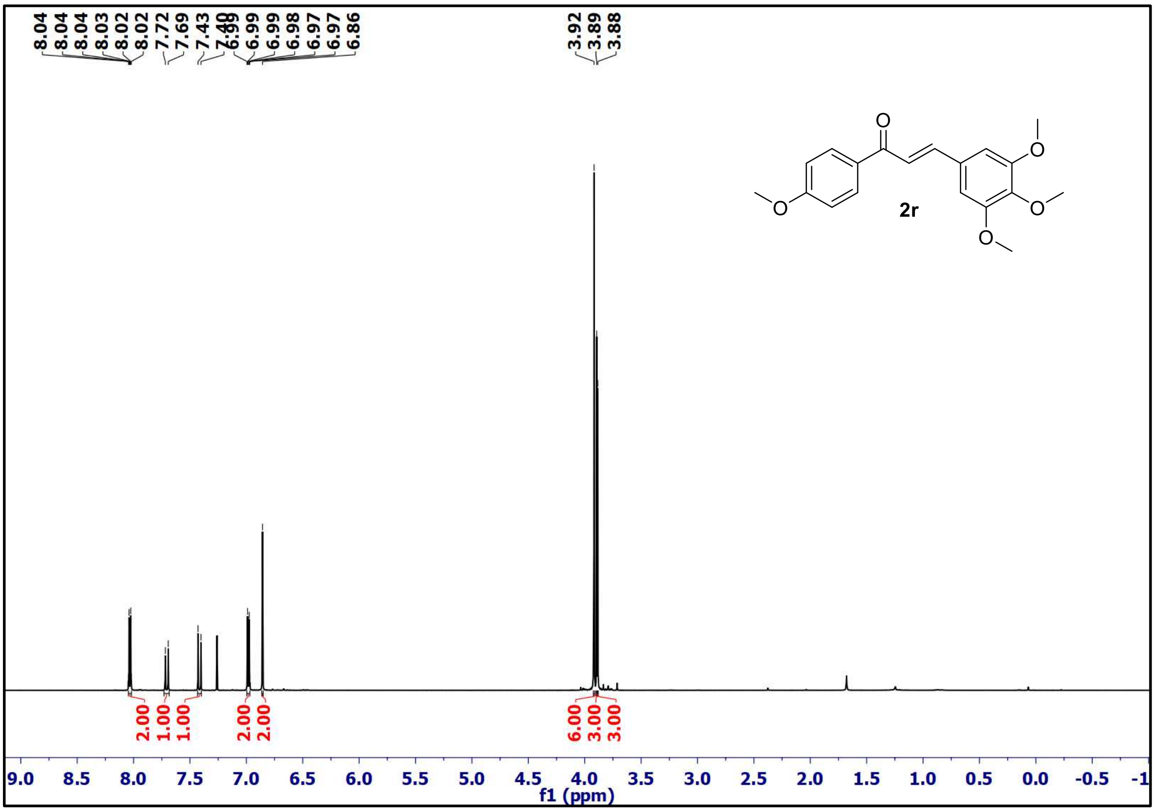

^{13}C NMR (150 MHz, $CDCl_3$)

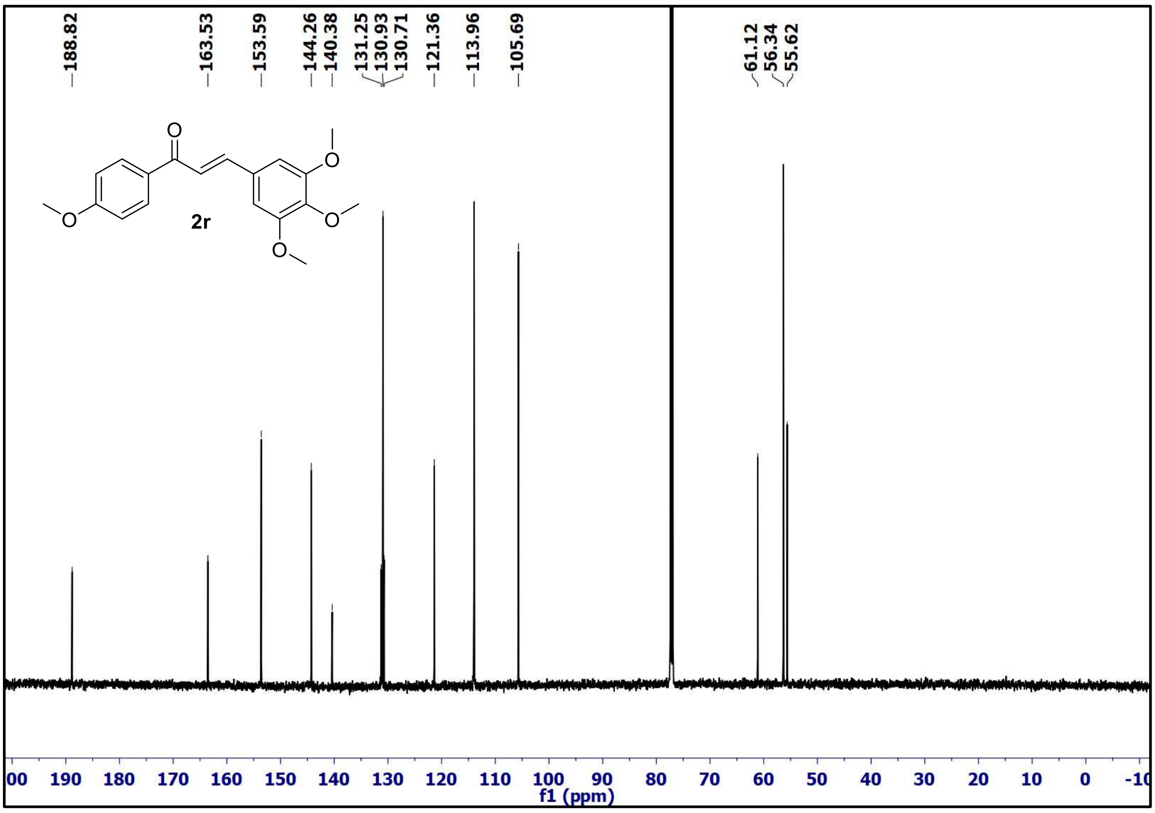

(*E*)-1-(4-Methoxyphenyl)-3-(4-(trifluoromethyl)phenyl)prop-2-en-1-one (2s)

^{1}H NMR (600 MHz, $CDCl_3$)

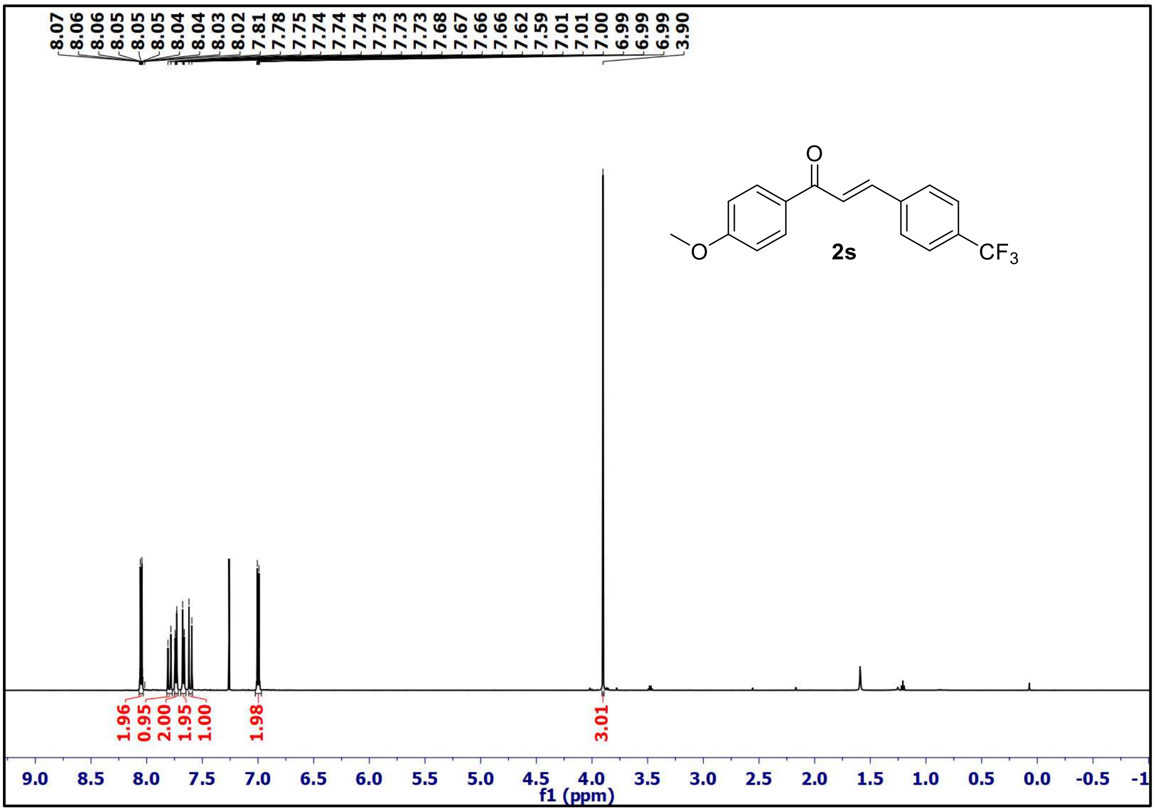

^{13}C NMR (150 MHz, $CDCl_3$)

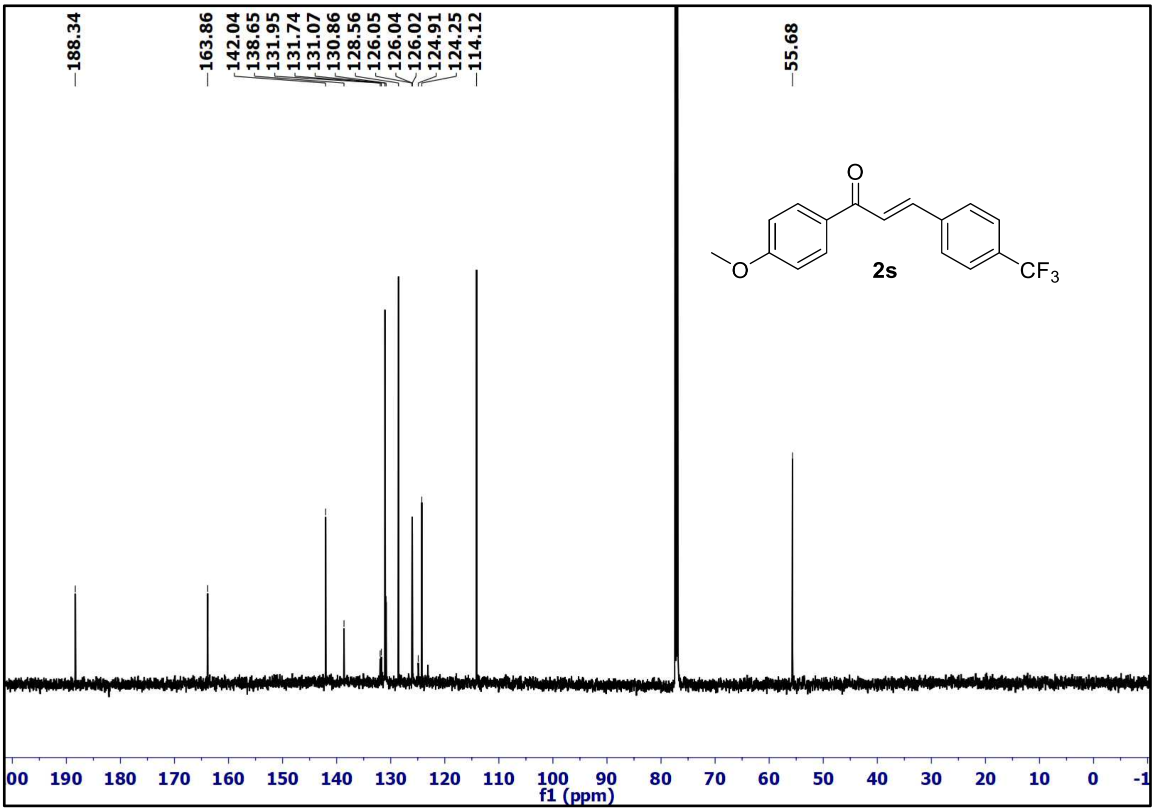

^{19}**F NMR** (564 MHz, $CDCl_3$)

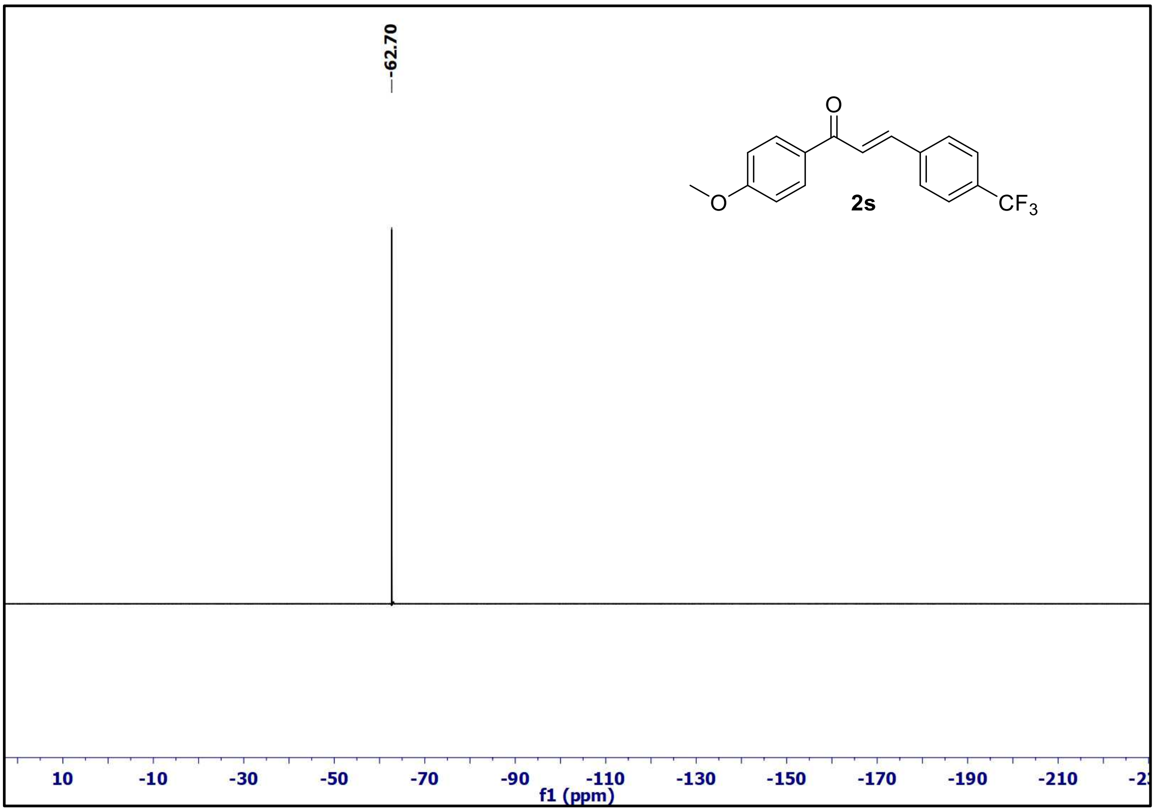

(*E*)-3-(4-Fluorophenyl)-1-(4-methoxyphenyl)prop-2-en-1-one (2t)

^{1}H NMR (600 MHz, $CDCl_3$)

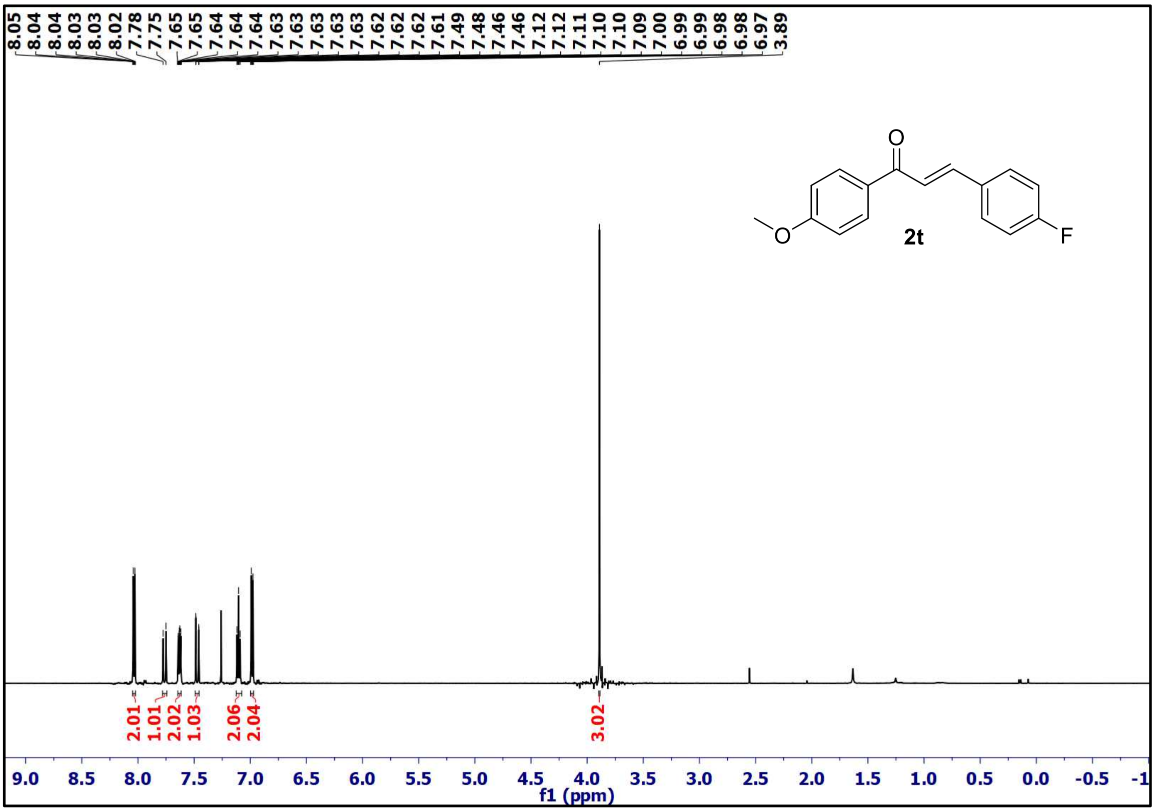

^{13}C NMR (150 MHz, $CDCl_3$)

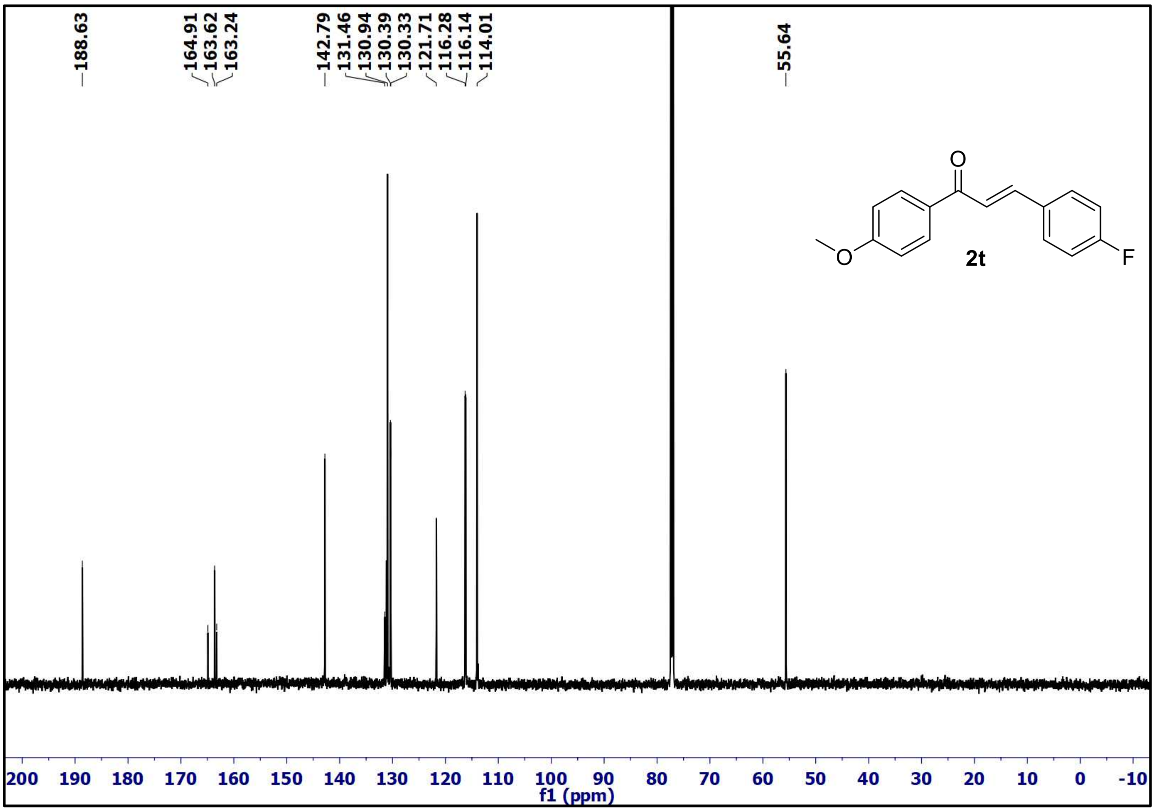

^{19}F NMR (564 MHz, $CDCl_3$)

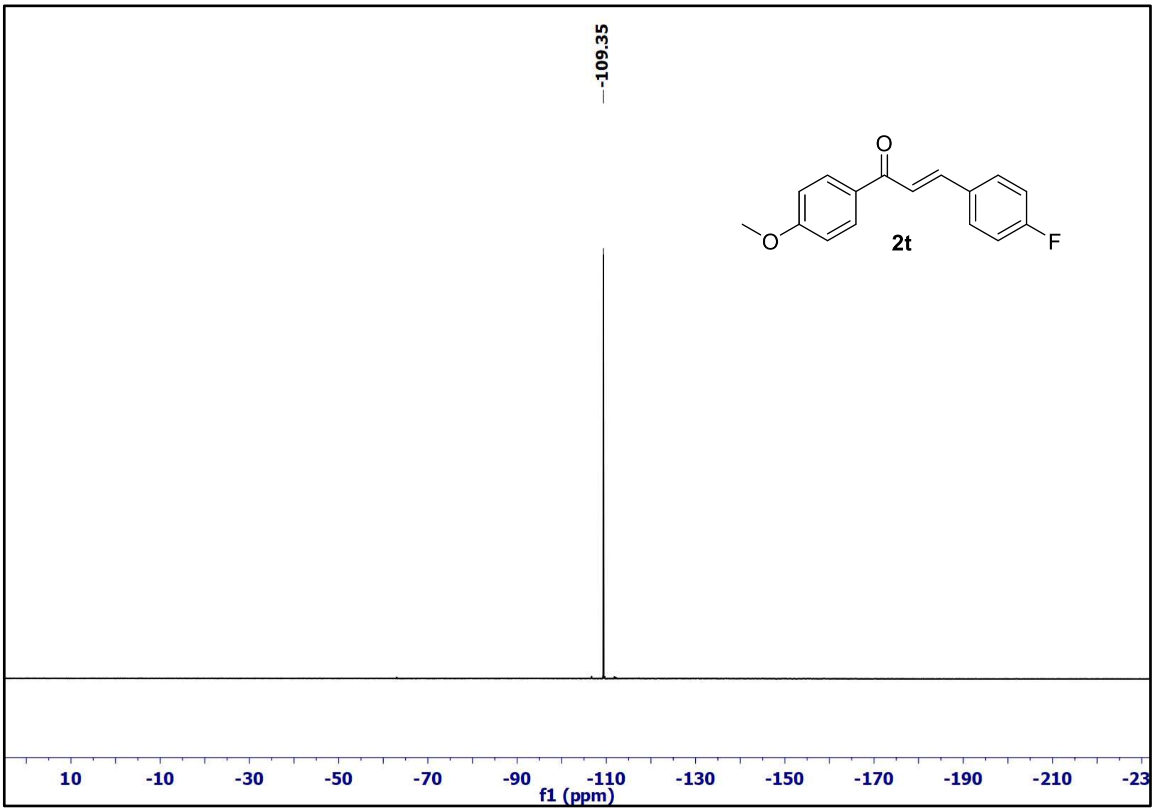

(*E*)-3-(4-Iodophenyl)-1-(4-methoxyphenyl)prop-2-en-1-one (2u)

^{1}**H NMR** (600 MHz, $CDCl_3$)

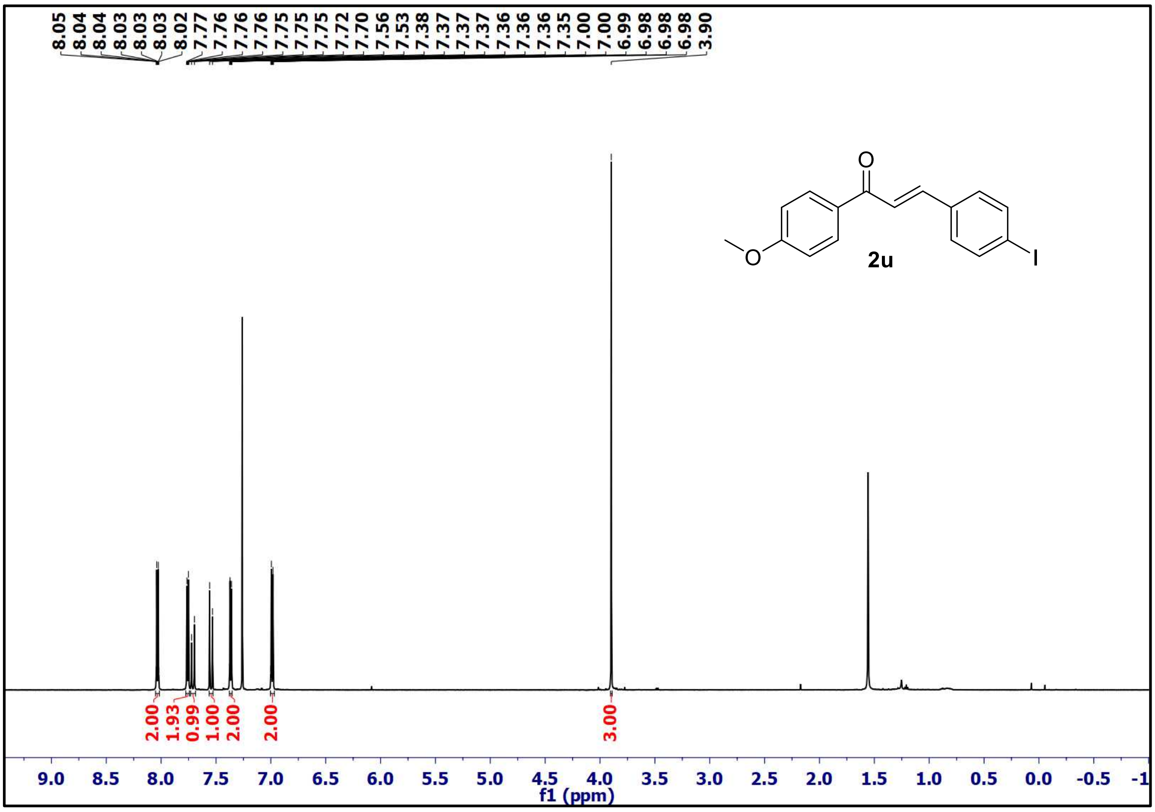

^{13}**C NMR** (150 MHz, $CDCl_3$)

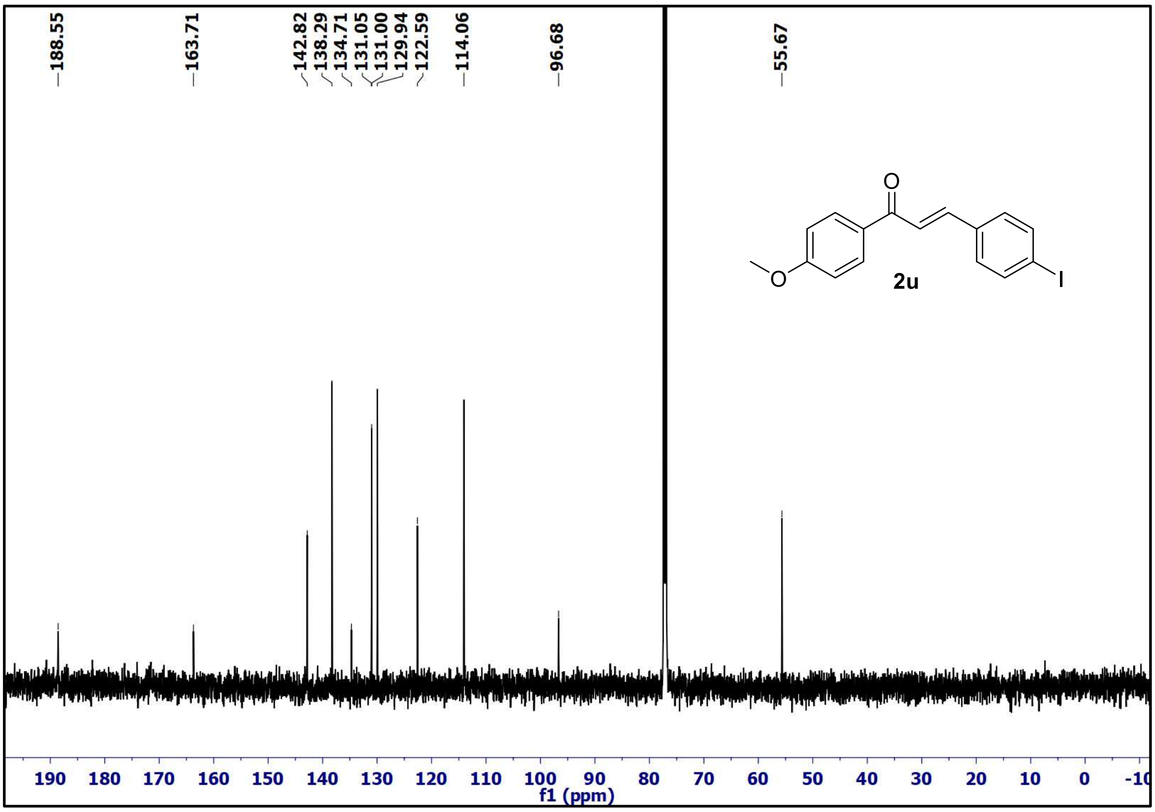

(*E*)-3-(Benzo[d][1,3]dioxol-5-yl)-1-(4-methoxyphenyl)prop-2-en-1-one (2v)

^{1}H NMR (600 MHz, $CDCl_3$)

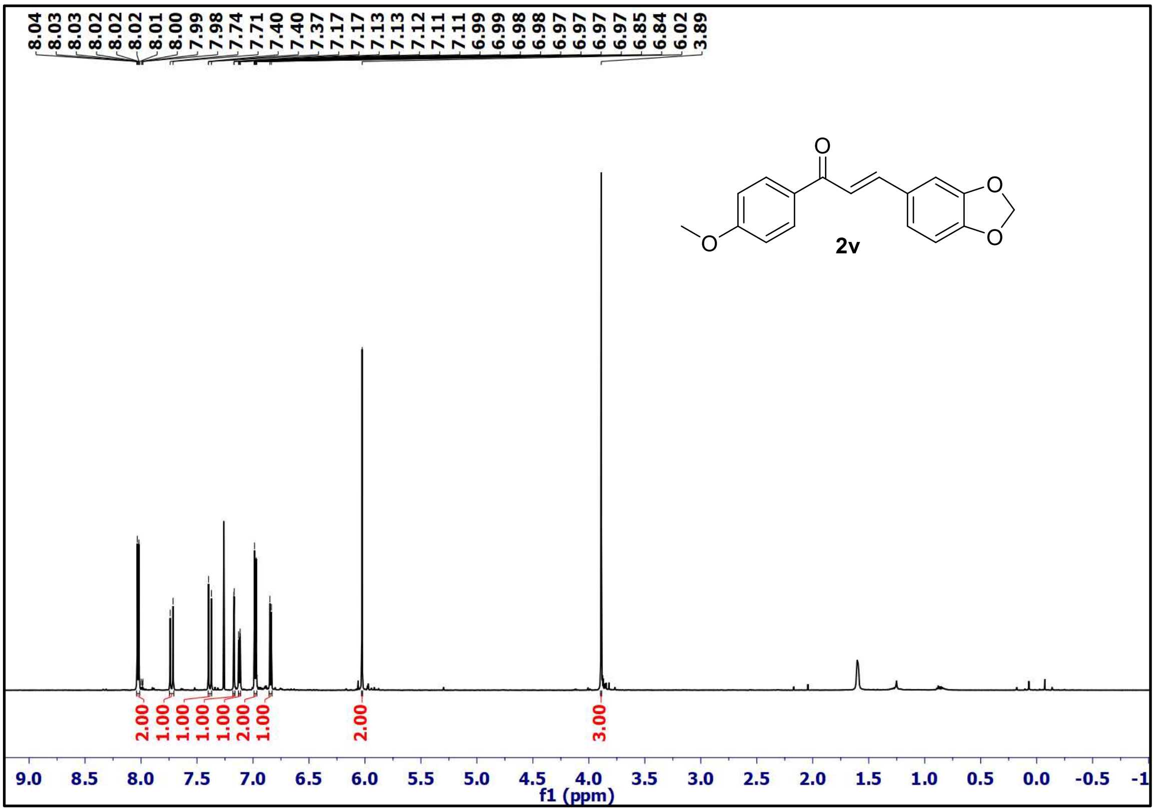

^{13}C NMR (150 MHz, $CDCl_3$)

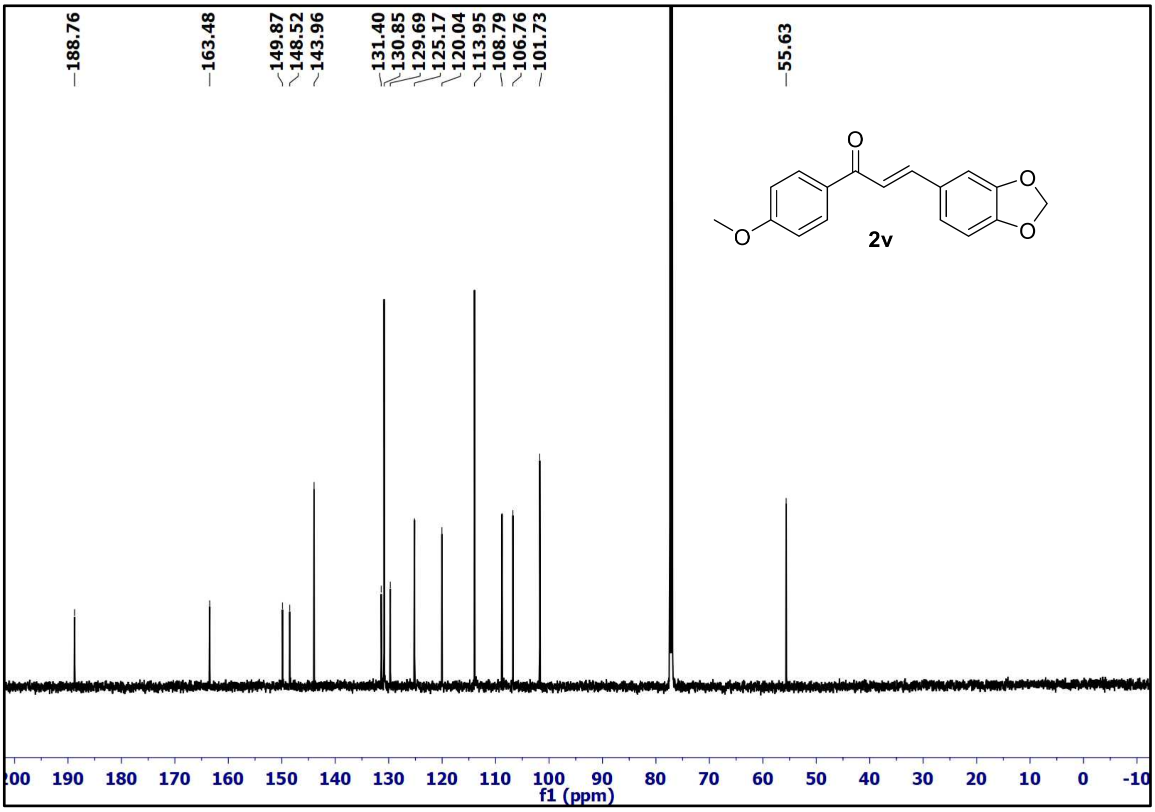

(2*E*,4*E*)-1-(4-Methoxyphenyl)-5-phenylpenta-2,4-dien-1-one (2w)

^{1}H NMR (600 MHz, $CDCl_3$)

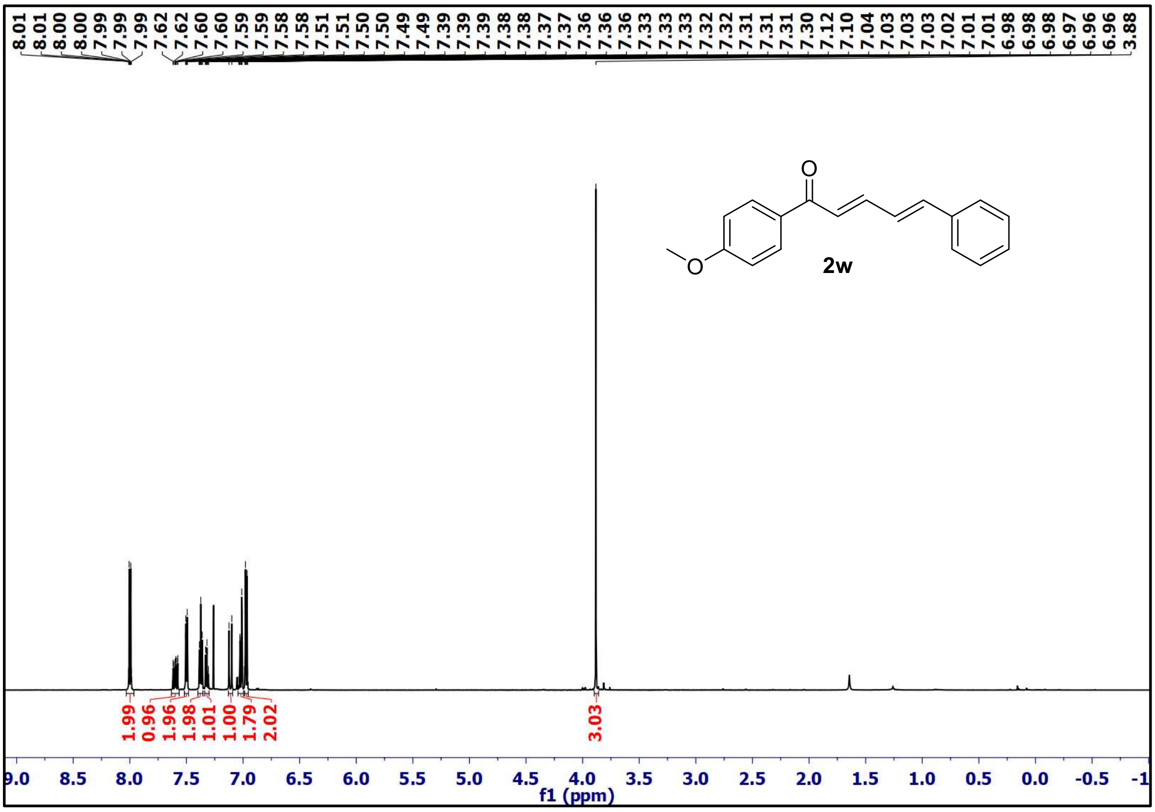

^{13}C NMR (150 MHz, $CDCl_3$)

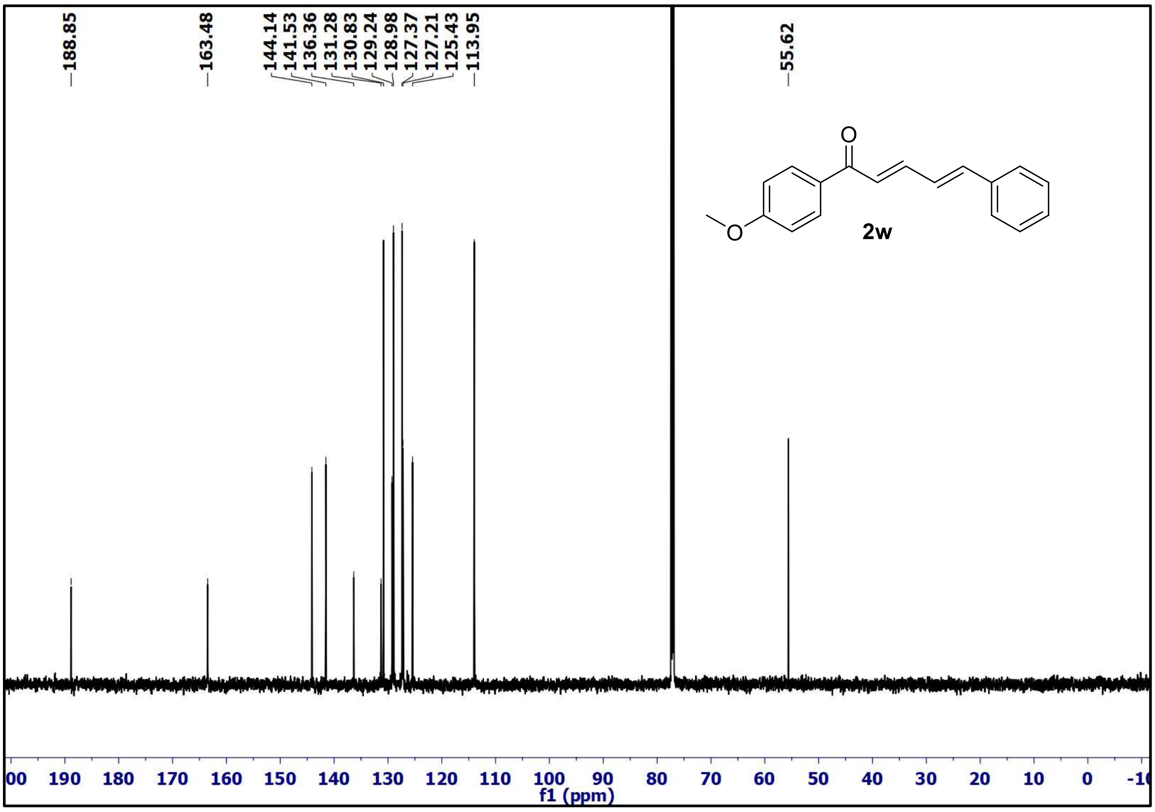

(*E*)-1-(4-Methoxyphenyl)-3-(thiophen-2-yl)prop-2-en-1-one (2x)

^{1}H NMR (600 MHz, $CDCl_3$)

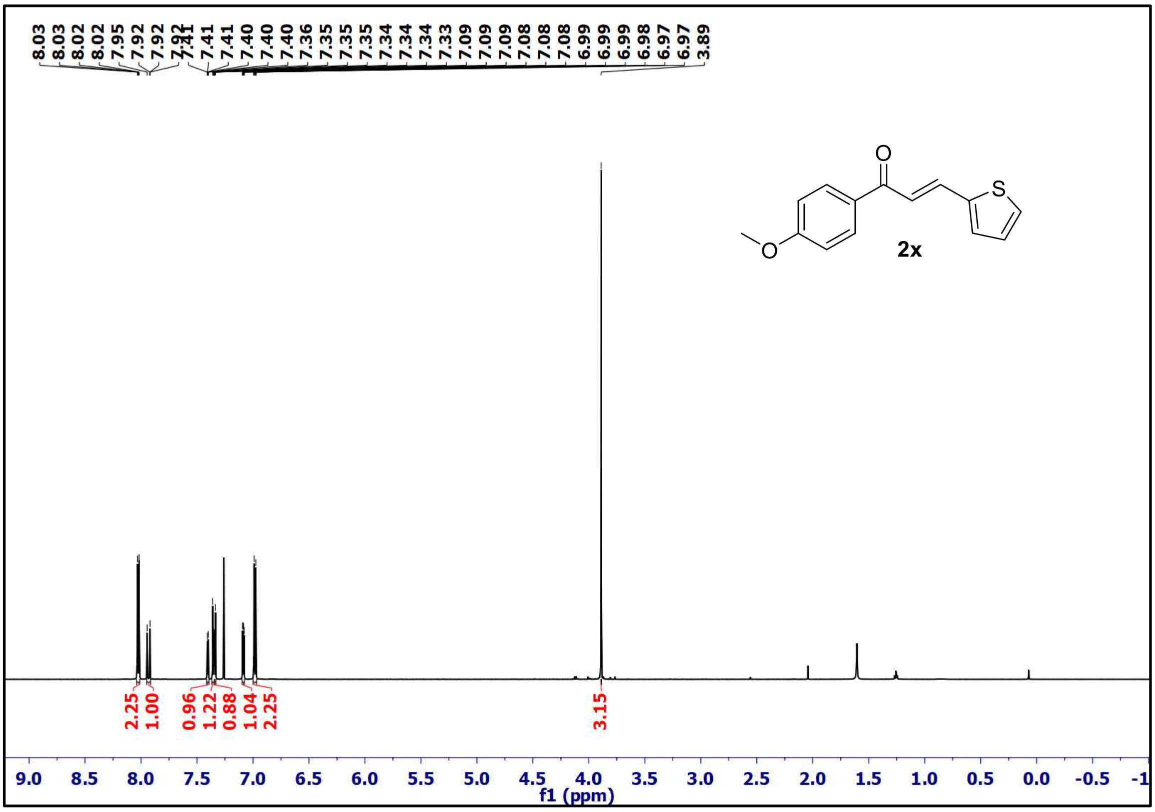

^{13}C NMR (150 MHz, $CDCl_3$)

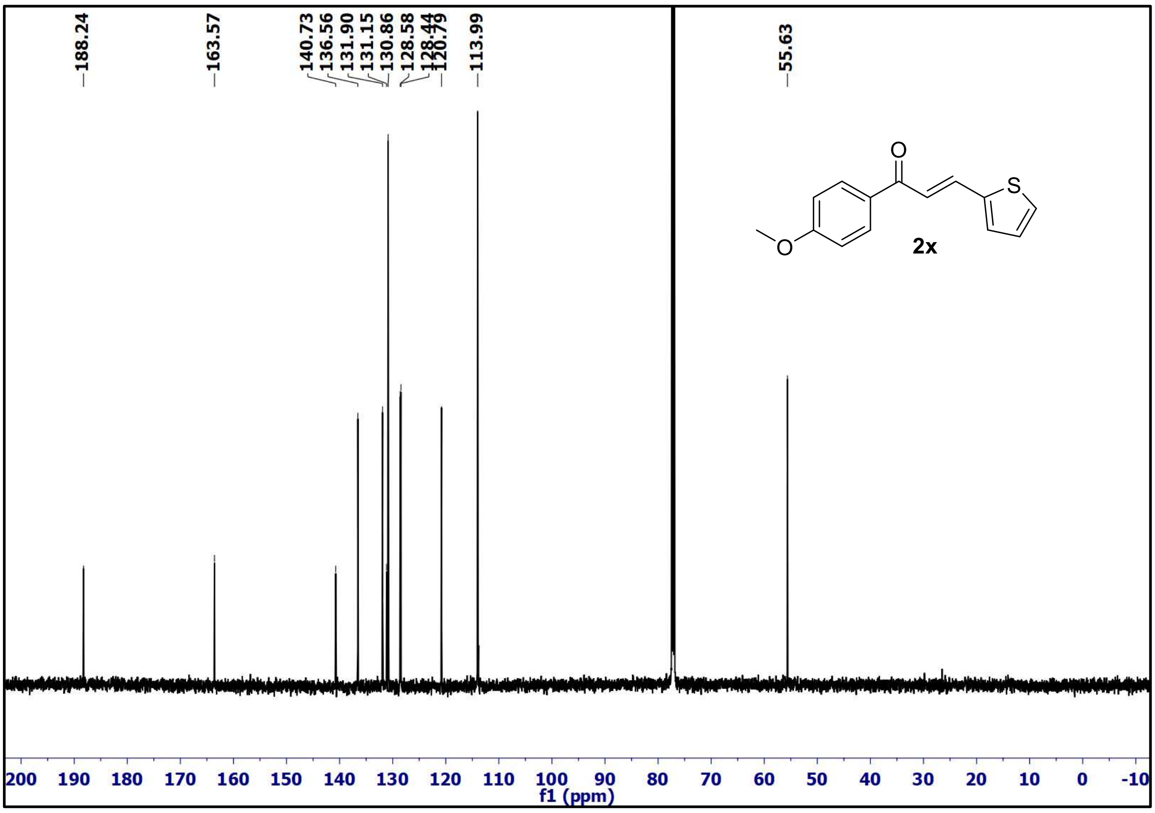

(*E*)-3-(Furan-2-yl)-1-(4-methoxyphenyl)prop-2-en-1-one (2y)

^{1}H NMR (600 MHz, $CDCl_3$)

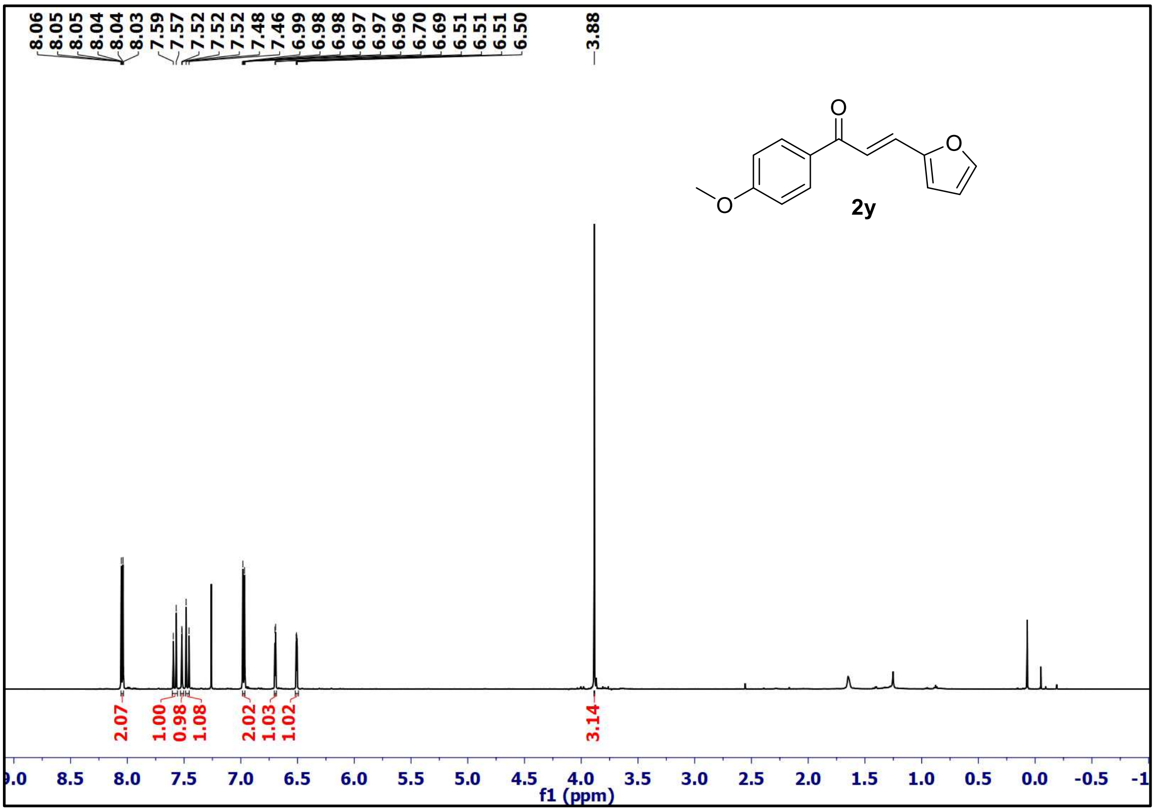

^{13}C NMR (150 MHz, $CDCl_3$)

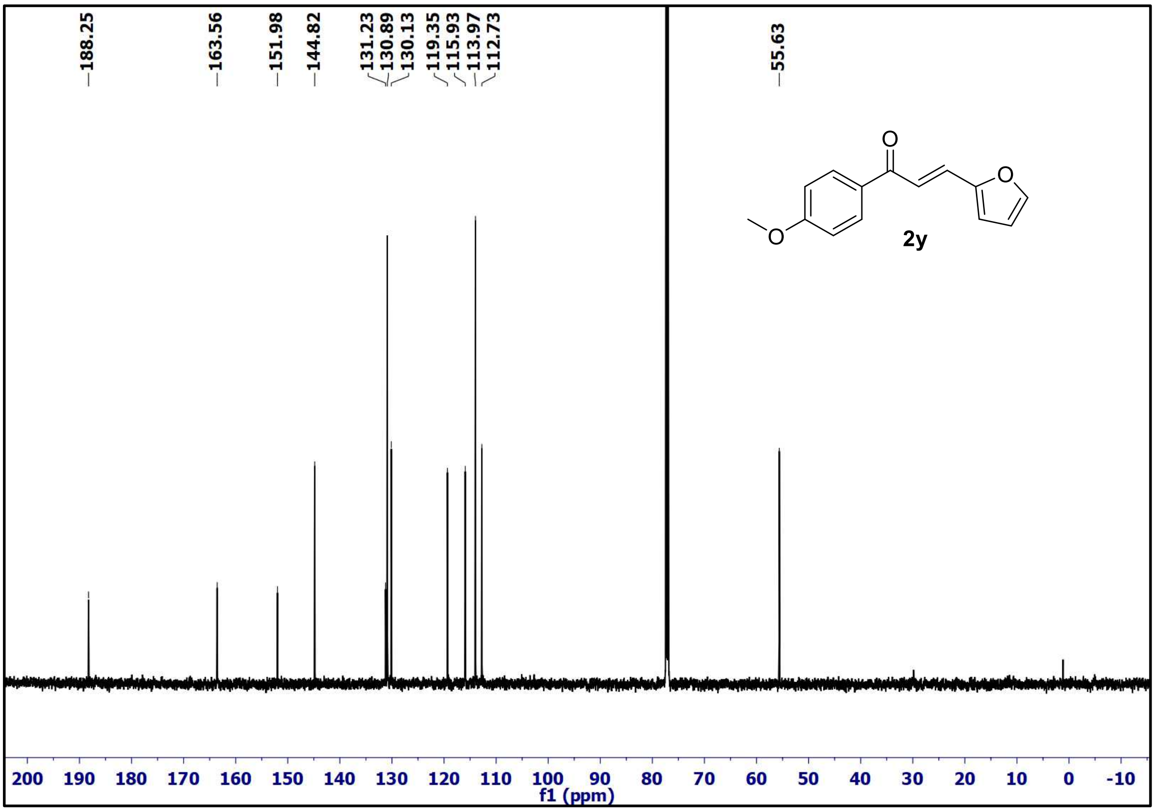

(*E*)-1-(4-Methoxyphenyl)-3-(pyridin-4-yl)prop-2-en-1-one (2z)

^{1}H NMR (600 MHz, $CDCl_3$)

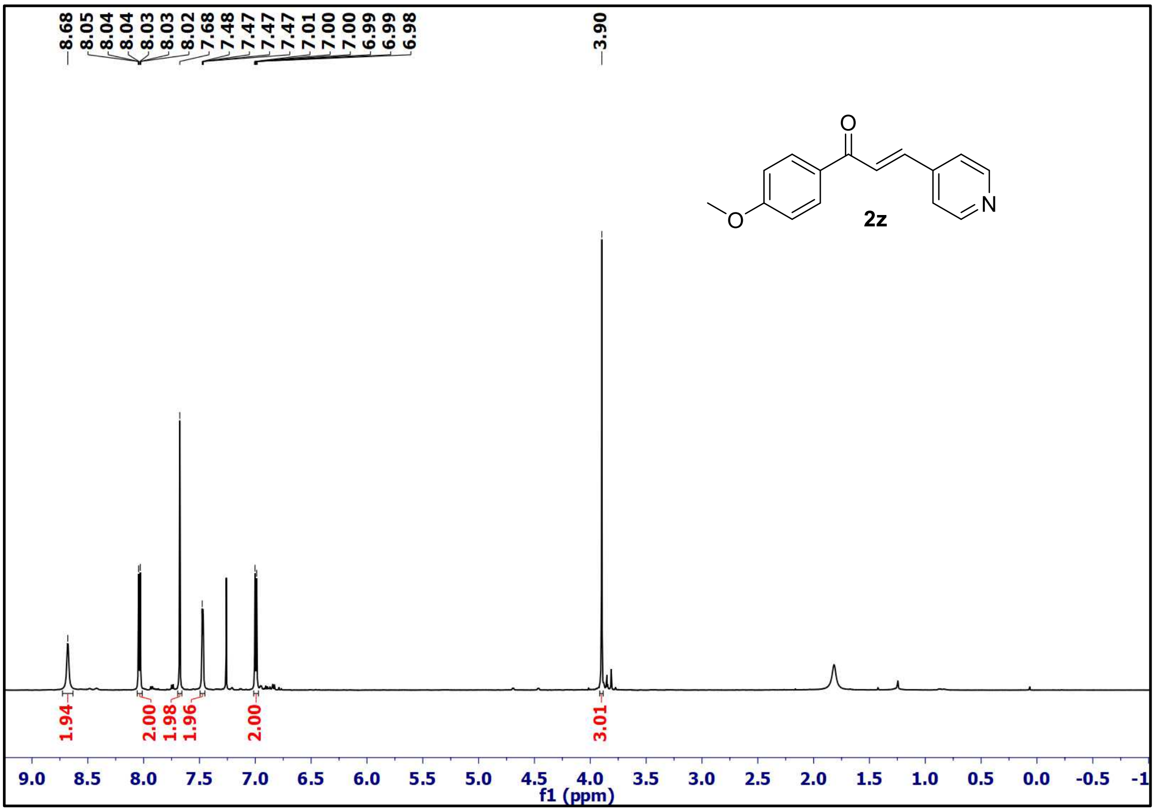

^{13}C NMR (150 MHz, $CDCl_3$)

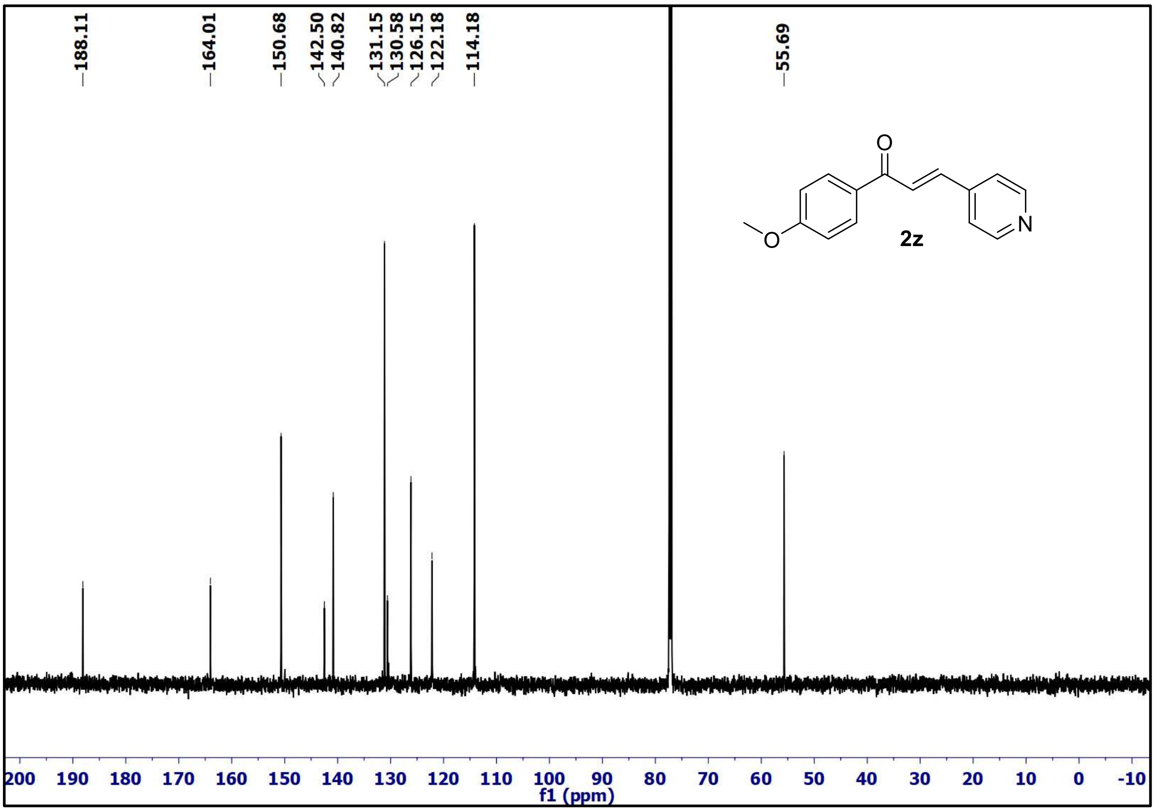

(*E*)-3-Cyclohexyl-1-(4-methoxyphenyl)prop-2-en-1-one (2aa)

^{1}H NMR (600 MHz, $CDCl_3$)

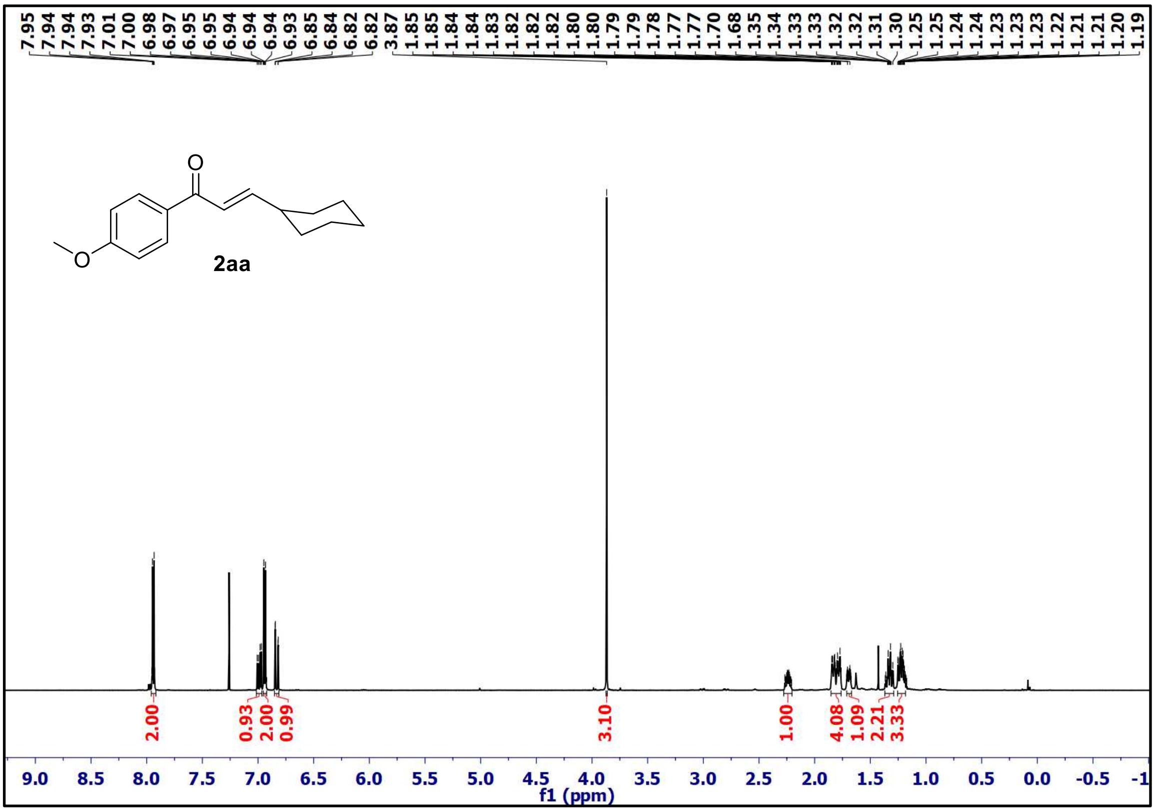

^{13}C NMR (150 MHz, $CDCl_3$)

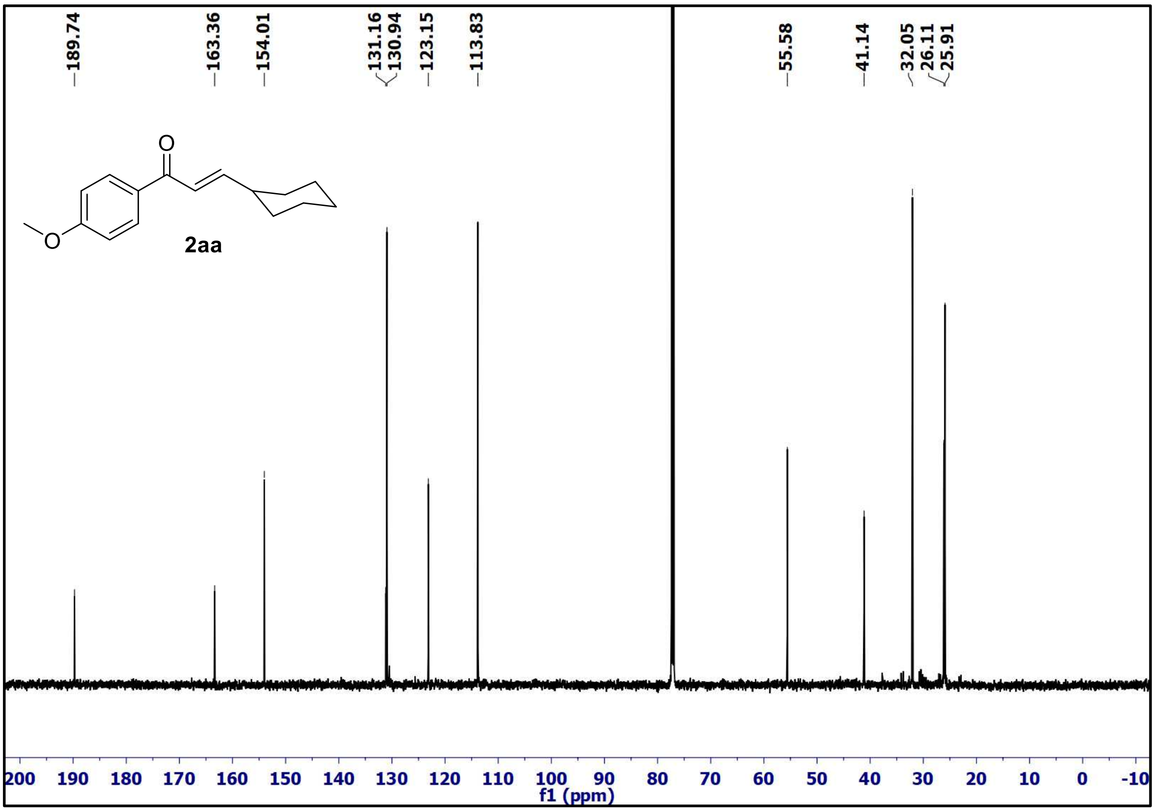

1-(4-Methoxyphenyl)-3,3-diphenylprop-2-en-1-one (2ab)

^{1}**H NMR** (600 MHz, $CDCl_3$)

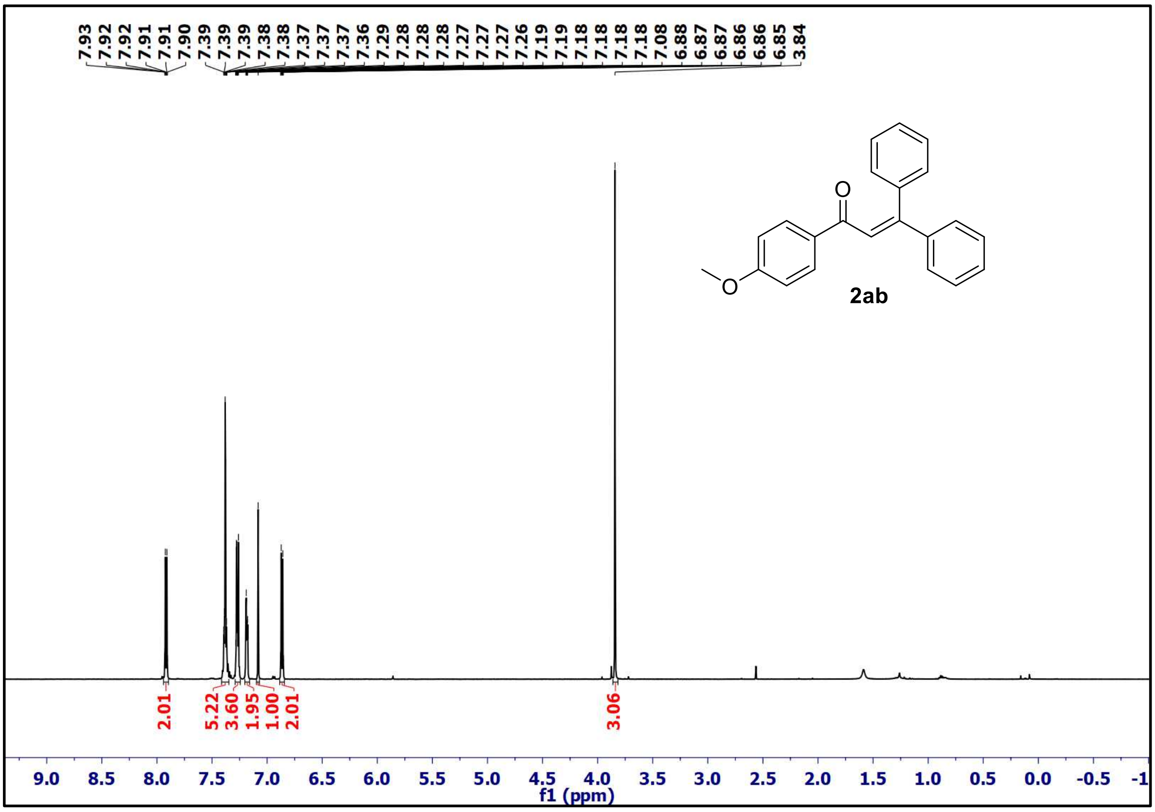

^{13}**C NMR** (150 MHz, $CDCl_3$)

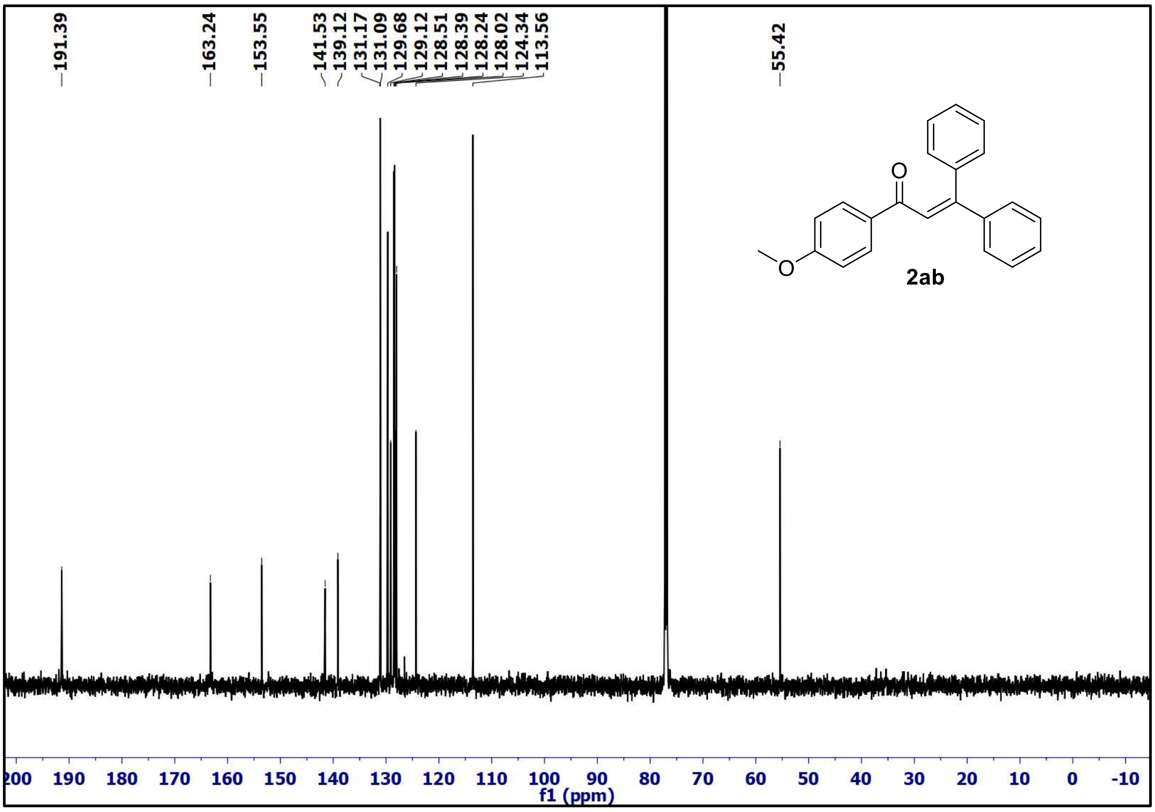

2-(9*H*-Fluoren-9-ylidene)-1-(4-methoxyphenyl)ethan-1-one (2ac)

^{1}H NMR (600 MHz, $CDCl_3$)

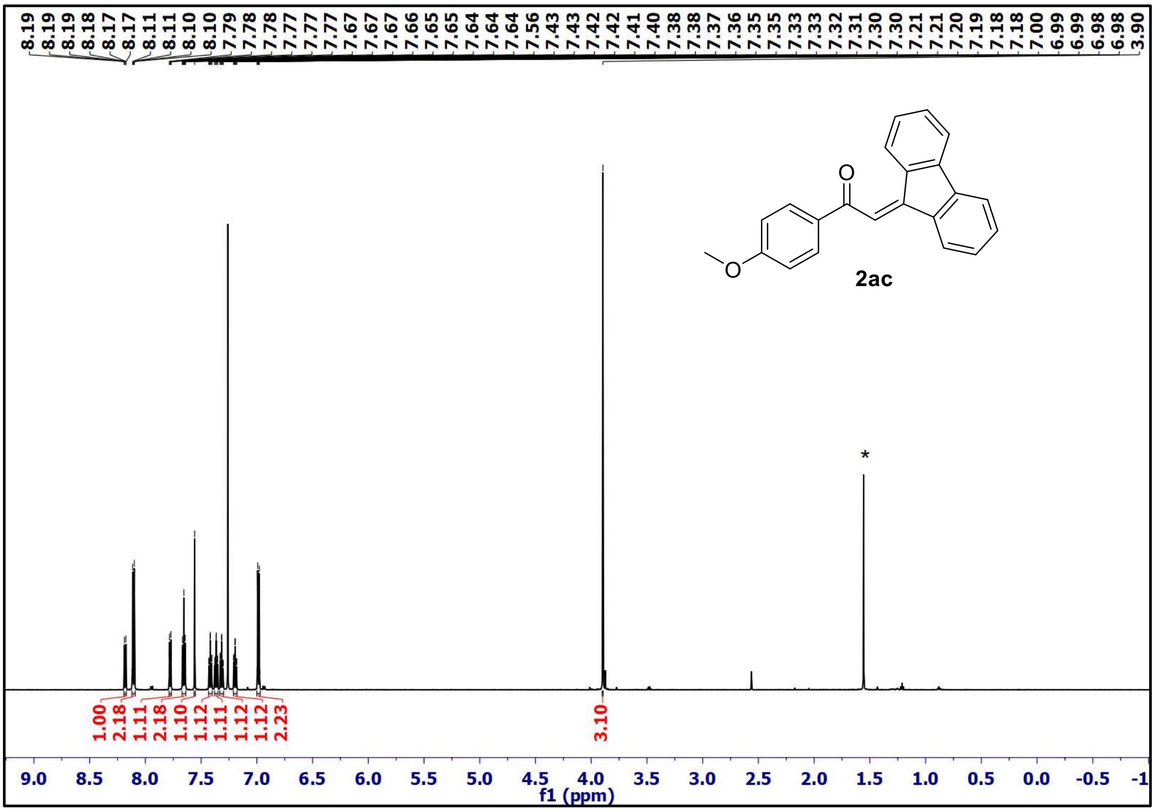

*H_2O residual peak

^{13}C NMR (150 MHz, $CDCl_3$)

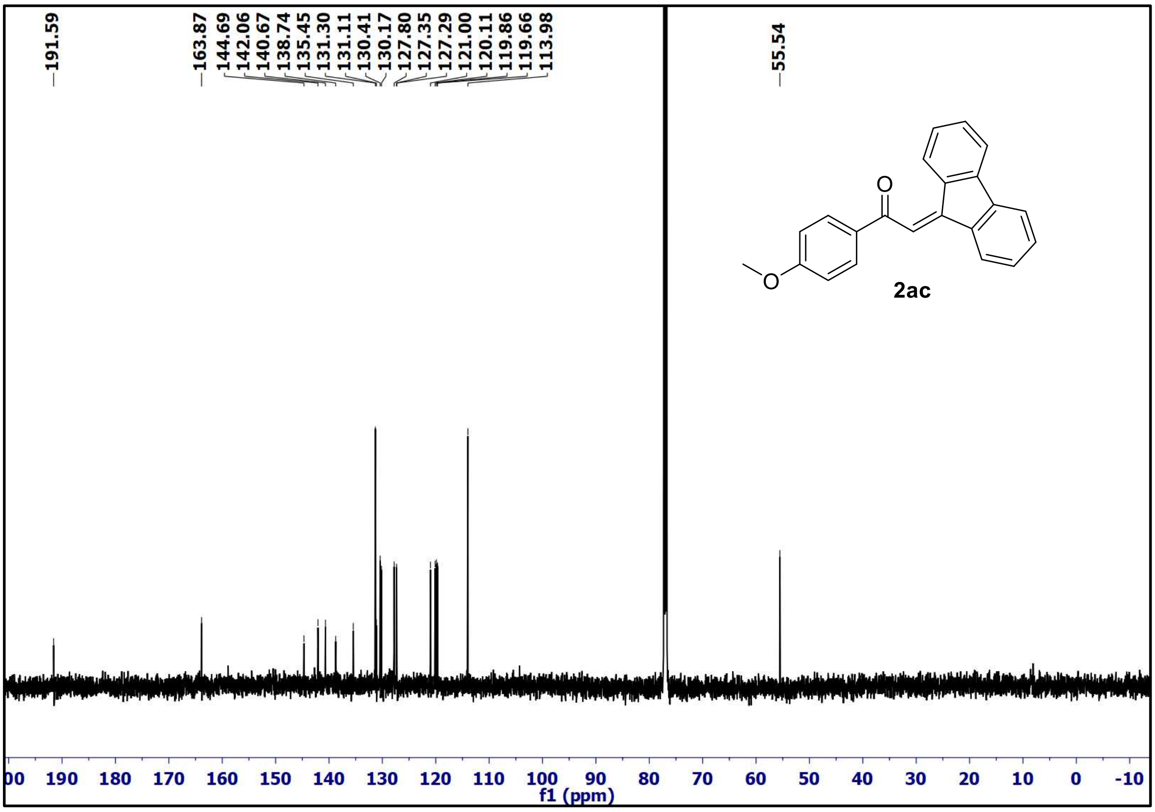

Synthesis and analysis of compounds **2ad-2ai**

General procedure. To a stirred solution of the appropriate amide (0.2 mmol, 1.0 eq.) in anhydrous CPME (0.04 M, 5 mL), $LiCH_2SiMe_3$ (0.3 mmol, 1.5 eq.) was added at room temperature. The resulting reaction mixture was stirred for 30 min then the appropriate aldehyde (0.24 mmol, 1.2 eq.) was added. The mixture was stirred for1 hour and then quenched with H_2O. After extraction with EtOAc (3 x 10 mL), the combined organic layers were dried over Na_2SO_4 and evaporated under reduced pressure. The crude products were purified by flash column chromatography on silica gel.

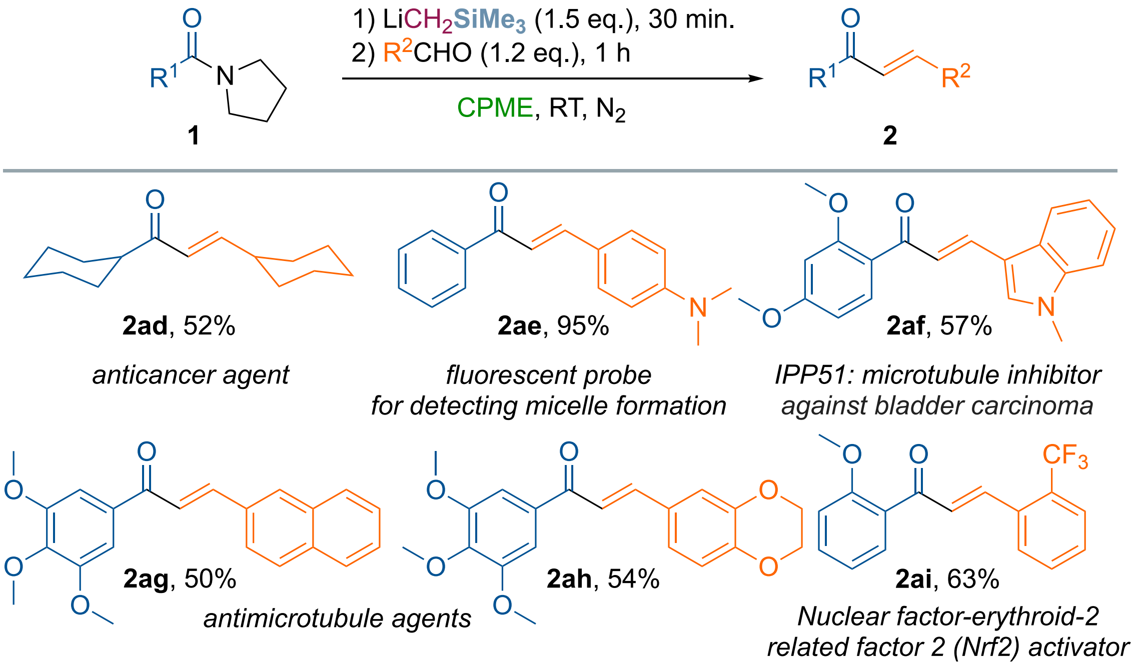

Scheme S6. Synthesis of α,β-unsaturated ketones **2ad-2ai**. Reaction conditions: **1** (0.2 mmol, 1.0 eq.), CPME (5.0 mL), $LiCH_2SiMe_3$ (0.3 mmol, 1.5 eq.) 30 min, RT, under N_2, then aldehyde (0.24 mmol, 1.2 eq.), 1 h. Reported yields refer to isolated products after flash column chromatography on silica gel.

(*E*)-1,3-Dicyclohexylprop-2-en-1-one (2ad): General procedure starting from **1n** and cyclohexanecarbaldehyde. The crude was purified by flash column chromatography on silica gel (petroleum ether/DEE 95/5 v/v) to give **2ad** as a colourless oil (23 mg, 52%, R_f = 0.28 petroleum ether/DEE 95/5 v/v).[26] ^{1}H NMR (600 MHz, $CDCl_3$) δ 6.79 (dd, J = 15.8, 6.8 Hz, 1H), 6.09 (dd, J = 15.9, 1.4 Hz, 1H), 2.58-2.48 (m, 1H), 2.17-2.07 (m, 1H), 1.84-1.71 (m, 8H) superimposed to 1.71-1.64 (m, 2H), 1.39-1.09 (m, 10H). $^{13}C\{^1H\}$ NMR (150 MHz, $CDCl_3$) δ 204.0, 152.1, 126.1, 48.7, 40.7, 31.9, 28.9, 26.0, 26.0, 25.9, 25.8. EI-MS *m/z* (%): 220 (M^+, 1), 137 (100), 119 (8), 67 (14), 55 (59). HRMS (ESI) *m/z*: $[M+Na]^+$ Calcd for $C_{15}H_{24}ONa$ 243.1719; Found 243.1722.

(*E*)-3-(4-(Dimethylamino)phenyl)-1-phenylprop-2-en-1-one (2ae): General procedure starting from **1b** and 4-(*N*,*N*-dimethylamino)benzaldehyde. The crude was purified by flash column chromatography on silica gel (petroleum ether/EtOAc 9/1 v/v) to give **2ae** as a yellow solid (49 mg, 95%, R_f = 0.42 petroleum ether/EtOAc

9/1 v/v), mp. 110.2-111.8 °C.[19] ^{1}H NMR (600 MHz, $CDCl_3$) δ 8.01-7.99 (m, 2H), 7.79 (d, *J* = 15.5 Hz, 1H), 7.57-7.54 (m, 3H), 7.50-7.47 (m, 2H), 7.34 (d, *J* =15.5 Hz, 1H), 6.71-6.79 (m, 2H), 3.05 (s, 6H). $^{13}C\{^1H\}$ NMR (150 MHz, $CDCl_3$) δ 190.9, 152.2, 146.0, 139.2, 132.3, 130.6, 128.6, 128.5, 122.8, 117.1, 112.0, 40.3. EI-MS *m/z* (%): 251 (M^+, 100), 250 (51), 174 (14), 77 (34). HRMS (ESI) *m/z*: $[M+H]^+$ Calcd for $C_{17}H_{18}NO$ 252.1383; Found 252.1384.

(*E*)-1-(2,4-Dimethoxyphenyl)-3-(1-methyl-*1H*-indol-3-yl)prop-2-en-1-one (2af): General procedure starting from **1af** and 1-methyl-1*H*-indole-3-carbaldehyde. The crude was purified by flash column chromatography on silica gel (DCM/DEE 98/2 v/v) to give **2af** as a yellow solid (36 mg, 57%, R_f = 0.15 DCM/DEE 98/2 v/v), mp. 134.0-135.8 °C.[27] ^{1}H NMR (600 MHz, $CDCl_3$) δ 8.00 (d, *J* = 8.0 Hz, 1H), 7.94 (d, *J* = 15.7 Hz, 1H), 7.79 (d, *J* = 8.6 Hz, 1H), 7.59 (d, *J* = 15.8 Hz, 1H), 7.39 (br s, 1H), 7.36 (d, *J* = 8.1 Hz, 1H), 7.33-7.30 (m, 1H), 7.28 (dd, *J* = 7.9, 1.3 Hz, 1H), 6.58 (dd, *J* = 8.6, 2.2 Hz, 1H), 6.53 (d, *J* = 2.4 Hz, 1H), 3.94 (s, 3H), 3.88 (s, 3H), 3.81 (br s, 3H). $^{13}C\{^1H\}$ NMR (150 MHz, $CDCl_3$) δ 190.9, 163.7, 160.2, 138.4, 136.5, 134.2, 132.8, 126.3, 123.1, 123.0, 122.8, 121.4, 121.0, 113.3, 110.1, 105.2, 98.9, 55.9, 55.6, 33.3. HRMS (ESI) *m/z*: $[M+H]^+$ Calcd for $C_{20}H_{20}NO_3$ 322.1438; Found 322.1446.

(*E*)-3-(Naphthalen-2-yl)-1-(3,4,5-trimethoxyphenyl)prop-2-en-1-one (2ag): General procedure starting from **1ag** and 2-naphthaldehyde. The crude was purified by flash column chromatography on silica gel (petroleum ether/EtOAc 85/15 v/v) to give **2ag** as a white solid (34 mg, 50%, R_f= 0.22 petroleum ether/EtOAc 85/15 v/v), mp. 132.1-136.4 °C.[27] ^{1}H NMR (600 MHz, $CDCl_3$) δ 8.04 (br s, 1H), 7.99 (d, *J* = 15.6 Hz, 1H), 7.92-7.84 (m, 3H), 7.82-7.79 (m, 1H), 7.59 (d, *J* = 15.6 Hz, 1H), 7.56-7.51 (m, 2H), 7.32 (s, 2H), 3.97 (s, 6H), 3.95 (s, 3H). $^{13}C\{^1H\}$ NMR (150 MHz, $CDCl_3$) δ 189.4, 153.3, 145.0, 142.7, 134.5, 133.7, 133.5, 132.5, 130.8, 128.9, 128.8, 128.0, 127.6, 126.96, 123.78, 122.02, 106.30, 61.14, 56.6. HRMS (ESI) *m/z*: $[M+H]^+$ Calcd for $C_{22}H_{21}O_4$ 349.1434; Found 349.1431.

(*E*)-3-(2,3-Dihydrobenzo[b][1,4]dioxin-6-yl)-1-(3,4,5-trimethoxyphenyl)prop-2-en-1-one (2ah): General procedure starting from **1ag** and 2,3-dihydrobenzo[b][1,4]dioxine-6-carbaldehyde. The crude was purified by flash column chromatography on silica gel (petroleum ether/EtOAc 8/2 v/v) to give **2ah** as a yellow solid (40 mg, 54%, R_f= 0.18 petroleum ether/EtOAc 8/2 v/v), mp. 110.4-114.6 °C.[28] ^{1}H NMR (600 MHz, $CDCl_3$) δ 7.72 (d, *J* = 15.5 Hz, 1H), 7.34 (d, *J* = 15.5 Hz, 1H), 7.26 (s, 2H), 7.20 (d, *J* = 2.1 Hz, 1H), 7.15 (dd, *J* = 8.4, 2.1 Hz, 1H), 6.90 (d, *J* = 8.3 Hz, 1H), 4.32-4.28 (m, 4H), 3.94 (s, 6H) superimposed to 3.93 (s, 3H). $^{13}C\{^1H\}$ NMR (150 MHz, $CDCl_3$) δ 189.2, 153.2, 146.1, 144.5, 143.8, 142.4, 133.8, 128.6, 122.8, 120.0, 117.8, 116.9, 106.1, 64.7, 64.3, 61.0, 56.4. HRMS (ESI) *m/z*: $[M+H]^+$ Calcd for $C_{20}H_{21}O_6$ 357.1333; Found 357.1335.

(*E*)-1-(2-Methoxyphenyl)-3-(2-(trifluoromethyl)phenyl)prop-2-en-1-one (2ai): General procedure starting from **1c** and 2-(trifluoromethyl)benzaldehyde. The crude was purified by flash column chromatography on silica gel (petroleum ether/EtOAc 9/1 v/v) to give **2ai** as a yellow oil (39 mg, 63%, R_f = 0.30 petroleum

ether/EtOAc 9/1 v/v).[29] ^{1}H NMR (600 MHz, $CDCl_3$) δ 7.96-7.91 (m, 1H), 7.9 (d, *J* = 7.9 Hz, 1H), 7.71 (d, *J* = 7.9 Hz, 1H), 7.63 (dd, *J* = 7.6, 1.8 Hz, 1H), 7.57 (t, *J* = 7.5 Hz, 1H), 7.50-7.46 (m, 2H), 7.30 (d, *J* = 15.7 Hz, 1H), 7.05 (td, *J* = 7.5, 1.0 Hz, 1H), 7.00 (d, *J* = 8.4 Hz, 1H), 3.89 (s, 3H). $^{13}C\{^1H\}$ NMR (150 MHz, $CDCl_3$) δ 192.9, 158.3, 138.7, 134.4, 133.3, 132.2, 131.2, 130.7, 129.5, 129.3 (q, *J* = 30.4 Hz, 1C), 128.8, 128.1, 126.3 (q, *J* = 5.5 Hz, 1C), 124.1 (q, *J* = 273.9 Hz, 1C), 121.0, 111.7, 55.8. ^{19}F NMR (564 MHz, $CDCl_3$) δ -55.83 (s, 3F). EI-MS *m/z* (%): 306 (M^+, 20), 151 (61), 135 (100), 77 (46). HRMS (ESI) *m/z*: $[M+H]^+$ Calcd for $C_{17}H_{14}F_3O_2$ 307.0940; Found 307.0946.

^{1}H and ^{13}C NMR spectra of compounds **2ad-2ai**

(*E*)-1,3-Dicyclohexylprop-2-en-1-one (2ad)

^{1}H NMR (600 MHz, CDCl$_3$)

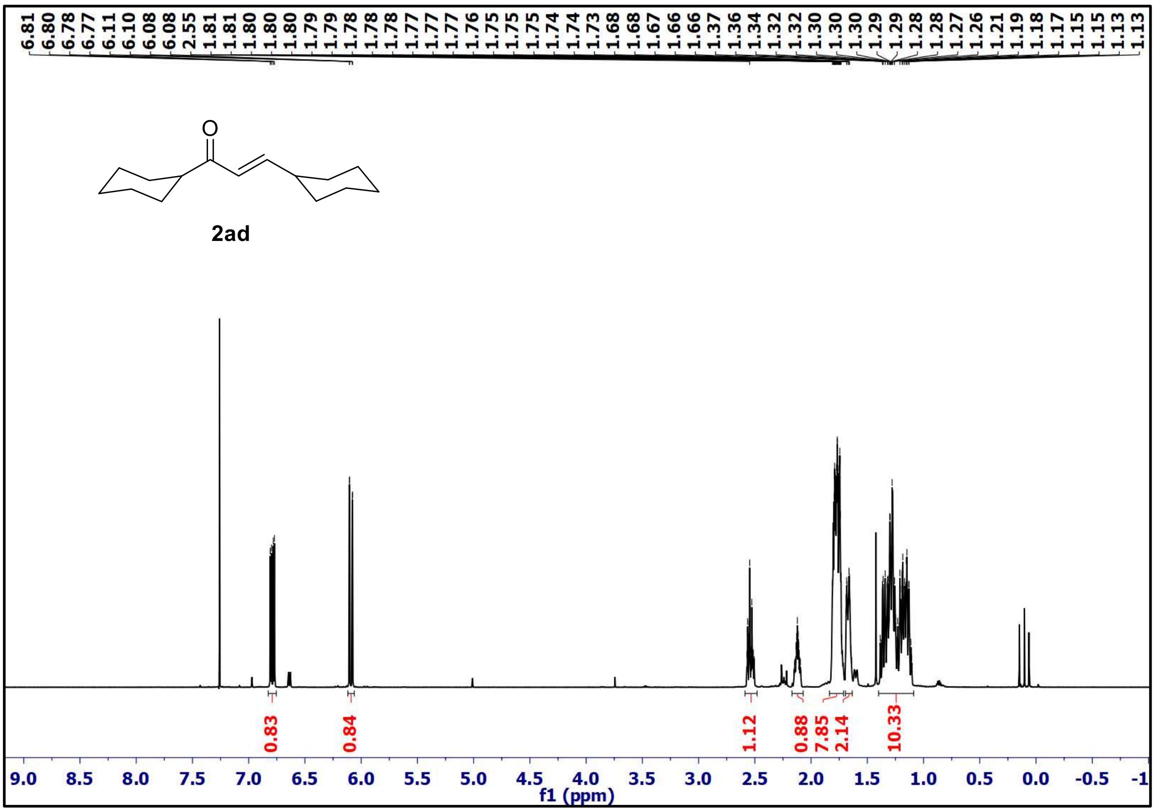

^{13}C NMR (150 MHz, CDCl$_3$)

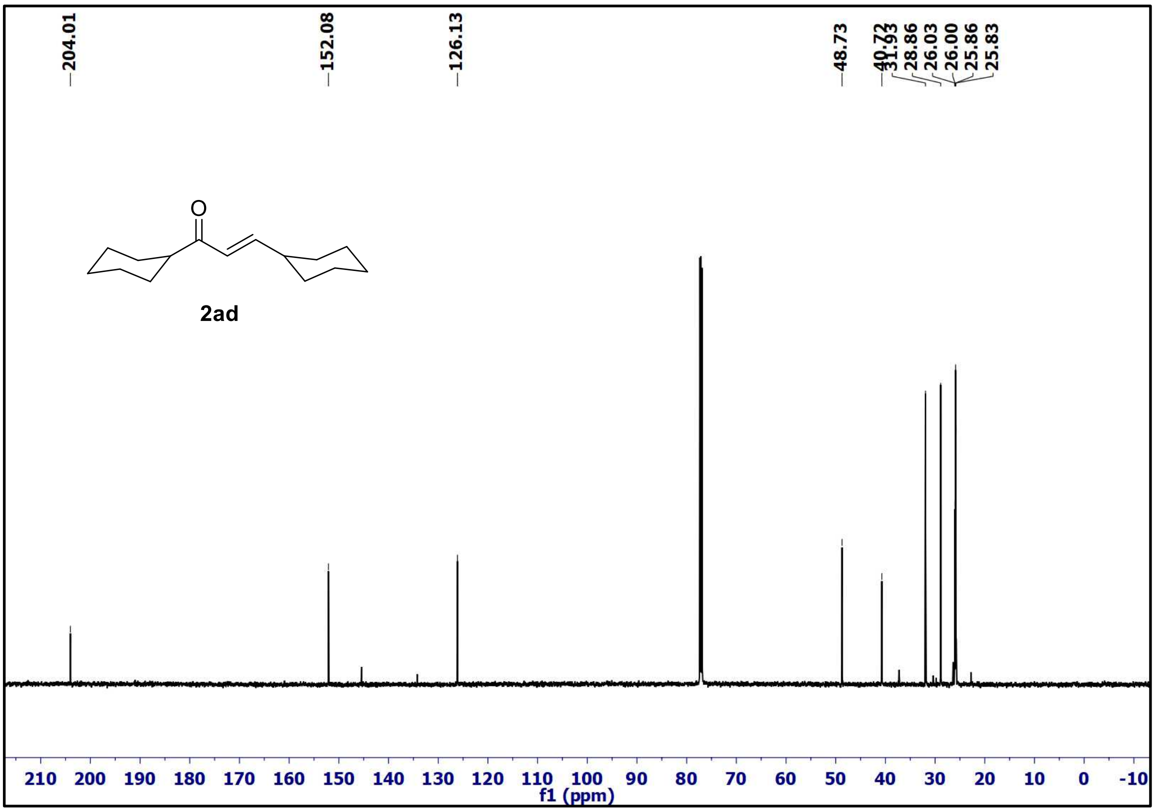

(*E*)-3-(4-(Dimethylamino)phenyl)-1-phenylprop-2-en-1-one (2ae)

^{1}H NMR (600 MHz, $CDCl_3$)

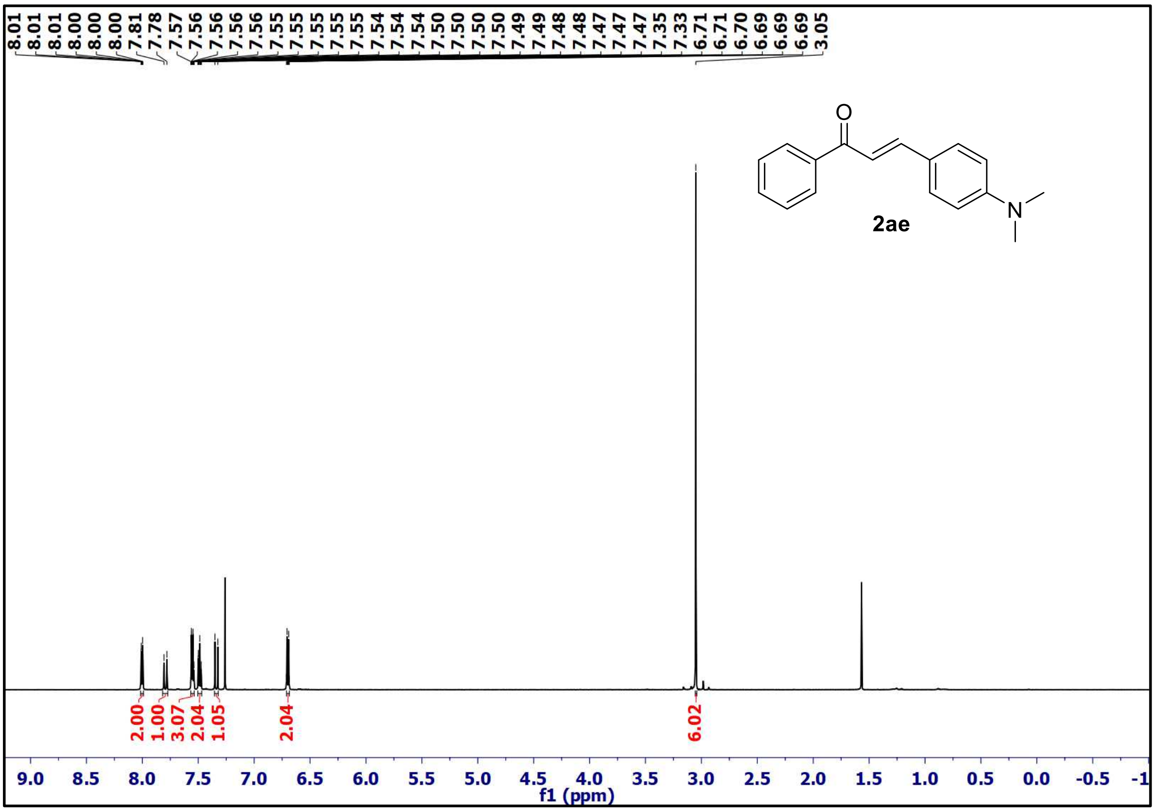

^{13}C NMR (150 MHz, $CDCl_3$)

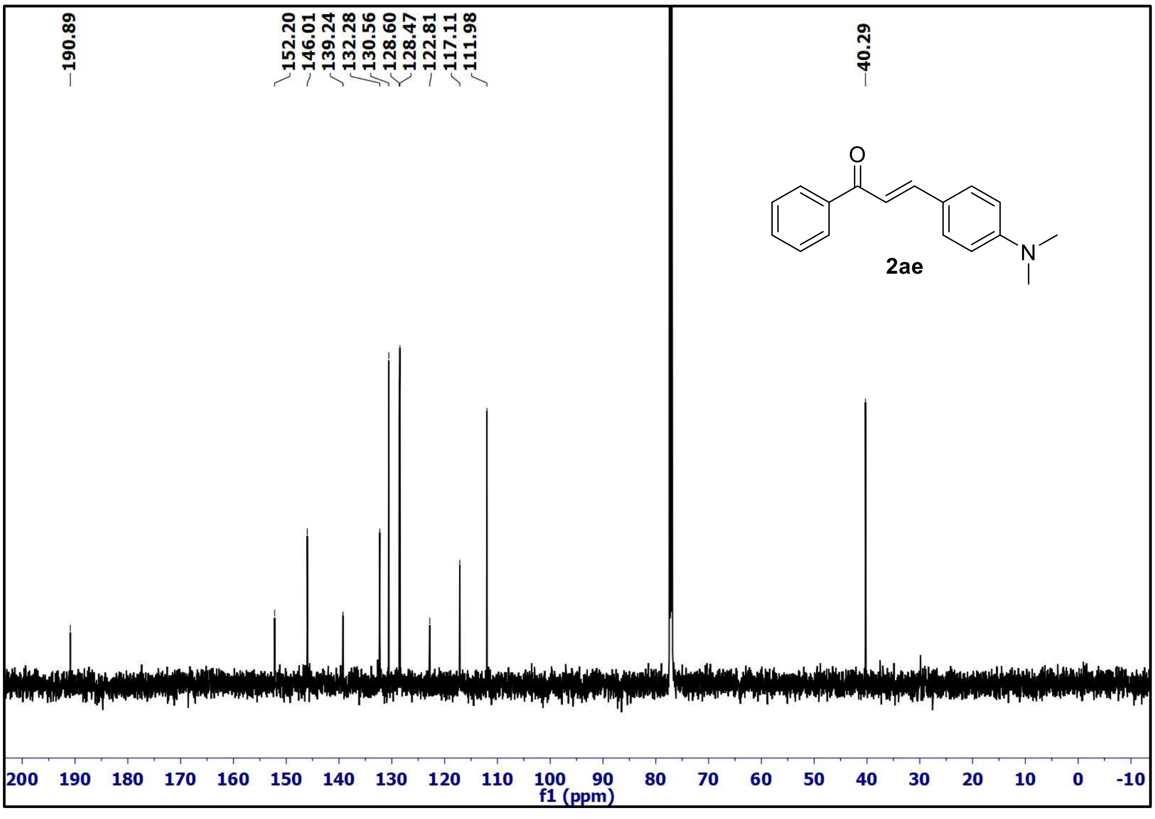

(*E*)-1-(2,4-Dimethoxyphenyl)-3-(1-methyl-*1H*-indol-3-yl)prop-2-en-1-one (2af)

^{1}H NMR (600 MHz, $CDCl_3$)

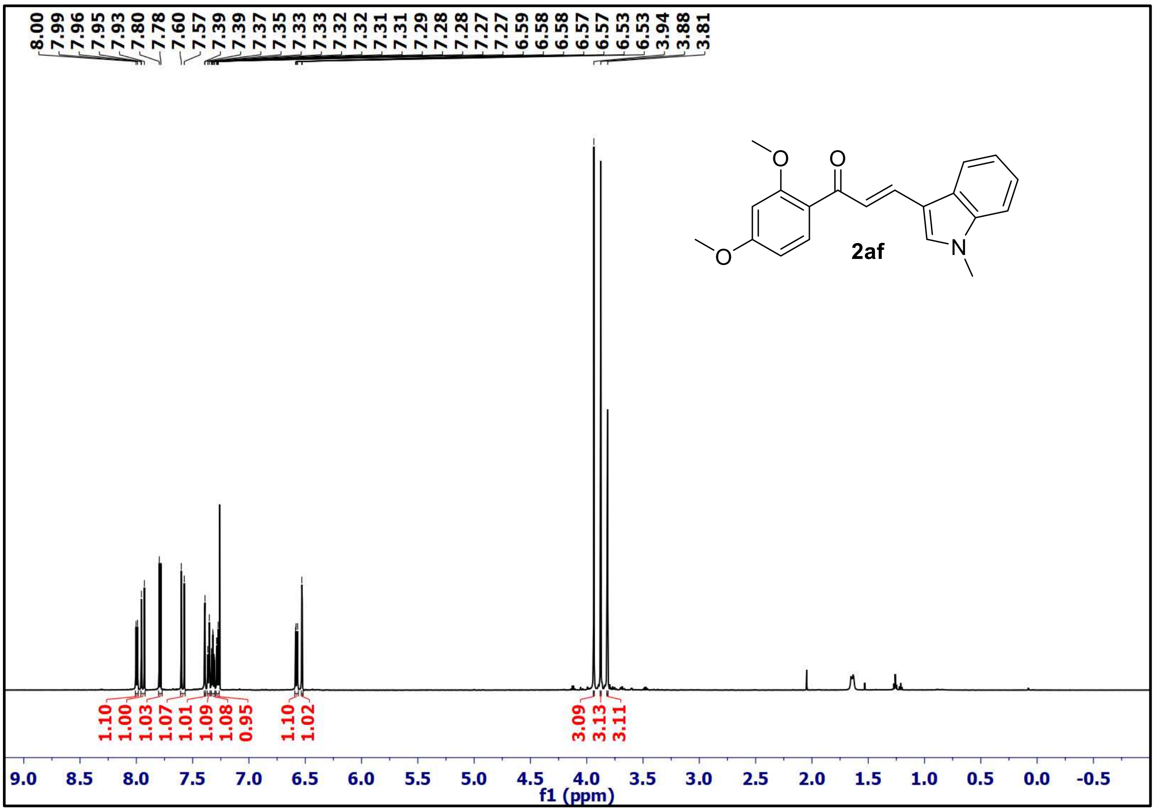

^{13}C NMR (150 MHz, $CDCl_3$)

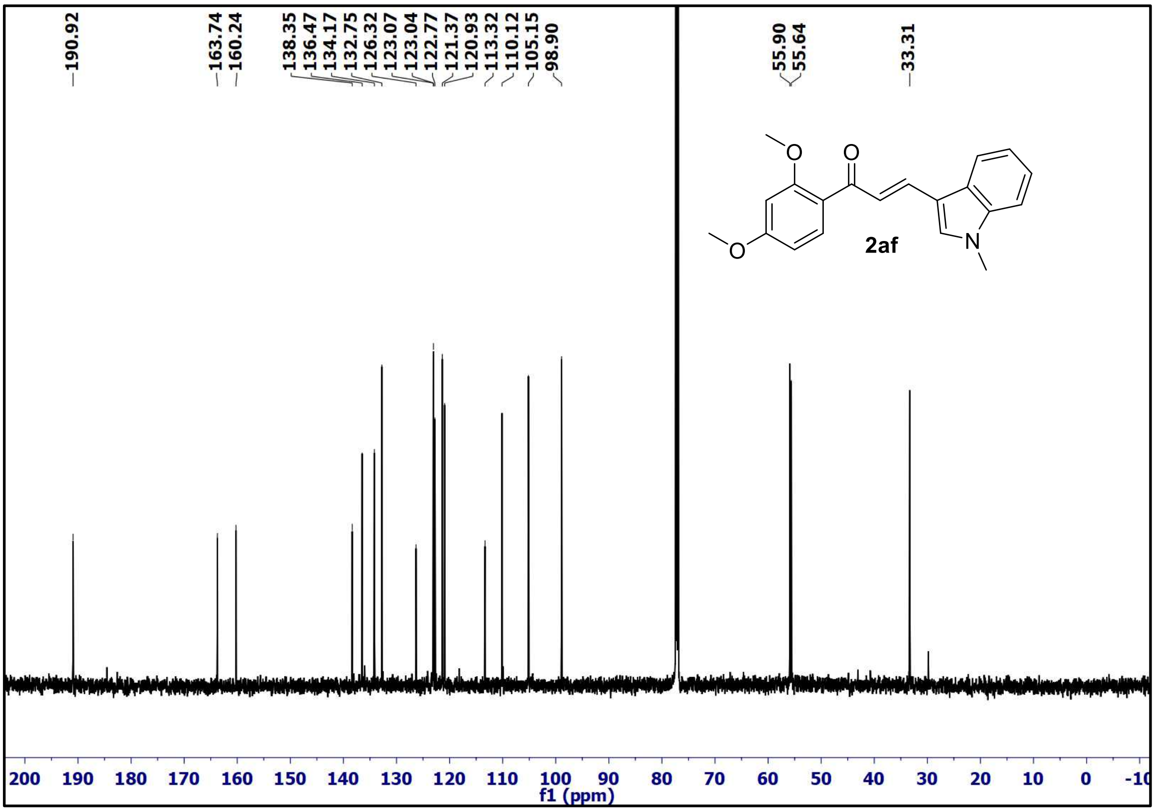

(*E*)-3-(Naphthalen-2-yl)-1-(3,4,5-trimethoxyphenyl)prop-2-en-1-one (2ag)

^{1}H NMR (600 MHz, $CDCl_3$)

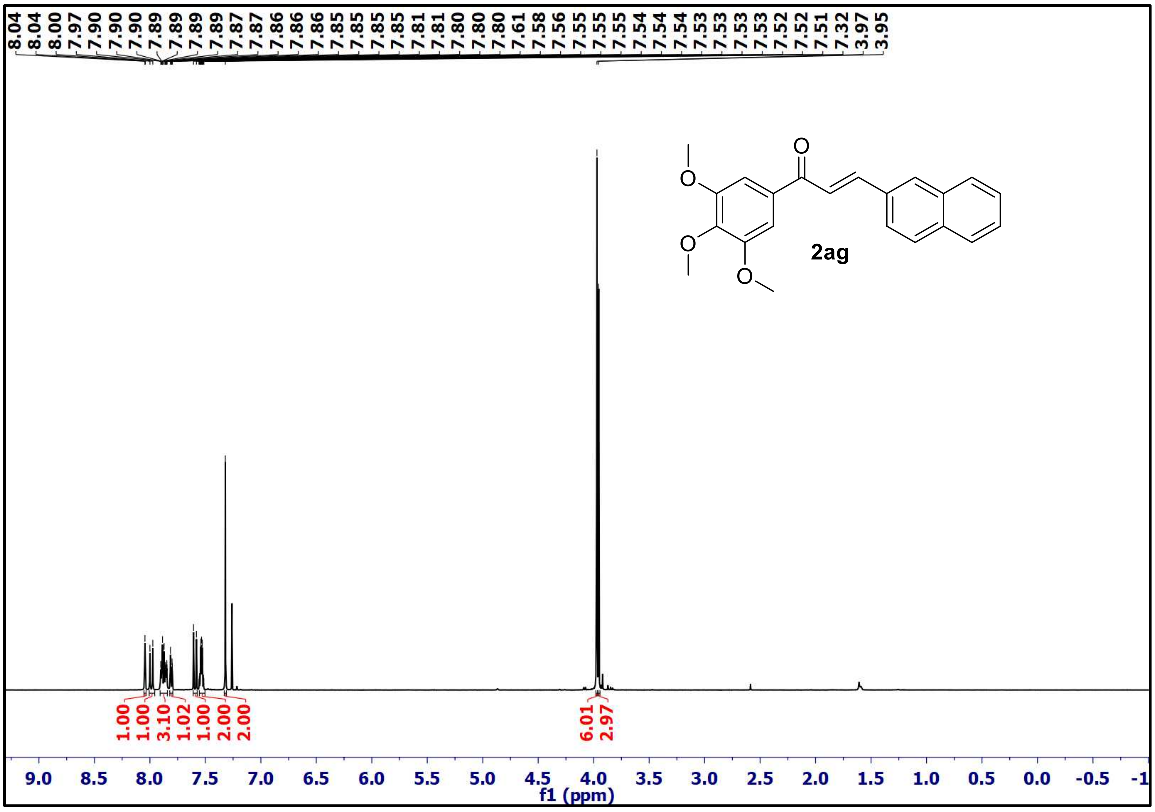

^{13}C NMR (150 MHz, $CDCl_3$)

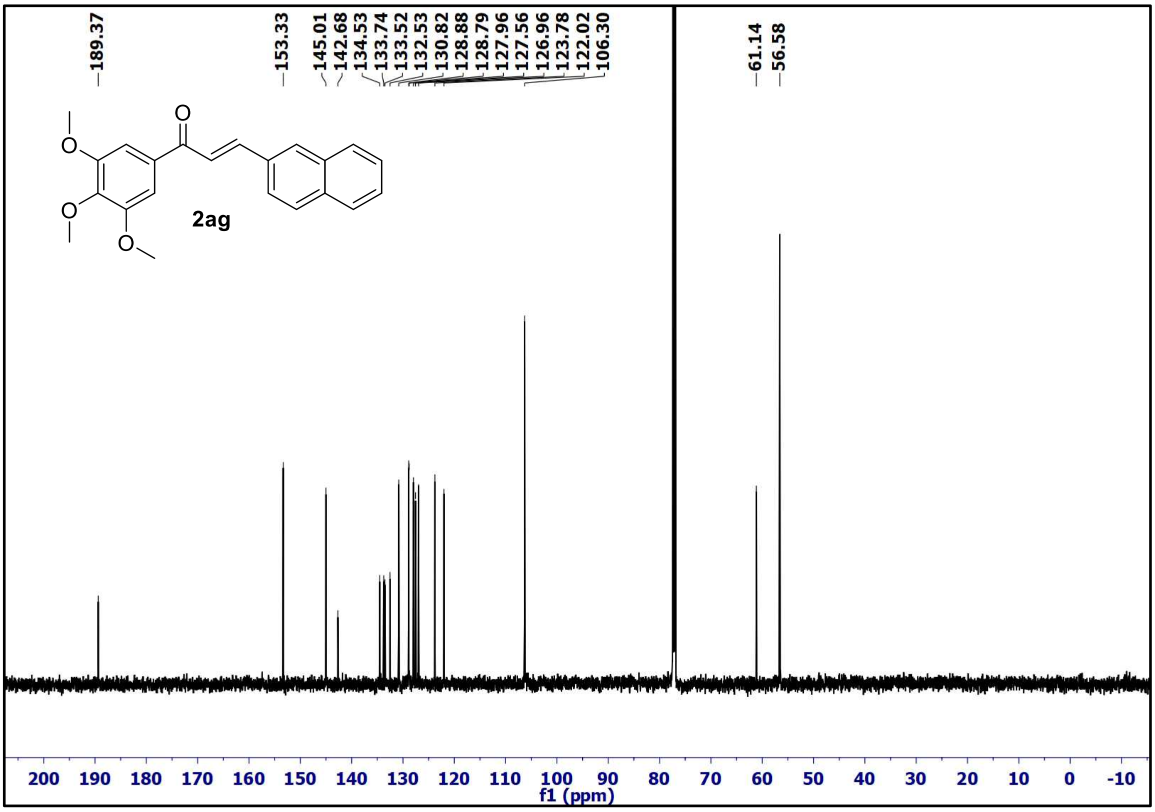

(*E*)-3-(2,3-Dihydrobenzo[b][1,4]dioxin-6-yl)-1-(3,4,5-trimethoxyphenyl)prop-2-en-1-one (2ah)

^{1}**H NMR** (600 MHz, $CDCl_3$)

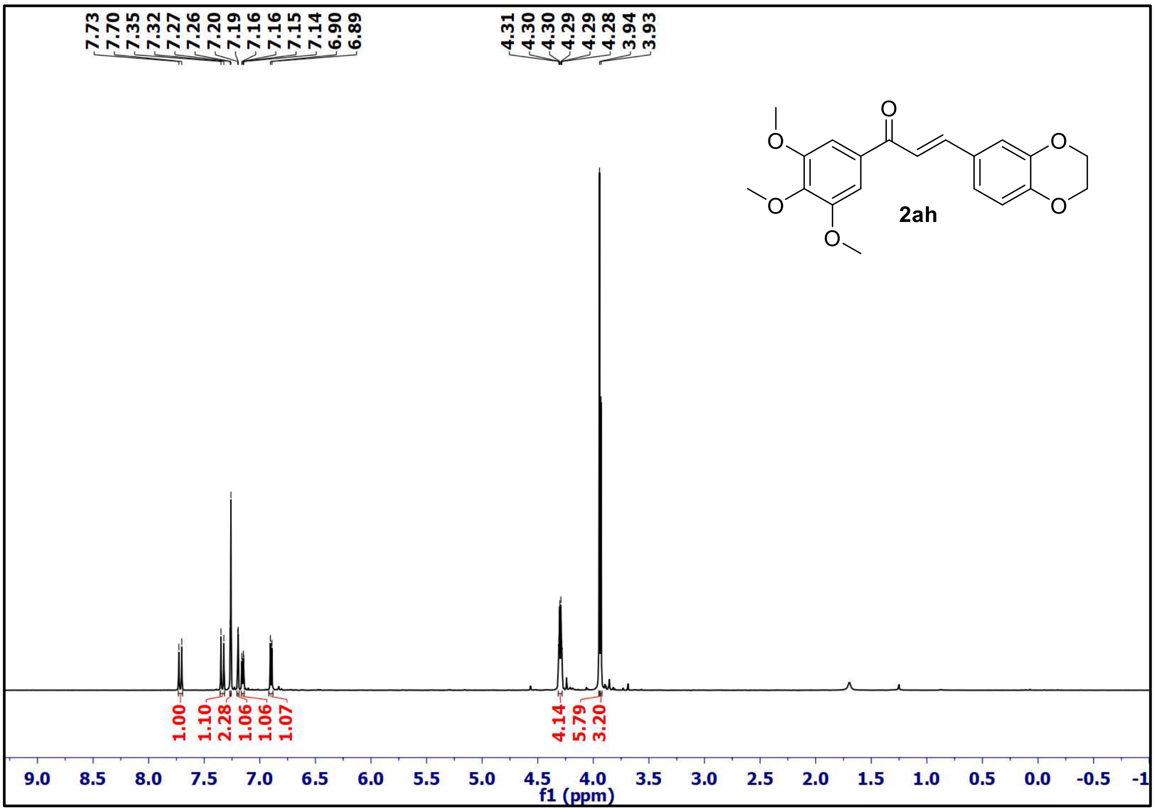

^{13}**C NMR** (150 MHz, $CDCl_3$)

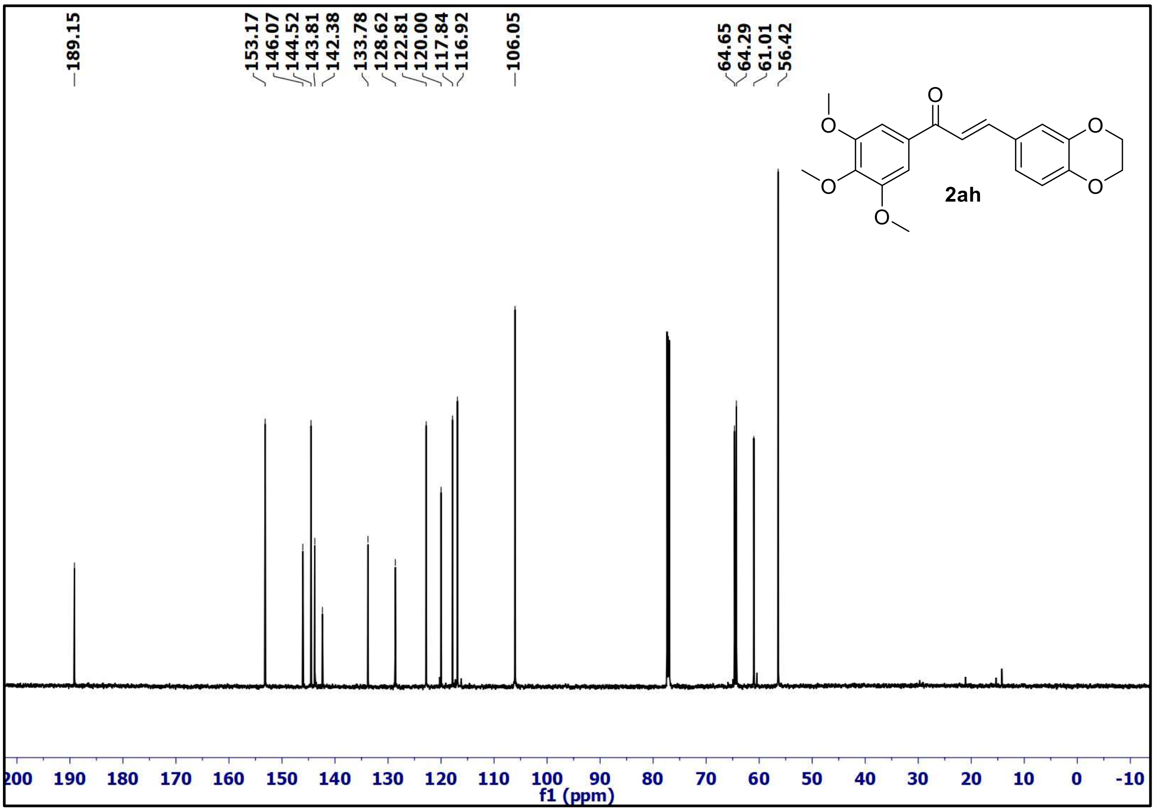

(*E*)-1-(2-Methoxyphenyl)-3-(2-(trifluoromethyl)phenyl)prop-2-en-1-one (2ai)

^{1}H NMR (600 MHz, $CDCl_3$)

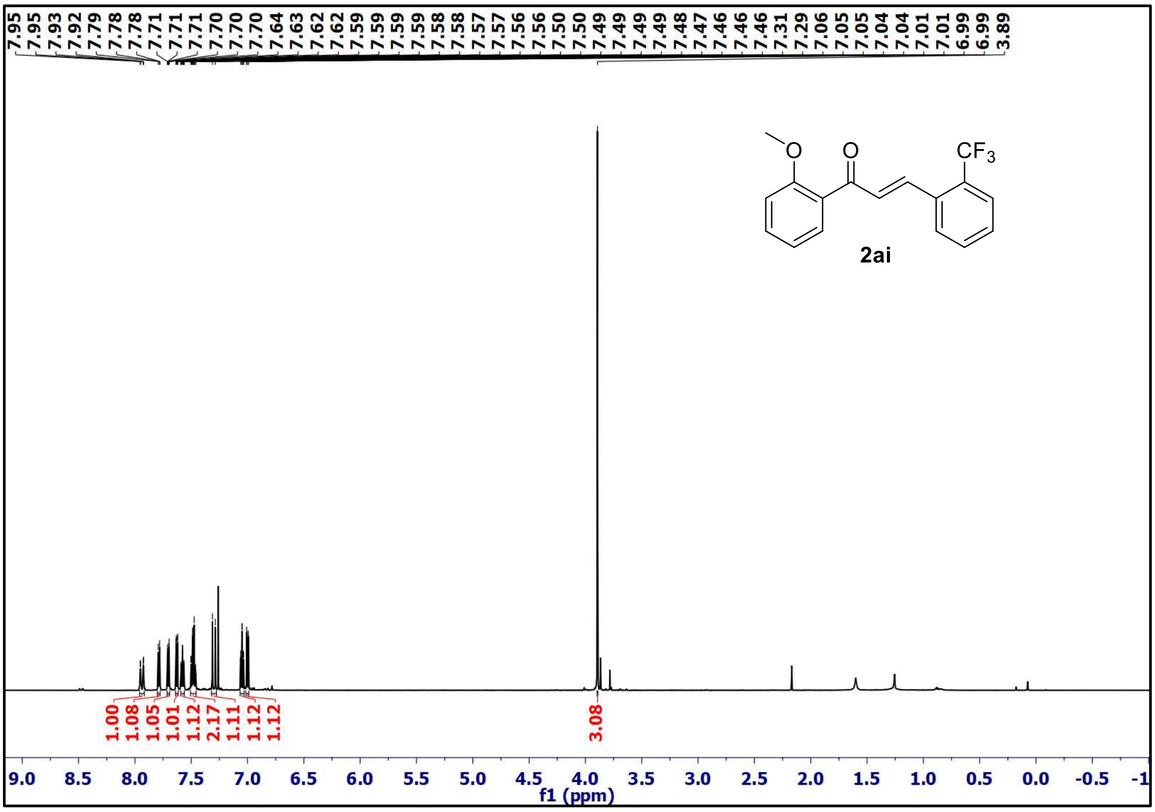

^{13}C NMR (150 MHz, $CDCl_3$)

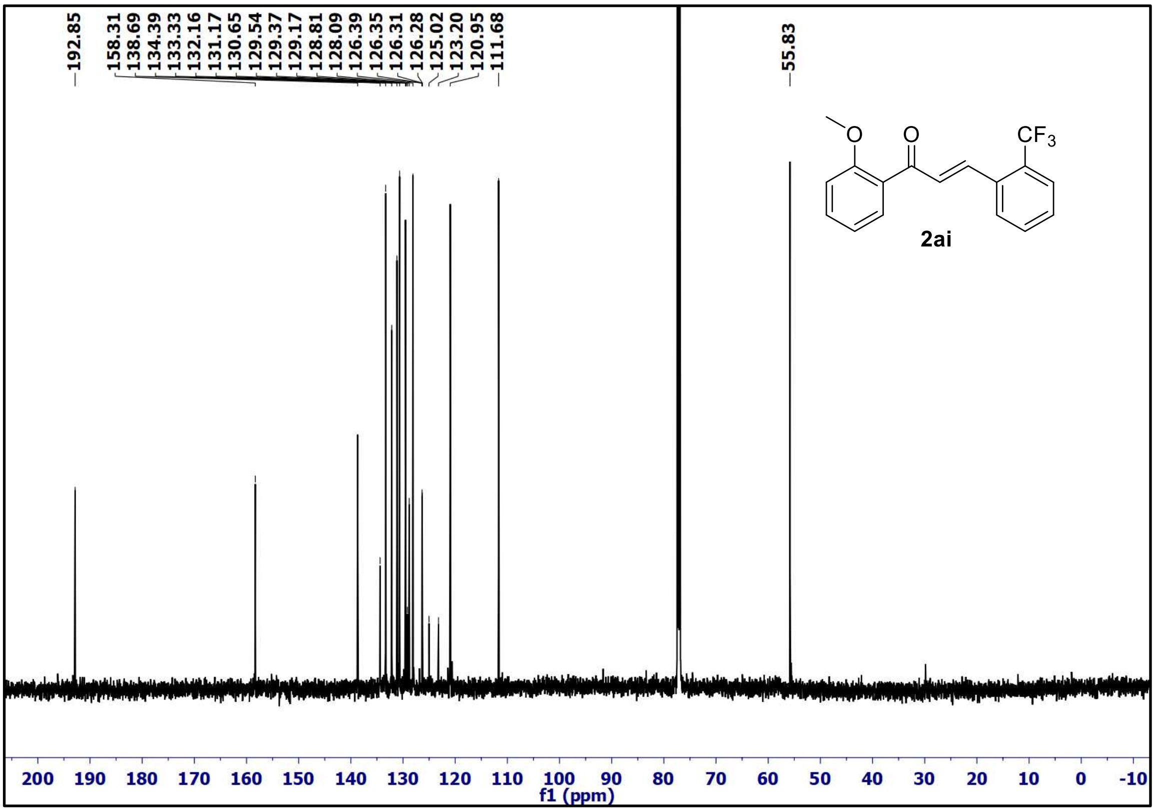

^{19}F NMR (150 MHz, $CDCl_3$)

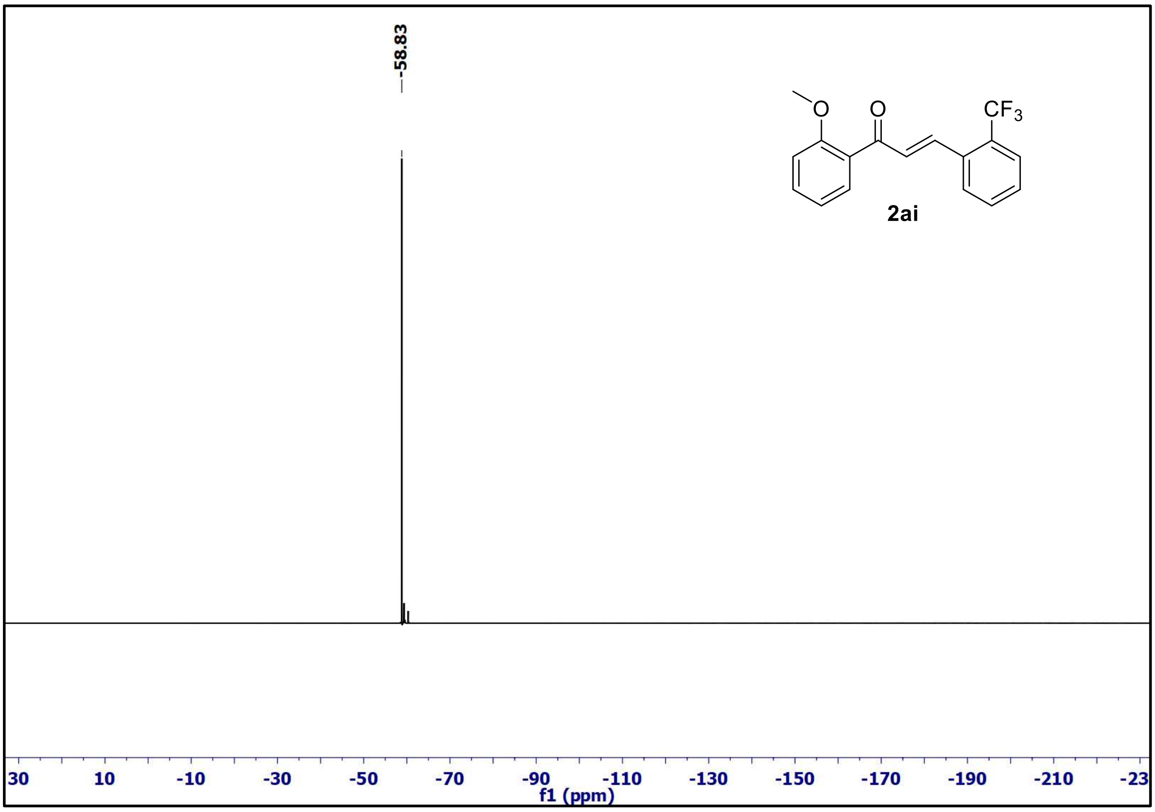

Substrate limitations

Table S2. Examples of unsuccessful alkenylation of amides using $LiCH_2SiMe_3$ and benzaldehyde under optimized conditions.[a]

Starting Material	Recovered compounds
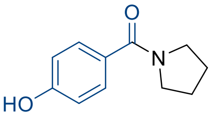	*Starting material* (75%) + 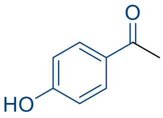 (25%)
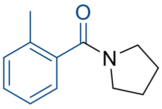	*Starting material* (74%)
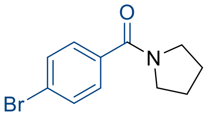	*Starting material* (37%) + 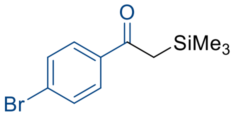 (40%) + 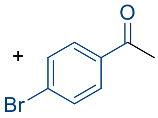 (20%)
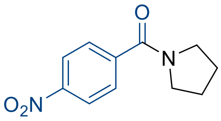	*Complex mixture*
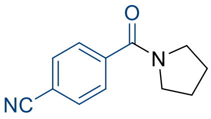	*Complex mixture*
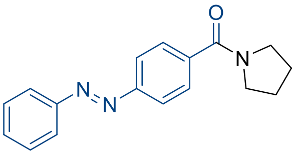	*Complex mixture*
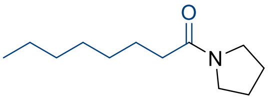	*Complex mixture*

[a] Reaction conditions: amide (0.2 mmol, 1.0 eq.), $LiCH_2SiMe_3$ (0.7 M in hexanes, 0.3 mmol, 1.5 eq.), CPME (5 mL), 30 min, RT, then PhCHO (0.24 mmol, 1.2 eq.), 1 h.

Electrophilic quench experiments

Table S3. Protonation and deuteration experiments on **1a**.[a]

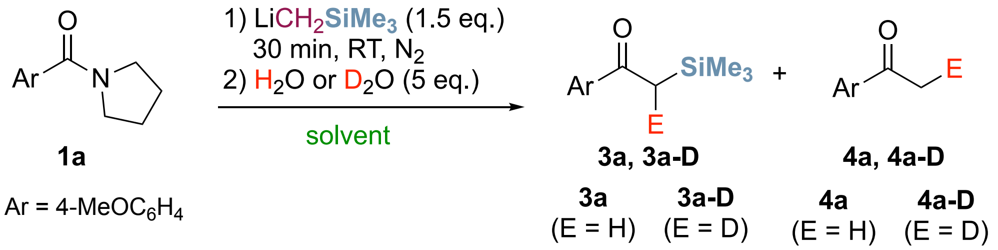

Entry	Solvent	$LiCH_2SiMe_3$ (eq.)	E^+	3a	3a-D	4a	4a-D
1	CPME	1.5	H_2O	100	0	0	0
2	CPME	1.5	D_2O	0	70 (40)	0	30 (60)
3	THF	1.5	H_2O	0	0	100	0
4	THF	1.5	D_2O	0	18 (51)	0	82 (131)[b]
5	CPME	2.0	D_2O	0	75 (73)	0	25 (65)
6	THF	2.0	D_2O	0	0	0	100 (170)[b]

[a] Reaction conditions: **1a** (0.2 mmol), $LiCH_2SiMe_3$ (0.7 M in hexanes, 0.3 mmol), CPME *or* THF (5 mL), 30 min, RT, then H_2O *or* D_2O. Ratios are based on ^1H NMR integration. Values in brackets refer to the overall D incorporation (%) based on ^1H NMR integration and confirmed with ^2H NMR. [b] As mixtures of isotopomers.

General procedure. In a flame-dried flask equipped with a magnetic stirrer, (4-methoxyphenyl)(pyrrolidin-1-yl)methanone **1a** (0.2 mmol, 1 eq., 41 mg) was dissolved in the selected dried solvent, then, the appropriate amount of $LiCH_2SiMe_3$ (0.7 M in hexanes) was slowly added. The reaction mixture was stirred at room temperature for 30 min, then the electrophile (5 eq.) was added. The mixture was stirred for 10 min and then quenched with H_2O (5 mL). After extraction with DEE (3 x 10 mL), the combined organic layers were dried over Na_2SO_4 and the solvent removed under reduced pressure at room temperature. The crude reaction mixtures were analysed by ^1H and ^2H NMR.

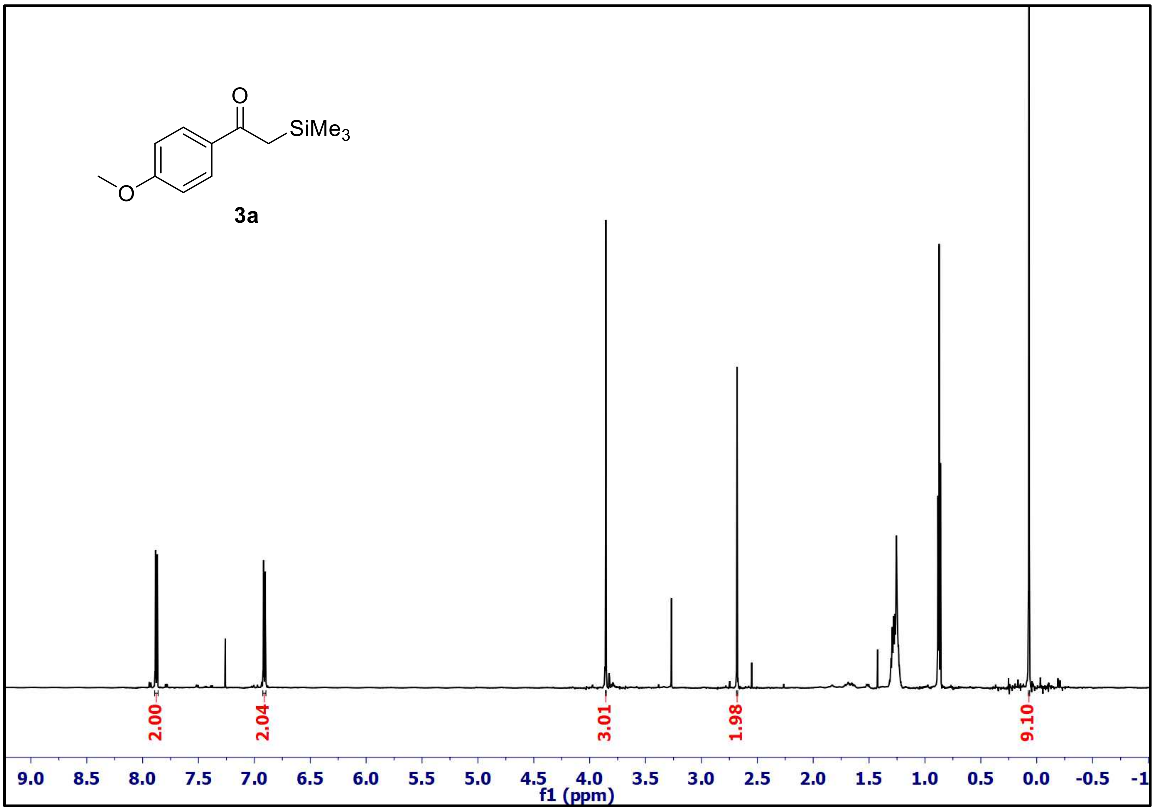

Figure S17: Table S3 entry 1: crude ^{1}H NMR spectrum of the reaction performed on **1a** (0.20 mmol, 1.0 eq.), CPME (5 mL), $LiCH_2SiMe_3$ (0.30 mmol, 1.5 eq.), quench with H_2O.

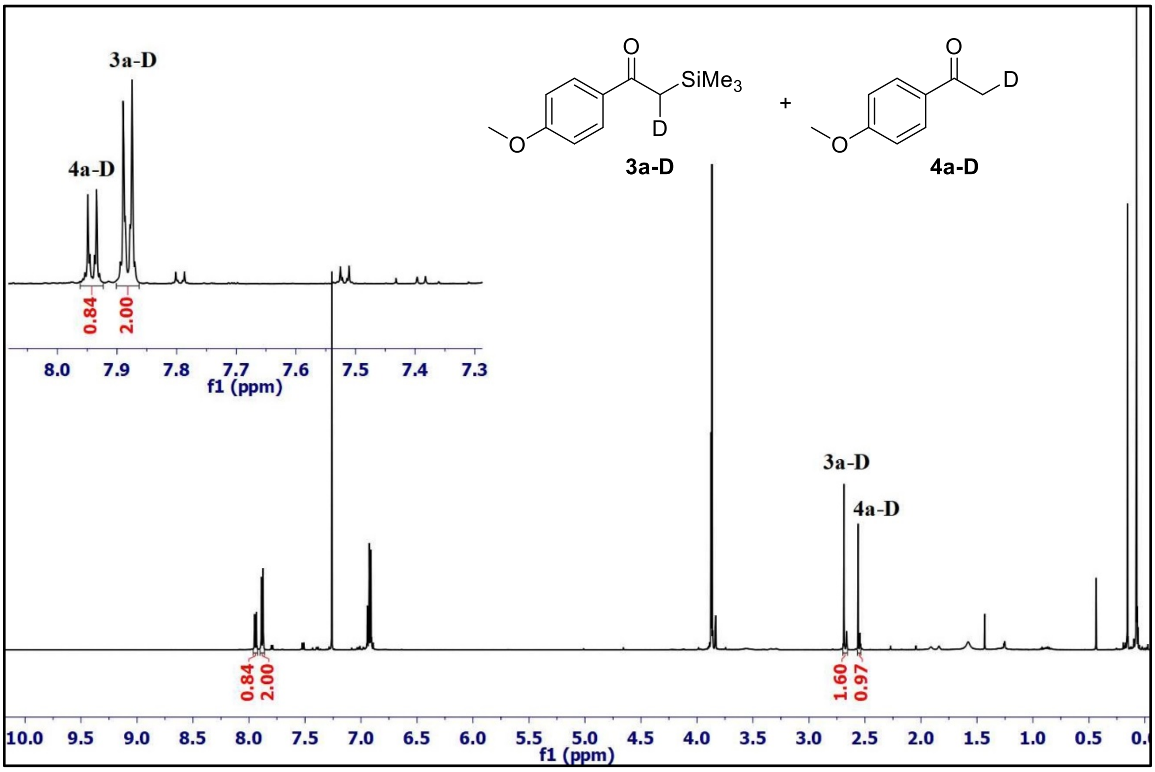

Figure S18: Table S3 entry 2: crude ^{1}H NMR spectrum of the reaction performed on **1a** (0.20 mmol, 1.0 eq.), CPME (5 mL), $LiCH_2SiMe_3$ (0.30 mmol, 1.5 eq.), quench with D_2O.

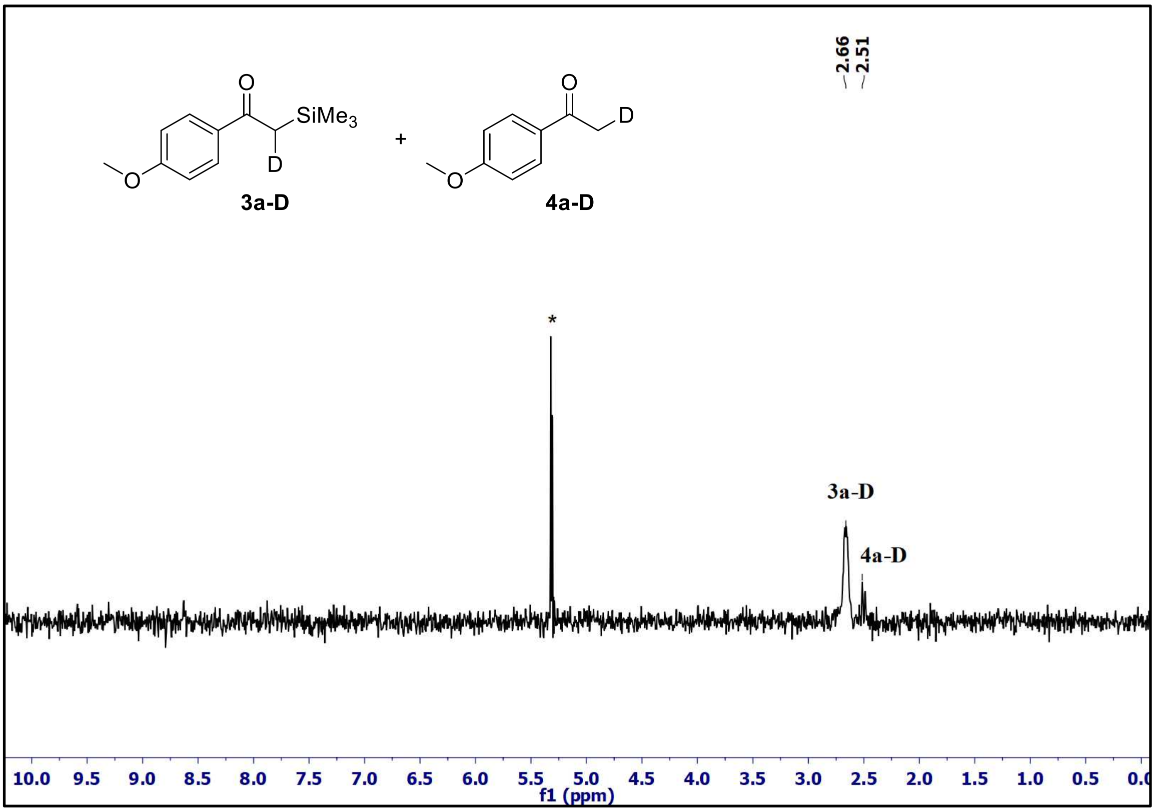

Figure S19: Table S3 entry 2: crude ^{2}H NMR spectrum of the reaction performed on **1a** (0.20 mmol, 1.0 eq.), CPME (5 mL), $LiCH_2SiMe_3$ (0.30 mmol, 1.5 eq.), quench with D_2O. * solvent residual peak

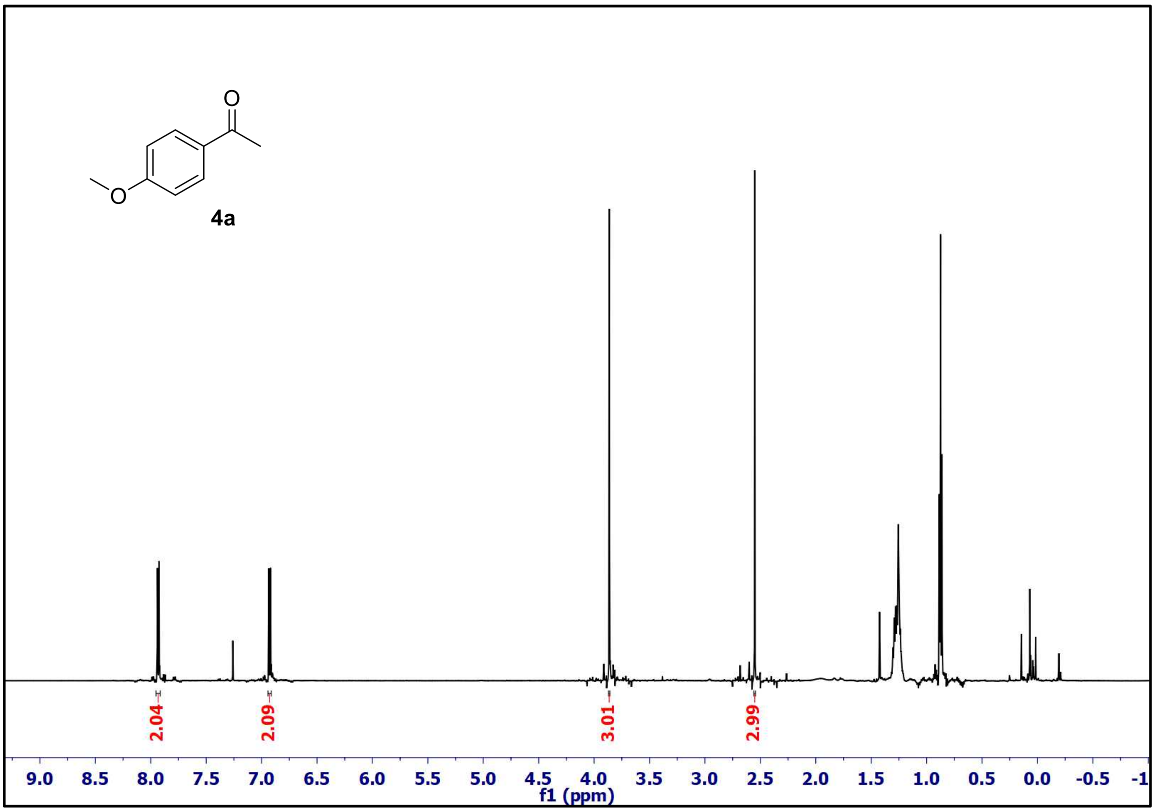

Figure S20: Table S3 entry 3: crude ^{1}H NMR spectrum of the reaction performed on **1a** (0.20 mmol, 1.0 eq.), THF (5 mL), $LiCH_2SiMe_3$ (0.30 mmol, 1.5 eq.), quench with H_2O.

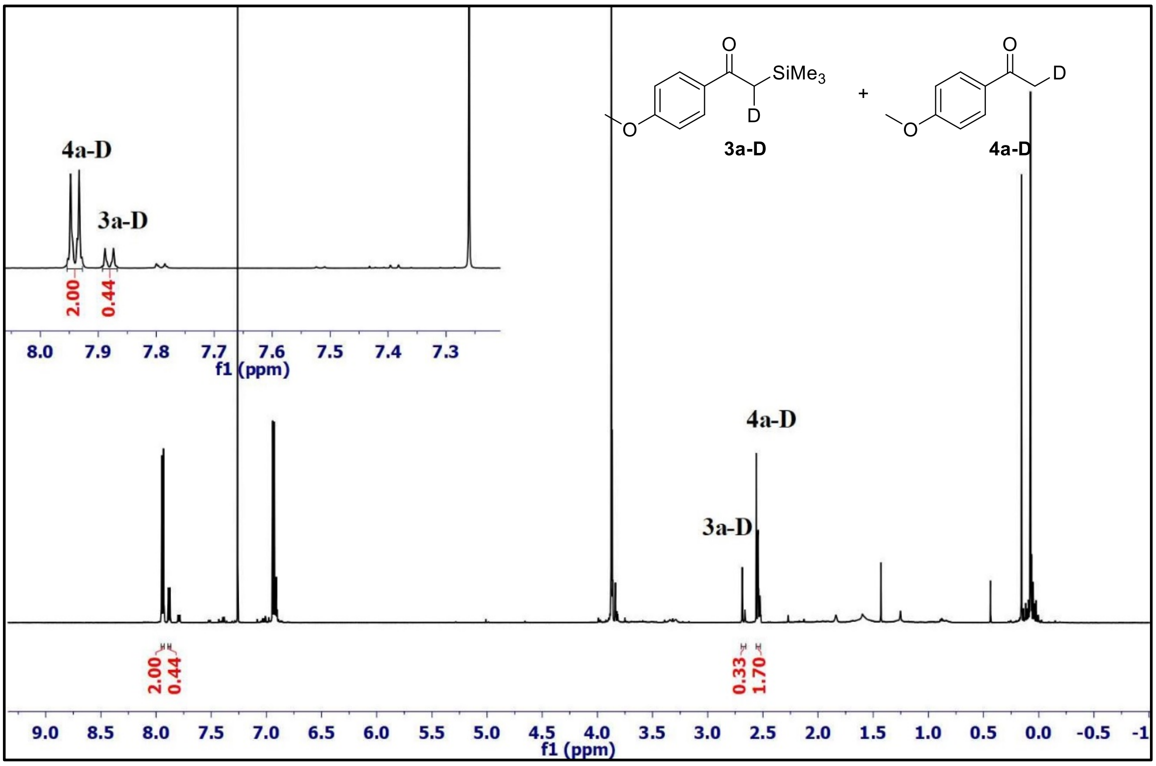

Figure S21: Table S3 entry 4: crude ^{1}H NMR spectrum of the reaction performed on **1a** (0.20 mmol, 1.0 eq.), THF (5 mL), $LiCH_2SiMe_3$ (0.30 mmol, 1.5 eq.), quench with D_2O.

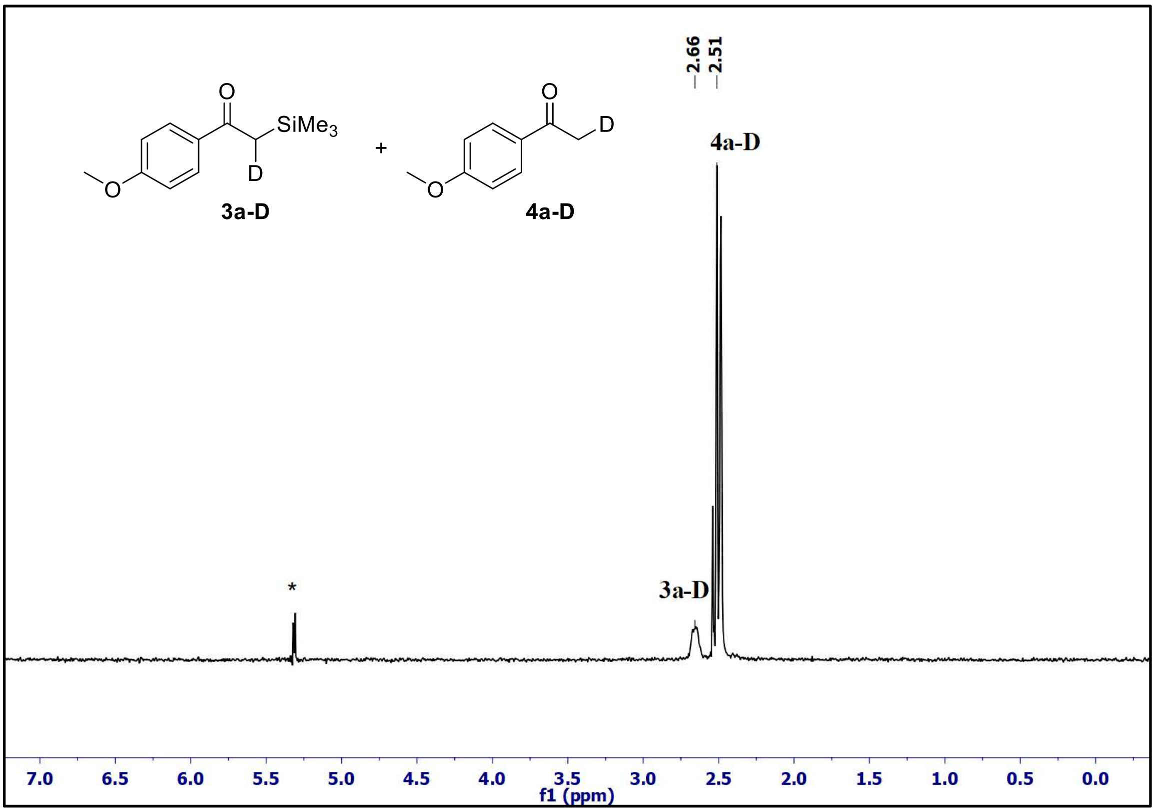

Figure S22: Table S3 entry 4: crude ^{2}H NMR spectrum of the reaction performed on **1a** (0.20 mmol, 1.0 eq.), THF (5 mL), $LiCH_2SiMe_3$ (0.30 mmol, 1.5 eq.), quench with D_2O. * solvent residual peak

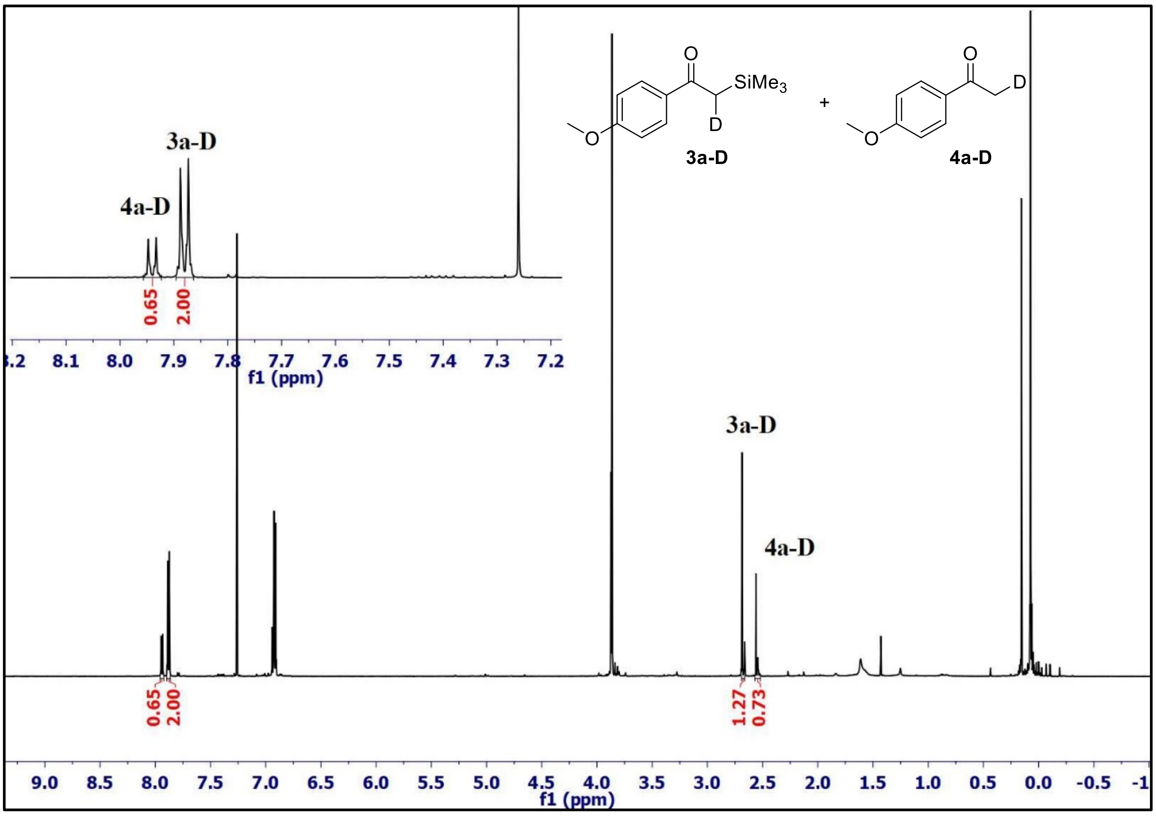

Figure S23: Table S3 entry 5: crude ^{1}H NMR spectrum of the reaction performed on **1a** (0.20 mmol, 1.0 eq.), CPME (5 mL), $LiCH_2SiMe_3$ (0.40 mmol, 2.0 eq.), quench with D_2O.

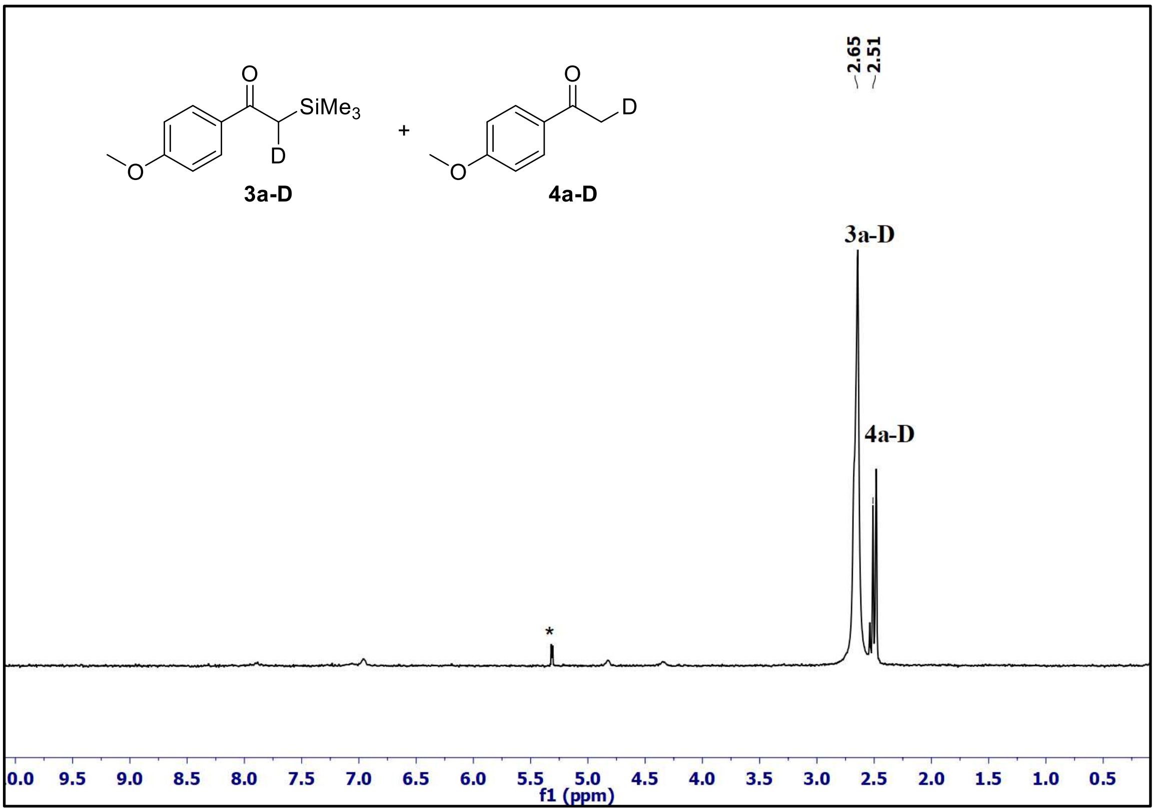

Figure S24: Table S3 entry 5: crude ^{2}H NMR spectrum of the reaction performed on **1a** (0.20 mmol, 1.0 eq.), CPME (5 mL), $LiCH_2SiMe_3$ (0.40 mmol, 2.0 eq.), quench with D_2O. * solvent residual peak

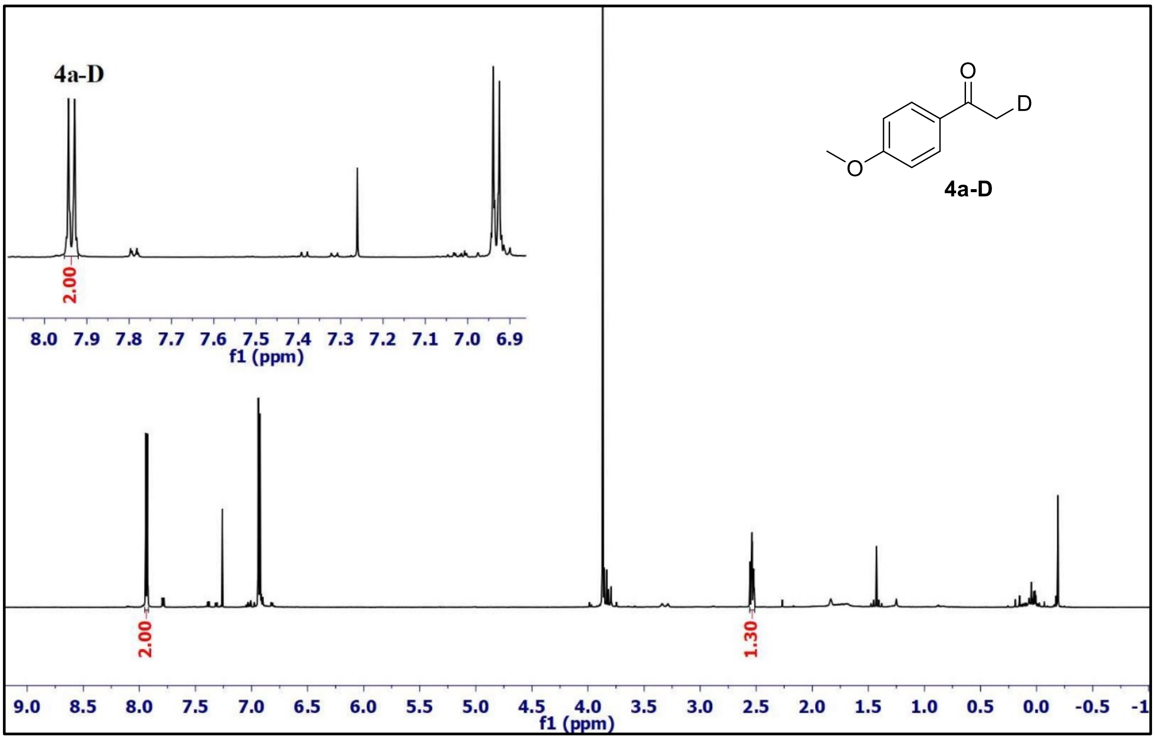

Figure S25: Table S3 entry 6: crude ^{1}H NMR spectrum of the reaction performed on **1a** (0.20 mmol, 1.0 eq.), THF (5 mL), $LiCH_2SiMe_3$ (0.40 mmol, 2.0 eq.), quench with D_2O.

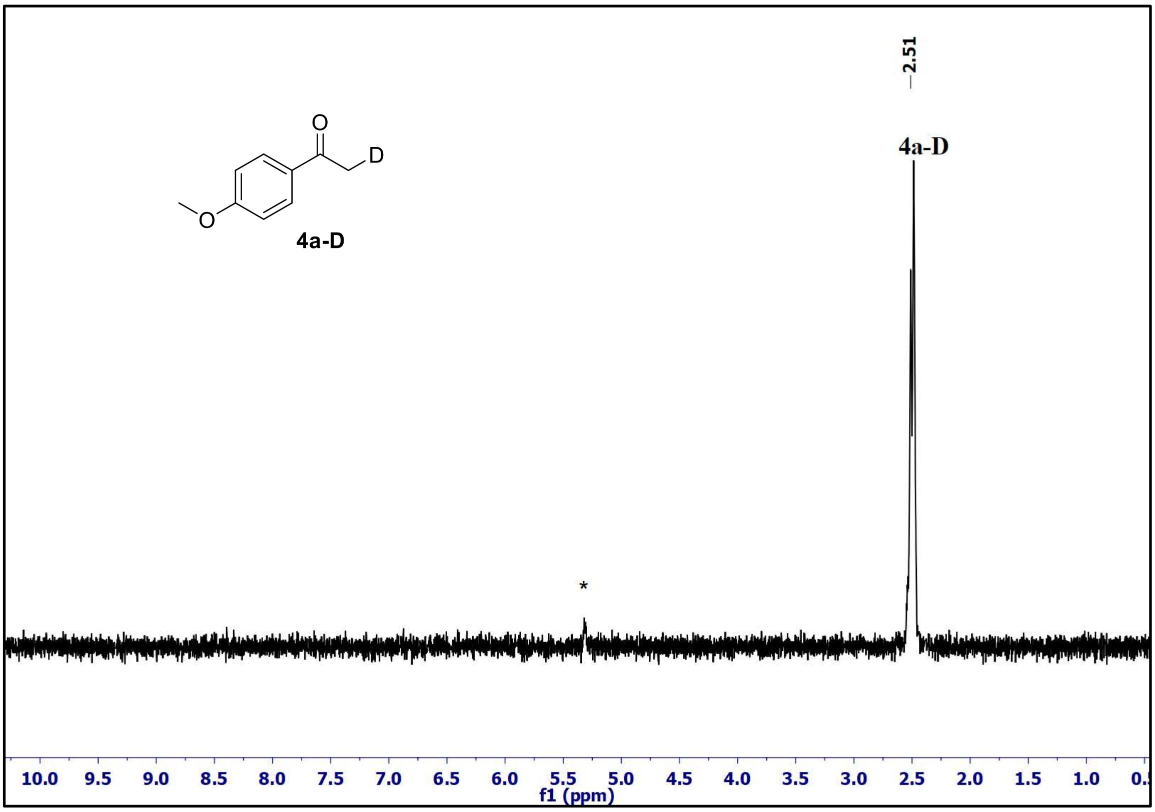

Figure S26: Table S3 entry 6: crude ^{2}H NMR spectrum of the reaction performed on **1a** (0.20 mmol, 1.0 eq.), THF (5 mL), $LiCH_2SiMe_3$ (0.40 mmol, 2.0 eq.), quench with D_2O. * solvent residual peak

In situ ^{13}C NMR monitoring of the S$_N$Ac reaction on ^{13}C-labelled **1b-^{13}C**

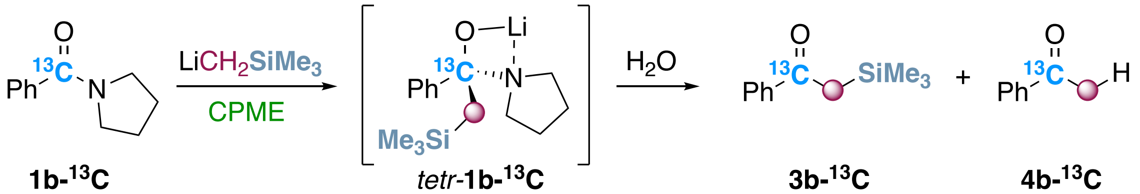

Synthesis and analysis of ^{13}C-labelled *N*-benzoylpyrrolidine **1b-^{13}C**

1-(^{13}C)Phenyl(pyrrolidin-1-yl)methanone (1b-^{13}C). To a stirred solution of 1-(^{13}C)benzoic acid (2.5 mmol, 1.0 eq., 0.3 g) in DCM (0.25 M, 10 mL), 1,1-carbonyldiimidazole (3 mmol, 1.2 eq., 0.49 g) was added at 0 °C. The mixture was allowed to warm to room temperature and stirred for 1 h, then pyrrolidine (3 mmol, 1.2 eq., 0.25 mL) was added. The solution was stirred for 18 hours at room temperature and quenched with 10 mL of aqueous 1M HCl. After extraction with DCM, the combined organic layers were washed with aqueous 1M HCl (3 x 10 mL) and aqueous 1M $NaHCO_3$ (3 x 10 mL), dried over Na_2SO_4 and evaporated under reduced pressure. Purification by flash column chromatography on silica gel (petroleum ether/acetone 8/2) gave **1b-^{13}C** as colorless oil (0.38 g, 72%, R_f = 0.28 petroleum ether/acetone). ^{1}H NMR (600 MHz, $CDCl_3$) δ 7.52-7.50 (m, 2H), 7.41-7.38 (m, 3H), 3.65 (t, *J* = 7.0 Hz, 2H), 3.42 (t, *J* = 6.7 Hz, 2H), 1.95 (quint, *J* = 6.6 Hz, 2H), 1.87 (quint, *J* = 6.8 Hz, 2H). ^{13}C{^{1}H} NMR (150 MHz, $CDCl_3$) δ 169.7, 137.2 (d, *J* = 64.9 Hz, 1C), 129.7, 128.2 (d, *J* = 4.3 Hz, 2C), 127.1, 49.6, 46.1, 26.4, 24.4. EI-MS *m/z* (%): 176 (M^+, 42), 175 (26), 147 (17), 106 (100), 77 (55). HRMS (ESI) *m/z*: $[M+Na]^+$ Calcd for $C_{10}{}^{13}C_1H_{13}NONa$ 199.0923; Found 199.0926.

^{1}H and ^{13}C NMR spectra of *N*-benzoylpyrrolidine **1b-^{13}C**

1-(^{13}C)Phenyl(pyrrolidin-1-yl)methanone (1b-^{13}C)

^{1}H NMR (600 MHz, $CDCl_3$)

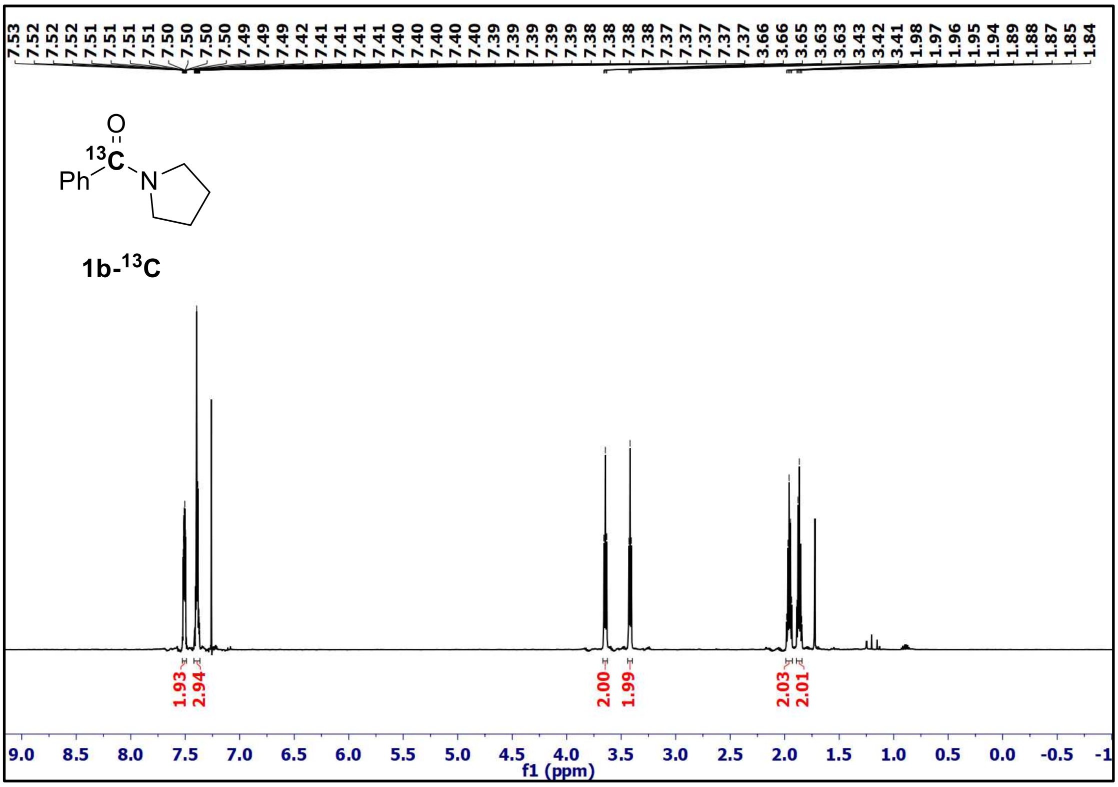

^{13}C NMR (150 MHZ, $CDCl_3$)

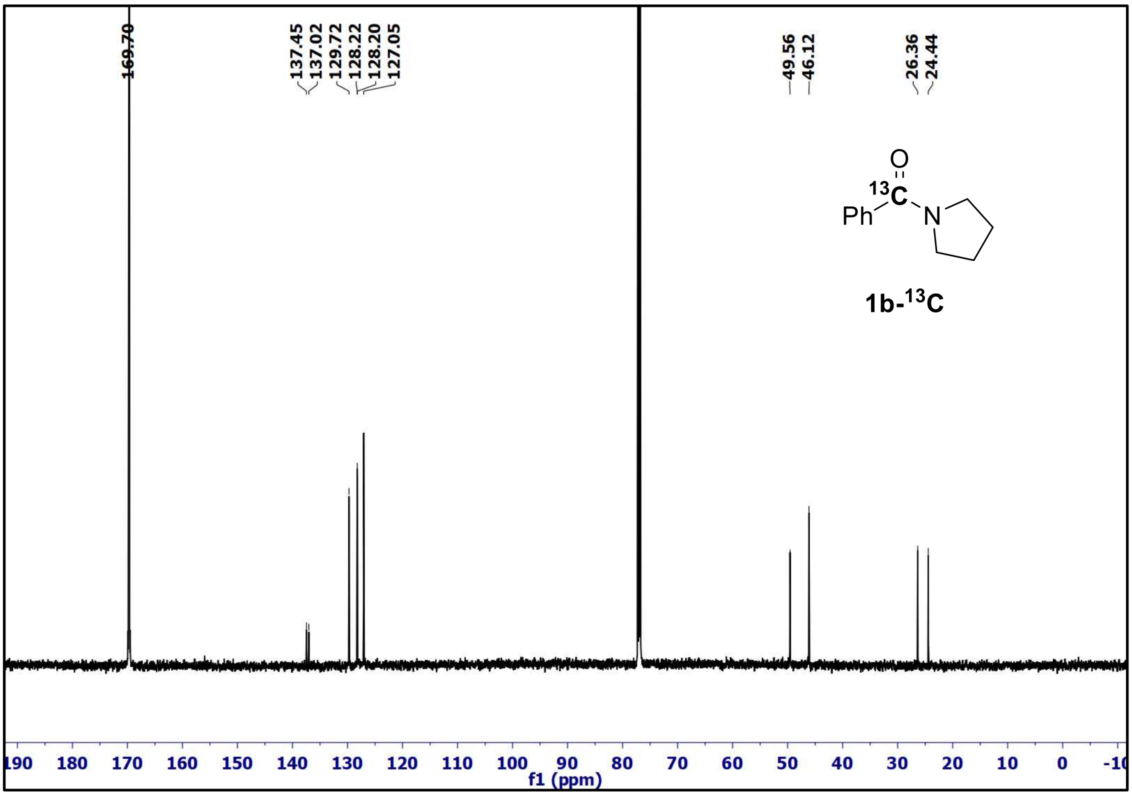

^{13}C{^{1}H} NMR spectra of *tetr*-**1b-^{13}C**

General procedure. In a nitrogen flushed NMR tube, **1b-^{13}C** (0.07 mmol, 1 eq., 12 mg) was dissolved in dry CPME. $LiCH_2SiMe_3$ (0.7 M in hexanes, 0.10 mmol, 1.5 eq., 0.14 mL) was added and the reaction was monitored by ^{13}C NMR. ^{1}H-decoupled ^{13}C NMR experiments were acquired after 30 min and 3 h (512 scans, relaxation delay = 2 s). The solution was quenched after 3 h with H_2O (1 mmol, 14 eq, 18 µL) and the ^{13}C{^{1}H} NMR spectrum was acquired. ^{13}C NMR spectra were calibrated on the CH_3OCH signal of CPME at 82.00 ppm.

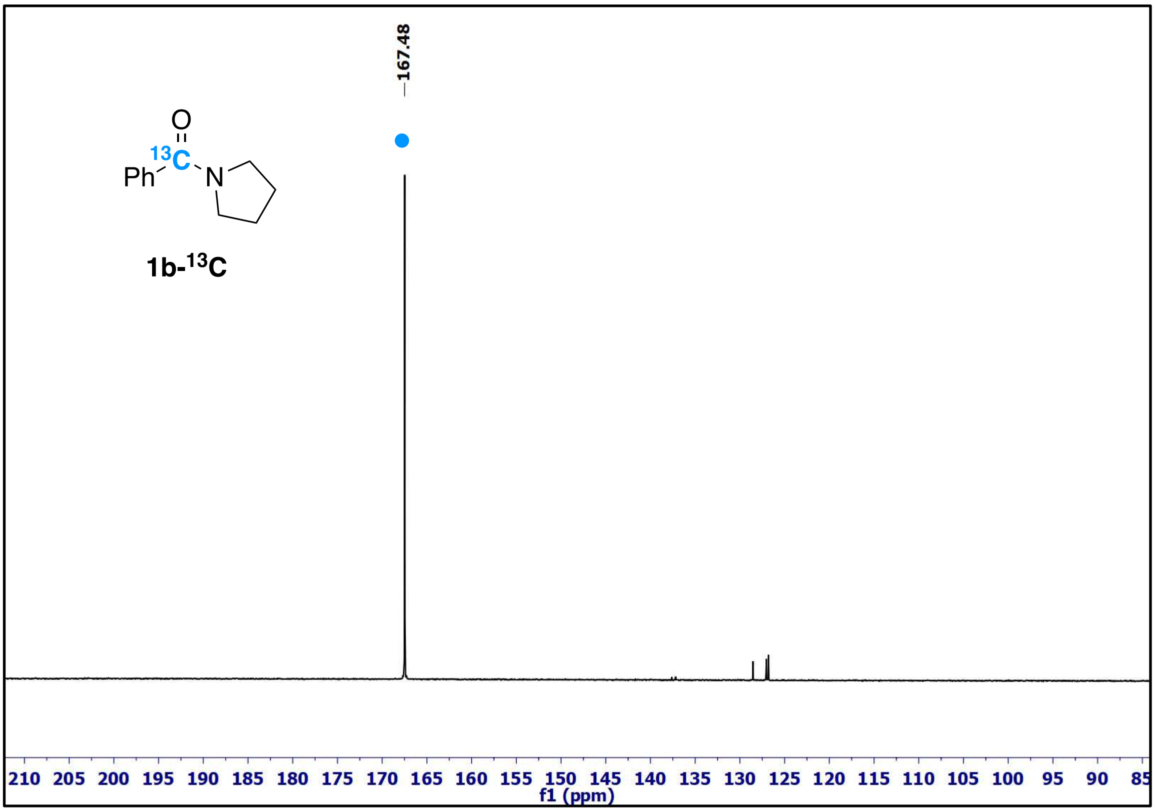

Figure S27: ^{13}C NMR spectrum of **1b-^{13}C** in CPME.

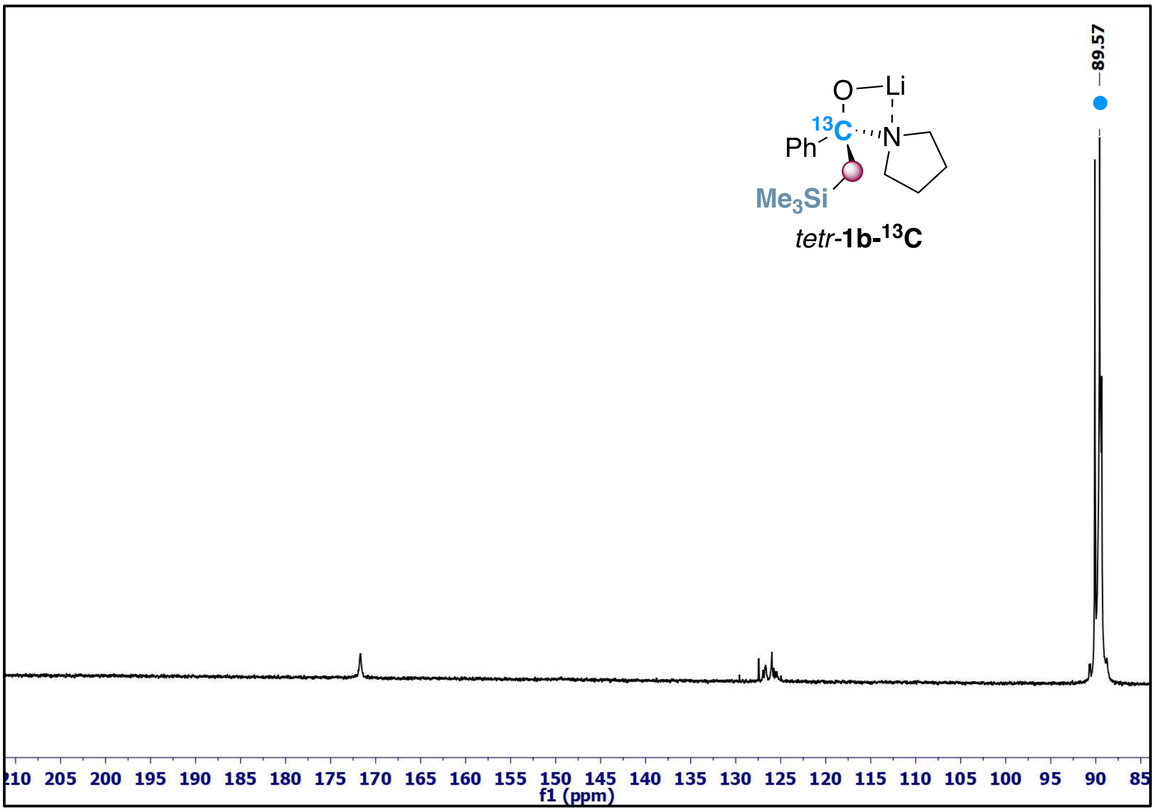

Figure S28: *in situ* ^{13}C NMR spectrum of *tetr*-**1b-^{13}C** in CPME after 30 min from the addition of $LiCH_2SiMe_3$ (0.1 mmol, 1.5 eq.).

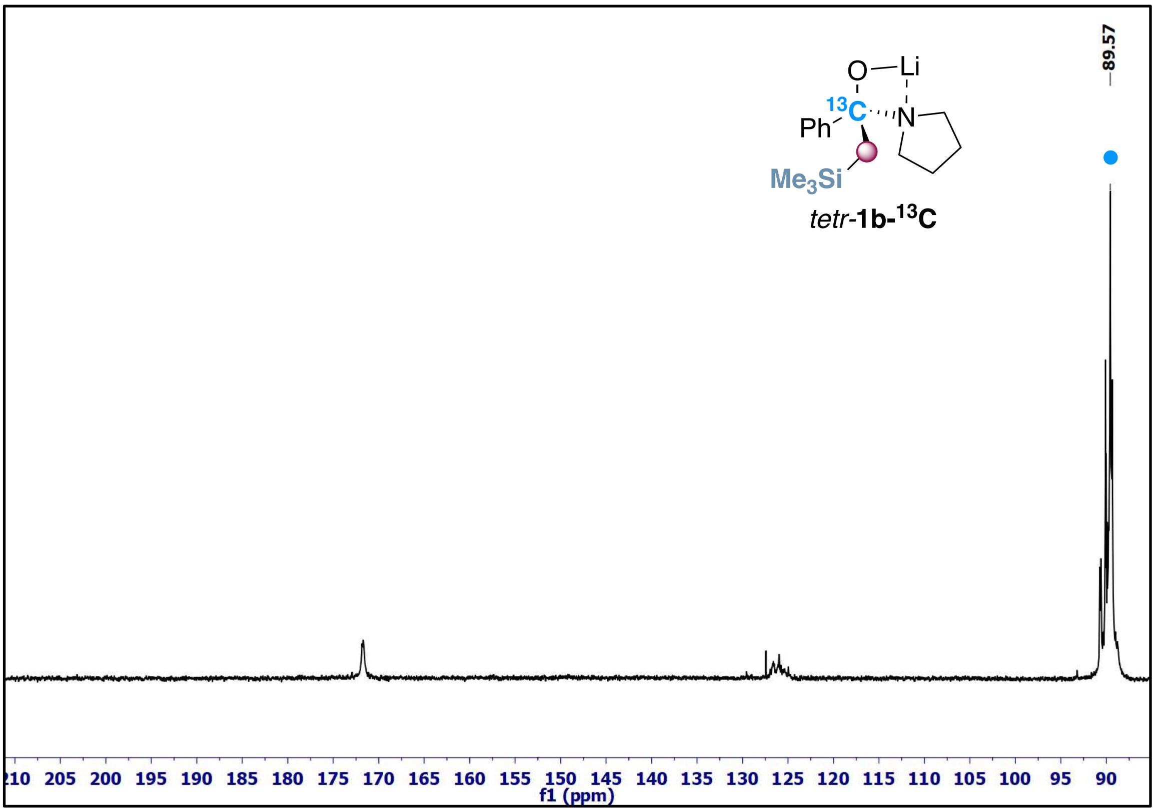

Figure S29: *in situ* ^{13}C NMR spectrum of *tetr*-**1b-^{13}C** in CPME after 3 h from the addition of $LiCH_2SiMe_3$ (0.1 mmol, 1.5 eq.).

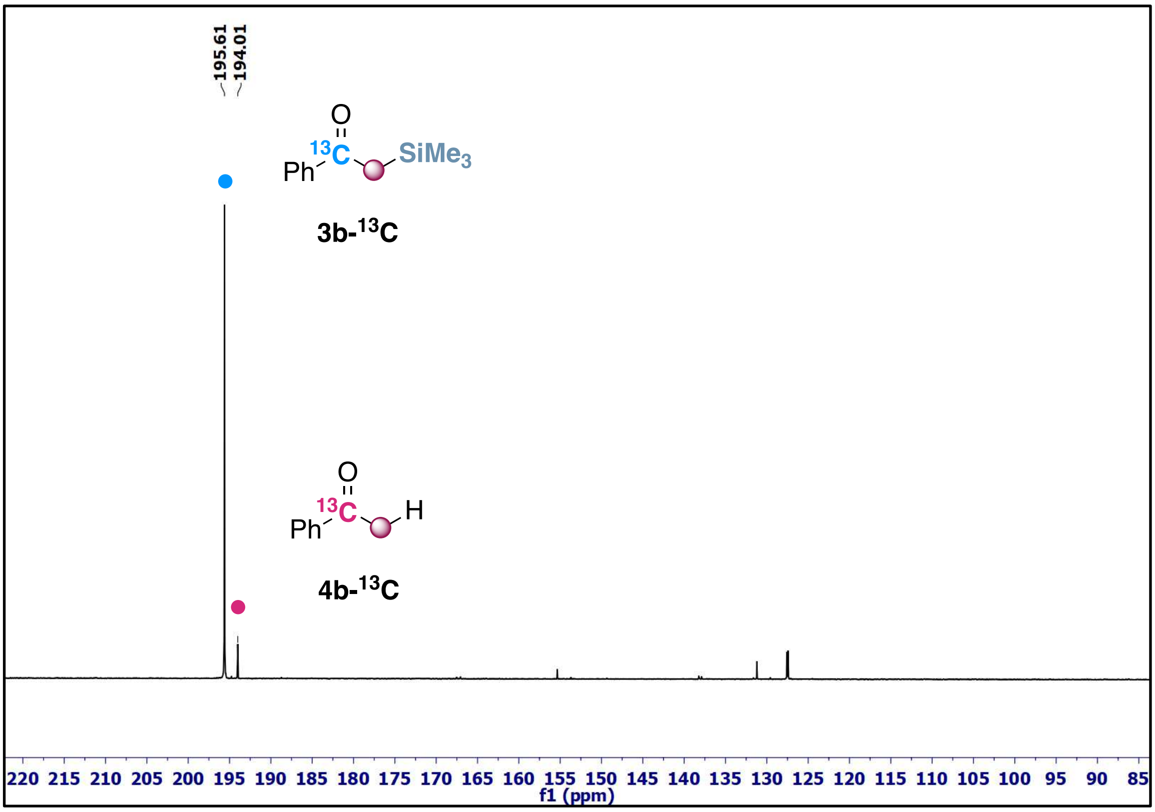

Figure S30: *in situ* ^{13}C NMR spectrum of the reaction mixture performed on **1b-^{13}C** (0.07 mmol, 1.0 eq.), CPME (0.3 mL), $LiCH_2SiMe_3$ (0.1 mmol, 1.5 eq.) after quench with H_2O.

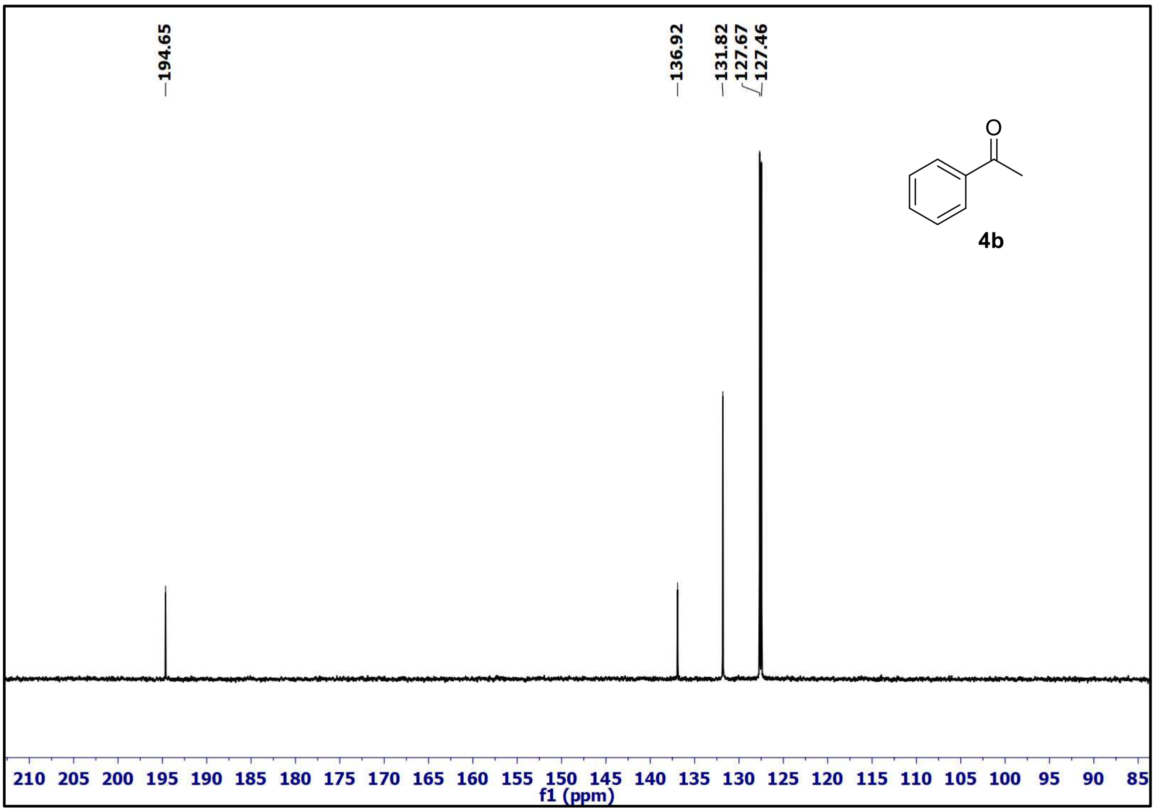

Figure S31: ^{13}C NMR spectrum of isolated acetophenone **4b** in CPME.

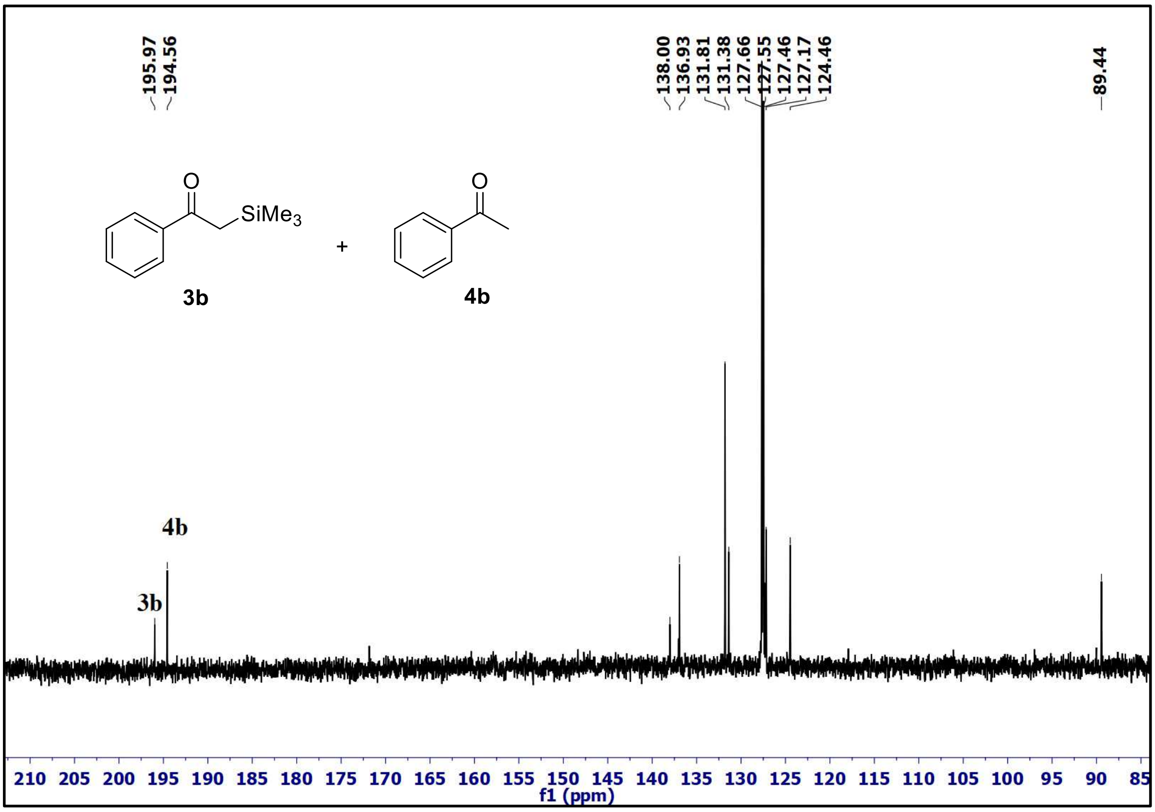

Figure S32: ^{13}C NMR spectrum of isolated α-trimethylsilylacetophenone **3b** in CPME. Protodesilylation into **4b** occurred in solution during the acquisition of the NMR experiment.[30]

Computational details

Method. Geometry optimizations and thermochemistry calculations were carried out by using the Gaussian 16 programs.[31] The stationary points on the energy hypersurface were optimized with DFT M06-2X functional.[32] The polarized 6-311+G(d) basis set was used in the DFT optimizations and in the vibrational analysis (both including solvent effects).[33,34] The molecules were considered as a solute in a polarized continuum (THF and CPME), within the Solvation Model based on Density (SMD)[35] and Integral Equation Formalism-Polarizable Continuum Model (IEF-PCM) schemes.[36] The Gibbs free energies (ΔG) were estimated at $T = 298$ K.

For a better energy assessment, the dimeric aggregates in solution and two explicit molecules of CPME or THF were used in the computations.[4] The parameters used for the non-standard CPME solvent are the following:

Static dielectric constant (ε) of the solvent = 4.76
Dynamic dielectric constant of the solvent (n^2) = 2.012
Hydrogen bond acidity = 0.0
Hydrogen bond basicity = 0.41
Surface tension at interface = 38.43
Carbon aromaticity = 0.0
Electronegative halogenicity = 0.0

a) **b)**

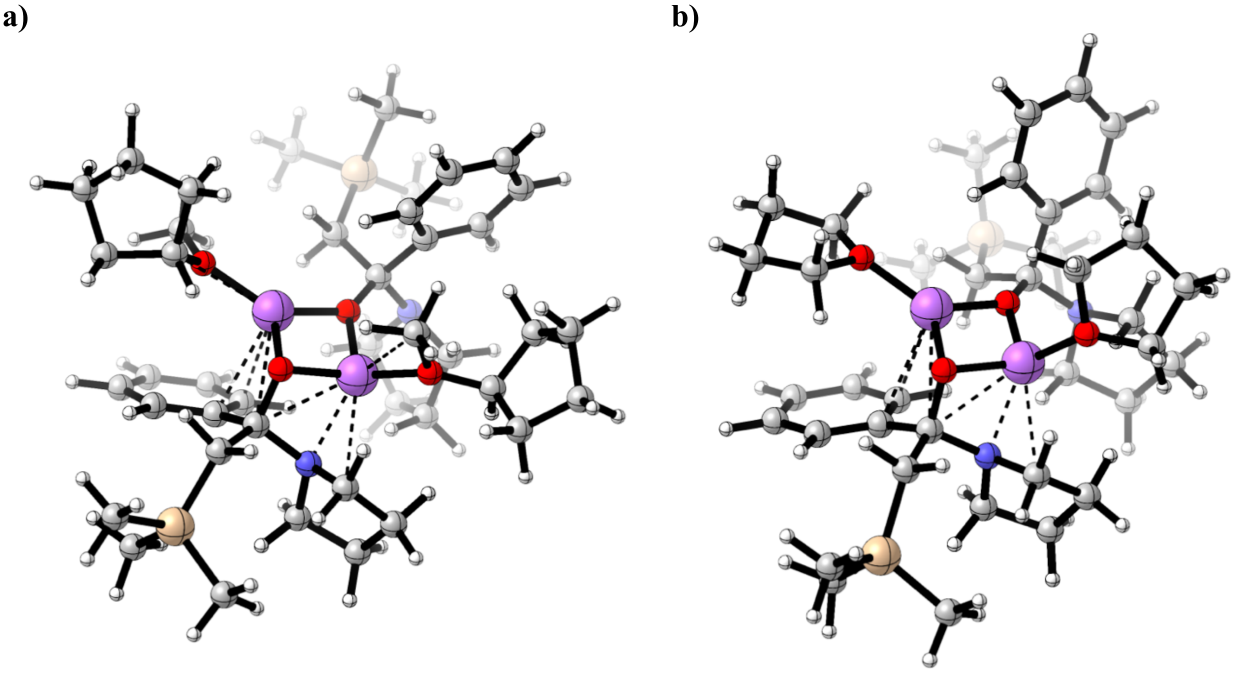

Figure S33. *anti tetr*-**1b** (a) in CPME, and (b) in THF. Structures were realized with CYLView20 (Legault, C. Y., Université de Sherbrooke, 2020 (http://www.cylview.org).

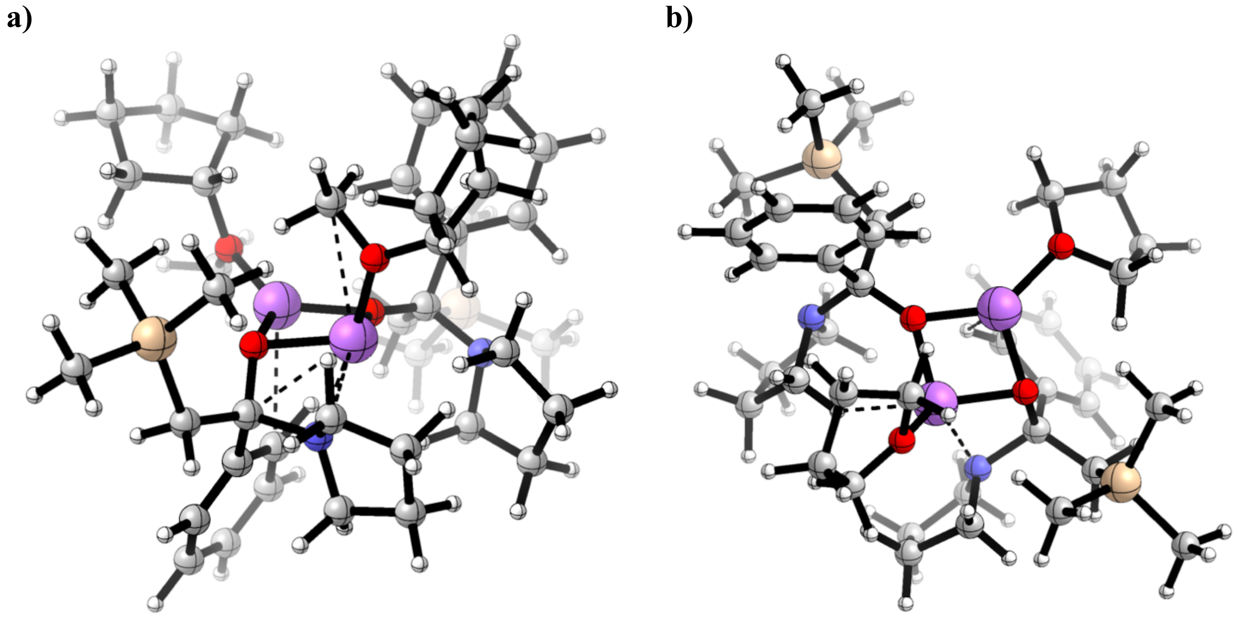

Figure S34. *syn tetr*-**1b** (a) in CPME, and (b) in THF. Structures were realized with CYLView20 (Legault, C. Y., Université de Sherbrooke, 2020 (http://www.cylview.org).

Energies and cartesian coordinates

Solvent: **CPME**

anti tetr-**1b**

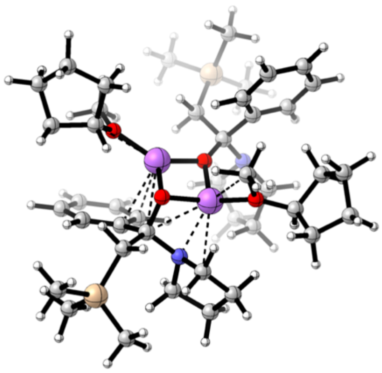

Atom	X	Y	Z	(Angstrom)
8	-1.05637	-0.08659	-0.30096	
8	1.50140	0.13204	0.89936	
3	0.26635	-1.14781	0.32609	
3	0.18584	1.22590	0.02557	
6	-2.35746	-0.27349	-0.69282	
6	2.57154	0.30501	0.04490	
6	-3.33116	0.27571	0.37227	
6	-4.52729	0.92477	0.05639	
6	-3.03219	0.06490	1.72020	
6	-5.39744	1.35380	1.05756	
1	-4.77630	1.10023	-0.98361	
6	-3.90360	0.47789	2.72479	
1	-2.09574	-0.42037	1.97815	

6	-5.09234	1.12852	2.39727
1	-6.31656	1.86480	0.78882
1	-3.65185	0.29950	3.76563
1	-5.76987	1.45810	3.17780
6	2.58004	-0.91150	-0.91295
6	1.60179	-1.00756	-1.91224
6	3.38730	-2.02999	-0.69388
6	1.44243	-2.16591	-2.66652
1	0.94221	-0.16470	-2.08818
6	3.23780	-3.19151	-1.45051
1	4.14614	-2.01865	0.07956
6	2.26462	-3.26797	-2.44014
1	0.67599	-2.20657	-3.43483
1	3.88606	-4.04085	-1.25885
1	2.14602	-4.17253	-3.02721
6	-2.64104	-1.79211	-0.83122
1	-2.30095	-2.24555	0.11087
1	-1.96684	-2.19310	-1.59760
6	3.88581	0.40036	0.86282
1	3.91796	-0.50656	1.47698
1	3.73581	1.19902	1.59864
8	0.51359	-2.98012	0.77138
6	-0.06223	-4.14564	0.21095
1	-0.55529	-3.84467	-0.71243
1	0.70159	-4.88986	-0.02797
1	-0.80558	-4.59267	0.87924
6	1.23190	-3.13058	2.00972
6	0.28462	-3.39950	3.20324
6	2.24699	-4.28907	2.01659
1	1.73726	-2.16875	2.10695
6	0.86547	-4.61762	3.94013
1	-0.71463	-3.62691	2.82135
1	0.18812	-2.52145	3.84420
6	1.58676	-5.40175	2.83780
1	2.55057	-4.58110	1.01018
1	3.14659	-3.94544	2.53681
1	1.59426	-4.29194	4.68923
1	0.10047	-5.20102	4.45640
1	2.30407	-6.12729	3.22680
1	0.85750	-5.94683	2.22930
8	-0.46249	2.76208	1.05523
6	-0.44729	2.37649	2.42253
1	0.15838	1.47054	2.47792
1	0.01065	3.14549	3.05205
1	-1.45455	2.16304	2.78723
6	-1.16728	3.96880	0.73457
6	-2.62173	3.98002	1.24500
6	-0.50109	5.23477	1.32388
1	-1.14098	3.99245	-0.35501
6	-2.61436	4.91017	2.46289
1	-2.98934	2.97091	1.44410
1	-3.25930	4.40807	0.46468
6	-1.62128	6.00258	2.04786
1	0.28215	4.94216	2.02741
1	-0.01976	5.82923	0.54530
1	-3.60445	5.30215	2.70466
1	-2.24150	4.38138	3.34617
1	-2.11159	6.69201	1.35312
1	-1.25108	6.59220	2.88908
6	2.49141	2.80734	0.00175
6	2.97213	1.79857	-2.01306
6	2.10736	3.86307	-1.02782
1	3.54832	2.93248	0.27200
1	1.89600	2.83445	0.91345

6	2.65388	3.27241	-2.34741
1	4.05597	1.66467	-1.90551
1	2.63474	1.11363	-2.78985
1	1.01913	3.95752	-1.07074
1	2.52250	4.84538	-0.79491
1	1.91962	3.34624	-3.15167
1	3.55320	3.79422	-2.68009
7	2.27608	1.55473	-0.74145
6	-2.34221	1.84986	-1.92194
6	-1.90742	-0.16389	-3.11847
6	-1.18759	2.08537	-2.89684
1	-2.09712	2.13825	-0.89998
1	-3.21751	2.43611	-2.22956
6	-1.47367	1.03107	-3.96727
1	-1.01488	-0.68800	-2.76207
1	-2.51922	-0.87850	-3.67739
1	-1.15260	3.10739	-3.28154
1	-0.22765	1.86901	-2.41160
1	-2.29282	1.36531	-4.61206
1	-0.61426	0.79918	-4.60059
7	-2.65738	0.40305	-1.98184
14	5.65325	0.62242	0.19720
6	6.08389	-0.32012	-1.37831
1	7.05758	0.02733	-1.74224
1	6.17178	-1.39437	-1.19475
1	5.36218	-0.18426	-2.18741
6	6.76996	-0.04031	1.56354
1	6.59806	0.48295	2.50991
1	6.59320	-1.10628	1.74109
1	7.82782	0.07979	1.30841
6	6.13148	2.43129	-0.04491
1	7.21239	2.49337	-0.21388
1	5.64251	2.91336	-0.89528
1	5.90970	3.02574	0.84736
14	-4.36325	-2.51418	-1.12408
6	-5.35456	-1.69986	-2.49789
1	-6.26509	-2.27729	-2.69238
1	-5.65603	-0.68098	-2.24362
1	-4.78808	-1.65522	-3.43282
6	-4.06045	-4.31234	-1.60848
1	-3.50619	-4.38508	-2.55011
1	-3.47764	-4.83662	-0.84335
1	-5.00008	-4.85945	-1.73689
6	-5.37192	-2.52321	0.46643
1	-6.29347	-3.10005	0.33235
1	-4.80917	-2.99204	1.28082
1	-5.65458	-1.51909	0.79393

E= -2648.094362 (Hartree)

syn tetr-**1b**

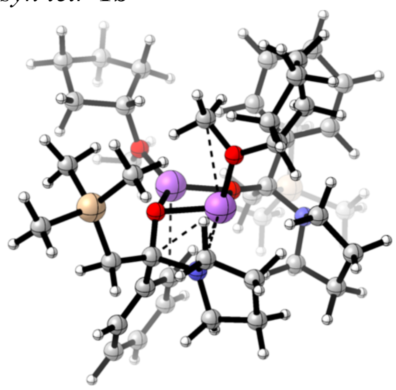

Atom X Y Z (Angstrom)

8	0.71438	-0.14329	-0.30697
8	-1.99704	0.57889	-0.20842
3	-0.33446	1.33653	-0.20078
3	-0.88914	-0.96655	-0.02095
6	2.01213	-0.50609	-0.54622
6	-2.78764	0.11078	-1.22745
6	2.80362	-0.57244	0.77787
6	3.74422	-1.55874	1.07688
6	2.59341	0.44425	1.71305
6	4.43470	-1.54529	2.28884
1	3.93107	-2.34940	0.35920
6	3.29028	0.47359	2.91663
1	1.86130	1.21332	1.48619
6	4.21110	-0.53059	3.21452
1	5.15044	-2.33115	2.50822
1	3.11319	1.27535	3.62714
1	4.74989	-0.51848	4.15597
6	-2.36699	0.71357	-2.59430
6	-1.00597	0.88023	-2.87169
6	-3.27869	1.04919	-3.59742
6	-0.57191	1.38707	-4.09304
1	-0.26774	0.55492	-2.14398
6	-2.85039	1.56014	-4.82264
1	-4.34154	0.90477	-3.44038
6	-1.49481	1.73855	-5.07562
1	0.49219	1.49871	-4.28118
1	-3.58287	1.81678	-5.58106
1	-1.16117	2.13712	-6.02771
6	2.69245	0.57456	-1.42787
1	2.53964	1.52251	-0.89388
1	2.11313	0.67225	-2.35399
6	-4.27272	0.42621	-0.92078
1	-4.96852	-0.29820	-1.36095
1	-4.52522	1.38395	-1.39308
6	-3.23181	-2.25889	-0.40865
6	-2.70502	-2.04921	-2.63576
6	-2.76238	-3.66164	-0.81373
1	-4.32111	-2.17804	-0.54302
1	-2.99690	-2.01079	0.62412
6	-2.42237	-3.52254	-2.31684
1	-3.74088	-1.92067	-2.99283
1	-2.03504	-1.64969	-3.39625
1	-1.87333	-3.94575	-0.24482
1	-3.52945	-4.41292	-0.61884
1	-1.36612	-3.74134	-2.49264
1	-3.00828	-4.19246	-2.94811
7	-2.51253	-1.38105	-1.34347
6	1.36815	-2.89835	-0.57603
6	1.65639	-1.88686	-2.59989
6	1.54797	-4.07536	-1.53669
1	0.29331	-2.65156	-0.52136
1	1.71542	-3.09195	0.44011
6	1.67274	-3.39046	-2.92005
1	0.62989	-1.49068	-2.68421
1	2.29935	-1.31294	-3.26863
1	2.46051	-4.62469	-1.29537
1	0.71313	-4.77777	-1.48242
1	2.61112	-3.66852	-3.40390
1	0.86123	-3.66172	-3.59791
7	2.12445	-1.82467	-1.21521
14	-4.69107	0.80285	0.88585
6	-4.39335	-0.52421	2.18744
1	-4.73975	-0.14530	3.15616
1	-4.94722	-1.44525	1.98225

1	-3.33721	-0.77704	2.28500
6	-6.55612	1.12047	0.88029
1	-6.91718	1.39908	1.87590
1	-6.82594	1.93256	0.19687
1	-7.11584	0.23244	0.56772
6	-3.85375	2.38850	1.45966
1	-2.80855	2.19528	1.70785
1	-3.86430	3.15367	0.67685
1	-4.35527	2.80454	2.34057
14	4.53623	0.52856	-1.84964
6	5.19853	-1.14936	-2.37958
1	4.63353	-1.56494	-3.21899
1	6.24069	-1.04907	-2.70296
1	5.16964	-1.87989	-1.56855
6	4.75601	1.72129	-3.29418
1	4.37015	2.71740	-3.05219
1	5.80997	1.83522	-3.56740
1	4.22251	1.37135	-4.18430
6	5.55425	1.18362	-0.40685
1	6.60110	1.31565	-0.70091
1	5.18127	2.16015	-0.07918
1	5.53422	0.51679	0.45950
8	-0.97609	-1.54217	1.86979
6	-0.56697	-2.77764	2.47412
6	-0.83588	-0.37436	2.66261
6	-1.53830	-3.23160	3.59049
6	0.82739	-2.72919	3.12939
1	-0.57443	-3.47720	1.63734
1	0.21627	-0.13944	2.84103
1	-1.30325	0.43239	2.09631
1	-1.35469	-0.47420	3.62161
6	-0.66370	-3.53660	4.81980
1	-2.23883	-2.42034	3.80656
1	-2.13759	-4.08782	3.27552
6	0.56503	-2.63676	4.63590
1	1.33953	-3.67255	2.91354
1	1.44848	-1.92861	2.72361
1	-0.34864	-4.58483	4.80563
1	-1.18859	-3.36526	5.76183
1	1.42154	-2.95439	5.23406
1	0.32612	-1.60746	4.92228
8	0.29708	3.05824	0.30547
6	0.17246	3.50897	1.66569
6	0.96521	3.91654	-0.60488
6	-0.79197	4.70941	1.80881
6	1.50415	3.96708	2.29119
1	-0.21429	2.62638	2.18072
1	2.02225	4.02759	-0.34804
1	0.88755	3.45464	-1.58988
1	0.49559	4.90295	-0.64550
6	-0.01775	5.78934	2.58529
1	-1.06837	5.06667	0.81378
1	-1.71961	4.41855	2.30343
6	1.45404	5.49854	2.27007
1	1.52439	3.62506	3.33060
1	2.37249	3.53135	1.79207
1	-0.18714	5.67179	3.66020
1	-0.32474	6.80129	2.31440
1	2.14264	5.95391	2.98432
1	1.70895	5.87864	1.27592

E= -2648.092594 (Hartree)

3b (from *anti tetr*-**1b**)

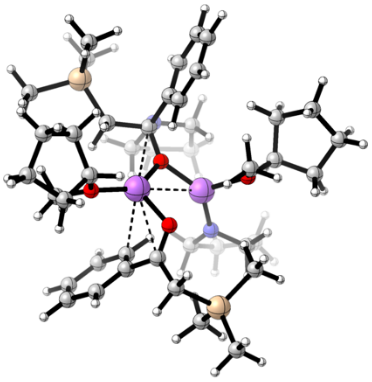

Atom	X	Y	Z	(Angstrom)
8	-0.56398	0.45567	-0.33263	
8	1.25837	-1.50198	1.14900	
3	-0.40014	-1.21513	0.24630	
3	1.18429	1.01460	-0.27960	
6	-1.78097	1.04001	-0.62089	
6	1.91958	-2.48144	0.79995	
6	-2.25384	1.90509	0.56557	
6	-2.96820	3.09454	0.40487	
6	-2.00812	1.45664	1.86514	
6	-3.40006	3.82752	1.50901	
1	-3.18731	3.45130	-0.59418	
6	-2.45316	2.17347	2.97302	
1	-1.44145	0.54164	2.00758	
6	-3.14554	3.37029	2.79927	
1	-3.94099	4.75637	1.35933	
1	-2.24879	1.80442	3.97328	
1	-3.48303	3.93838	3.65957	
6	1.61969	-3.11814	-0.52159	
6	1.33505	-2.28614	-1.61021	
6	1.58795	-4.50562	-0.67623	
6	1.03405	-2.84333	-2.84884	
1	1.41878	-1.20637	-1.49851	
6	1.25072	-5.05658	-1.90873	
1	1.80063	-5.15562	0.16580	
6	0.98211	-4.22770	-2.99628	
1	0.84525	-2.19536	-3.69811	
1	1.20651	-6.13394	-2.02307	
1	0.73890	-4.66219	-3.95971	
6	-2.83918	-0.07478	-0.83198	
1	-2.82432	-0.68687	0.07961	
1	-2.47419	-0.73588	-1.62863	
6	3.04960	-2.97351	1.60250	
1	3.28844	-4.01901	1.40141	
1	2.86579	-2.81711	2.66837	
8	-1.40245	-2.80368	0.54765	
6	-1.94900	-3.53965	-0.53547	
1	-1.40161	-3.23837	-1.42923	
1	-1.81824	-4.61454	-0.38956	
1	-3.01020	-3.31849	-0.68256	
6	-1.92157	-3.07747	1.86096	
6	-3.40588	-2.67561	2.02749	
6	-1.84327	-4.56640	2.25358	
1	-1.27739	-2.47733	2.50594	
6	-4.08477	-3.87517	2.70666	
1	-3.85159	-2.50832	1.04344	
1	-3.51268	-1.74493	2.58766	

6	-3.28656	-5.07731	2.19007
1	-1.13406	-5.11688	1.63159
1	-1.48541	-4.63017	3.28510
1	-3.97650	-3.80574	3.79378
1	-5.15161	-3.93426	2.48309
1	-3.44376	-5.98331	2.77841
1	-3.57238	-5.29551	1.15556
8	1.71865	2.06818	1.25022
6	1.42428	1.67846	2.57905
1	1.32773	0.59249	2.56992
1	2.22973	1.95537	3.26655
1	0.48622	2.11668	2.92800
6	1.90098	3.47037	1.00602
6	0.72214	4.33469	1.49456
6	3.15968	4.04347	1.69867
1	1.99799	3.51580	-0.08051
6	1.20572	4.97507	2.80052
1	-0.20090	3.75488	1.58357
1	0.54160	5.11946	0.75294
6	2.68434	5.26313	2.51003
1	3.59215	3.28009	2.35094
1	3.92739	4.30338	0.96773
1	0.64225	5.87155	3.06776
1	1.11695	4.26837	3.63174
1	2.76200	6.16892	1.90052
1	3.27655	5.42457	3.41319
6	3.90086	1.55938	-1.25014
6	2.72764	0.55451	-2.89401
6	4.13406	2.49550	-2.45749
1	4.75461	0.84217	-1.21483
1	3.90753	2.09278	-0.29261
6	3.49385	1.70594	-3.61970
1	3.30752	-0.38339	-3.05729
1	1.73753	0.37565	-3.33773
1	3.60370	3.43946	-2.29301
1	5.18861	2.73132	-2.63251
1	2.81879	2.33218	-4.20928
1	4.24895	1.31292	-4.30687
7	2.63031	0.91854	-1.49808
6	-0.76393	3.00918	-1.73202
6	-1.42452	1.14891	-3.05713
6	0.00607	3.03423	-3.06001
1	-0.08708	2.87398	-0.88143
1	-1.29618	3.95112	-1.56605
6	-0.88013	2.21355	-3.99984
1	-0.65717	0.37953	-2.87592
1	-2.31856	0.65590	-3.44768
1	0.19620	4.04880	-3.41585
1	0.96764	2.53252	-2.93264
1	-1.70262	2.82213	-4.38970
1	-0.33512	1.78976	-4.84624
7	-1.73086	1.89027	-1.82854
14	4.61784	-1.93686	1.15462
6	4.83389	-1.96875	-0.70345
1	5.80346	-1.54885	-0.98815
1	4.78681	-2.98909	-1.09780
1	4.05574	-1.37323	-1.19218
6	6.04650	-2.77357	2.03028
1	5.88399	-2.81558	3.11136
1	6.19107	-3.79702	1.67203
1	6.97883	-2.22734	1.85661
6	4.35048	-0.19856	1.79147
1	5.24232	0.41423	1.62336
1	3.51353	0.29037	1.28499

1	4.14778	-0.20325	2.86732
14	-4.67469	0.27065	-1.13119
6	-5.05847	1.59427	-2.40910
1	-6.13566	1.61227	-2.60898
1	-4.76680	2.59176	-2.07289
1	-4.55138	1.40348	-3.35946
6	-5.39050	-1.36291	-1.74771
1	-4.90397	-1.69402	-2.67114
1	-5.26758	-2.15890	-1.00527
1	-6.46232	-1.27511	-1.95389
6	-5.54582	0.67691	0.48856
1	-6.63219	0.68541	0.34807
1	-5.32151	-0.07910	1.24905
1	-5.25686	1.64990	0.89392

E= -2648.060039 (Hartree)

Solvent: **THF**

anti tetr-**1b**

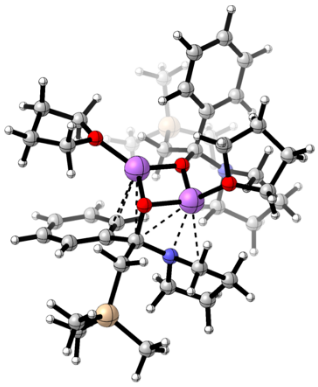

Atom	X	Y	Z	(Angstrom)
8	1.00074	-0.29983	0.34496	
8	-1.68133	-0.07765	1.37728	
3	-0.14269	0.92844	1.06696	
3	-0.46238	-1.34739	0.62735	
6	2.18779	-0.25053	-0.33799	
6	-2.62490	-0.06845	0.37258	
6	3.37861	-0.29366	0.64372	
6	4.57944	-0.95182	0.37066	
6	3.27418	0.41437	1.84392	
6	5.63818	-0.91955	1.27719	
1	4.68361	-1.50035	-0.55838	
6	4.33310	0.46424	2.74621	
1	2.34444	0.92592	2.07329	
6	5.52143	-0.20935	2.46864	
1	6.55779	-1.44893	1.04866	
1	4.22944	1.02336	3.67077	
1	6.34574	-0.18036	3.17334	
6	-2.45782	1.26624	-0.39365	
6	-1.31811	1.45854	-1.18693	
6	-3.29119	2.36729	-0.18609	
6	-1.03188	2.69142	-1.76420	
1	-0.63904	0.62503	-1.33490	
6	-3.01265	3.60557	-0.76539	
1	-4.17227	2.28140	0.43806	
6	-1.88452	3.77514	-1.56016	
1	-0.13990	2.80787	-2.37326	
1	-3.68287	4.44071	-0.58744	
1	-1.66790	4.73847	-2.00977	
6	2.28679	1.09411	-1.10790	
1	2.08200	1.87709	-0.36543	
1	1.44799	1.14472	-1.80888	

6	-4.04452	-0.21488	0.97907
1	-4.14661	0.59778	1.70726
1	-4.01656	-1.11454	1.60589
6	-2.61796	-2.55392	-0.05813
6	-2.75916	-1.20737	-1.93758
6	-2.09344	-3.43760	-1.18529
1	-3.70252	-2.69192	0.04007
1	-2.17096	-2.74968	0.91847
6	-2.49789	-2.64421	-2.44482
1	-3.83068	-0.97621	-1.98993
1	-2.23731	-0.45889	-2.53270
1	-1.00246	-3.51742	-1.11941
1	-2.50833	-4.44677	-1.16100
1	-1.71467	-2.66339	-3.20438
1	-3.40000	-3.06050	-2.89778
7	-2.27646	-1.20930	-0.54974
6	2.18080	-2.69699	-0.72231
6	1.37890	-1.36881	-2.41017
6	2.15959	-3.62747	-1.93538
1	1.23150	-2.76848	-0.17021
1	2.98279	-2.92593	-0.01980
6	1.54550	-2.75427	-3.05484
1	0.34966	-1.25082	-2.02763
1	1.57429	-0.55915	-3.11517
1	3.17615	-3.92824	-2.19827
1	1.58614	-4.53690	-1.74207
1	2.21513	-2.70150	-3.91551
1	0.58911	-3.14375	-3.40928
7	2.34475	-1.36567	-1.30827
14	-5.70496	-0.28536	0.05353
6	-5.92754	0.91231	-1.38670
1	-6.84047	0.64142	-1.92931
1	-6.05053	1.94285	-1.04273
1	-5.10285	0.90286	-2.10330
6	-6.99509	0.16334	1.35239
1	-8.00905	0.11044	0.94288
1	-6.95052	-0.51485	2.21084
1	-6.84531	1.18017	1.72989
6	-6.15095	-2.01400	-0.55706
1	-5.54817	-2.35861	-1.40131
1	-6.06421	-2.75802	0.24127
1	-7.19675	-2.01252	-0.88442
14	3.83933	1.66887	-2.01857
6	4.63338	0.38047	-3.13404
1	3.90511	-0.05398	-3.82589
1	5.42417	0.84289	-3.73511
1	5.08059	-0.44029	-2.56914
6	3.26119	3.09871	-3.10585
1	2.76058	3.87208	-2.51291
1	4.09875	3.57560	-3.62535
1	2.55292	2.75899	-3.86897
6	5.11264	2.35855	-0.81453
1	5.93376	2.84094	-1.35586
1	4.65923	3.11710	-0.16710
1	5.54627	1.59067	-0.16860
8	-0.05372	-2.76057	1.94773
6	0.76357	-2.26377	3.00982
6	0.15530	-4.17198	1.90083
6	2.07007	-3.04322	2.88841
1	0.87355	-1.18903	2.86727
1	0.26187	-2.46120	3.96502
6	1.62659	-4.39924	2.29376
1	-0.52429	-4.65234	2.61361
1	-0.09368	-4.51449	0.89518

1	2.57091	-3.14809	3.85152
1	2.74576	-2.52199	2.20797
1	1.70854	-5.20977	3.01882
1	2.23094	-4.66593	1.42585
8	0.10110	2.65597	1.80290
6	-0.99714	3.17867	2.58155
6	0.91754	3.73361	1.30674
6	-1.02168	4.67165	2.29082
1	-1.90172	2.64639	2.28437
1	-0.79320	2.97785	3.63772
6	0.46278	4.97033	2.07008
1	1.96489	3.47464	1.47348
1	0.73857	3.83759	0.23167
1	-1.46211	5.24255	3.10878
1	-1.58881	4.86948	1.37758
1	0.98412	5.03864	3.02860
1	0.64370	5.88765	1.50931

E= -2490.890637 (Hartree)

syn tetr-**1b**

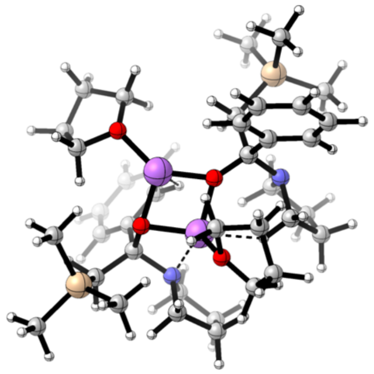

Atom	X	Y	Z	(Angstrom)
8	0.84225	-0.16353	0.24185	
8	-1.93980	-0.06008	0.62974	
3	-0.40412	0.72926	1.22620	
3	-0.64549	-1.11766	-0.28650	
6	2.16739	-0.05116	-0.09581	
6	-2.74550	0.26122	-0.43329	
6	3.04139	-0.90500	0.85157	
6	4.12951	-1.66755	0.42345	
6	2.76464	-0.86687	2.22105	
6	4.90459	-2.38781	1.33274	
1	4.37140	-1.69916	-0.63259	
6	3.53945	-1.57335	3.13511	
1	1.92295	-0.27707	2.57169	
6	4.61402	-2.34419	2.69263	
1	5.73953	-2.98184	0.97479	
1	3.30482	-1.52600	4.19381	
1	5.21720	-2.90151	3.40151	
6	-2.46041	1.69935	-0.94849	
6	-1.13131	2.12437	-1.05400	
6	-3.45699	2.57624	-1.38352	
6	-0.80792	3.37474	-1.57285	
1	-0.32906	1.44940	-0.76929	
6	-3.14014	3.83520	-1.89633	
1	-4.49965	2.28165	-1.34771	
6	-1.81375	4.24208	-1.99495	
1	0.23482	3.66923	-1.65543	
1	-3.93674	4.49444	-2.22585	
1	-1.56674	5.21826	-2.39854	
6	2.62125	1.42190	0.07618	
1	2.35521	1.69415	1.10638	

1	1.98318	2.04931	-0.55816
6	-4.23129	0.09355	-0.03276
1	-4.87851	-0.15216	-0.88331
1	-4.60311	1.05790	0.33730
6	-2.88435	-2.01708	-1.54467
6	-2.65115	-0.21889	-2.95466
6	-2.33474	-2.63241	-2.83214
1	-3.98534	-2.01582	-1.57361
1	-2.56698	-2.53944	-0.64600
6	-2.29169	-1.44026	-3.81648
1	-3.72270	0.02031	-3.05540
1	-2.08332	0.66957	-3.22850
1	-1.32714	-3.01896	-2.65986
1	-2.95146	-3.46033	-3.18620
1	-1.29372	-1.32538	-4.24445
1	-2.99187	-1.55851	-4.64497
7	-2.34665	-0.64947	-1.58291
6	1.89045	-1.78263	-1.84705
6	1.95869	0.38142	-2.55836
6	2.25131	-1.91297	-3.32724
1	0.79150	-1.78592	-1.74882
1	2.27864	-2.58413	-1.21510
6	2.20963	-0.44828	-3.82976
1	0.87979	0.58123	-2.44366
1	2.48176	1.33812	-2.57712
1	3.25557	-2.32851	-3.43623
1	1.55965	-2.56736	-3.86208
1	3.16091	-0.16984	-4.28758
1	1.42733	-0.28284	-4.57250
7	2.44741	-0.48308	-1.48488
14	-4.57192	-1.02102	1.46008
6	-3.83828	-2.75194	1.52653
1	-4.12552	-3.20931	2.48059
1	-4.21006	-3.40345	0.73062
1	-2.74779	-2.72913	1.47521
6	-6.45111	-1.23470	1.48312
1	-6.77891	-1.80056	2.36161
1	-6.96800	-0.26930	1.50455
1	-6.80414	-1.77359	0.59718
6	-4.09717	-0.14227	3.05584
1	-3.01126	-0.07810	3.15494
1	-4.50165	0.87444	3.09760
1	-4.49067	-0.68505	3.92245
14	4.42385	1.96478	-0.12392
6	5.32243	1.26657	-1.62079
1	4.79861	1.48677	-2.55551
1	6.31973	1.71542	-1.69072
1	5.44944	0.18357	-1.55807
6	4.36163	3.84120	-0.30120
1	3.83428	4.30255	0.54084
1	5.36470	4.27806	-0.33993
1	3.83866	4.13659	-1.21711
6	5.40715	1.56412	1.43130
1	6.40338	2.01713	1.38371
1	4.90938	1.96112	2.32238
1	5.53854	0.48833	1.57833
8	-0.45815	-2.99305	0.31118
6	0.07820	-3.05298	1.64238
6	-0.22290	-4.24258	-0.36054
6	1.20730	-4.06391	1.55286
1	0.39584	-2.04599	1.90836
1	-0.70424	-3.39220	2.33201
6	0.59899	-5.10344	0.60539
1	-1.18546	-4.69410	-0.61621

1	0.32188	-4.03145	-1.28426
1	1.47995	-4.47573	2.52540
1	2.09108	-3.59538	1.10996
1	-0.05402	-5.78030	1.16150
1	1.34560	-5.70136	0.08209
8	-0.58236	2.37014	2.18084
6	-1.95849	2.75049	2.40007
6	0.22596	3.54284	1.95419
6	-2.06807	4.17162	1.87403
1	-2.58459	2.02756	1.87629
1	-2.16354	2.69981	3.47472
6	-0.68682	4.73731	2.21362
1	1.08834	3.50178	2.62232
1	0.57594	3.52214	0.91879
1	-2.88488	4.72228	2.34212
1	-2.22425	4.16007	0.79253
1	-0.64426	5.02901	3.26621
1	-0.41053	5.59866	1.60432

E= -2490.898009 (Hartree)

3b (from *syn tetr*-**1b**)

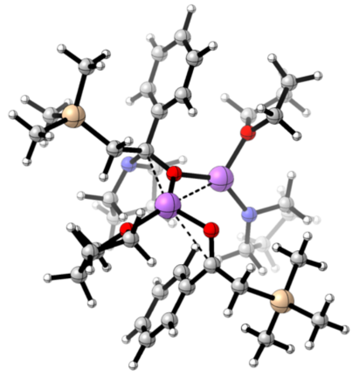

Atom	X	Y	Z	(Angstrom)
8	0.88701	-0.00445	0.16020	
8	-1.91734	0.42961	1.68894	
3	-0.32454	1.12336	0.85013	
3	0.18393	-1.71356	-0.04162	
6	2.14519	0.44493	-0.20763	
6	-3.01733	0.80087	1.28488	
6	3.24424	-0.49698	0.31992	
6	4.38091	-0.83905	-0.41383	
6	3.14131	-0.96256	1.63291	
6	5.38465	-1.63133	0.14555	
1	4.48753	-0.47991	-1.43023	
6	4.14273	-1.74081	2.20140	
1	2.25291	-0.71480	2.20416	
6	5.27220	-2.08318	1.45630	
1	6.25714	-1.89058	-0.44577	
1	4.04380	-2.08609	3.22617	
1	6.05403	-2.69433	1.89524	
6	-3.13053	1.39264	-0.08564	
6	-2.34719	0.84232	-1.10469	
6	-4.00134	2.44801	-0.36917	
6	-2.44967	1.33518	-2.40169	
1	-1.69882	-0.00579	-0.89185	
6	-4.07533	2.95826	-1.66095	
1	-4.60980	2.88677	0.41439	
6	-3.30785	2.39635	-2.67957	
1	-1.86477	0.88470	-3.19647	
1	-4.73892	3.78869	-1.87536	
1	-3.38291	2.78280	-3.69011	

6	2.41119	1.83322	0.43251
1	2.20106	1.72682	1.50617
1	1.66024	2.53804	0.06153
6	-4.24974	0.57634	2.06208
1	-4.96641	1.39491	1.96119
1	-4.00968	0.41423	3.11521
6	-1.37004	-4.02712	-0.72191
6	-2.14883	-2.43168	-2.07355
6	-1.39902	-4.73595	-2.10160
1	-2.33962	-4.24820	-0.21976
1	-0.58338	-4.41509	-0.06807
6	-1.95176	-3.63519	-3.03555
1	-3.20785	-2.45791	-1.71715
1	-2.02386	-1.47613	-2.59201
1	-0.38719	-5.02834	-2.39797
1	-2.01655	-5.63983	-2.10087
1	-1.22483	-3.37850	-3.81150
1	-2.87871	-3.93131	-3.53670
7	-1.20557	-2.61428	-0.99638
6	2.02788	-0.69876	-2.40260
6	1.41961	1.60095	-2.26240
6	0.88789	-0.40488	-3.37734
1	1.77869	-1.51161	-1.71610
1	2.92770	-1.00496	-2.94908
6	1.08909	1.08371	-3.65774
1	0.48300	1.70349	-1.69179
1	1.91808	2.57499	-2.27411
1	0.91350	-1.03691	-4.26720
1	-0.06995	-0.56073	-2.87100
1	1.93920	1.23336	-4.33155
1	0.21738	1.57778	-4.09223
7	2.28711	0.56801	-1.67590
14	-5.14944	-0.99871	1.40190
6	-5.87630	-0.63610	-0.28702
1	-6.47757	-1.48791	-0.62216
1	-6.53120	0.24067	-0.26696
1	-5.10402	-0.46403	-1.04203
6	-6.51376	-1.35307	2.63588
1	-6.10915	-1.54754	3.63341
1	-7.21097	-0.51348	2.71439
1	-7.08959	-2.23363	2.33477
6	-3.90940	-2.38960	1.31484
1	-4.39777	-3.30912	0.97711
1	-3.08502	-2.17357	0.62274
1	-3.46839	-2.59143	2.29671
14	4.09195	2.69792	0.34367
6	4.90411	2.70844	-1.35040
1	5.77938	3.36762	-1.33529
1	5.24150	1.71540	-1.65484
1	4.22589	3.07975	-2.12418
6	3.74231	4.48964	0.82186
1	3.11763	4.98599	0.07144
1	3.21718	4.55353	1.78092
1	4.66616	5.06997	0.91359
6	5.28676	1.98653	1.61242
1	6.19486	2.59668	1.66833
1	4.83734	1.97643	2.61100
1	5.58870	0.96268	1.37513
8	1.24895	-3.10312	0.80865
6	2.08985	-3.89683	-0.03847
6	1.16131	-3.68124	2.11587
6	2.20771	-5.24442	0.66099
1	1.61794	-3.94965	-1.02284
1	3.06351	-3.40197	-0.12940

6	2.16690	-4.82971	2.13407
1	1.37687	-2.90688	2.85578
1	0.13667	-4.03797	2.26703
1	3.11986	-5.77543	0.38674
1	1.34835	-5.87492	0.41617
1	3.14943	-4.46904	2.44841
1	1.86008	-5.63415	2.80336
8	-0.71138	3.01292	0.94351
6	-1.33095	3.60277	2.09797
6	-0.74472	3.94374	-0.16023
6	-1.23562	5.10036	1.86353
1	-2.37864	3.28139	2.15249
1	-0.80291	3.24681	2.98333
6	-1.48173	5.17796	0.35496
1	0.28825	4.17565	-0.43497
1	-1.24109	3.46531	-1.00657
1	-0.23243	5.45769	2.11073
1	-1.96384	5.66029	2.45066
1	-1.10742	6.09513	-0.09983
1	-2.55116	5.09745	0.14385

E= -2490.853520 (Hartree)

References

(1) Kofron, W. G.; Baclawski, L. M. A Convenient Method for Estimation of Alkyllithium Concentrations. *J. Org. Chem.* **1976**, *41*, 1879–1880.

(2) Jakhar, V. K.; Johnson, E. C.; Kavuturu, A.; Heller, J. K.; Veige, A. S.; Ghiviriga, I. Precise NMR Method for Titering Organometal Reagents. *Org. Lett.* **2021**, *23*, 4945–4948.

(3) Xu, L.; Zhang, S.; Li, W.; Zhang, Z. Visible-Light-Mediated Oxidative Amidation of Aldehydes by Using Magnetic CdS Quantum Dots as a Photocatalyst. *Chem. Eur. J.* **2021**, *27*, 5483–5491.

(4) Ghinato, S.; Territo, D.; Maranzana, A.; Capriati, V.; Blangetti, M.; Prandi, C. A Fast and General Route to Ketones from Amides and Organolithium Compounds under Aerobic Conditions: Synthetic and Mechanistic Aspects. *Chem. Eur. J.* **2021**, *27*, 2868–2874.

(5) Zhou, L.; Qiu, J.; Wang, C.; Zhang, F.; Yang, K.; Song, Q. Synthesis of α-Aminosilanes by 1,2-Metalate Rearrangement Deoxygenative Silylation of Aromatic Amides. *Org. Lett.* **2022**, *24*, 3249–3253.

(6) Zhang, M.-J.; Li, H.-X.; Young, D. J.; Li, H.-Y.; Lang, J.-P. Reaction Condition Controlled Nickel(II)-Catalyzed C–C Cross-Coupling of Alcohols. *Org. Biomol. Chem.* **2019**, *17*, 3567–3574.

(7) Zhou, Y.; Li, Z.; Yang, X.; Chen, X.; Li, M.; Chen, T.; Yin, S.-F. Phosphorous Acid Promoted Hydration–Condensation of Aromatic Alkynes with Aldehydes Affording Chalcones in an Oil/Water Two-Phase System. *Synthesis* **2015**, *48*, 231–237.

(8) Uehara, A.; Olivero, S.; Michelet, B.; Martin-Mingot, A.; Thibaudeau, S.; Duñach, E. Direct and Selective C-H Carbamoylation of (Hetero)Aromatics with TMSOTf-Activated Carbamoyl Chloride: Direct and Selective C-H Carbamoylation of (Hetero)Aromatics with TMSOTf-Activated Carbamoyl Chloride. *Eur. J. Org. Chem.* **2019**, *2019*, 46–49.

(9) Spieß, P.; Berger, M.; Kaiser, D.; Maulide, N. Direct Synthesis of Enamides via Electrophilic Activation of Amides. *J. Am. Chem. Soc.* **2021**, *143*, 10524–10529.

(10) Chardon, A.; Mohy El Dine, T.; Legay, R.; De Paolis, M.; Rouden, J.; Blanchet, J. Borinic Acid Catalysed Reduction of Tertiary Amides with Hydrosilanes: A Mild and Chemoselective Synthesis of Amines. *Chem. Eur. J.* **2017**, *23*, 2005–2009.

(11) Gao, P.-S.; Zhang, K.; Yang, M.-M.; Xu, S.; Sun, H.-M.; Zhang, J.-L.; Gao, Z.-W.; Zhang, W.-Q.; Xu, L.-W. A Robust Multifunctional Ligand-Controlled Palladium-Catalyzed Carbonylation Reaction in Water. *Chem. Commun.* **2018**, *54*, 5074–5077.

(12) Wang, J.; Liu, C.; Yuan, J.; Lei, A. Copper-Catalyzed Oxidative Coupling of Alkenes with Aldehydes: Direct Access to α,β-Unsaturated Ketones. *Angew. Chem. Int. Ed.* **2013**, *52*, 2256–2259.

(13) Meng, G.; Szostak, M. Palladium-Catalyzed Suzuki–Miyaura Coupling of Amides by Carbon–Nitrogen Cleavage: General Strategy for Amide N–C Bond Activation. *Org. Biomol. Chem.* **2016**, *14*, 5690–5707.

(14) Shaykhutdinova, P.; Oestreich, M. Achieving Enantioselectivity in Difficult Cyclohexa-1,3-Diene Diels–Alder Reactions with Sulfur-Stabilized Silicon Cations as Lewis Acid Catalysts. *Org. Lett.* **2018**, *20*, 7029–7033.

(15) Ishikura, M.; Terashima, M. Palladium-Catalyzed Carbonylative Cross-Coupling Reaction with Triethyl(1-Methylindol-2-Yl)Borate: A Simple Route to 1-Methyl-2-Indolyl Ketones. *J. Org. Chem.* **1994**, *59*, 2634–2637.

(16) Pierce, B. M.; Simpson, B. F.; Ferguson, K. H.; Whittaker, R. E. Phosphine-Mediated Partial Reduction of Alkynes to Form Both (*E*)- and (*Z*)-Alkenes. *Org. Biomol. Chem.* **2018**, *16*, 6659–6662.

(17) Sumino, S.; Ui, T.; Hamada, Y.; Fukuyama, T.; Ryu, I. Carbonylative Mizoroki–Heck Reaction of Alkyl Iodides with Arylalkenes Using a Pd/Photoirradiation System. *Org. Lett.* **2015**, *17*, 4952–4955.

(18) Ramar, T.; Subbaiah, M. A. M.; Ilangovan, A. Orchestrating a β-Hydride Elimination Pathway in Palladium(II)-Catalyzed Arylation/Alkenylation of Cyclopropanols Using Organoboron Reagents. *J. Org. Chem.* **2022**, *87*, 4508–4523.

(19) Unoh, Y.; Hirano, K.; Satoh, T.; Miura, M. Palladium-Catalyzed Decarboxylative Arylation of Benzoylacrylic Acids toward the Synthesis of Chalcones. *J. Org. Chem.* **2013**, *78*, 5096–5102.

(20) Sheng, W.; Cui, J.; Ruan, Z.; Yan, L.; Wu, Q.; Yu, C.; Wei, Y.; Hao, E.; Jiao, L. [*A*]-Phenanthrene-Fused BF_2 Azadipyrromethene (AzaBODIPY) Dyes as Bright Near-Infrared Fluorophores. *J. Org. Chem.* **2017**, *82*, 10341–10349.

(21) Gao, P.; Yan, D.; Bi, M.; Jiang, M.; Xiao, W.; Chen, J. Alkene Synthesis by Photo-Wolff-Kischner Reaction of Sulfur Ylides and *N*-Tosylhydrazones. *Chem. Eur. J.* **2021**, *27*, 14195–14201.

(22) Wang, J.; Lei, M.; Li, Q.; Ge, Z.; Wang, X.; Li, R. A Novel and Efficient Direct Aldol Condensation from Ketones and Aromatic Aldehydes Catalyzed by Proline–TEA through a New Pathway. *Tetrahedron* **2009**, *65*, 4826–4833.

(23) Roman, B. I.; De Ryck, T.; Verhasselt, S.; Bracke, M. E.; Stevens, C. V. Further Studies on Anti-Invasive Chemotypes: An Excursion from Chalcones to Curcuminoids. *Bioorg. Med. Chem. Lett.* **2015**, *25*, 1021–1025.

(24) Demidoff, F. C.; Caleffi, G. S.; Figueiredo, M.; Costa, P. R. R. Ru(II)-Catalyzed Asymmetric Transfer Hydrogenation of Chalcones in Water: Application to the Enantioselective Synthesis of Flavans BW683C and Tephrowatsin E. *J. Org. Chem.* **2022**, *87*, 14208–14222.

(25) Davies, A. M.; D. Hernandez, R.; Tunge, J. A. Direct Aroylation of Olefins through a Cobalt/Photoredox-Catalyzed Decarboxylative and Dehydrogenative Coupling with A-Oxo Acids. *Chem. – Eur. J.* **2022**, *28*.

(26) Hintermann, L.; Läng, F.; Maire, P.; Togni, A. Interactions of Cationic Palladium(II)- and Platinum(II)-η^3-Allyl Complexes with Fluoride: Is Asymmetric Allylic Fluorination a Viable Reaction? *Eur. J. Inorg. Chem.* **2006**, *2006*, 1397–1412.

(27) Valdameri, G.; Gauthier, C.; Terreux, R.; Kachadourian, R.; Day, B. J.; Winnischofer, S. M. B.; Rocha, M. E. M.; Frachet, V.; Ronot, X.; Di Pietro, A.; Boumendjel, A. Investigation of Chalcones as Selective Inhibitors of the Breast Cancer Resistance Protein: Critical Role of Methoxylation in Both Inhibition Potency and Cytotoxicity. *J. Med. Chem.* **2012**, *55*, 3193–3200.

(28) Ducki, S.; Rennison, D.; Woo, M.; Kendall, A.; Chabert, J. F. D.; McGown, A. T.; Lawrence, N. J. Combretastatin-like Chalcones as Inhibitors of Microtubule Polymerization. Part 1: Synthesis and Biological Evaluation of Antivascular Activity. *Bioorg. Med. Chem.* **2009**, *17*, 7698–7710.

(29) Kumar, V.; Kumar, S.; Hassan, M.; Wu, H.; Thimmulappa, R. K.; Kumar, A.; Sharma, S. K.; Parmar, V. S.; Biswal, S.; Malhotra, S. V. Novel Chalcone Derivatives as Potent Nrf2 Activators in Mice and Human Lung Epithelial Cells. *J. Med. Chem.* **2011**, *54*, 4147–4159.

(30) Sun, C.; Li, J.; Demerzhan, S.; Lee, D. Mild and Efficient Catalytic Method for α-Trimethylsilyl Ketones. *Arkivoc* **2011**, *2011*, 17–25.

(31) Gaussian 16, Revision B.01, Frisch, M. J.; Trucks, G. W.; Schlegel, H. B.; Scuseria, G. E.; Robb, M. A.; Cheeseman, J. R.; Scalmani, G.; Barone, V.; Petersson, G. A.; Nakatsuji, H.; Li, X.; Caricato, M.; Marenich, A. V.; Bloino, J.; Janesko, B. G.; Gomperts, R.; Mennucci, B.; Hratchian, H. P.; Ortiz, J. V.; Izmaylov, A. F.; Sonnenberg, J. L.; Williams-Young, D.; Ding, F.; Lipparini, F.; Egidi, F.; Goings, J.; Peng, B.; Petrone, A.; Henderson, T.; Ranasinghe, D.; Zakrzewski, V. G.; Gao, J.; Rega, N.; Zheng, G.; Liang, W.; Hada, M.; Ehara, M.; Toyota, K.; Fukuda, R.; Hasegawa, J.; Ishida, M.; Nakajima, T.; Honda, Y.; Kitao, O.; Nakai, H.; Vreven, T.; Throssell, K.; Montgomery, J. A., Jr.; Peralta, J. E.; Ogliaro, F.; Bearpark, M. J.; Heyd, J. J.; Brothers, E. N.; Kudin, K. N.; Staroverov, V. N.; Keith, T. A.; Kobayashi, R.; Normand, J.; Raghavachari, K.; Rendell, A. P.; Burant, J. C.; Iyengar, S. S.; Tomasi, J.; Cossi, M.; Millam, J. M.; Klene, M.; Adamo, C.; Cammi, R.; Ochterski, J. W.; Martin, R. L.; Morokuma, K.; Farkas, O.; Foresman, J. B.; Fox, D. J. Gaussian, Inc., Wallingford CT, **2016**.

(32) Zhao, Y.; Truhlar, D. G. The M06 Suite of Density Functionals for Main Group Thermochemistry, Thermochemical Kinetics, Noncovalent Interactions, Excited States, and Transition Elements: Two New Functionals and Systematic Testing of Four M06-Class Functionals and 12 Other Functionals. *Theor. Chem. Acc.* **2008**, *120*, 215–241.

(33) McLean, A. D.; Chandler, G. S. Contracted Gaussian Basis Sets for Molecular Calculations. I. Second Row Atoms, Z =11–18. *J. Chem. Phys.* **1980**, *72*, 5639–5648.

(34) Krishnan, R.; Binkley, J. S.; Seeger, R.; Pople, J. A. Self-consistent Molecular Orbital Methods. XX. A Basis Set for Correlated Wave Functions. *J. Chem. Phys.* **1980**, *72*, 650–654.

(35) Marenich, A. V.; Cramer, C. J.; Truhlar, D. G. Universal Solvation Model Based on Solute Electron Density and on a Continuum Model of the Solvent Defined by the Bulk Dielectric Constant and Atomic Surface Tensions. *J. Phys. Chem. B* **2009**, *113*, 6378–6396.

(36) Tomasi, J.; Mennucci, B.; Cammi, R. Quantum Mechanical Continuum Solvation Models. *Chem. Rev.* **2005**, *105*, 2999–3094.
